# Early Weaning Possibly Increases the Activity of Lipogenic and Adipogenic Pathways in Intramuscular Adipose Tissue of Nellore Calves

**DOI:** 10.3390/metabo13091028

**Published:** 2023-09-21

**Authors:** Ariane Enara Pedro, Juliana Akamine Torrecilhas, Rodrigo Nazaré Santos Torres, Germán Darío Ramírez-Zamudio, Welder Angelo Baldassini, Luis Artur Loyola Chardulo, Rogério Abdallah Curi, Gustavo Henrique Russo, Juliane Arielly Napolitano, Gustavo Lucas Bezerra Tinoco, Thiago Barcaça Mariano, Jordana Luiza Caixeta, Philipe Moriel, Guilherme Luis Pereira

**Affiliations:** 1 College of Agronomics and Veterinary Sciences, University of São Paulo State Júlio de Mesquita Filho, Jaboticabal 14884-900, Brazil; ariane.enara@unesp.br (A.E.P.); gustavo.russo@unesp.br (G.H.R.); gustavo.tinoco@unesp.br (G.L.B.T.); 2 College of Veterinary and nimal Science, University of São Paulo State Júlio de Mesquita Filho, Botucatu 18618-687, Brazil; juliana.akamine@unesp.br (J.A.T.); rodrigo.chaves@unesp.br (R.N.S.T.); w.baldassini@unesp.br (W.A.B.); luis.artur@unesp.br (L.A.L.C.); rogerio.curi@unesp.br (R.A.C.); 3 College of Animal Science and Foods Engineering, University of São Paulo, Pirassununga 13635-900, Brazil; germanramvz@gmail.com; 4 College of Agronomic Science, University of São Paulo State Júlio de Mesquita Filho, Botucatu 18610-034, Brazil; juliane.arielly@unesp.br (J.A.N.); thiago.barcaca@unesp.br (T.B.M.); jordana.caixeta@unesp.br (J.L.C.); 5 Institute of Food and Agricultural Sciences, University of Florida, Gainesville, FL 32603, USA; pmoriel@ufl.edu

**Keywords:** gene expression, functional enrichment, RNA-Seq

## Abstract

This study aimed to evaluate by wide-expression profile analysis how early weaning at 120 days can alter the skeletal muscle metabolism of calves supplemented with a concentrated diet until the growth phase. *Longissimus thoracis* muscle samples were obtained by biopsy from two groups of calves, early weaned (EW; n = 8) and conventionally weaned (CW; n = 8) at two different times (120 days of age—T1 [EW] and 205 days of age—T2 [CW]). Next, differential gene expression analysis and functional enrichment of metabolic pathways and biological processes were performed. The results showed respectively 658 and 165 differentially expressed genes when T1 and T2 were contrasted in the early weaning group and when early and conventionally weaned groups were compared at T2. The *FABP4*, *SCD1*, *FASN*, *LDLR*, *ADIPOQ*, *ACACA*, *PPARD*, and *ACOX3* genes were prospected in both comparisons described above. Given the key role of these differentially expressed genes in lipid and fatty acid metabolism, the results demonstrate the effect of diet on the modulation of energy metabolism, particularly favoring postnatal adipogenesis and lipogenesis, as well as a consequent trend in obtaining better quality cuts, as long as an environment for the maintenance of these alterations until adulthood is provided.

## 1. Introduction

Zebu breeds, including Nellore cattle, are tolerant to adverse environmental conditions such as heat, humidity, and endo- and ectoparasites typical of tropical climates, maintaining reasonable production rates [1]. However, the productive and meat quality indices of these breeds are lower compared to taurine cattle [2], these traits being complex and of low heritability because environmental factors mainly influence them or because they are difficult to measure [3,4]. Although it is a challenge, zebu breeders around the world have been using improved tools such as the use of massive information from molecular markers for genetic selection and reproductive biotechnologies in an attempt to improve production rates and the value of products in the international market [2].

Other strategies that directly influence the performance of animals correspond to nutritional management in the different stages of growth and include offering concentrate via creep-feeding [5] to providing different sources and levels of supplementation during the rearing phase [6]. Creep-feeding as a nutritional strategy not only improves weight gain and carcass quality by slaughtering animals earlier [5] but can also have indirect positive effects on dam performance, as it can reduce the production of milk due to a lower sucking stimulation of the calves caused by the replacement with concentrate [7,8]. In addition to the use of creep-feeding, early weaning as a management strategy for cows, mainly primiparous, aims to reduce the negative impact of lactation on nutritional status, allowing recovery of body condition for the next breeding season [9,10,11].

Traditionally, Nellore cows, the basis of Brazil’s breeding herd, are weaned approximately at the seventh month of lactation [12]. However, milk production after the third month of lactation does not meet the nutritional requirements to express the genetic potential of the offspring [13]. As for calves, early weaning per se has shown negative responses on post-weaning growth [14,15]. Therefore, nutrition after this type of management has to be adequate not to compromise the calves’ growth [11,16,17]. Studies indicate that early weaning associated with adequate nutritional management can lead to accelerated animal growth in the subsequent production [18,19,20], changes in skeletal muscle energy metabolism [21], and cellular dynamics in adipose tissue [22,23,24], thus improving meat quality. Although some results are still conflicting, the magnitude of metabolic modulation is directly related to the level and balance of nutrients in diets offered to calves [25]. However, research to understand skeletal muscle tissue metabolism affected by management strategies such as early weaning and nutritional plans applied during the growth phase of calves is limited. Therefore, zebu cattle need to be improved.

It is hypothesized that early weaning attended by moderate supplementation with concentrate until the traditional weaning time for beef cattle does not affect the growth of calves. However, it alters the gene regulation that regulates metabolic pathways related to fat deposition in skeletal muscle tissue, improving meat quality. Therefore, this study aimed to evaluate the effect of early weaning associated with a post-weaning supplementation protocol and compare it with traditional weaning on growth, changes in biological processes, and metabolic pathways regulated by gene expression profiles in skeletal muscle tissue.

## 2. Materials and Methods

### 2.1. Samples and Weight Recording

**Figure 1:**
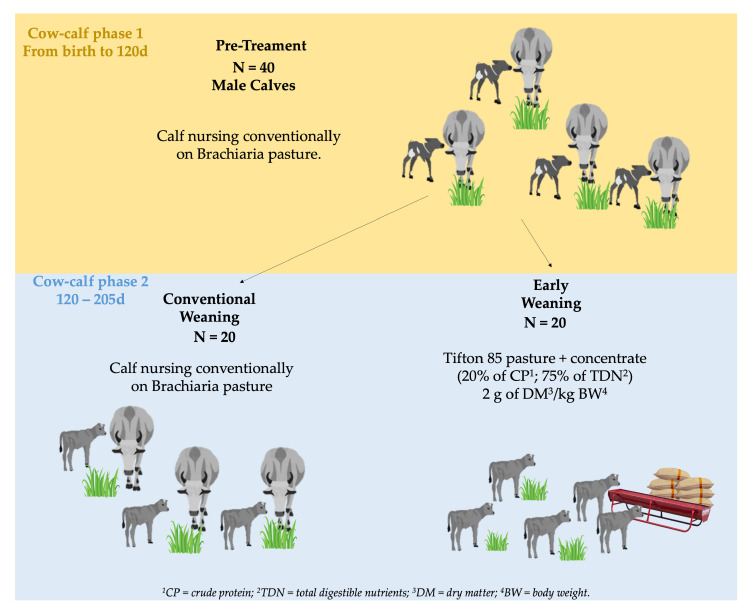
Supplementation and diets consisting of natural forage, soybean meal, corn, additives, and minerals are offered to Nellore cattle at different developmental stages and subjected to different weaning protocols.

Forty male Nellore calves born in the same month to contemporary cows of the same calving order were used. A field experiment was carried out on a duly regularized commercial farm, located in the State of Mato Grosso (Pantanal biome), Brazil. The animals were submitted to two different treatments, in which 20 male calves were kept with their respective dams in the same paddock on *Brachiaria* spp. pasture from birth until 205 days of age (~30 weeks) (Time point 2—T2 or Moment 2—M2) when they were conventionally weaned (CW), and 20 males calves were weaned early (EW) at 120 days of age (~16 weeks) (Time point 1—T1 or moment—M2), reassigned to the same paddock with Tifton 85 pasture and supplemented with concentrate (Figure 1) until 205 days of age (~30 weeks).

All calves were weighed after a 6 h fast at the beginning of the early weaning phase at 120 days (initial body weight, BWi) and at conventional weaning, at 205 days of age (weaning weight, WW). Average daily weight gain (ADG) was then calculated based on BWi and WW for both groups.

### 2.2. Growing Performance Statistics

To detect significant differences in weights between treatments and time points, fulfilling all assumptions, analysis of variance (ANOVA) was conducted using a linear model with random residuals, in which the fixed effects of treatment (EW and CW), the time point of weaning (T1 and T2), and their interaction on the response variable (WW) were tested. Significant differences between means of specific groups were evaluated using the Tukey test for multiple comparisons at a level of significance of 5%. The difference in ADG between treatments (EW and CW) was obtained using an independent t-test after observation of a normal distribution of the ADG records and homogeneity of variances between the two groups.

### 2.3. Collection of Muscle Tissue and Total RNA Extraction

About 1 g samples of the *Longissimus thoracis* (LT) muscle were collected by biopsy between the twelfth and thirteenth rib during early and conventional weaning. For this purpose, a local anesthetic was administered subcutaneously, and the site was shaved and cleaned. Next, a 1 cm incision was made with a scalpel blade, and a sterile biopsy needle was used to obtain 1 g of muscle tissue. The sample was immediately transferred to liquid nitrogen and stored in an ultra-freezer at −80 °C.

Total RNA was extracted individually from 100 mg of LT muscle using TRIzol^®^ (Life Technologies, Carlsbad, CA, USA) in accordance with the manufacturer’s instructions, and its quality was analyzed in a Bioanalyzer 2100^®^ (Agilent, Santa Clara, CA, USA). To ensure adequate total RNA quality, only samples with an RNA integrity number (RIN) ≥ 7 were used for sequencing.

### 2.4. RNA Sequencing

After total RNA extraction, the isolated mRNA was submitted to purification and fragmentation using oligo-dT beads for the construction of 32 cDNA libraries. The libraries consisted of eight samples of each treatment (CW and EW) collected at T1 and T2, respectively. The cDNA libraries for each sample were prepared and multiplexed from 2 µg total RNA using the Illumina Stranded mRNA Prep kit (Illumina, San Diego, CA, USA) according to the Illumina Stranded mRNA Prep, Ligation Reference Guide (Illumina, USA). The average size of the libraries was determined by quantitative PCR (RT-qPCR) using the KAPA Library Quantification kit (KAPA Biosystems, Wilmington, MA, USA) in a Bioanalyzer 2100^®^ (Agilent, Santa Clara, CA, USA). Clustering and sequencing were performed with the NextSeq 550^®^ System (Illumina, USA) in order to produce 150 bp paired-end (PE) reads, with an expected average coverage of 10 million PE reads per sample.

### 2.5. Mapping of Sequences to the Reference Genome

The pipeline indicating the steps and programs used to obtain the count matrix from transcriptome data is illustrated in Figure S1. First, FastQC v. 0.11.9 [26] was used to analyze the quality of raw reads. Sequencing adapters and low-quality sequences were removed using the fastp v.0.20.0 program [27], ignoring reads and quality scores of poly-G segments, characteristic of null reads. After this step, the quality of the reads was reassessed by joint graphic visualization of all FastQC outputs using the MultiQC v.1.13 program [28]. The reads were then mapped to the bovine reference genome (Bos taurus—ARS-UCD 1.2), available at http://www.ensembl.org/Bos_taurus/Info/Index/ (accessed on 24 September 2022), using STAR v.2.7.20 [29]. The reads were mapped independently for each sample and only sequences that mapped uniquely to the genome and to known chromosomes were used in the analysis of differential gene expression. Considering the generated sequences (aligned PE reads), the number of mapped sequences per gene (exons) was determined to generate a count matrix (genes × samples) using the feature counts v.2.0.3 software [30].

### 2.6. Identification of Differentially Expressed Genes

The edgeR v.3.38.1 package [31] of R v.4.2.1 [32] was used to identify differentially expressed genes (DEGs). First, for quality control and consistency analysis of the count data, gene expression levels were obtained by adjusting the counts to the size of the library of each sample based on counts per million (CPM) and then normalized by log2 transformation [log2(CPM)]. Genes with low CPM were removed according to the default option of the edgeR v.3.38.1 package [31] and principal component analysis (PCA) was performed using the factoextra v.1.0.7 package [33] of R v.4.2.1 [32]. To estimate the effects of the treatments on gene expression, normalization factors (size factors) were calculated for each sample using the trimmed mean of M-values (TMM) method. The negative binomial distribution hyperparameter was estimated using the robust empirical Bayes algorithm. A generalized linear model was then adjusted assuming a negative binomial distribution and quasi-likelihood was used for coefficients inference. The significance of the effect of treatment on gene expression was obtained as the likelihood ratio between the reduced and complete models for each gene, assuming that the values of each test belong to a χ2 distribution. Next, the *p*-values were adjusted by the Benjamini–Hochberg method to correct the false-discovery rate (FDR) for multiple tests. Finally, the relative expression of each gene was obtained based on the logarithmic function of the ratio between the CPM of the different contrasts, as follows: $log2\left(\frac{mean\left(CPM\_A\right)}{mean\left(CPM\_B\right)}\right)=log2\left(FC\right)$, where FC is the fold change between contrasts. A $log2\left(FC\right)$ value ≤ 0.5 or ≥0.5, and an adjusted *p*-value < 0.05 were defined as the thresholds for the detection of DEGs between the different contrasts (EW vs. CW at T1; EW vs. CW at T2; T1 vs. T2 in EW; T1 vs. T2 in CW). The terms “upregulated” and “downregulated” refer to EW compared to CW and to T2 compared to T1.

### 2.7. Enrichment of Functional Terms

Enrichment of biological processes (BP) belonging to gene ontology (GO) terms and metabolic pathways annotated in the Kyoto Encyclopedia of Genes and Genomes (KEGG) was performed using the clusterProfiler v.4.4.4 [34], enrichplot v.1.18.3 [35], and enrichR v.3.1 [36] packages of R v.4.2.1 [32]. Over-representation analysis of BP and KEGG was performed using a hypergeometric test, considering an alpha level of significance of 5% corrected by FDR for GO terms tests. The abundance of genes related to a GO term or KEGG pathway present in the DEG list was compared to that of non-DEGs.

## 3. Results

### 3.1. Pre- and Post-Early Weaning Performance

No significant differences (*p* < 0.05) in BWi, WW, or ADG estimates were observed between the EW and CW groups (Table 1). A greater variation (SD) and range (minimum and maximum) of final body weight (BWf) was observed in the EW group compared to the CW group. In general, BWf or ADG was not affected by the stress associated with early weaning. However, environmental and intrinsic factors may have caused greater than expected heterogeneity in the performance of EW calves.

### 3.2. RNA Extraction and Sequencing

The mean total RNA concentration obtained for the 32 samples was 260.27 ng/µL and the mean RIN was 7.60 (range: 7.02 to 8.64). Among the 750 million reads obtained by sequencing (~23.60 million reads/sample), 94.3% passed the quality filters, providing about 710 million reads for alignment (~22.60 million reads/sample); of these, 94.31% had quality ≥ Q30 (~2.1 Gb/sample) (Table S1).

Sequencing of mRNA resulted in 321.56 million PE reads (2 × 100 bp) (~10.05 million PE reads/sample); of these, 316.5 million were uniquely mapped PE reads (Table S2). On average, 9.9 million uniquely mapped PE reads per sample were obtained, corresponding to 88.81% of all PE reads generated. Among all uniquely mapped PE reads, about 70.35% were assigned to exons (~7.9 million PE reads/sample) (Table S2) and were used for the subsequent descriptive and inferential analyses.

### 3.3. Count Matrix and Quality

Among 27,607 bovine genes, 16,034 were mapped to at least one PE read in one sample. After the deletion of features with low counts of PE reads already assigned using the edgeR package algorithm, 14,073 genes remained with sufficient counts within the groups, which were submitted to the analysis of differential gene expression. Figure S3 shows the size of the mapped libraries and the boxplots of the normalized read counts [log2(CPM)], with the consistent quartile distribution of samples between groups indicating good quality of the sequencing data.

A PCA analysis using measurements of the expression of each gene, reported as standardized log2CPM (Figure S2), showed that the similarity of individuals within groups was not much greater than between the different groups, indicating that the overall gene expression profile was different between the two time points and similar between individuals at each time point; however, the same is not observed when the treatments within time points are compared, particularly for the contrast of EW vs. CW at T1, which would be expected considering that the EW group had not yet been weaned. Although the division between treatments was more consistent at T2 than at T1, a better separation between individuals of the EW and CW groups at T2 would be expected. This was not observed, probably because they share expression profiles of many more similar growth-related genes than those expected to be altered by the applied treatments. Together, the first two components captured approximately 20% of the total variation observed between expression levels in the total sample (Figure S2). Figure S3 shows the results of cluster analysis illustrated as a heatmap, in which individuals (*n*) are grouped based on shared expression levels of the 100 most DEGs (*m*). These genes, in turn, are grouped according to the expression profile shared between samples. In this case, it is possible to observe the expression level of each gene in each sample and at each time point. The results show that, although the samples were not grouped in a more defined manner based on their treatments, there is a clear division between the EW and CW groups when we consider the genes most likely to be affected by the treatments.

### 3.4. Differential Gene Expression

**Figure 2:**
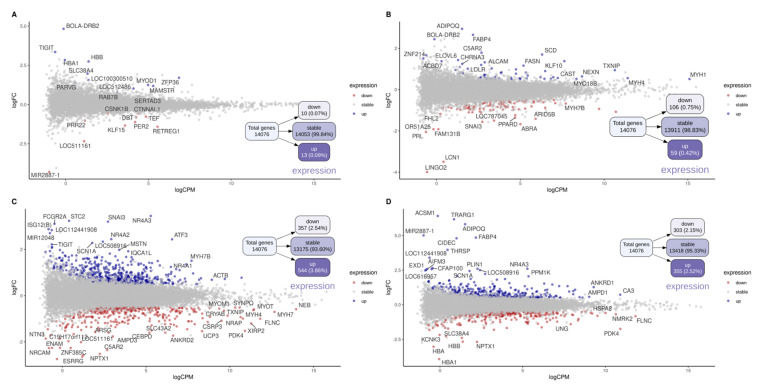
MD plot of different contrasts between the early weaning (EW), conventional weaning (CW), time 1 (T1), and time 2 (T2) groups. MDplot containing general expression normalised (logCPM) (x-axis), Fold Change (logCPM) (y-axis) and significative up- and down regulated differentially expressed genes(blue or red dots, respectivelly). The total number and percentage of genes and DEGs prospected are represented into boxes in treeplot for each contrasts: early weaning (EW) vs. conventional weaning (CW) at time 1 (T1) (**A**) and time 2 (T2) (**B**), and T1 vs. T2 within EW (**C**), and CW (**D**).

Analysis of differential gene expression identified respectively 23, 165, 901, and 658 DEGs for the following contrasts: EW vs. CW at T1, EW vs. CW at T2, T1 vs. T2 in CW, and T1 vs. T2 in EW (Figure 2; Table S3). As expected, there were few prospected DEGs within T1, in contrast to the other comparisons, including that between groups in T2. Within this context, in addition to the larger number of DEGs prospected at T2 (EW vs. CW), there are also genes that play key roles in energy metabolism, such as those involved in carbohydrate and lipid metabolism, and even some that may be important for the differentiation of muscle fibers. These genes include *FABP4*, *SCD1*, *FASN*, *LDLR*, *ADIPOQ*, *ACACA*, *PPARD*, and *ACOX3*, which are classically related to energy metabolism and are modulated by lipogenic and adipogenic activity.

When differences over time (T1 vs. T2) were analyzed within each treatment (EW or CW), we found a large number of DEGs compared to those identified based on the contrasts within T1 and T2 (Figure 2). Although both treatments returned abundant findings, in addition to the DEGs commonly modulated during early mammalian development, there was broad modulation of gene expression in the EW group, probably due to the intake of the concentrated diet. Many of these genes were also observed in the contrast of EW vs. CW at T2. Logically, these DEGs were not found in CW calves between T1 and T2, which consumed the same habitual diet that may have led to the modulation of genes commonly involved in growth and genes involved in environmental responses and in the gradual change to a predominantly grazing diet.

### 3.5. Functional Enrichment Analysis of Differentially Expressed Genes

**Figure 3:**
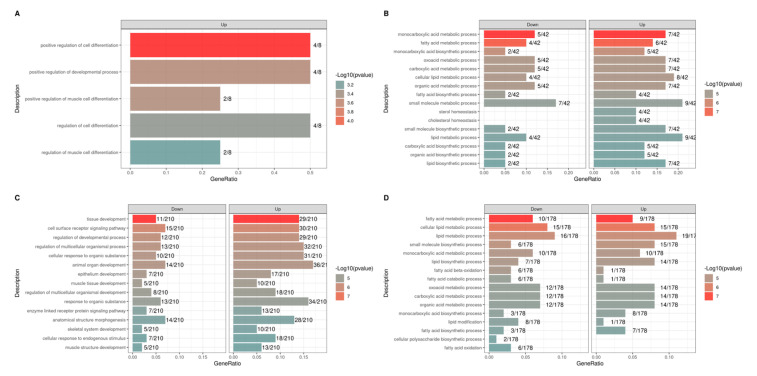
Biological process terms (BP) enriched from combined up- and downregulated DEGs in each contrast test: early weaning (EW) vs. conventional weaning (CW) at time 1 (T1) (**A**) and time 2 (T2) (**B**), and T1 vs. T2 within EW (**C**), and CW (**D**).

**Figure 4:**
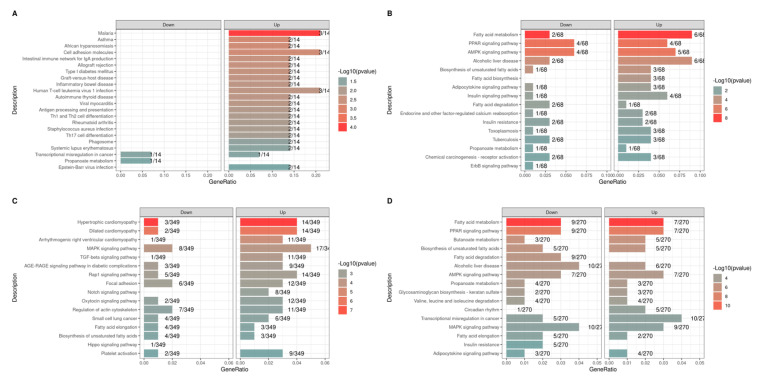
KEGG pathways enriched from combined up- and downregulated DEGs in each contrast test: early weaning (EW) vs. conventional weaning (CW) at time 1 (T1) (**A**) and time 2 (T2) (**B**), and T1 vs. T2 within EW (**C**) and CW (**D**).

The list of DEGs obtained for each contrast was used for functional analysis (Figure 2). Considering the contrasts of interest (EW vs. CW at T2 and T1 vs. T2 in EW), broad activity of BP (Figure 3) and metabolic pathways (Figure 4) related to metabolism, signaling, and metabolic processes of fatty acids and lipids was observed. Among BP terms, we observed an abundance of metabolic and biosynthetic processes of lipids, fatty acids, organic acids, and mono- and carboxylic acids. In EW vs. CW at T2, sterol and cholesterol homeostasis terms were exclusively enriched based only on genes downregulated in EW (Figure 2B). Similarly, in the contrast of T1 vs. T2 in EW, the proportion of downregulated genes was considerably higher for fatty acid polysaccharides bioprocesses, fatty acid oxidation, fatty-acid beta-oxidation, and fatty acid catabolic process terms (Figure 3D).

**Figure 5:**
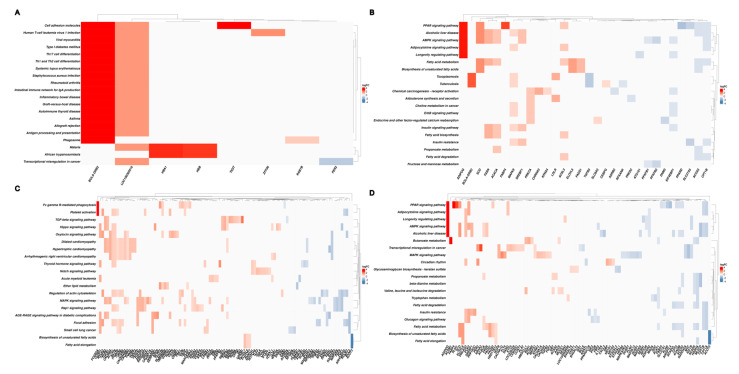
Clustergram of enriched KEGG pathways based on log2 (fold change) of up- and downregulated DEGs of different contrasts: early weaning (EW) vs. conventional weaning (CW) at time 1 (T1) (**A**) and time 2 (T2) (**B**), and T1 vs. T2 within EW (**C**) and CW (**D**).

**Figure 6:**
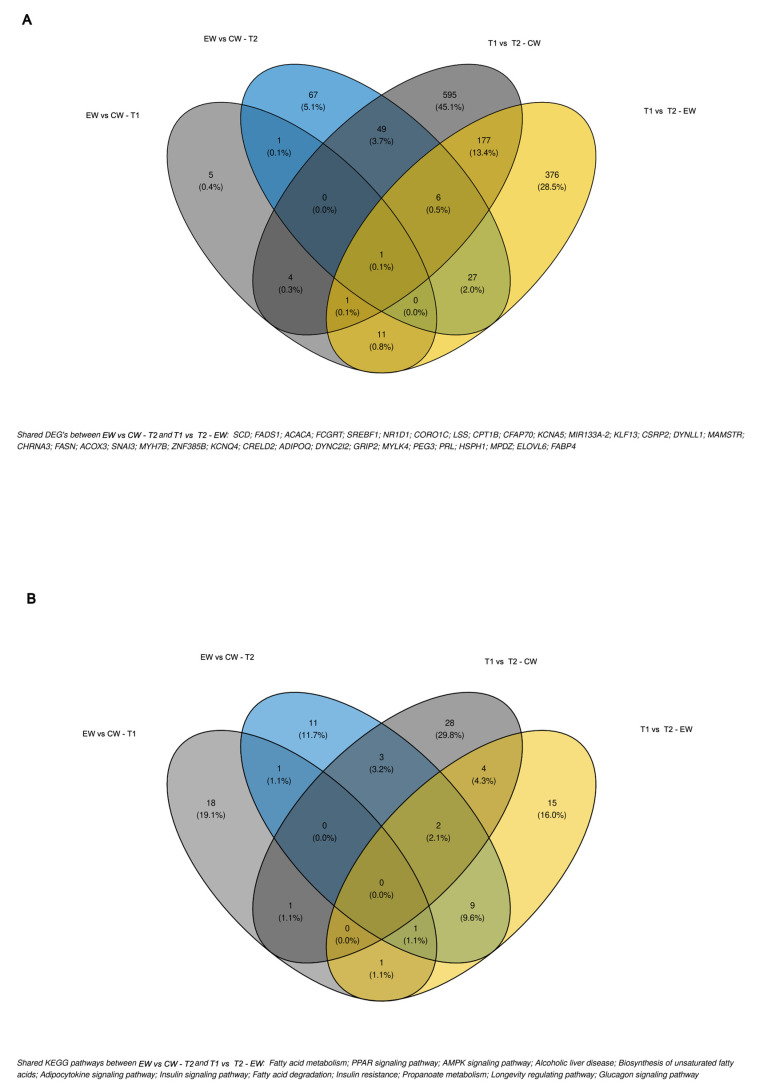
Venn diagram of shared DEGs (**A**) and KEGG pathways (**B**) of all contrast tests and description of DEGs and KEGG pathways shared by EW vs. CW at T2 and T1 vs. T2 in EW. Gray colors are related to contrasts in which none of the groups were affected by the treatment. The blue color indicates contrast between EW and CW treatments at time point 2 and the yellow color indicates contrast comparing EW at time point 1 and 2.

Among the enriched metabolic pathways, we highlight fatty acid metabolism, PPAR, AMPK, adipocytokine signaling pathways, fatty acid biosynthesis, and fatty acid degradation (Figure 4B,D). The last was observed exclusively in the contrast of T1 vs. T2 in EW, with only downregulated genes after EW (2). The downregulated genes include *ACOT3*, *ACOX3*, *PPARD*, *CPT1A*, *CEPT1B*, *CPT2*, *MLYCD*, and *FABP3* (Figure 5B,D; Tables S4 and S5), which are closely linked to the utilization of fat stores as energy and to antagonistic activities to lipogenesis. On the other hand, genes that play a key role in adipogenic and lipogenic metabolism were upregulated in pathways that participate in adipocyte differentiation and maturation, as well as in lipogenic activities, in addition to possible changes in myocyte energy profiles. We highlight the *FABP4*, *SCD1*, *FASN*, *PLIN1*, *LDLR*, and *ADIPOQ* genes shared between EW vs. CW at T2 and T1 vs. T2 in EW (Figure 5B,D and Figure 6; Table S6).

## 4. Discussion

Early weaning accompanied by a high concentrate diet or supplementation shows contradictory responses regarding the performance of calves during the weaning period [37,38,39]. However, EW is associated with higher carcass weight, with a reduction in or lack of effect on kidney, pelvic, heart fat (KPH), and with an increase in the marbling score of the carcass [19,24,37,38,40]. Our results showed the absence of an effect of EW even when combined with supplementation on ADG compared to CW calves (205 days, no supplementation). However, the results indicate that EW, followed by supplementation, induces the maximum fat deposition potential, mainly as marbling or intramuscular fat (IMF) in breeds with a low potential for IMF deposition, as is the case of Nellore cattle.

Nellore animals are recognized for their low IMF content and tougher meat, factors that reduce their acceptance by consumers [41,42]. However, higher expression of adipogenic and lipogenic genes such as *FABP4*, *SCD1*, *FASN*, *LDLR*, *ADIPOQ*, *ACACA*, *PPARD*, and *ACOX3* was observed in the EW group. These genes are related to the enrichment of lipid and fatty acid biosynthetic pathways, while downregulated genes are associated with lipid oxidation pathways.

The deposition of IMF is a multifactorial physiological process that depends on factors related to genetics, management, and animal nutrition. The mechanisms that control adipogenesis in the fetus and postnatally in skeletal muscle in vivo remain poorly defined [43].

Although little is known about IMF deposition in ruminants, studies show that the development of marbling occurs by an increase in the number (hyperplasia) and volume of adipocytes (hypertrophy) or a combination of both processes [44], indicating that the window of opportunity for increasing intramuscular adipocytes in cattle is small. The fact that the development of marbling starts during the prenatal phase (end of gestation) and extends up to 250 postnatal days, which is related to pre-adipocyte hyperplasia [45], renders the increase in marbling in meat even more complex. Within this context, EW can be used as a strategy to accelerate the differentiation and maturation of intramuscular adipocytes. This was observed in the EW group at T2 in which the *ADIPOQ* and *THRSP* gene upregulated. The *ADIPOQ* gene is secreted by mature adipocytes and promotes adipocyte differentiation [46], and when upregulated, and associated with increased lipid deposition in mature adipocytes [47]. Meanwhile, higher expression of the THRSP gene is associated with higher marbling content in breeds such as the wagyu, which are recognized for their high marbling ability in the meat [48].

Among the metabolic pathways enriched in the EW group, the fatty acid metabolism, PPAR, AMPK, and adipocytokine signaling pathways can be highlighted. The PPAR family is associated with the regulation of adipocyte differentiation [49], with *PPARG* being one of the most studied members of this family. Its ability to induce adipocyte differentiation has been extensively investigated [50]. As suggested by [47] who evaluated EW in Angus and Angus x Simmental cattle, *PPARG* can upregulate the expression of activators of fatty acid synthesis (*THRPS*, *SREBP1*, and *INSIG1*) and fat synthesis-related enzymes (*FASN*, *SCD*, *ELOVL6*, *PCK1*, and *DGAT2*), which is consistent with our results. We also observed the upregulation of genes such as *THRPS*, *FASN*, *SCD,* and *ELOVL6* when compared to the CW group at T2 and in contrast to T2 vs. T1 in EW. These findings indicate high recruitment of adipocytes within the “marbling window” which, according to [51], occurs up to 250 days after birth. Within this context, EW acts not only by increasing the number of adipocytes in IMF but also by the deposition of fat in adipocytes of intramuscular adipose tissue, which are known to have a low rate of fatty acid synthesis and deposition [52,53]. The greater expression of the *PLIN1* gene for the EW group in addition to acting in the regulation of triglyceride synthesis, also favored the formation of large lipid droplets, by inhibiting the hydrolysis of triglyceride in adipocytes [54]. This can also be favored by the action of the PLIN1 gene, which is expressed in adipocytes. In addition to regulating the synthesis of triglycerides, this gene also participates in the formation of large lipid droplets by inhibiting the hydrolysis of triglycerides in adipocytes [54].

The decrease in the biological pathways involved in the degradation of fatty acids in response to EW cannot be solely related to the downregulation of *PPARD*. The decrease in the biological pathways involved in fatty acid degradation in response to EW might be related not only to the downregulation of *PPARD*. According to [50], in cattle, higher expression of *PPARD* acts on the stimulation of fatty acid oxidation as a mechanism to save glucose in animals receiving low-starch diets. Supplementation in EW acts as a positive signal of the animal’s nutritional status, reducing fluctuations in the expression of the *THRSP* gene throughout the growth period. According to [55], the expression of this gene oscillates during the development of the animal and is sensitive to environmental and nutritional signals. The increase in *THRSP* gene expression for periods greater than 280 days of life was observed in animals with a high potential for marbling deposition [55].

In addition to the reduction in lipid oxidation in the EW group, it is possible that early grain intake by animals of this group reduces the competition for nutrients destined for fat and muscle deposition pathways in the carcass. During the growth phase, nutrients are partitioned in favor of bone and muscle growth, while the fat deposition rate is relatively low [56]. We observed upregulation of *MYOD1* in the EW group at T1 (up to 120 days). This gene acts together with *MyF5* as the first myogenic regulatory factor and is necessary for the determination of the myogenic lineage [57]. However, in mature muscle tissue, it has been suggested that the upregulation of *Myf5* [58,59] and *MYOD1* [60] is related to the proliferation and differentiation of satellite cells for hypertrophy, respectively.

In the control group, upregulation of the myostatin gene (*MSTN*) was observed for the T2 vs. T1 contrast, which may indicate muscle growth restriction in these animals. The *MSTN* gene is mainly expressed in muscle, where it acts as a negative regulator of skeletal muscle growth and development [61]. Its detection in adipose tissue is associated with the regulation of the development of this tissue [62]. In addition to inhibiting adipogenesis in pre-adipocytes [63], the high expression of *MSTN* and *PPARG* reduces IMF content and the size of adipocytes.

Our results help to understand the mechanism associated with the response to EW according to [24,37,38] EW has the potential to stimulate the deposition of IMF, with a minimal increase in subcutaneous fat or even KPH, which is recognized as a “carcass contamination fat” and is accompanied by an increase in carcass weight. The positive effect of EW on carcass weight and IMF content can be associated with the positive effect of EW on energy metabolism in the growth phase, which was accompanied by the stimulation of adipogenic and lipogenic pathways, without affecting the metabolic pathways associated with muscle growth. Our results also indicate the possibility of reducing saturated fatty acids in the meat of EW animals. Within this context, genes associated with the *de novo* biosynthesis and unsaturation of fatty acids such as *SCD*, *FASN*, *ELOVL6*, *ACACA*, and *FABP4* were upregulated in animals of the EW group. Furthermore, upregulated *FABP4*, *G6PD*, *FASN*, and *ACACA* act on the terminal differentiation of adipocytes [64,65]. *FASN* and *ACACA* together perform de novo synthesis of fatty acids [43], such as palmitate from acetyl-CoA and malonyl-CoA [66]. The palmitate (C16) is used as a substrate for elongase, an enzyme encoded by the *ELOVL6* gene that catalyzes the elongation of saturated and monounsaturated fatty acids with 12, 14, and 16 carbons, in which C18 fatty acids are the final product [46]. An increase in the energy and/or starch content of cattle diets is associated with higher expression of the *SCD* gene [67] that encodes ∆9 desaturase, an enzyme that converts saturated fatty acids into cis-9 monounsaturated fatty acids [52]. When upregulated, all of these genes are associated with greater IMF deposition in cattle, as observed for the *SCD* gene [52,68], which was also found to stimulate the expression of *FASN*, *ACACA*, and *FABP4* [69]. Higher expression of *FABP4* is not only associated with an increase in IMF content but also in triacylglycerol [70], and stimulates adipocyte differentiation [71].

Early weaning has the potential to alter the expression of genes that are important for increasing the IMF content of Nellore meat. This breed has been recognized for having high lipid turnover, as observed by [66] who compared Nellore and Angus animals receiving the same finishing diet. Within this context, EW favors a reduction in the finishing period of animals with low-fat deposition capacity by stimulating adipogenic and lipogenic pathways and increasing fat deposition, which may improve meat qualitative parameters such as tenderness and juiciness.

## 5. Conclusions

Early weaning associated with supplementation did not affect weight gain compared to traditionally weaned calves. However, early weaning, associated with an adequate nutritional strategy, positively affects regulating pathways related to energy metabolism, adipogenesis, and lipogenesis in skeletal muscle tissue increases PPAR, AMPK, and the adiponectin signaling pathway and biosynthesis of mono- and polyunsaturated fat acids. Therefore, this management and nutritional strategy can potentially increase intramuscular fat in Nellore beef.

## Figures and Tables

**Table 1:** Live weight at 120 (time 1) and 205 (time 2) days of age and average daily weight gain (ADG) of early (EW) and conventionally weaned (CW) Nellore calves.

Categories	Group	Days	n	Min.	Max.	Median	Mean ^1^	SD ^2^
Live weight (kg)	CW	120	20	103	155	122	122.50 ± 2.961 ^b^	13.241
	EW	120	20	96	141	125	123.15 ± 2.606 ^b^	11.654
	CW	205	20	169	216	198	194.39 ± 3.023 ^a^	12.825
	EW	205	20	156	221	191	190.32 ± 4.375 ^a^	19.070
ADG (kg)	CW	205–120	20	0.553	1.047	0.853	0.845 ± 0.028 ^a^	0.127
	EW	205–120	20	0.588	1.024	0.788	0.786 ± 0.030 ^a^	0.133

^1^ Mean = mean ± standard error; ^2^ SD = standard deviation. Different letters indicate significant differences (*p*-value > 0.05) between means by Tukey’s multiple test and t for the Live Weight and ADG categories, respectively.

## Data Availability

The data presented in this study are available on request from the corresponding author. The data are not publicly available due to privacy and patent intent.

## Supplementary Materials

The following supporting information can be downloaded at: https://www.mdpi.com/article/10.3390/metabo13091028/s1, Supplementary Material S1: Alignment Workflow scheme and descriptive statistics and supplementary DEG results; Supplementary Material S2: Diferentially expressed genes; Supplementary Material S3: Enriched ontology genes terms to biological process; Supplementary Material S4: Enriched KEGG pathways; Supplementary Material S5: Differentially expressed genes shared between contrasts.
