# Supplementary material for: Early Weaning Possibly Increases the Activity of Lipogenic and Adipogenic Pathways in Intramuscular Adipose Tissue of Nellore Calves
Source: Metabolites. 2023 Sep 21;13(9):1028. doi: 10.3390/metabo13091028 (PMC10536964; doi:10.3390/metabo13091028)
Supplement: Supplementary file 1 [file metabolites-13-01028-s001.zip › Supplementary 1 - misc.pdf]

Supplementary Material:

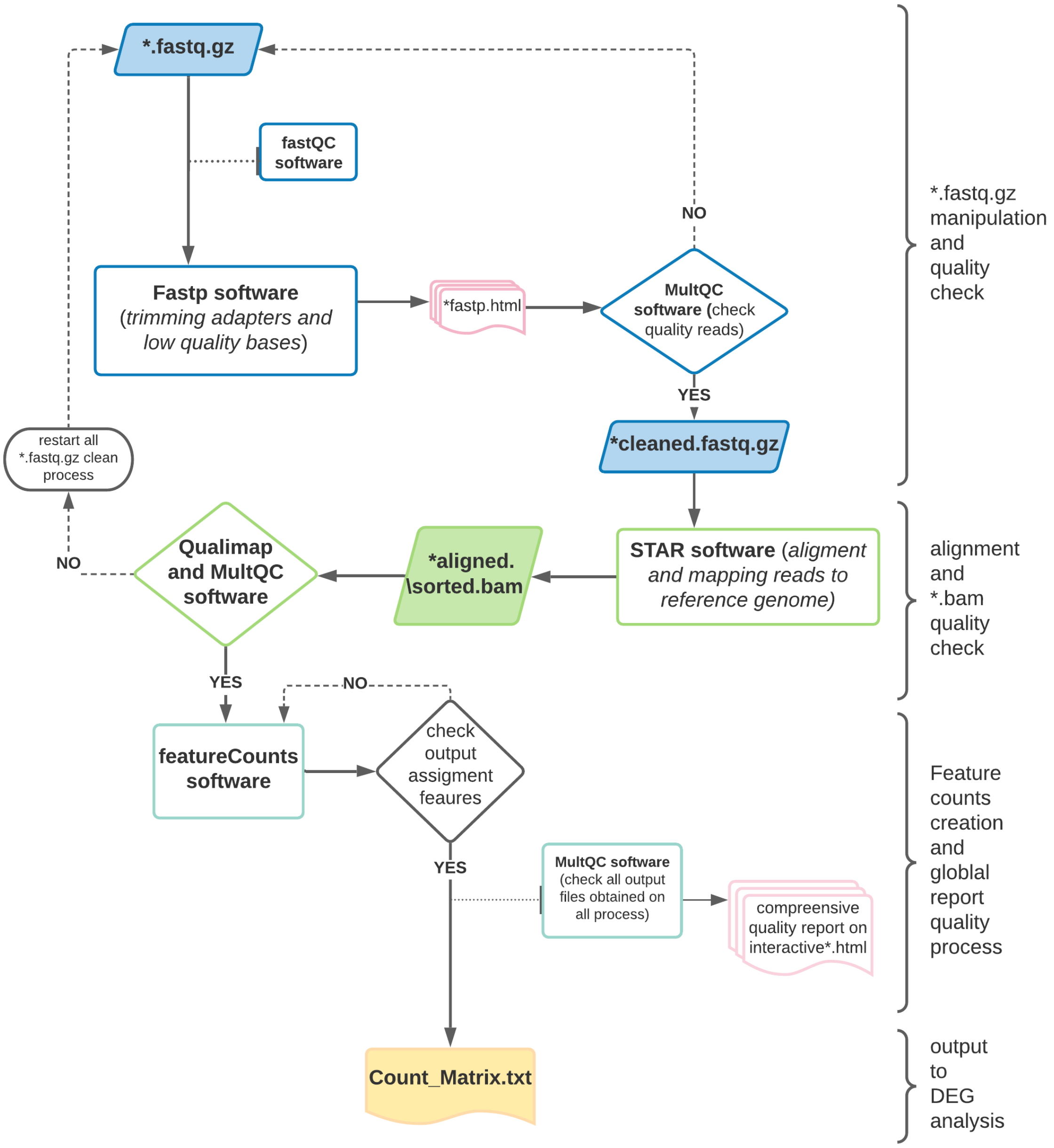

Figura S1 - Alignment and quality control at obtained raw count matrix fluxogram

Table S1 – Quality scores of reads after fastp QC control.

Sample Name	Total Mb reads	Passed Filter (Mb)	Low Quality (Mb)	Too Many N (Mb)	% > Q30	Mb Q30 bases	GC content	% PF	% Adapter
S10	24,55	23,25	1,30	1,82E-04	94,40%	2.185,30	52,20%	94,70%	5,00%
S11	22,36	21,26	1,10	4,48E-04	94,50%	1.993,60	52,20%	95,10%	6,10%
S12	24,26	23,15	1,10	3,16E-04	94,50%	2.175,70	51,10%	95,40%	5,80%
S13	23,64	22,44	1,20	3,20E-04	94,50%	2.109,20	52,40%	94,90%	5,30%
S14	19,14	17,98	1,15	2,42E-04	94,30%	1.687,60	52,60%	94,00%	4,90%
S15	24,44	23,01	1,43	3,56E-04	94,20%	2.156,40	52,10%	94,20%	5,30%
S16	22,93	21,64	1,29	1,96E-04	94,50%	2.034,40	54,40%	94,40%	5,30%
S17	21,41	20,12	1,29	5,16E-04	94,20%	1.885,60	52,30%	94,00%	5,20%
S18	24,44	22,80	1,64	1,68E-04	94,10%	2.137,00	52,70%	93,30%	4,80%
S19	25,64	24,19	1,45	3,60E-04	94,40%	2.268,80	52,80%	94,30%	5,70%
S1	22,45	21,09	1,36	3,44E-04	94,30%	1.970,00	52,50%	94,00%	6,90%
S20	25,12	23,72	1,39	1,86E-04	94,40%	2.228,80	53,70%	94,40%	5,10%
S21	22,96	21,59	1,36	4,04E-04	94,30%	2.017,70	52,10%	94,10%	6,50%
S22	23,00	21,63	1,37	3,20E-04	94,20%	2.029,30	52,00%	94,00%	4,90%
S23	23,24	21,95	1,29	3,28E-04	94,30%	2.055,00	51,90%	94,50%	6,10%
S24	24,44	22,90	1,53	3,08E-04	94,20%	2.146,90	52,10%	93,70%	5,10%
S25	25,77	24,38	1,39	4,18E-04	94,30%	2.284,60	51,60%	94,60%	5,90%
S26	27,93	26,48	1,45	4,68E-04	94,40%	2.485,50	52,40%	94,80%	5,20%
S27	23,34	21,95	1,39	4,24E-04	94,30%	2.052,30	51,90%	94,10%	6,40%
S28	26,34	24,86	1,48	4,24E-04	94,30%	2.329,30	51,50%	94,40%	5,90%
S29	22,83	21,36	1,47	3,40E-04	94,10%	2.002,10	52,20%	93,60%	4,70%
S2	23,87	22,82	1,05	3,58E-04	94,60%	2.142,80	52,20%	95,60%	6,20%
S30	24,35	23,03	1,31	4,14E-04	94,30%	2.161,40	51,70%	94,60%	4,90%
S31	25,04	23,62	1,42	4,58E-04	94,30%	2.210,30	52,00%	94,30%	6,30%
S32	25,53	24,05	1,47	4,06E-04	94,30%	2.246,00	52,40%	94,20%	7,00%
S3	22,52	21,11	1,41	2,84E-04	94,20%	1.974,80	52,30%	93,70%	5,80%
S4	21,57	20,28	1,29	2,54E-04	94,20%	1.895,80	52,80%	94,00%	6,40%
S5	20,58	19,21	1,37	2,88E-04	94,10%	1.793,10	52,40%	93,30%	6,30%
S6	22,11	20,76	1,35	2,46E-04	94,20%	1.943,90	52,90%	93,90%	5,70%
S7	23,86	22,68	1,18	3,04E-04	94,50%	2.127,20	51,70%	95,10%	6,10%
S8	23,20	22,04	1,17	2,20E-04	94,50%	2.073,00	52,10%	95,00%	4,80%
S9	22,21	20,76	1,45	4,24E-04	94,10%	1.932,40	52,40%	93,50%	7,20%
Total	755,05	712,14	42,90	0,01		66735,80			
Mean	23,60	22,25	1,34	3,35E-04	94,31%	2085,494	52,30%	94,30%	5,71%
SD	1,76	1,71	0,13	9,22E-05	0,14%	161,8689	0,61%	0,58%	0,70%
Min.	19,14	17,98	1,05	1,68E-04	94,10%	1687,60	51,10%	93,30%	4,70%
Max.	27,93	26,48	1,64	5,16E-04	94,60%	2485,50	54,40%	95,60%	7,20%

Table S2 – Metrics of alignments and assigned exons.

Sample Name	Total Mapped	Total Unmapped	% Uniquely Aligned	M Uniquely Aligned[1]	% Uniquely Assigned	M Uniquely Assigned[2]
GLP-10_S10	10,53	1,09	89,20%	10,4	69,30%	8,2
GLP-11_S11	9,73	0,90	90,00%	9,6	70,60%	7,65
GLP-12_S12	10,65	0,92	90,60%	10,5	69,80%	8,25
GLP-13_S13	10,27	0,94	90,10%	10,1	71,70%	8,2
GLP-14_S14	8,07	0,92	88,30%	7,9	70,50%	6,45
GLP-15_S15	10,35	1,15	88,60%	10,2	69,50%	8,15
GLP-16_S16	9,87	0,95	89,40%	9,7	75,90%	8,4
GLP-17_S17	9,01	1,05	88,10%	8,9	68,10%	7
GLP-18_S18	10,13	1,27	87,50%	10	67,10%	7,8
GLP-19_S19	10,89	1,20	88,70%	10,7	68,60%	8,45
GLP-1_S1	9,51	1,04	88,80%	9,4	70,30%	7,55
GLP-20_S20	10,73	1,13	88,90%	10,5	70,70%	8,55
GLP-21_S21	9,74	1,05	88,60%	9,6	66,90%	7,4
GLP-22_S22	9,72	1,10	88,50%	9,6	69,30%	7,65
GLP-23_S23	9,93	1,04	88,90%	9,8	68,20%	7,65
GLP-24_S24	10,26	1,19	88,20%	10,1	67,80%	7,9
GLP-25_S25	11,06	1,13	89,30%	10,9	69,00%	8,55
GLP-26_S26	12,04	1,19	89,30%	11,8	66,70%	9
GLP-27_S27	9,80	1,17	87,90%	9,6	67,20%	7,5
GLP-28_S28	11,17	1,26	88,40%	11	67,10%	8,5
GLP-29_S29	9,48	1,20	87,30%	9,3	66,10%	7,2
GLP-2_S2	10,62	0,79	91,60%	10,5	73,10%	8,5
GLP-30_S30	10,45	1,06	89,20%	10,3	68,00%	8
GLP-31_S31	10,59	1,22	88,20%	10,4	65,70%	7,9
GLP-32_S32	10,84	1,19	88,30%	10,6	71,70%	8,85
GLP-3_S3	9,46	1,09	88,20%	9,3	69,80%	7,5
GLP-4_S4	9,10	1,03	88,40%	9	68,10%	7,05
GLP-5_S5	8,57	1,03	87,90%	8,4	69,60%	6,8
GLP-6_S6	9,33	1,05	88,50%	9,2	70,70%	7,45
GLP-7_S7	10,35	0,99	89,90%	10,2	70,80%	8,2
GLP-8_S8	10,05	0,97	89,70%	9,9	72,90%	8,2
GLP-9_S9	9,22	1,16	87,30%	9,1	68,00%	7,2
Total	321,56	34,51		316,5		251,65
Mean	10,05	1,08	88,81%	9,9	70,34%	7,86
SD	0,81	0,11	0,95%	0,79	2,23%	0,61
Min.	**8,07**	**0,79**	**87,30%**	**7,90**	**65,70%**	**6,45**
Max .	12,04	1,27	91,60%	11,80	75,90%	9,00

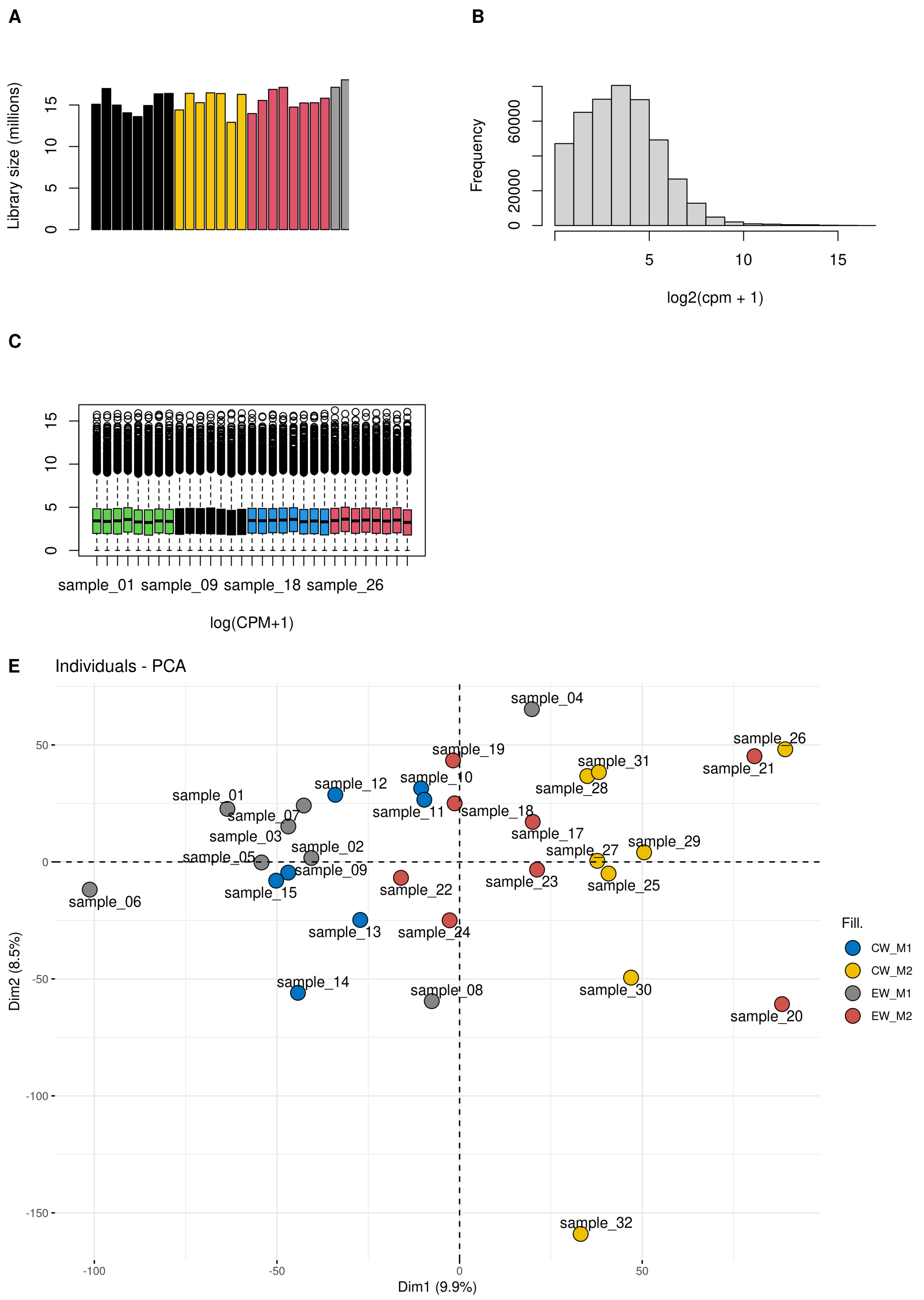

Figure S2 – Size Library (A), distribution of all normalized scaled counts (B), boxplot of normalized scaled counts per sample (C) and PCA plot (E).

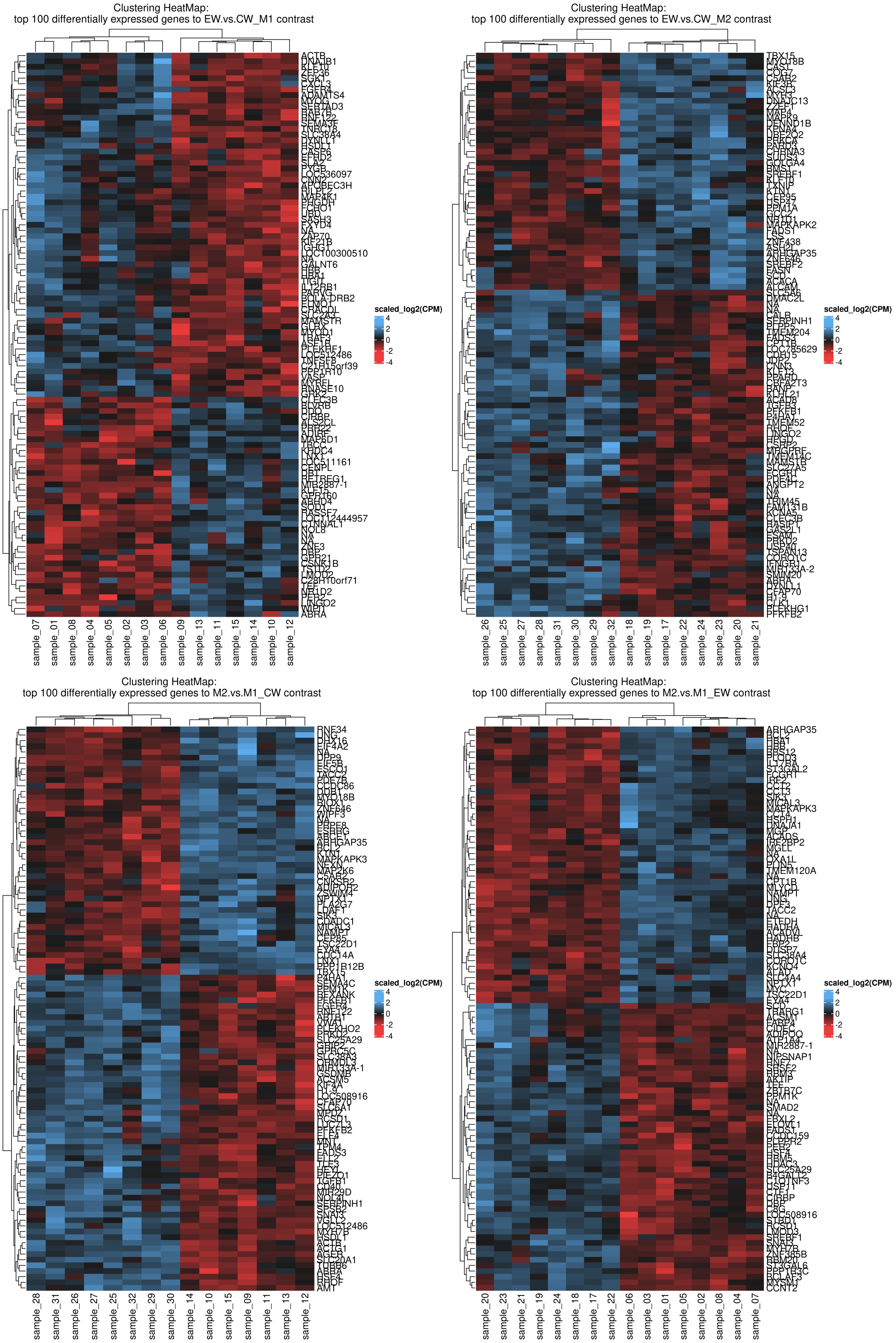

Figura S3. Clustergram of scaled log2(CPM) from 100 more expressed genes to each one contrast.

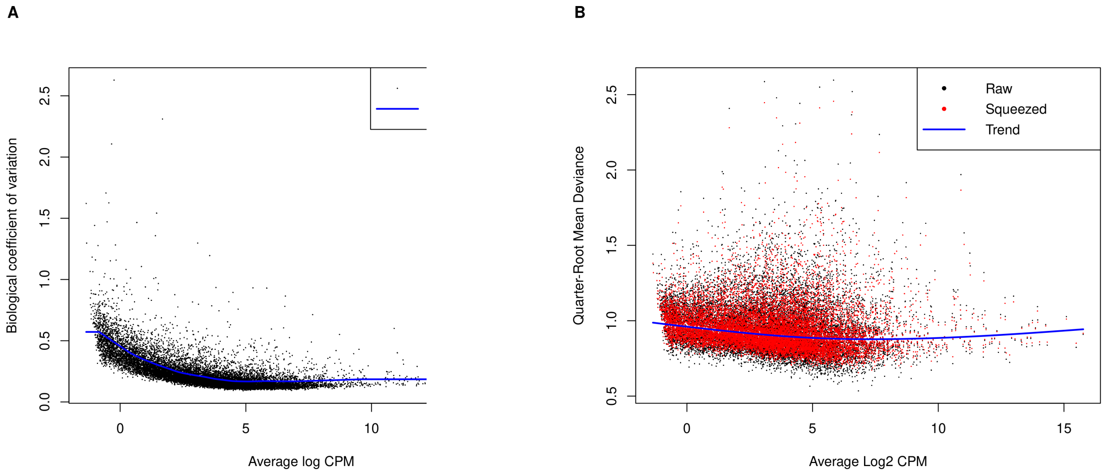

Figure S4 – Biological variation and counts deviation.

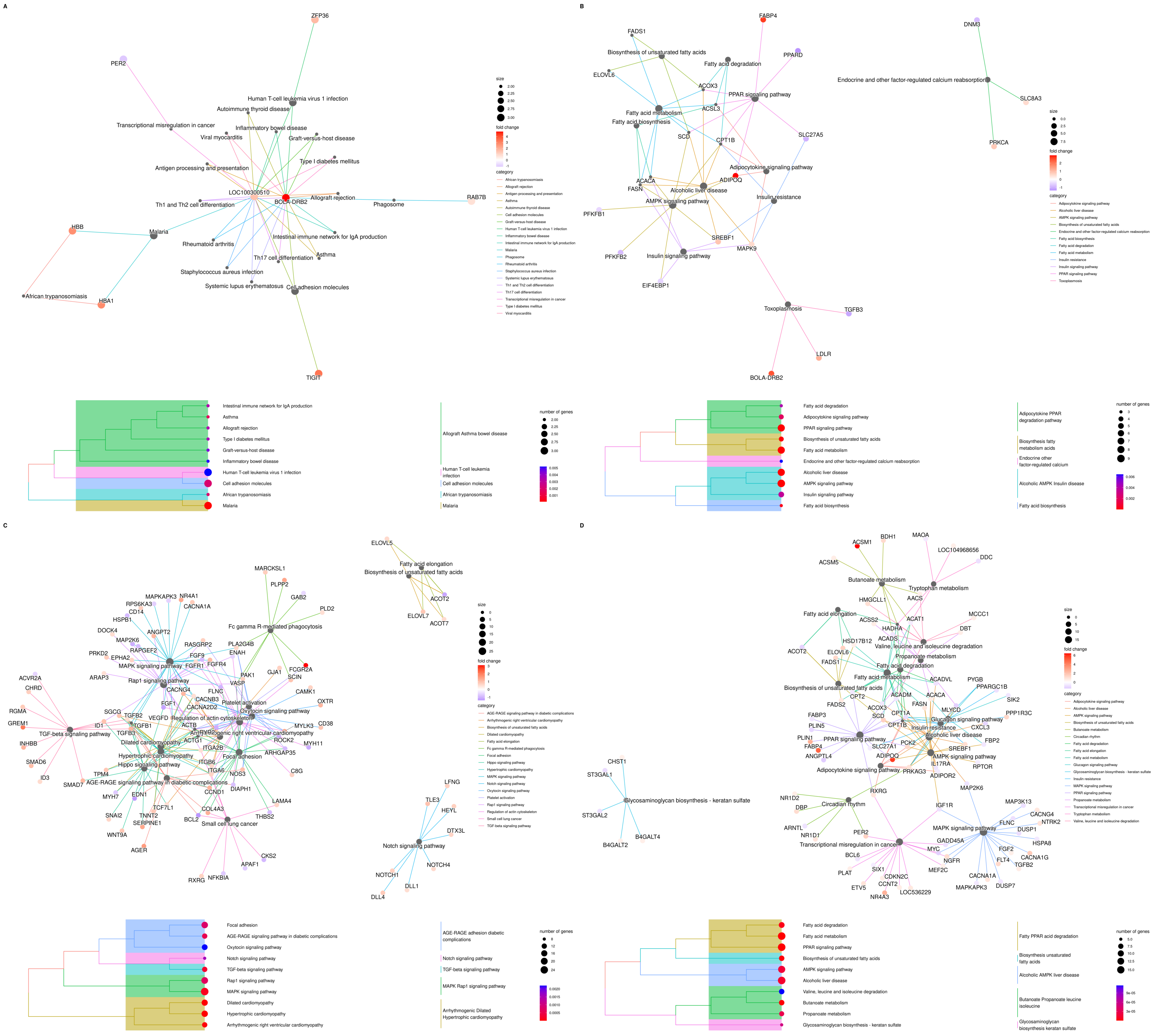

Figure S5 - Network DEGs interfaced by enriched KEGG Pathways
