# Supplementary material for: Early Weaning Possibly Increases the Activity of Lipogenic and Adipogenic Pathways in Intramuscular Adipose Tissue of Nellore Calves
Source: Metabolites. 2023 Sep 21;13(9):1028. doi: 10.3390/metabo13091028 (PMC10536964; doi:10.3390/metabo13091028)
Supplement: Supplementary file 1 [file metabolites-13-01028-s001.zip › Supplementary 2 - DEG list.pdf]

1. Prospected DEGs to EW vs CW into Time 1

ENSEMBL	SYMBOL	ENTREZID	GENETYPE	logFC	logCPM	F	PValue	FDR
ENSBTAG00000014197	*SLC38A4*	527476	protein-coding	1.96760862	1.31931627	47.1604889	6.78E-08	0.00095419
ENSBTAG00000008313	*KLF15*	407241	protein-coding	-1.3340748	3.58758725	32.5671916	2.11E-06	0.01063183
ENSBTAG00000038397	*BOLA-DRB2*	538700	protein-coding	4.82883661	-0.1292263	32.0992771	2.38E-06	0.01063183
ENSBTAG00000001879	*PER2*	512237	protein-coding	-1.1155324	4.19762175	29.6931973	4.55E-06	0.01063183
ENSBTAG00000011617	*MAMSTR*	505540	protein-coding	1.18628229	5.28737126	29.3653218	4.98E-06	0.01063183
ENSBTAG00000008573	*ZFP36*	282127	protein-coding	1.70977905	6.84759534	29.1909431	5.23E-06	0.01063183
ENSBTAG00000016580	*TEF*	616076	protein-coding	-0.7705148	4.84639851	29.0510743	5.44E-06	0.01063183
ENSBTAG00000006320	*DBT*	280759	protein-coding	-0.8022455	4.25959176	28.4311146	6.46E-06	0.01063183
ENSBTAG00000004997	*CTNNAL1*	515749	protein-coding	-0.5779569	4.38934478	28.2504124	6.80E-06	0.01063183
ENSBTAG00000037644	*HBB*	280813	protein-coding	2.747092	1.38130493	27.3140321	8.86E-06	0.01247563
ENSBTAG00000016444	*RETREG1*	540068	protein-coding	-1.4121939	5.55079677	26.639511	1.08E-05	0.01376688
ENSBTAG00000002216	*MYOD1*	281938	protein-coding	1.2456536	4.97894654	24.4335899	2.06E-05	0.02232196
ENSBTAG00000054844	*HBA1*	100140149	protein-coding	2.84118762	-0.0496511	22.16739	4.14E-05	0.04159066
ENSBTAG00000008564	*LOC511161*	511161	protein-coding	-2.3382612	1.11506481	21.3269542	5.40E-05	0.04410225
ENSBTAG00000010502	*SERTAD3*	767935	protein-coding	0.51812042	4.42123942	21.1571445	5.70E-05	0.04410225
ENSBTAG00000039794	*CSNK1B*	785423	protein-coding	-0.5565634	2.54151174	21.0588369	5.88E-05	0.04410225
ENSBTAG00000008057	*PARVG*	790892	protein-coding	1.38889939	0.56873845	20.9238249	6.14E-05	0.04410225
ENSBTAG00000012774	*RAB7B*	538050	protein-coding	0.76092977	3.11049423	20.8416548	6.31E-05	0.04410225
ENSBTAG00000011921	*TIGIT*	100140801	protein-coding	3.35154312	-0.6351425	20.3894445	7.30E-05	0.04410225
ENSBTAG00000025550	*PRR22*	616551	protein-coding	-1.0220893	1.16540463	20.3791173	7.33E-05	0.04410225
ENSBTAG00000001143	*LOC512486*	512486	protein-coding	1.02085294	4.09341919	20.192599	7.79E-05	0.04410225
ENSBTAG00000054661	*MIR2887-1*	100498812	ncRNA	-4.2944742	-0.992082	20.1740803	7.83E-05	0.04410225
ENSBTAG00000027204	*LOC100300510*	100300510	protein-coding	1.56590918	1.37476048	19.7408816	9.03E-05	0.0488852

2. Prospected DEGs to EW vs CW into Time 2

ENSEMBL	SYMBOL	ENTREZID	GENETYPE	logFC	logCPM	F	PValue	FDR
ENSBTAG00000018352	ABRA	539379	protein-coding	-1.67359	5.00194902	65.5984173	2.00E-09	1.55E-05
ENSBTAG00000032534	RHOF	617894	protein-coding	-0.969409	4.59044253	65.0272227	2.20E-09	1.55E-05
ENSBTAG00000023745	BANP	513446	protein-coding	-0.8385521	3.6340194	49.3524359	4.28E-08	0.00020076
ENSBTAG00000055207	SCD	280924	protein-coding	1.70030698	6.29933393	43.1166075	1.64E-07	0.00057722
ENSBTAG00000001027	SERPINH1	510850	protein-coding	-0.8794851	6.48306976	40.8459749	2.75E-07	0.00066496
ENSBTAG00000032996	P4HA1	518288	protein-coding	-0.5864958	4.79769172	40.7188043	2.83E-07	0.00066496
ENSBTAG00000000874	CAST	281039	protein-coding	0.56327682	7.236706	38.828792	4.42E-07	0.00088869
ENSBTAG00000010927	CBFA2T3	523815	protein-coding	-0.9551136	4.90245873	34.7191396	1.21E-06	0.00179167
ENSBTAG00000043567				-0.9343654	9.69991559	34.5259962	1.27E-06	0.00179167
ENSBTAG00000001748	TRIM45	539091	protein-coding	-0.8488774	4.23992508	33.6781043	1.58E-06	0.00202283
ENSBTAG00000043570				-1.0750833	10.6998651	33.2937532	1.75E-06	0.00204754
ENSBTAG00000022294	FADS1	533107	protein-coding	1.0265584	3.28921006	31.8723289	2.53E-06	0.00274163
ENSBTAG00000020060	TXNIP	506790	protein-coding	0.94341066	10.6402975	30.6160055	3.54E-06	0.00298461
ENSBTAG00000017567	ACACA	281590	protein-coding	0.8341368	3.87684792	30.6040313	3.55E-06	0.00298461
ENSBTAG00000014265	SREBF2	507102	protein-coding	0.68165355	5.43772898	30.5119957	3.64E-06	0.00298461
ENSBTAG00000016070	LINGO2	539968	protein-coding	-3.9863699	-0.5669052	30.0950116	4.08E-06	0.00298461
ENSBTAG00000013926	FCGRT	338062	protein-coding	-0.6564832	7.08418064	30.003316	4.18E-06	0.00298461
ENSBTAG00000016316	ZZEF1	508144	protein-coding	0.54433281	5.3755685	29.8475704	4.37E-06	0.00298461
ENSBTAG00000032223	COG7	504790	protein-coding	0.61891904	3.50092031	29.775482	4.45E-06	0.00298461
ENSBTAG00000000172	PFKFB1	282304	protein-coding	-0.5004347	5.37531359	29.238232	5.16E-06	0.00330274
ENSBTAG00000011034	ANGPT2	282141	protein-coding	-1.0985205	2.13647254	28.5407942	6.27E-06	0.00367525
ENSBTAG00000007884	SREBF1	539361	protein-coding	0.89745757	6.48331898	27.9996685	7.30E-06	0.00375312
ENSBTAG00000001061	PRKCA	282001	protein-coding	0.81372544	3.83184413	27.9956468	7.30E-06	0.00375312
ENSBTAG00000012178	NR1D1	768225	protein-coding	0.98181212	4.73723832	27.9832057	7.33E-06	0.00375312
ENSBTAG00000007993	CORO1C	515798	protein-coding	-0.5292733	4.32832386	27.9178915	7.47E-06	0.00375312
ENSBTAG00000017416	KPNA4	535090	protein-coding	0.52652786	6.43722542	27.6133128	8.14E-06	0.0039348
ENSBTAG00000018936	LSS	615906	protein-coding	0.71298561	2.83633693	27.5079976	8.39E-06	0.0039348
ENSBTAG00000017258	ACSL3	100138312	protein-coding	0.77229314	4.73843427	27.3733624	8.71E-06	0.00395681
ENSBTAG00000002126	PFKFB2	287019	protein-coding	-0.8526011	4.1299211	27.1955581	9.17E-06	0.00403296
ENSBTAG00000000698	MYO18B	540437	protein-coding	0.51680621	8.34917908	27.0269313	9.62E-06	0.00410441
ENSBTAG00000037735	C5AR2	522144	protein-coding	1.78825232	2.68082591	26.5490255	1.10E-05	0.0043053
ENSBTAG00000003215	KIF3B	508404	protein-coding	0.52733228	3.24870746	26.4711334	1.13E-05	0.0043053
ENSBTAG00000015511	FADS3	515925	protein-coding	-0.7770949	4.0465563	26.449456	1.14E-05	0.0043053
ENSBTAG00000017542	PPARD	353106	protein-coding	-1.3622515	4.68360399	26.443017	1.14E-05	0.0043053
ENSBTAG00000004709	MAPK9	534125	protein-coding	0.55277053	3.72541888	25.7649	1.39E-05	0.00476524
ENSBTAG00000043979	H1-9	615867	protein-coding	-0.9283654	2.80727203	25.3361101	1.57E-05	0.00527838
ENSBTAG00000016048	CPT1B	509459	protein-coding	-0.5633775	6.07723072	24.6492795	1.93E-05	0.00618163
ENSBTAG00000007767	TBX15	521974	protein-coding	0.52886668	6.82483376	24.4028952	2.08E-05	0.00641653
ENSBTAG00000007246	TMEM204	615464	protein-coding	-0.7263684	3.83722251	24.3771077	2.10E-05	0.00641653
ENSBTAG00000014396	KLF10	522795	protein-coding	1.37831211	7.62884858	23.6890782	2.58E-05	0.00742018
ENSBTAG00000009451	JDP2	531476	protein-coding	-0.7338472	2.58513589	23.5617115	2.69E-05	0.00746244

ENSBTAG00000015185	MRGPRF	615886	protein-coding	-0.6840597	2.31711123	23.5333249	2.71E-05	0.00746244
ENSBTAG00000012004	TGFB3	538957	protein-coding	-1.1036339	2.65678485	23.4760578	2.76E-05	0.00746244
ENSBTAG00000015202	DMAC2L	493709	protein-coding	-0.5326851	3.82847429	23.1847034	3.01E-05	0.0080069
ENSBTAG00000050814	CFAP70	529891	protein-coding	-0.8738044	1.92829515	23.0545112	3.14E-05	0.00809125
ENSBTAG00000007553	KCNA5	508960	protein-coding	-1.0601482	2.58344716	23.0306058	3.16E-05	0.00809125
ENSBTAG00000011530	CDH15	522202	protein-coding	-0.6134909	4.02924144	22.7918752	3.40E-05	0.00848371
ENSBTAG00000012504	GAS2L1	518935	protein-coding	-0.8158262	2.25565151	22.4849838	3.75E-05	0.00893143
ENSBTAG00000000088	ALCAM	281614	protein-coding	1.32245675	2.87227277	22.266171	4.01E-05	0.00918384
ENSBTAG00000009679	PLEKHG1	525772	protein-coding	-0.8605992	3.82674798	22.0769087	4.26E-05	0.00936247
ENSBTAG00000051572	ZNF646	783820	protein-coding	0.51483982	3.34241771	22.076206	4.26E-05	0.00936247
ENSBTAG00000012805	TSPAN13	526460	protein-coding	-0.512014	6.20661429	22.0266882	4.32E-05	0.00936322
ENSBTAG00000015164	SLC27A5	533016	protein-coding	-0.9716295	1.41465392	21.8687329	4.54E-05	0.00969235
ENSBTAG00000010652	PDE4C	524624	protein-coding	-0.8130089	5.66632179	21.7788198	4.68E-05	0.00982297
ENSBTAG00000029906	MIR133A-2	100313253	ncRNA	-0.8484847	4.54766606	21.5197775	5.08E-05	0.01035516
ENSBTAG00000051090	KLF13	789710	protein-coding	-0.9581861	2.01089512	21.469219	5.16E-05	0.01037277
ENSBTAG00000015599	PRKD2	782793	protein-coding	-0.5711914	4.36989469	21.0216475	5.95E-05	0.01180027
ENSBTAG00000008350	KLHL21	506632	protein-coding	-0.6161633	6.37956486	20.8971747	6.20E-05	0.01192071
ENSBTAG00000011864				-0.6402114	3.8590172	20.8049094	6.38E-05	0.01192071
ENSBTAG00000006832	SLC5A6	516021	protein-coding	-0.7598421	3.37733	20.7515777	6.49E-05	0.01192071
ENSBTAG00000047836	LOC785629	785629	pseudo	-0.6495887	2.27718264	20.7261186	6.55E-05	0.01192071
ENSBTAG00000013406	CSRP2	539381	protein-coding	-0.8710917	2.20165045	20.7099061	6.58E-05	0.01192071
ENSBTAG00000024605	DYNLL1	404151	protein-coding	-0.5901687	3.19108492	20.3161313	7.48E-05	0.0129953
ENSBTAG00000024509	TMEM52	617403	protein-coding	-0.9651804	2.18952828	20.1349567	7.93E-05	0.01361888
ENSBTAG00000009418	FAM131B	617268	protein-coding	-1.9164568	0.1056641	19.9907839	8.32E-05	0.01410534
ENSBTAG00000027635	PLPP5	616337	protein-coding	-0.6482454	2.82246623	19.7960011	8.87E-05	0.0148588
ENSBTAG00000011617	MAMSTR	505540	protein-coding	-0.9202103	5.28737126	19.5594227	9.59E-05	0.01533552
ENSBTAG00000003130	CHRNA3	282702	protein-coding	1.24667456	1.52878788	19.3773033	0.00010184	0.01610701
ENSBTAG00000015980	FASN	281152	protein-coding	1.16193952	5.18927207	19.2450339	0.00010642	0.0164616
ENSBTAG00000011803	MYH3	281338	protein-coding	0.60141853	6.20151445	18.9601576	0.00011705	0.01771601
ENSBTAG00000016294	ZNF438	510512	protein-coding	0.59723488	1.8868881	18.6508383	0.00012988	0.01904331
ENSBTAG00000025942	HPGD	512259	protein-coding	-0.9407792	1.94850966	18.610419	0.00013166	0.01910581
ENSBTAG00000050406				-0.9436442	1.22196418	18.3046322	0.00014603	0.02055503
ENSBTAG00000049358	ZNF287	514947	protein-coding	0.54095709	2.4770329	18.2362137	0.00014947	0.02079801
ENSBTAG00000012314	LDLR	281276	protein-coding	1.19670777	2.66185065	18.0401029	0.0001598	0.02102255
ENSBTAG00000004893	ACOX3	510065	protein-coding	-0.5935566	4.3671705	17.8048855	0.00017322	0.02215845
ENSBTAG00000017560	CEBPG	617530	protein-coding	0.50034488	3.49592912	17.779477	0.00017474	0.02215845
ENSBTAG00000017528	SNAI3	526694	protein-coding	-1.5604398	2.72648565	17.7459328	0.00017676	0.02217799
ENSBTAG00000001762	RFXANK	100125917	protein-coding	-0.5225187	3.74122443	17.7249881	0.00017804	0.02217799
ENSBTAG00000052146	MSANTD3	534309	protein-coding	0.79755163	1.57485374	17.6253516	0.00018426	0.02262449
ENSBTAG00000002531	ARHGAP10	510704	protein-coding	0.51805397	5.00162767	17.5350144	0.0001901	0.02286993
ENSBTAG00000003512	MYH7B	521764	protein-coding	-0.6732295	7.71129595	17.4661107	0.00019468	0.02307321
ENSBTAG00000001835	GJA1	281193	protein-coding	-0.6767206	2.72424799	17.0888496	0.00022195	0.02503165
ENSBTAG00000038397	BOLA-DRB2	538700	protein-coding	2.44291715	-0.1292263	17.0413714	0.00022566	0.02503165

ENSBTAG00000007816	LOC787045	787045	pseudo	-1.5020387	3.4291056	17.0233421	0.00022709	0.02503165
ENSBTAG00000003049	MICALL2	510644	protein-coding	-0.5720565	3.48722919	16.9943174	0.0002294	0.02503165
ENSBTAG00000043545				-0.9338317	2.57688579	16.7112111	0.00025336	0.02686489
ENSBTAG00000025424	NOTCH4	536128	protein-coding	-0.5638943	5.14049173	16.6718336	0.00025689	0.02692734
ENSBTAG00000008854	RFX1	789747	protein-coding	-0.6322931	3.01719206	16.6568469	0.00025825	0.02692734
ENSBTAG00000008921	NEXN	504819	protein-coding	0.6393117	8.67976771	16.5993433	0.00026354	0.02727683
ENSBTAG00000020829	PTPRK	509657	protein-coding	-0.5453659	2.91828756	16.5298932	0.00027009	0.0273626
ENSBTAG00000006349	EXTL1	511060	protein-coding	-0.965311	3.96425233	16.4959245	0.00027335	0.0273626
ENSBTAG00000025337	MYH13	511687	protein-coding	0.60064985	6.96722339	16.4926996	0.00027366	0.0273626
ENSBTAG00000018156	KRTCAP3	508550	protein-coding	-0.8331398	1.65161356	16.293486	0.00029369	0.02812755
ENSBTAG00000049218				-1.1016792	1.91725337	16.2752344	0.0002956	0.02812755
ENSBTAG00000003120	ZNF385B	613328	protein-coding	1.01159137	1.85641527	16.2081053	0.00030274	0.02823099
ENSBTAG00000007888	EFR3B	517590	protein-coding	-0.8987772	1.8326351	16.1655415	0.00030736	0.02827749
ENSBTAG00000048982				-0.6177871	3.86743951	16.1093237	0.00031359	0.02847776
ENSBTAG00000003541	ZNF614	100137855	protein-coding	0.5937067	2.62800478	16.0809421	0.00031678	0.02858327
ENSBTAG00000002644	KCNQ4	528741	protein-coding	-0.5626554	5.7925016	16.0524077	0.00032002	0.02869212
ENSBTAG00000049453	OR51A25	785036	protein-coding	-1.9187211	-0.1866385	15.9616119	0.00033059	0.02875958
ENSBTAG00000018562	LDAF1	784639	protein-coding	0.82602236	5.32968079	15.891963	0.00033894	0.0292694
ENSBTAG00000021538	MYO1E	523485	protein-coding	-0.683684	3.97209178	15.7395945	0.00035801	0.03028359
ENSBTAG00000012126	PNPLA7	536255	protein-coding	-0.5053372	2.98286663	15.7296579	0.00035929	0.03028359
ENSBTAG00000047801	CRELD2	515222	protein-coding	-0.7718869	1.77290842	15.6968085	0.00036356	0.0304615
ENSBTAG00000052213	ADIPOQ	282865	protein-coding	2.94594398	1.51973568	15.6488527	0.0003699	0.03080916
ENSBTAG00000021407	LOC788915	788915	protein-coding	-0.9891909	3.20872595	15.6238342	0.00037326	0.03090557
ENSBTAG00000015343	DYNC2I2	531489	protein-coding	-0.5960553	2.23120486	15.5650607	0.00038126	0.03121392
ENSBTAG00000011896	GRIP2	509537	protein-coding	-0.8146321	4.13042848	15.5135941	0.00038842	0.03142194
ENSBTAG00000000658				-0.6937541	3.21030276	15.4618117	0.00039577	0.03165261
ENSBTAG00000003152	IFI27	507138	protein-coding	-1.2353106	4.69779431	15.397245	0.00040514	0.03221902
ENSBTAG00000020289	ZNF214	100140999	protein-coding	1.51003855	-0.7913046	15.355249	0.00041136	0.03238721
ENSBTAG00000027654	EIF4EBP1	509613	protein-coding	-0.5271392	5.33164861	15.3210375	0.00041651	0.03257082
ENSBTAG00000018527	HDGFL3	784070	protein-coding	-0.5060757	3.239157	15.2154154	0.00043282	0.03359143
ENSBTAG00000023179	TRIB1	521857	protein-coding	-0.6233215	5.81052352	15.1345552	0.00044578	0.03400561
ENSBTAG00000004039	DHX35	513148	protein-coding	0.56792531	2.59559779	15.0985001	0.00045168	0.03400561
ENSBTAG00000024815	ANKRD28	529062	protein-coding	0.50043949	4.46554042	15.0924409	0.00045268	0.03400561
ENSBTAG00000012079	MYLK4	518025	protein-coding	-0.9743884	3.38755201	14.9863994	0.00047059	0.03486316
ENSBTAG00000021938	ARHGAP44	509257	protein-coding	-0.8340587	1.93662899	14.9321296	0.00048004	0.03537733
ENSBTAG00000014616	SEPSECS	518874	protein-coding	0.60974445	2.85811339	14.8812588	0.00048909	0.03560313
ENSBTAG00000054572	PEG3	444864	protein-coding	-0.6891016	3.7269962	14.8537508	0.00049405	0.03560313
ENSBTAG00000010365	SQOR	507858	protein-coding	-0.504968	3.5438604	14.7231647	0.00051838	0.03612206
ENSBTAG00000010457	NUAK1	519892	protein-coding	0.54508037	2.98497029	14.7084584	0.00052119	0.03613952
ENSBTAG00000019577	ACKR4	281672	protein-coding	-0.8931528	0.87882967	14.5878169	0.00054494	0.03705562
ENSBTAG00000048771				-0.7387928	1.75797531	14.541086	0.00055444	0.03718702
ENSBTAG00000032869	RNASE10	100140137	protein-coding	1.0964689	1.38473508	14.5400515	0.00055465	0.03718702
ENSBTAG00000012854	GSDMB	509296	protein-coding	-0.7591873	1.94351893	14.4698896	0.00056926	0.03742637

ENSBTAG00000015274	PRL	280901	protein-coding	-2.0532795	-0.6367476	14.4302779	0.00057769	0.03742637
ENSBTAG00000013569	CD38	327677	protein-coding	-0.9117996	1.6931239	14.3913916	0.00058609	0.03749915
ENSBTAG00000015490	HS1BP3	526092	protein-coding	-0.6778832	2.75631986	14.274218	0.00061221	0.03864321
ENSBTAG00000000510	ATG101	513049	protein-coding	-0.6636382	4.64676997	14.2304051	0.00062229	0.03910422
ENSBTAG00000008509	SLC38A3	326576	protein-coding	-0.7261662	1.17555339	14.2051119	0.00062819	0.03924106
ENSBTAG00000052883	NANOS1	786356	protein-coding	0.79754931	1.57933327	14.1579914	0.00063934	0.03924106
ENSBTAG00000020745	HIVEP3	509866	protein-coding	0.82538825	1.41622359	14.130451	0.00064596	0.03924106
ENSBTAG00000025129	MNT	618576	protein-coding	-0.7716143	3.18886214	13.9879423	0.00068138	0.0404624
ENSBTAG00000044185	SOX6	534321	protein-coding	0.57685554	4.45175684	13.9772385	0.00068412	0.0404624
ENSBTAG00000049862				1.66659859	-0.6179587	13.829165	0.00072329	0.04207034
ENSBTAG00000001592	INSIG1	511899	protein-coding	0.72066002	2.89509642	13.7262262	0.00075193	0.04302479
ENSBTAG00000011534	RAMP1	617017	protein-coding	-0.5346011	3.55439022	13.7020512	0.00075882	0.04324375
ENSBTAG00000001086	FHL2	510008	protein-coding	-1.1785084	0.22594739	13.6540852	0.00077271	0.04365544
ENSBTAG00000002914	GALNT18	614203	protein-coding	-0.784265	3.05875961	13.6440445	0.00077565	0.04365544
ENSBTAG00000014319	DNM3	506315	protein-coding	-0.8474463	0.71016824	13.634525	0.00077845	0.04365544
ENSBTAG00000053188				-0.5735116	1.77358404	13.6228796	0.00078189	0.04367433
ENSBTAG00000020626	ERCC6L2	508357	protein-coding	0.50277215	3.28398104	13.5739142	0.00079653	0.04386339
ENSBTAG00000021357	ZNF518A	524691	protein-coding	0.51286249	3.23121921	13.5607462	0.00080052	0.04386339
ENSBTAG00000000256				-0.5727529	4.31768973	13.5576017	0.00080148	0.04386339
ENSBTAG00000018204	MYH1	281337	protein-coding	0.51292107	15.0968718	13.505309	0.00081754	0.04418419
ENSBTAG00000006081	PPM1J	100848925	protein-coding	-0.5352886	4.93492318	13.4966147	0.00082025	0.04418419
ENSBTAG00000005012	HSPH1	507165	protein-coding	-0.6074174	5.28098267	13.4875878	0.00082306	0.04418419
ENSBTAG00000005928	MINPP1	541067	protein-coding	0.50963669	3.15416506	13.4727237	0.00082773	0.04418419
ENSBTAG00000019432	LOC616254	616254	protein-coding	-0.5121051	3.99156939	13.4262083	0.0008425	0.04475097
ENSBTAG00000024407	LOC787530	787530	pseudo	-0.5721467	2.92578335	13.3798133	0.00085751	0.04511587
ENSBTAG00000043961	MPDZ	536863	protein-coding	-0.5092575	6.99785008	13.3753161	0.00085898	0.04511587
ENSBTAG00000010564	ELOVL6	533333	protein-coding	1.43170578	1.28516024	13.1961465	0.00091981	0.04674111
ENSBTAG00000051791	MIR29D	100313025	ncRNA	-0.8943442	0.38388725	13.1255059	0.00094504	0.04767902
ENSBTAG00000051333	ARID5B	783538	protein-coding	-1.4129229	5.91096226	13.0922097	0.00095719	0.04796262
ENSBTAG00000002858	ACBD7	508284	protein-coding	1.37371482	0.29742214	13.0360036	0.00097808	0.04796262
ENSBTAG00000005583	HYI	515954	protein-coding	-0.5485477	2.59283138	13.0286742	0.00098084	0.04796262
ENSBTAG00000037794	MYH4	408020	protein-coding	0.55750545	11.4082534	13.0074122	0.00098889	0.04796262
ENSBTAG00000051936				-0.6272081	2.89906994	12.9827411	0.00099832	0.04812445
ENSBTAG00000048659				0.95346911	1.13017522	12.9712695	0.00100274	0.04817244
ENSBTAG00000002144	ADRB2	281605	protein-coding	-0.6236252	4.40983118	12.8852977	0.00103652	0.04929051
ENSBTAG00000027080	SLC8A3	540307	protein-coding	0.5478509	4.95633001	12.8573121	0.00104777	0.04965801
ENSBTAG00000009599	LCN1	511158	protein-coding	-3.4937919	0.41140639	12.819728	0.00106309	0.04997437
ENSBTAG00000037526	FABP4	281759	protein-coding	2.65514508	2.1718995	12.8148549	0.0010651	0.04997437

3. Prospected DEGs to T2 vs T1 in Conventional Weaning group

ENSEMBL	SYMBOL	ENTREZID	GENETYPE	logFC	logCPM	F	PValue	FDR
ENSBTAG00000002126	*PFKFB2*	287019	protein-coding	1.66346812	4.1299211	89.7241145	4.85E-11	6.82E-07
ENSBTAG00000021970	*EYA4*	538786	protein-coding	-1.329733	6.82448232	80.1049911	1.94E-10	1.14E-06
ENSBTAG00000003512	*MYH7B*	521764	protein-coding	1.50880945	7.71129595	78.5895369	2.44E-10	1.14E-06
ENSBTAG00000048271	*MICAL3*	532244	protein-coding	-1.0587176	6.4044722	71.7033178	7.19E-10	2.53E-06
ENSBTAG00000010786	*TACC2*	533768	protein-coding	-0.9785381	8.37452628	67.1239714	1.54E-09	4.33E-06
ENSBTAG00000016411	*RNF122*	510037	protein-coding	1.13205066	4.86033306	64.3915494	2.46E-09	5.77E-06
ENSBTAG00000015543	*ARHGAP35*	540310	protein-coding	-0.8976848	6.83932883	62.5035446	3.43E-09	6.90E-06
ENSBTAG00000017528	*SNAI3*	526694	protein-coding	3.19978614	2.72648565	61.5232383	4.09E-09	7.20E-06
ENSBTAG00000000698	*MYO18B*	540437	protein-coding	-0.7776952	8.34917908	57.4447858	8.67E-09	1.36E-05
ENSBTAG00000043961	*MPDZ*	536863	protein-coding	1.10301688	6.99785008	56.7397574	9.90E-09	1.39E-05
ENSBTAG00000015888	*PDE7B*	522509	protein-coding	-0.8824184	4.07166774	54.6654606	1.47E-08	1.89E-05
ENSBTAG00000022058	*UNG*	511381	protein-coding	-1.2356837	7.9015851	53.955633	1.69E-08	1.97E-05
ENSBTAG00000031439	*CDC14A*	614777	protein-coding	-1.0750704	3.89863622	53.5938431	1.82E-08	1.97E-05
ENSBTAG00000006189	*ACTG1*	404122	protein-coding	0.75313939	8.2761483	52.6490082	2.19E-08	2.20E-05
ENSBTAG00000003514	*HSF4*	614788	protein-coding	0.9250284	4.74972679	51.8325816	2.58E-08	2.30E-05
ENSBTAG00000021920	*SEMA4C*	526028	protein-coding	0.88790628	4.13611933	51.6357375	2.68E-08	2.30E-05
ENSBTAG00000007895	*SLC20A1*	514255	protein-coding	1.49781031	5.69488037	51.0668363	3.01E-08	2.30E-05
ENSBTAG00000010392	*ESRRG*	536732	protein-coding	-2.6387879	1.19031337	51.0362248	3.03E-08	2.30E-05
ENSBTAG00000021294	*VWA1*	505917	protein-coding	1.16991132	3.1358257	50.9193093	3.10E-08	2.30E-05
ENSBTAG00000008921	*NEXN*	504819	protein-coding	-1.1434548	8.67976771	49.5294485	4.12E-08	2.90E-05
ENSBTAG00000012854	*GSDMB*	509296	protein-coding	1.4741565	1.94351893	49.1245449	4.49E-08	3.01E-05
ENSBTAG00000032534	*RHOF*	617894	protein-coding	0.88711866	4.59044253	48.6050971	5.00E-08	3.20E-05
ENSBTAG00000037449	*ACSM5*	100298888	protein-coding	1.04796437	4.93712704	47.9140997	5.78E-08	3.34E-05
ENSBTAG00000015119	*SIK3*	506539	protein-coding	-0.8283015	5.50892163	47.6588621	6.10E-08	3.34E-05
ENSBTAG00000005754	*PPM1K*	540329	protein-coding	1.40867956	5.35383935	47.645196	6.12E-08	3.34E-05
ENSBTAG00000014420	*AGER*	280986	protein-coding	1.58197388	1.52424487	47.6056104	6.17E-08	3.34E-05
ENSBTAG00000050814	*CFAP70*	529891	protein-coding	1.40383837	1.92829515	47.2910748	6.59E-08	3.44E-05
ENSBTAG00000046664	*TSC22D1*	510359	protein-coding	-0.8882267	5.72630658	47.0829405	6.89E-08	3.46E-05
ENSBTAG00000001609	*MAP2K6*	286883	protein-coding	-1.077206	6.11989122	46.4006615	7.98E-08	3.87E-05
ENSBTAG00000026199	*ACTB*	280979	protein-coding	0.68748308	8.92285982	45.7757192	9.13E-08	4.28E-05
ENSBTAG00000007214	*ELL2*	782605	protein-coding	1.56485488	4.19328693	45.2825428	1.02E-07	4.55E-05
ENSBTAG00000020457	*TGFB1*	282089	protein-coding	0.98406842	3.49158712	45.2015556	1.03E-07	4.55E-05
ENSBTAG00000005857	*SLC6A1*	407118	protein-coding	1.3199162	4.86457922	44.9423416	1.09E-07	4.67E-05
ENSBTAG00000021134	*DPP9*	521100	protein-coding	-0.5578257	5.12847709	44.6476449	1.17E-07	4.82E-05
ENSBTAG00000043979	*H1-9*	615867	protein-coding	1.3331957	2.80727203	44.5320355	1.20E-07	4.82E-05
ENSBTAG00000001143	*LOC512486*	512486	protein-coding	1.47475751	4.09341919	44.3587456	1.24E-07	4.86E-05
ENSBTAG00000016220	*WIPF3*	786606	protein-coding	-0.973471	4.21942509	43.8018027	1.41E-07	5.35E-05
ENSBTAG00000037735	*C5AR2*	522144	protein-coding	-2.3537189	2.68082591	43.4833822	1.51E-07	5.60E-05
ENSBTAG00000051572	*ZNF646*	783820	protein-coding	-0.7340699	3.34241771	42.8575285	1.74E-07	6.28E-05
ENSBTAG00000051759				-0.6901826	4.09858522	42.2229596	2.01E-07	7.06E-05
ENSBTAG00000020658	*LNX1*	540694	protein-coding	-1.18291	2.5475883	40.6908996	2.85E-07	9.79E-05

ENSBTAG00000052248				-1.4874289	2.17192557	40.4808621	3.00E-07	0.00010039
ENSBTAG00000008509	*SLC38A3*	326576	protein-coding	1.31547877	1.17555339	40.3357969	3.10E-07	0.00010043
ENSBTAG00000018770	*HSDL1*	505213	protein-coding	0.88184347	2.2021102	40.1761556	3.22E-07	0.00010043
ENSBTAG00000010543	*FGFR4*	317696	protein-coding	1.0882867	3.39138072	40.1598712	3.23E-07	0.00010043
ENSBTAG00000001027	*SERPINH1*	510850	protein-coding	0.94265512	6.48306976	40.0894426	3.28E-07	0.00010043
ENSBTAG00000046282	*VGLL2*	541260	protein-coding	1.24102421	6.47862384	39.9448072	3.40E-07	0.00010168
ENSBTAG00000015511	*FADS3*	515925	protein-coding	0.97586287	4.0465563	38.942245	4.30E-07	0.00012357
ENSBTAG00000018238	*ABTB1*	507417	protein-coding	0.67506839	5.03545884	38.2739002	5.05E-07	0.00013962
ENSBTAG00000011896	*GRIP2*	509537	protein-coding	1.34368397	4.13042848	38.2595031	5.06E-07	0.00013962
ENSBTAG00000051791	*MIR29D*	100313025	ncRNA	1.66275004	0.38388725	38.1830489	5.16E-07	0.00013962
ENSBTAG00000018562	*LDAF1*	784639	protein-coding	-1.2854062	5.32968079	37.1267435	6.66E-07	0.0001705
ENSBTAG00000019302	*BCL2*	281020	protein-coding	-1.3865576	2.14262997	37.0337342	6.82E-07	0.00017131
ENSBTAG00000015599	*PRKD2*	782793	protein-coding	0.80959272	4.36989469	36.6732013	7.44E-07	0.00018385
ENSBTAG00000014215	*CCDC86*	540913	protein-coding	-0.9135105	3.39966444	36.3769128	8.01E-07	0.00019436
ENSBTAG00000025129	*MNT*	618576	protein-coding	1.26978341	3.18886214	36.1329493	8.51E-07	0.00020295
ENSBTAG00000015580	*TLE3*	514326	protein-coding	0.981724	3.5147095	35.9723858	8.85E-07	0.00020768
ENSBTAG00000009050	*ADIPOR2*	407234	protein-coding	-0.9701998	6.01592529	35.8932494	9.03E-07	0.00020833
ENSBTAG00000044202	*CNKSR2*	534112	protein-coding	-0.9621799	3.71216559	35.6546383	9.58E-07	0.00021539
ENSBTAG00000001762	*RFXANK*	100125917	protein-coding	0.76512315	3.74122443	35.6231878	9.66E-07	0.00021539
ENSBTAG00000012861	*KIF4A*	537127	protein-coding	1.24913546	2.63058813	35.5669736	9.79E-07	0.00021539
ENSBTAG00000036363	*MIR133A-1*	100313361	ncRNA	1.62305847	1.47350077	35.4537561	1.01E-06	0.00021597
ENSBTAG00000008688	*HEYL*	538609	protein-coding	0.75469759	5.19982095	35.3941873	1.02E-06	0.00021597
ENSBTAG00000016532	*MAPKAPK3*	615215	protein-coding	-0.5751995	5.59402952	35.3730765	1.03E-06	0.00021597
ENSBTAG00000015509	*NAMPT*	520472	protein-coding	-1.2630636	6.65944432	35.2376628	1.06E-06	0.00021883
ENSBTAG00000000172	*PFKFB1*	282304	protein-coding	0.59485085	5.37531359	35.2033716	1.07E-06	0.00021883
ENSBTAG00000009579	*CEP85*	517520	protein-coding	-0.860725	6.36001922	34.6124913	1.25E-06	0.00024477
ENSBTAG00000008940	*NPTX1*	513538	protein-coding	-2.5216339	2.238817	34.60096	1.25E-06	0.00024477
ENSBTAG00000020736	*CD40*	286849	protein-coding	1.59817787	1.59892572	34.5909606	1.25E-06	0.00024477
ENSBTAG00000053346	*SPSB2*	613651	protein-coding	0.86180516	3.5438641	34.4270868	1.31E-06	0.00025168
ENSBTAG00000008348	*NOL4L*	785034	protein-coding	1.2179769	2.48798346	34.2743373	1.36E-06	0.00025716
ENSBTAG00000002271	*CDADC1*	512184	protein-coding	-0.615911	4.76604252	34.2363942	1.37E-06	0.00025716
ENSBTAG00000020944	*PIEZO1*	789183	protein-coding	0.64164212	5.35326695	33.8385175	1.52E-06	0.00028096
ENSBTAG00000017597	*ESCO1*	535035	protein-coding	-0.6583404	4.40303912	33.3178411	1.73E-06	0.00031305
ENSBTAG00000032996	*P4HA1*	518288	protein-coding	0.55293508	4.79769172	33.2403693	1.77E-06	0.00031535
ENSBTAG00000002315	*RNF34*	506764	protein-coding	-0.520975	5.27007697	33.1374691	1.82E-06	0.00031982
ENSBTAG00000001851	*LOC508916*	508916	protein-coding	2.33450697	2.41501041	32.9837087	1.89E-06	0.00032874
ENSBTAG00000025372	*RIOX1*	511031	protein-coding	-0.6831767	2.53621325	32.7045057	2.03E-06	0.00034924
ENSBTAG00000019061	*ELF4*	504514	protein-coding	0.95312986	3.58320498	32.4364752	2.18E-06	0.00037011
ENSBTAG00000008411	*PLEKHO2*	613696	protein-coding	0.66295141	3.69697939	32.0305009	2.43E-06	0.00040214
ENSBTAG00000006960	*DHX16*	506405	protein-coding	-0.5039179	4.70226688	31.9640849	2.47E-06	0.00040394
ENSBTAG00000014151	*RCSD1*	512205	protein-coding	0.63780309	6.41129436	31.9255439	2.50E-06	0.00040394
ENSBTAG00000013666	*SLC25A29*	507686	protein-coding	0.91030545	3.0706802	31.8245471	2.56E-06	0.00041017
ENSBTAG00000013855	*ORMDL3*	615368	protein-coding	0.62124953	4.99591042	31.3798958	2.89E-06	0.00045644

ENSBTAG00000046337	*TUBB6*	534206	protein-coding	1.01996415	4.0969528	31.222134	3.01E-06	0.0004708
ENSBTAG00000018352	*ABRA*	539379	protein-coding	1.24865039	5.00194902	31.0982454	3.11E-06	0.00047483
ENSBTAG00000002527	*ZSWIM4*	781514	protein-coding	-1.1126854	2.89742256	31.0649042	3.14E-06	0.00047483
ENSBTAG00000019315	*PLA2G7*	282311	protein-coding	-1.1660494	3.71616605	31.0223275	3.18E-06	0.00047483
ENSBTAG00000018663	*GPRC5C*	535664	protein-coding	0.69863758	5.88936435	30.989818	3.20E-06	0.00047483
ENSBTAG00000007767	*TBX15*	521974	protein-coding	-0.6128449	6.82483376	30.9885116	3.20E-06	0.00047483
ENSBTAG00000004553	*TPM4*	535277	protein-coding	0.90099937	6.19028934	30.9363479	3.25E-06	0.00047651
ENSBTAG00000014724	*EIF4A2*	286819	protein-coding	-0.5375516	8.79584183	30.6844451	3.48E-06	0.00049955
ENSBTAG00000002321	*AMT*	280719	protein-coding	0.6249744	4.52500815	30.3543612	3.80E-06	0.00053528
ENSBTAG00000014782	*STAB1*	505543	protein-coding	0.86597536	4.32708212	30.2533029	3.91E-06	0.00053963
ENSBTAG00000011034	*ANGPT2*	282141	protein-coding	1.15126387	2.13647254	30.251594	3.91E-06	0.00053963
ENSBTAG00000052678	*MIR29C*	791043	ncRNA	1.4115451	1.60037987	30.0634885	4.12E-06	0.00056246
ENSBTAG00000003510	*MMRN2*	512308	protein-coding	0.85431139	4.47623494	29.8956438	4.31E-06	0.0005794
ENSBTAG00000003721	*CHST1*	506932	protein-coding	0.89493255	3.43501149	29.8843545	4.32E-06	0.0005794
ENSBTAG00000003876	*MCU*	536656	protein-coding	-0.6450128	5.38453111	29.7490673	4.49E-06	0.00059558
ENSBTAG00000048659				-1.487631	1.13017522	29.7055088	4.54E-06	0.00059711
ENSBTAG00000018603	*RGMA*	518756	protein-coding	0.83314516	3.92513225	29.5733172	4.71E-06	0.00061344
ENSBTAG00000019585	*MYOM1*	538404	protein-coding	-0.5141212	10.3223038	29.3978971	4.94E-06	0.00063354
ENSBTAG00000017860	*HIPK2*	510158	protein-coding	-1.1197403	3.58659217	29.3595659	4.99E-06	0.00063354
ENSBTAG00000018975	*KCNT1*	529468	protein-coding	0.74858425	3.80232538	29.3566566	5.00E-06	0.00063354
ENSBTAG00000020060	*TXNIP*	506790	protein-coding	-0.9296964	10.6402975	28.6046083	6.16E-06	0.0007733
ENSBTAG00000003215	*KIF3B*	508404	protein-coding	-0.5635884	3.24870746	28.4605358	6.41E-06	0.00079131
ENSBTAG00000015839	*MAP4*	281295	protein-coding	-0.5602146	9.36785856	28.3624641	6.59E-06	0.00080629
ENSBTAG00000037729	*CERS1*	615296	protein-coding	0.60819133	3.00000023	28.286613	6.73E-06	0.00081654
ENSBTAG00000051239				0.55076996	5.34565346	28.2471069	6.80E-06	0.00081859
ENSBTAG00000019604	*VASP*	514902	protein-coding	0.5923565	4.3872949	28.086328	7.12E-06	0.00084388
ENSBTAG00000012450	*RAPGEF2*	100139484	protein-coding	-0.8733091	6.00635017	28.078813	7.13E-06	0.00084388
ENSBTAG00000012172	*MIDN*	523615	protein-coding	1.17129181	5.0338681	28.0482118	7.20E-06	0.00084409
ENSBTAG00000014508	*FBXO40*	613597	protein-coding	-1.1114037	7.54329817	27.8895341	7.53E-06	0.00087548
ENSBTAG00000003885	*PEX2*	512677	protein-coding	-0.6706478	2.97822877	27.7849183	7.75E-06	0.00088641
ENSBTAG00000015698	*FYCO1*	100139246	protein-coding	-0.5683737	7.42253838	27.7301997	7.87E-06	0.00088641
ENSBTAG00000012370	*MGP*	282660	protein-coding	-0.8373798	5.65136816	27.7031443	7.93E-06	0.00088641
ENSBTAG00000018114	*ACVR2A*	281598	protein-coding	-0.5539023	4.24008406	27.6609325	8.03E-06	0.00088994
ENSBTAG00000040206	*ZNF770*	786537	protein-coding	-0.6022032	4.32149864	27.532392	8.33E-06	0.00090551
ENSBTAG00000005666	*LRRC20*	521721	protein-coding	0.58246894	5.98310186	27.517188	8.36E-06	0.00090551
ENSBTAG00000012302	*RTN4RL1*	539358	protein-coding	1.33558157	2.0175335	27.5003028	8.40E-06	0.00090551
ENSBTAG00000014479	*TUBGCP4*	510865	protein-coding	-0.6377373	3.28378436	27.4908344	8.43E-06	0.00090551
ENSBTAG00000011467	*BATF2*	522469	protein-coding	1.51944616	2.33919754	27.3974628	8.65E-06	0.00092288
ENSBTAG00000047605	*LOC100337457*	100337457	protein-coding	-0.9783935	4.24304455	27.3673737	8.73E-06	0.00092384
ENSBTAG00000052909	*C8G*	517356	protein-coding	0.7118863	3.42797568	27.3005401	8.90E-06	0.00093461
ENSBTAG00000019805	*RHBDF1*	529167	protein-coding	0.75930078	4.49216785	27.2410133	9.05E-06	0.00094361
ENSBTAG00000000053	*FILIP1*	514193	protein-coding	-0.7973362	6.01615451	27.1282013	9.35E-06	0.00096478
ENSBTAG00000039190	*SLC9A5*	512686	protein-coding	1.21116334	5.27380074	27.0834272	9.47E-06	0.00096478

ENSBTAG00000016282	*EIF5*	504752	protein-coding	-0.595405	8.18958866	27.080076	9.48E-06	0.00096478
ENSBTAG00000018823	*GRN*	767942	protein-coding	0.60077546	5.04166258	27.0548539	9.55E-06	0.00096478
ENSBTAG00000018156	*KRTCAP3*	508550	protein-coding	1.15277756	1.65161356	27.0197439	9.64E-06	0.00096478
ENSBTAG00000025494	*PAQR8*	531018	protein-coding	-1.0687848	1.98609242	26.9856874	9.74E-06	0.00096478
ENSBTAG00000006870	*RASGEF1A*	514877	protein-coding	1.19825541	1.77533818	26.9583287	9.81E-06	0.00096478
ENSBTAG00000010637	*ARSG*	513067	protein-coding	-1.6026112	2.75580936	26.9555713	9.82E-06	0.00096478
ENSBTAG00000038316	*GPATCH8*	534759	protein-coding	0.57155829	6.58935871	26.9385395	9.87E-06	0.00096478
ENSBTAG00000009183	*SHISA5*	616861	protein-coding	0.68830315	4.00963183	26.8777643	1.00E-05	0.00097503
ENSBTAG00000017071	*C1QTNF3*	531659	protein-coding	0.92231051	4.53285159	26.7908699	1.03E-05	0.00099289
ENSBTAG00000050314				1.73552821	0.5728727	26.7500205	1.04E-05	0.00099782
ENSBTAG00000051340				-2.5719316	1.24945426	26.6095927	1.09E-05	0.00102518
ENSBTAG00000002402	*IGF2R*	281849	protein-coding	-0.7461911	7.03267572	26.5717101	1.10E-05	0.00102956
ENSBTAG00000009042	*ATXN7L2*	541097	protein-coding	0.88523136	3.91236319	26.472816	1.13E-05	0.00104631
ENSBTAG00000009679	*PLEKHG1*	525772	protein-coding	0.99068494	3.82674798	26.4702585	1.13E-05	0.00104631
ENSBTAG00000005259	*UCP3*	281563	protein-coding	-1.5359258	8.34374545	26.4142258	1.15E-05	0.0010565
ENSBTAG00000005408	*CLK1*	613808	protein-coding	0.50378614	7.11612677	26.3615621	1.17E-05	0.00106016
ENSBTAG00000004039	*DHX35*	513148	protein-coding	-0.7533174	2.59559779	26.3575804	1.17E-05	0.00106016
ENSBTAG00000019327	*NRAP*	532640	protein-coding	-0.9917438	10.778737	26.2872875	1.19E-05	0.00106705
ENSBTAG00000025898	*TBC1D8*	527162	protein-coding	-0.7168983	3.82474485	26.2837925	1.19E-05	0.00106705
ENSBTAG00000000111	*UGP2*	281565	protein-coding	-0.7098667	7.98697257	26.0952792	1.26E-05	0.00111551
ENSBTAG00000001576	*TMEM140*	515475	protein-coding	-0.9669927	4.55723336	26.0333047	1.28E-05	0.00112878
ENSBTAG00000005847	*ROCK2*	282041	protein-coding	-1.1086925	7.71001158	26.0118035	1.29E-05	0.00112884
ENSBTAG00000015345	*BNIPL*	614493	protein-coding	1.70686025	0.19100621	25.9255465	1.32E-05	0.00115054
ENSBTAG00000018838	*IQCA1L*	101903592	protein-coding	1.91847	3.93250363	25.8758862	1.34E-05	0.00115316
ENSBTAG00000029906	*MIR133A-2*	100313253	ncRNA	0.97816031	4.54766606	25.8416813	1.36E-05	0.00115772
ENSBTAG00000035844	*PLAAT1*	540539	protein-coding	0.51428626	5.98718757	25.820394	1.37E-05	0.00115795
ENSBTAG00000001865	*SGCG*	506155	protein-coding	0.92151034	6.34002917	25.7565419	1.39E-05	0.00117278
ENSBTAG00000023745	*BANP*	513446	protein-coding	0.60923907	3.6340194	25.5473283	1.48E-05	0.00121976
ENSBTAG00000010191	*PAK1*	533729	protein-coding	0.74724105	4.80209477	25.5447765	1.48E-05	0.00121976
ENSBTAG00000012605	*GPR157*	538468	protein-coding	1.22327654	3.43286632	25.5425754	1.48E-05	0.00121976
ENSBTAG00000003209	*BLCAP*	281649	protein-coding	0.50997637	6.60015165	25.4631335	1.52E-05	0.00122098
ENSBTAG00000054434				-0.9087144	1.80328314	25.4519164	1.52E-05	0.00122098
ENSBTAG00000001987	*SWAP70*	533720	protein-coding	0.59368513	4.17210985	25.4426632	1.53E-05	0.00122098
ENSBTAG00000013306	*ZBTB7C*	614110	protein-coding	0.94667431	1.85832752	25.4414931	1.53E-05	0.00122098
ENSBTAG00000036297	*RBFA*	510927	protein-coding	-0.6375721	3.07591559	25.327498	1.58E-05	0.00124614
ENSBTAG00000018307	*SEMA3F*	531141	protein-coding	0.63454092	3.37226012	25.3153442	1.58E-05	0.00124614
ENSBTAG00000015596	*SLCO2B1*	317704	protein-coding	0.69249758	4.47735896	25.2953436	1.59E-05	0.00124657
ENSBTAG00000006370	*CCT3*	504735	protein-coding	-0.5097758	6.44522562	25.1618019	1.66E-05	0.00128266
ENSBTAG00000001823	*STC2*	540573	protein-coding	3.23498363	0.40207964	25.039651	1.72E-05	0.00129999
ENSBTAG00000025458	*DPF3*	787359	protein-coding	-0.9408093	2.72668686	25.0090251	1.74E-05	0.00129999
ENSBTAG00000017616	*ADSS1*	784089	protein-coding	0.50193831	8.05901062	24.9702928	1.76E-05	0.00130752
ENSBTAG00000050008				0.64733156	4.7384194	24.914391	1.79E-05	0.00132249
ENSBTAG00000032055	*VPS39*	520990	protein-coding	-0.5110998	4.49728044	24.8538357	1.82E-05	0.00133954

ENSBTAG00000022799	*NOTCH1*	767866	protein-coding	0.7471118	5.55997429	24.7890579	1.85E-05	0.00134459
ENSBTAG00000012504	*GAS2L1*	518935	protein-coding	0.87560419	2.25565151	24.7526406	1.87E-05	0.00135234
ENSBTAG00000023472	*PPP1R14A*	615348	protein-coding	-0.9621832	2.19380515	24.7288655	1.89E-05	0.00135442
ENSBTAG00000021505	*USP36*	504581	protein-coding	-0.5930246	3.54361593	24.7052917	1.90E-05	0.00135442
ENSBTAG00000054931	*CACNG4*	519331	protein-coding	1.16336649	3.58805924	24.6964651	1.91E-05	0.00135442
ENSBTAG00000048140	*DNAAF5*	100300875	protein-coding	-0.5145969	3.74604032	24.5285235	2.00E-05	0.0014172
ENSBTAG00000009703	*MYH7*	282714	protein-coding	-0.7036243	12.6664818	24.4472076	2.05E-05	0.00143781
ENSBTAG00000012720	*ANKRD2*	100847218	protein-coding	-1.7281639	6.61374099	24.406986	2.08E-05	0.00144002
ENSBTAG00000023445	*SIAH2*	785659	protein-coding	0.5351026	4.29739413	24.4055164	2.08E-05	0.00144002
ENSBTAG00000000312	*GRINA*	510225	protein-coding	0.5235859	4.17845577	24.3904867	2.09E-05	0.00144002
ENSBTAG00000007782	*MYOT*	507884	protein-coding	-0.5972156	11.4351307	24.3766032	2.10E-05	0.00144002
ENSBTAG00000014081	*NBAS*	521982	protein-coding	-0.5652412	4.19263875	24.3502562	2.11E-05	0.00144373
ENSBTAG00000012147	*ARRDC1*	786098	protein-coding	0.81499236	3.32233375	24.3235578	2.13E-05	0.00144373
ENSBTAG00000016593	*FMNL3*	540105	protein-coding	0.61311114	4.15596051	24.3198435	2.13E-05	0.00144373
ENSBTAG00000009903	*PTCH1*	520994	protein-coding	-0.5888013	3.49023292	24.2671135	2.17E-05	0.00145986
ENSBTAG00000005967	*UBR2*	527153	protein-coding	-0.5362523	6.96046734	24.1887612	2.22E-05	0.00148769
ENSBTAG00000053952				-1.1844687	3.40408693	24.1038688	2.28E-05	0.00151218
ENSBTAG00000020030	*FITM2*	518159	protein-coding	-0.7839278	4.86539871	24.1034199	2.28E-05	0.00151218
ENSBTAG00000044150	*UCK2*	541028	protein-coding	-1.08241	1.34428754	23.8877332	2.43E-05	0.00159187
ENSBTAG00000021407	*LOC788915*	788915	protein-coding	1.32274111	3.20872595	23.6810215	2.59E-05	0.00167379
ENSBTAG00000025848	*RBM3*	509771	protein-coding	0.64612675	5.12694798	23.6773932	2.59E-05	0.00167379
ENSBTAG00000002869	*ARL4A*	767906	protein-coding	0.71348783	3.40494112	23.6427181	2.62E-05	0.00168114
ENSBTAG00000031950	*RAB3IP*	533284	protein-coding	0.80513898	2.29162741	23.6282664	2.63E-05	0.00168114
ENSBTAG00000011617	*MAMSTR*	505540	protein-coding	1.06841295	5.28737126	23.6182641	2.64E-05	0.00168114
ENSBTAG00000011224	*CITED2*	521378	protein-coding	-0.8711487	4.28860316	23.5261444	2.71E-05	0.00172137
ENSBTAG00000002145	*MMP17*	513072	protein-coding	1.53193069	0.38122602	23.5019163	2.74E-05	0.0017264
ENSBTAG00000002382	*DDX10*	528483	protein-coding	-0.7189886	1.93088253	23.3786903	2.84E-05	0.00176125
ENSBTAG00000008010	*FLCN*	526652	protein-coding	0.76792439	4.53967829	23.3192853	2.89E-05	0.00178579
ENSBTAG00000002216	*MYOD1*	281938	protein-coding	1.20768264	4.97894654	23.152757	3.04E-05	0.0018543
ENSBTAG00000002566	*PGPEP1*	615089	protein-coding	0.62398414	4.39529601	23.1495195	3.05E-05	0.0018543
ENSBTAG00000019091	*RNPC3*	768060	protein-coding	0.54891112	4.43856048	23.0320164	3.16E-05	0.00188896
ENSBTAG00000012004	*TGFB3*	538957	protein-coding	1.16644722	2.65678485	23.018054	3.17E-05	0.00188896
ENSBTAG00000031231	*IRF1*	789216	protein-coding	0.90195358	5.05933373	23.0112795	3.18E-05	0.00188896
ENSBTAG00000017666	*ABCC5*	511769	protein-coding	0.8312507	6.71263389	22.9627375	3.23E-05	0.00190946
ENSBTAG00000017743	*XIRP2*	506090	protein-coding	-1.2026779	11.272415	22.9170728	3.27E-05	0.00192049
ENSBTAG00000006671	*DDX27*	514567	protein-coding	-0.5179752	4.06137807	22.8928864	3.30E-05	0.00192689
ENSBTAG00000022084				-0.657891	2.53624098	22.8374115	3.36E-05	0.00194416
ENSBTAG00000010284	*ETNPPL*	515186	protein-coding	0.58152208	5.90488622	22.7697653	3.43E-05	0.00196918
ENSBTAG00000012969	*DKK2*	541161	protein-coding	1.16487716	1.69430926	22.707785	3.49E-05	0.00199929
ENSBTAG00000013744	*SYNPO*	533531	protein-coding	-0.5146019	10.5826683	22.6484123	3.56E-05	0.0020283
ENSBTAG00000049747				1.37260341	2.32422844	22.617155	3.59E-05	0.00203986
ENSBTAG00000004732	*SPTB*	527711	protein-coding	-0.6714702	7.89725762	22.5037266	3.72E-05	0.00210477
ENSBTAG00000015457	*FGFR1*	281768	protein-coding	0.55377773	4.45021557	22.4884119	3.74E-05	0.0021064

ENSBTAG00000047955	*ZNF774*	100125278	protein-coding	-1.1297788	3.04824857	22.4447535	3.79E-05	0.00212562
ENSBTAG00000008862	*GOLGB1*	532923	protein-coding	-0.5520886	5.35232065	22.4338005	3.81E-05	0.00212562
ENSBTAG00000016161	*FLII*	514446	protein-coding	-0.5294514	7.15744245	22.3250147	3.94E-05	0.00216145
ENSBTAG00000004005	*WDR7*	536469	protein-coding	-0.6040378	4.15446097	22.3175219	3.95E-05	0.00216145
ENSBTAG00000000981	*KLHDC8B*	527321	protein-coding	0.74712054	2.41840362	22.2670555	4.01E-05	0.00218738
ENSBTAG00000018382	*SMS*	615950	protein-coding	-0.5116069	5.30002678	22.2478589	4.03E-05	0.00219209
ENSBTAG00000038124	*FCGR2A*	782652	protein-coding	3.10483693	-0.4697056	22.2103697	4.08E-05	0.00220949
ENSBTAG00000006832	*SLC5A6*	516021	protein-coding	0.83137883	3.37733	22.1308769	4.18E-05	0.0022469
ENSBTAG00000011266	*ZBTB16*	534401	protein-coding	-1.1090452	5.77516961	22.1136772	4.21E-05	0.0022469
ENSBTAG00000052420	*MIR12060*	114482212	ncRNA	1.22259848	0.64064534	22.103921	4.22E-05	0.0022469
ENSBTAG00000014828	*CACNA1A*	282648	protein-coding	0.81577955	2.0494841	22.0741068	4.26E-05	0.0022469
ENSBTAG00000053162				-0.5075523	5.73037353	22.0723647	4.26E-05	0.0022469
ENSBTAG00000001146	*HIVEP2*	540396	protein-coding	-0.7647712	7.0037666	22.0226858	4.33E-05	0.00226948
ENSBTAG00000025931	*NEU3*	281349	protein-coding	-0.8489044	4.05783798	22.0122296	4.34E-05	0.00226948
ENSBTAG00000040055	*CAB39*	511451	protein-coding	-0.5390979	7.1534487	22.0051122	4.35E-05	0.00226948
ENSBTAG00000005617	*SLC41A3*	524085	protein-coding	0.89102586	3.02221482	21.9719777	4.40E-05	0.00228484
ENSBTAG00000021210	*HES2*	516433	protein-coding	1.14788622	2.51203347	21.8182499	4.62E-05	0.00236778
ENSBTAG00000051920	*MIR29E*	100313099	ncRNA	1.73418541	0.15571823	21.8126177	4.63E-05	0.00236778
ENSBTAG00000045954	*LRRC14B*	100301203	protein-coding	0.79757929	3.79543694	21.7287065	4.75E-05	0.0024107
ENSBTAG00000008849	*SORT1*	511591	protein-coding	-0.8508383	4.95774758	21.7215765	4.76E-05	0.0024107
ENSBTAG00000000815	*EPHA2*	512798	protein-coding	0.69132823	3.37551024	21.6875897	4.81E-05	0.0024183
ENSBTAG00000019347	*PLXDC1*	536989	protein-coding	1.18754713	3.1863339	21.6824119	4.82E-05	0.0024183
ENSBTAG00000003650	*NR4A2*	540245	protein-coding	2.44285027	2.9237212	21.6777744	4.83E-05	0.0024183
ENSBTAG00000011368	*NOC3L*	510425	protein-coding	-0.5238862	3.52751562	21.6364834	4.89E-05	0.00244149
ENSBTAG00000004659	*COQ10B*	514221	protein-coding	1.53005926	3.30852664	21.5809532	4.98E-05	0.00246058
ENSBTAG00000012863	*GDPD2*	514673	protein-coding	1.36573256	1.74049512	21.5786219	4.98E-05	0.00246058
ENSBTAG00000048660	*C19H17orf113*	116601118	protein-coding	-1.8675407	-0.1607789	21.4257424	5.23E-05	0.00254026
ENSBTAG00000053073				1.60302116	1.3347069	21.4237893	5.23E-05	0.00254026
ENSBTAG00000013392	*PLD2*	522159	protein-coding	0.52082142	3.67239929	21.4007112	5.27E-05	0.00255022
ENSBTAG00000046162	*ZNF385C*	100298918	protein-coding	-2.2617723	0.49763085	21.3780734	5.31E-05	0.0025599
ENSBTAG00000020393	*METTL21C*	519447	protein-coding	1.81713145	2.83448417	21.3180651	5.41E-05	0.00259164
ENSBTAG00000011397	*UNC13B*	530692	protein-coding	0.5327443	4.47733664	21.1559507	5.70E-05	0.00269803
ENSBTAG00000002028	*DIPK1A*	541171	protein-coding	0.65868012	3.71539476	21.1556097	5.70E-05	0.00269803
ENSBTAG00000047420	*BTNL9*	100125313	protein-coding	-0.9959889	5.22809644	21.1500393	5.71E-05	0.00269803
ENSBTAG00000001244	*PLAT*	281407	protein-coding	0.72719207	4.58343806	21.0727121	5.86E-05	0.00274732
ENSBTAG00000024509	*TMEM52*	617403	protein-coding	1.0706995	2.18952828	20.9822458	6.03E-05	0.00278982
ENSBTAG00000049058	*INHBB*	530430	protein-coding	1.20184652	1.90727396	20.9811838	6.03E-05	0.00278982
ENSBTAG00000012441	*SMAD7*	535916	protein-coding	1.14994834	3.04231853	20.978893	6.03E-05	0.00278982
ENSBTAG00000003985	*CCDC15*	512305	protein-coding	0.70842998	3.7772687	20.9689715	6.05E-05	0.00278982
ENSBTAG00000044022	*KIAA1671*	533883	protein-coding	-1.0405471	3.98969782	20.9632799	6.06E-05	0.00278982
ENSBTAG00000046121	*LOC100196897*	100196897	protein-coding	1.47660668	2.59006198	20.8893066	6.21E-05	0.00283848
ENSBTAG00000015384	*PHF13*	540889	protein-coding	0.97394666	1.08716982	20.8785601	6.23E-05	0.0028391
ENSBTAG00000021538	*MYO1E*	523485	protein-coding	0.81579537	3.97209178	20.819657	6.35E-05	0.00287393

ENSBTAG00000016354	*IER2*	525380	protein-coding	0.87930119	3.77668803	20.810768	6.37E-05	0.00287393
ENSBTAG00000012194	*DENND2C*	522279	protein-coding	1.05039186	1.79085761	20.7442575	6.51E-05	0.00292691
ENSBTAG00000010389	*STBD1*	513376	protein-coding	1.14591448	6.7890088	20.7008731	6.60E-05	0.00295878
ENSBTAG00000019090	*PLEKHH3*	541100	protein-coding	1.32422433	3.91335589	20.6739641	6.66E-05	0.00297516
ENSBTAG00000050914	*CYYR1*	768230	protein-coding	-0.5163914	5.48795179	20.5816983	6.86E-05	0.00302694
ENSBTAG00000031824	*RBM19*	507065	protein-coding	-0.5038114	3.15054617	20.5263803	6.98E-05	0.00307209
ENSBTAG00000019843	*HAUS6*	538833	protein-coding	-0.604353	3.88758457	20.4960681	7.05E-05	0.0030928
ENSBTAG00000013616	*AP4E1*	536144	protein-coding	-0.5001321	4.53852549	20.3826266	7.32E-05	0.00319367
ENSBTAG00000015938	*ARAP3*	112447442	protein-coding	0.50093656	5.44387469	20.35397	7.39E-05	0.00319507
ENSBTAG00000009976				-0.5332932	4.44082918	20.3509617	7.39E-05	0.00319507
ENSBTAG00000001133	*VWA8*	524159	protein-coding	-0.6773705	4.56655003	20.348458	7.40E-05	0.00319507
ENSBTAG00000010843	*PGRMC2*	534021	protein-coding	0.5588735	6.16404876	20.3026392	7.51E-05	0.00321773
ENSBTAG00000046814	*ACOT2*	511033	protein-coding	-1.1748048	2.129367	20.2990912	7.52E-05	0.00321773
ENSBTAG00000008131	*RNF115*	614061	protein-coding	-0.7749904	4.78604328	20.2704755	7.59E-05	0.00323758
ENSBTAG00000015060	*GBP5*	516949	protein-coding	1.10946496	3.39888806	20.2448054	7.65E-05	0.00325494
ENSBTAG00000016229	*KLF9*	539139	protein-coding	-0.8305906	5.81364668	20.2327844	7.68E-05	0.00325789
ENSBTAG00000027696	*TTC30B*	538713	protein-coding	-1.390292	0.17444364	20.1660119	7.85E-05	0.00330386
ENSBTAG00000001773				-0.7204334	3.73216048	20.1623414	7.86E-05	0.00330386
ENSBTAG00000030348	*ZNF582*	790311	protein-coding	-0.9053058	1.61410873	20.1165311	7.98E-05	0.00334375
ENSBTAG00000015251	*ALG13*	515282	protein-coding	0.87975108	2.92272785	20.0946448	8.04E-05	0.00335777
ENSBTAG00000025337	*MYH13*	511687	protein-coding	-0.6751467	6.96722339	20.0856081	8.06E-05	0.00335777
ENSBTAG00000014472	*LCMT2*	538825	protein-coding	-1.5178502	0.17022578	20.0545906	8.15E-05	0.00338204
ENSBTAG00000039695	*PPP1R18*	539829	protein-coding	0.5536275	3.6156489	20.0190584	8.24E-05	0.00339325
ENSBTAG00000011808	*MSTN*	281187	protein-coding	1.97802362	3.39475405	20.017608	8.24E-05	0.00339325
ENSBTAG00000018214	*SHISA2*	617336	protein-coding	1.30660345	5.82186987	19.9252818	8.50E-05	0.0034672
ENSBTAG00000017680	*NOS3*	287024	protein-coding	0.99797829	4.76799538	19.8904814	8.60E-05	0.00349694
ENSBTAG00000053238	*KALRN*	540675	protein-coding	-0.7494676	3.41858537	19.8260874	8.78E-05	0.00353496
ENSBTAG00000015424	*WDR12*	528209	protein-coding	-0.5684612	3.15501018	19.8220616	8.79E-05	0.00353496
ENSBTAG00000053836	*CCDC71L*	112446372	protein-coding	-1.6638059	0.6758319	19.8011713	8.85E-05	0.00353496
ENSBTAG00000021090	*ABCC1*	281588	protein-coding	-0.7153737	5.70063419	19.7950101	8.87E-05	0.00353496
ENSBTAG00000004895	*AFAP1L2*	615436	protein-coding	0.56966185	3.69092831	19.7888022	8.89E-05	0.00353496
ENSBTAG00000008832	*CCL1*	786156	protein-coding	1.17496337	2.61890427	19.725373	9.08E-05	0.00358857
ENSBTAG00000009210	*ZBTB44*	540532	protein-coding	-0.5445238	4.49970722	19.665361	9.26E-05	0.0036399
ENSBTAG00000000332	*ERF*	789798	protein-coding	0.83126874	3.31259869	19.6333515	9.36E-05	0.00366834
ENSBTAG00000003822	*CROCC*	530641	protein-coding	0.56897502	3.75775442	19.558494	9.59E-05	0.00374661
ENSBTAG00000043567				0.7221252	9.69991559	19.5527148	9.61E-05	0.00374661
ENSBTAG00000000423	*RBM4B*	505756	protein-coding	0.50906667	3.31178632	19.4675249	9.88E-05	0.00384051
ENSBTAG00000039283				-2.2564853	-0.1393763	19.3734385	0.00010197	0.00392241
ENSBTAG00000008652	*STYXL2*	100124500	protein-coding	-0.5783386	7.8890971	19.3314996	0.0001034	0.0039552
ENSBTAG00000019967	*RASA3*	282532	protein-coding	0.57678541	4.48727442	19.301645	0.00010444	0.00397308
ENSBTAG00000054250				0.78066346	2.68683351	19.2924413	0.00010476	0.00397452
ENSBTAG00000000731	*WFIKKN2*	531979	protein-coding	0.73998573	4.30286228	19.2054013	0.00010784	0.00404779
ENSBTAG00000009145	*SLC7A4*	504923	protein-coding	1.03264304	2.5527491	19.1944987	0.00010823	0.00405173

ENSBTAG00000003165	*ADAMTS9*	537051	protein-coding	0.81186334	4.86348459	19.1801723	0.00010875	0.00405802
ENSBTAG00000011358	*IER3*	505455	protein-coding	1.03215939	3.84879201	19.1409097	0.00011018	0.0040814
ENSBTAG00000000507	*NR4A1*	528390	protein-coding	1.35061788	7.39380534	19.1264086	0.00011072	0.00409045
ENSBTAG00000017266	*ITGA6*	535043	protein-coding	0.68531926	6.37037262	19.0815132	0.00011239	0.00410707
ENSBTAG00000007732	*ARPP21*	618648	protein-coding	-0.6288124	5.59048772	19.0649471	0.00011301	0.00410707
ENSBTAG00000003015	*SESN1*	538226	protein-coding	-0.9220632	5.9984065	19.0647143	0.00011302	0.00410707
ENSBTAG00000019052	*ANK3*	511203	protein-coding	0.68096708	7.03864575	19.0636911	0.00011306	0.00410707
ENSBTAG00000027425	*CCDC190*	617478	protein-coding	1.35827044	2.14871309	19.0546501	0.0001134	0.00410707
ENSBTAG00000009451	*JDP2*	531476	protein-coding	0.68181378	2.58513589	19.0444168	0.00011379	0.00410707
ENSBTAG00000006542	*RABEP2*	511736	protein-coding	1.32804322	4.55254435	18.9509131	0.00011741	0.00422686
ENSBTAG00000031548	*LOC514257*	514257	protein-coding	-0.7651879	5.62425071	18.9260496	0.0001184	0.004239
ENSBTAG00000030247	*NSMF*	615854	protein-coding	0.67471267	3.7767503	18.9195624	0.00011865	0.004239
ENSBTAG00000009788	*ACOT2*	785383	protein-coding	-1.3697119	0.22037739	18.9090174	0.00011907	0.00424325
ENSBTAG00000015821	*AMPD3*	519803	protein-coding	-1.7633225	3.01225221	18.8296714	0.00012229	0.00433586
ENSBTAG00000011969	*HSPB1*	516099	protein-coding	-0.918784	8.80952526	18.7211682	0.00012684	0.00446447
ENSBTAG00000019409	*GNPTAB*	509610	protein-coding	-0.7588462	4.8256365	18.7169677	0.00012701	0.00446447
ENSBTAG00000012674	*CNKSR3*	506781	protein-coding	0.72438707	2.91396797	18.7129943	0.00012718	0.00446447
ENSBTAG00000005470	*BIN3*	507115	protein-coding	-0.700386	2.20210316	18.6635615	0.00012932	0.00450577
ENSBTAG00000001835	*GJA1*	281193	protein-coding	0.74051967	2.72424799	18.5967302	0.00013227	0.0045491
ENSBTAG00000053806	*LOC618737*	618737	protein-coding	1.31849693	2.16392991	18.5943716	0.00013238	0.0045491
ENSBTAG00000000671	*PARP3*	507426	protein-coding	1.67813726	2.06730287	18.5867281	0.00013272	0.0045491
ENSBTAG00000020073	*NCEH1*	534212	protein-coding	-0.8042928	3.06442967	18.56436	0.00013373	0.00455768
ENSBTAG00000004585				0.60778059	3.11434686	18.5411734	0.00013478	0.00457432
ENSBTAG00000049022	*MIR12048*	114483693	ncRNA	2.7023475	-0.7411765	18.5255696	0.00013549	0.00457432
ENSBTAG00000017310	*EVA1C*	516536	protein-coding	1.12016068	0.54801375	18.5217717	0.00013566	0.00457432
ENSBTAG00000015988	*MYH11*	530050	protein-coding	-1.1076493	5.20740978	18.5195818	0.00013577	0.00457432
ENSBTAG00000015751	*MEOX1*	529059	protein-coding	0.97698143	2.30875201	18.5091314	0.00013625	0.00457432
ENSBTAG00000047502	*FKBP5*	535704	protein-coding	-1.4513221	7.45698684	18.5081293	0.00013629	0.00457432
ENSBTAG00000009199	*GLIS2*	527935	protein-coding	0.8001422	3.14673212	18.5038815	0.00013649	0.00457432
ENSBTAG00000007246	*TMEM204*	615464	protein-coding	0.65241213	3.83722251	18.4936576	0.00013696	0.00457927
ENSBTAG00000039861	*OAS1Y*	654488	protein-coding	1.3876696	4.55054756	18.4563805	0.0001387	0.0046155
ENSBTAG00000003808	*HDAC9*	535415	protein-coding	1.23413752	3.02290874	18.422553	0.0001403	0.0046577
ENSBTAG00000014818	*MYLK3*	526379	protein-coding	1.42131795	3.59631271	18.3909832	0.00014181	0.00469675
ENSBTAG00000047155	*C28H10orf71*	540418	protein-coding	-0.6978139	7.8478661	18.3762081	0.00014252	0.00470927
ENSBTAG00000006081	*PPM1J*	100848925	protein-coding	0.66657818	4.93492318	18.2604425	0.00014824	0.00484134
ENSBTAG00000020414	*DHX37*	529207	protein-coding	-0.5904195	3.29172193	18.241629	0.00014919	0.00486115
ENSBTAG00000013869	*SH3BP4*	520462	protein-coding	-0.6453565	4.11701964	18.1946255	0.0001516	0.0049168
ENSBTAG00000002878	*CHRD*	509942	protein-coding	0.77538425	2.79173775	18.1250939	0.00015523	0.00499972
ENSBTAG00000021661	*APAF1*	537782	protein-coding	-0.6668897	4.15350952	18.1238097	0.0001553	0.00499972
ENSBTAG00000006253	*FLNC*	528415	protein-coding	-0.9981815	11.8151321	18.1186533	0.00015558	0.00499972
ENSBTAG00000031715	*PCMTD2*	505807	protein-coding	-0.5626602	5.76961703	18.1039291	0.00015636	0.00501346
ENSBTAG00000025424	*NOTCH4*	536128	protein-coding	0.59974216	5.14049173	18.0372866	0.00015996	0.00510562
ENSBTAG00000016746	*UBE2C*	506962	protein-coding	0.98057339	1.552143	17.9694195	0.00016372	0.00520193

ENSBTAG00000047088				-0.7795371	2.30738847	17.9017049	0.00016756	0.00529057
ENSBTAG00000016782	*SLC39A10*	521004	protein-coding	-0.5691491	4.59792792	17.9003942	0.00016763	0.00529057
ENSBTAG00000001816	*SLC25A42*	504608	protein-coding	-0.6486059	2.99050858	17.838717	0.00017122	0.0053916
ENSBTAG00000027766	*C1QTNF5*	614671	protein-coding	0.96446415	2.24181727	17.8202709	0.0001723	0.00541375
ENSBTAG00000007554	*IFI6*	512913	protein-coding	1.67463523	3.80831786	17.8133507	0.00017271	0.00541454
ENSBTAG00000020584	*IFT81*	519602	protein-coding	0.7785144	2.31630783	17.8065645	0.00017312	0.00541512
ENSBTAG00000006907	*NEB*	407121	protein-coding	-0.5884613	13.9571834	17.7850607	0.0001744	0.00543113
ENSBTAG00000020407	*MTSS1*	523498	protein-coding	0.50220347	5.35957701	17.6914879	0.00018011	0.00557183
ENSBTAG00000015702	*PITX3*	512000	protein-coding	-1.3082987	2.90333734	17.6455075	0.00018298	0.00563603
ENSBTAG00000008564	*LOC511161*	511161	protein-coding	-2.0110144	1.11506481	17.6048686	0.00018557	0.00569322
ENSBTAG00000004112	*GAMT*	515270	protein-coding	0.53313951	6.34658229	17.5928369	0.00018634	0.00569322
ENSBTAG00000039601	*SAP25*	100296776	protein-coding	0.89897865	1.65605658	17.5778092	0.00018731	0.00570676
ENSBTAG00000008817	*LAMA4*	529670	protein-coding	0.67267518	5.92244528	17.5613854	0.00018837	0.00572682
ENSBTAG00000016033	*HOXD9*	100295338	protein-coding	-0.5494684	3.70957492	17.5276414	0.00019058	0.00578148
ENSBTAG00000005946	*USP13*	100141289	protein-coding	-0.6819269	6.85134615	17.5035563	0.00019217	0.00581728
ENSBTAG00000003219				0.81646711	2.37788893	17.4882013	0.0001932	0.00582532
ENSBTAG00000012896	*METTL7B*	506952	protein-coding	-1.1764746	1.17649973	17.4871531	0.00019327	0.00582532
ENSBTAG00000030521	*IER5L*	782019	protein-coding	0.9433945	1.4965729	17.4662125	0.00019467	0.00584266
ENSBTAG00000009292	*TMEM100*	613987	protein-coding	0.98868483	2.78727508	17.4333316	0.0001969	0.00587422
ENSBTAG00000014707	*ISG15*	281871	protein-coding	1.70166995	3.169749	17.4322358	0.00019698	0.00587422
ENSBTAG00000020125	*RBFOX2*	514951	protein-coding	0.55479457	6.10246605	17.4144015	0.0001982	0.00588988
ENSBTAG00000000625	*SMAD6*	511279	protein-coding	1.05440287	1.26366651	17.4123452	0.00019834	0.00588988
ENSBTAG00000012987	*ULK1*	526310	protein-coding	-0.5840099	6.65278597	17.4007832	0.00019913	0.00589117
ENSBTAG00000046307	*CEBPD*	281678	protein-coding	-1.6032642	5.19570904	17.3995594	0.00019922	0.00589117
ENSBTAG00000014608	*KDM1B*	513667	protein-coding	-0.5891441	3.76452021	17.3846051	0.00020025	0.00590937
ENSBTAG00000018520	*SCN1A*	529590	protein-coding	2.27981187	1.78598967	17.3763756	0.00020083	0.00591065
ENSBTAG00000000564	*NSG2*	613901	protein-coding	1.15841783	1.95603382	17.3719153	0.00020114	0.00591065
ENSBTAG00000016169	*ID1*	497011	protein-coding	0.97871565	4.80761423	17.3487762	0.00020276	0.00594587
ENSBTAG00000020854	*BCL6B*	100138731	protein-coding	0.79892599	3.91905603	17.3324376	0.00020391	0.00596725
ENSBTAG00000009055	*RNF144B*	524166	protein-coding	-0.6696627	6.78982828	17.2662537	0.00020865	0.00608073
ENSBTAG00000008096	*EDN1*	281137	protein-coding	-1.3631616	0.57120187	17.242844	0.00021036	0.00609204
ENSBTAG00000010493	*SERTAD1*	539321	protein-coding	0.80071501	3.56646969	17.2406638	0.00021052	0.00609204
ENSBTAG00000013131	*FAM110D*	787384	protein-coding	0.81380531	2.0586777	17.2369631	0.00021079	0.00609204
ENSBTAG00000013078	*LOC104968411*	104968411	protein-coding	-1.1354801	3.57477774	17.1790694	0.00021508	0.00613631
ENSBTAG00000006291	*CYLD*	536421	protein-coding	-0.602527	5.15948779	17.1749481	0.00021539	0.00613631
ENSBTAG00000005413	*NLRC5*	782441	protein-coding	0.56600019	4.75533581	17.1717878	0.00021563	0.00613631
ENSBTAG00000050406				0.92848304	1.22196418	17.1694712	0.0002158	0.00613631
ENSBTAG00000009302	*RCAN2*	525636	protein-coding	-0.9157088	2.52094954	17.1486892	0.00021737	0.00613631
ENSBTAG00000033801	*VRK2*	523902	protein-coding	-0.6796309	3.17649207	17.1467949	0.00021751	0.00613631
ENSBTAG00000021911	*PTPRG*	540575	protein-coding	-0.542184	4.31611581	17.1442866	0.0002177	0.00613631
ENSBTAG00000005444	*UGGT1*	516332	protein-coding	-0.5250524	4.34224714	17.1423421	0.00021785	0.00613631
ENSBTAG00000043570				0.77451059	10.6998651	17.1350098	0.00021841	0.00613631
ENSBTAG00000006563	*KLHL40*	514526	protein-coding	-1.1083403	7.52348913	17.1191841	0.00021961	0.00615797

ENSBTAG00000003359	*ELOVL5*	617293	protein-coding	0.61469986	3.35199085	17.1057486	0.00022065	0.00617459
ENSBTAG00000014605	*ETV6*	504512	protein-coding	0.55027665	3.96599252	17.0802693	0.00022262	0.00621737
ENSBTAG00000049378				1.6281474	2.06573774	16.9645418	0.00023181	0.00642302
ENSBTAG00000053812				0.89375526	1.83515343	16.9203606	0.00023542	0.00649756
ENSBTAG00000018062	*KIAA1522*	788353	protein-coding	-1.3584573	3.169349	16.8628918	0.00024021	0.0066168
ENSBTAG00000034147	*OLFML2B*	513053	protein-coding	0.7747999	3.36032047	16.8441278	0.0002418	0.00664749
ENSBTAG00000027064	*BTBD11*	539986	protein-coding	-1.5773872	1.96439829	16.8071048	0.00024496	0.00670613
ENSBTAG00000016546	*PARP12*	513185	protein-coding	0.7508712	3.88640411	16.7451499	0.00025035	0.00681612
ENSBTAG00000000125	*SPATA5*	100297992	protein-coding	-0.6999298	2.50259028	16.7390628	0.00025089	0.00681754
ENSBTAG00000008192	*PLPPR2*	520247	protein-coding	0.80584207	4.57685553	16.7075377	0.00025369	0.0068611
ENSBTAG00000020267	*WNT9A*	511308	protein-coding	0.88477423	1.83999075	16.6791001	0.00025624	0.00689638
ENSBTAG00000018398	*GPR4*	512876	protein-coding	1.09261842	1.31887622	16.6629315	0.0002577	0.00691772
ENSBTAG00000046544	*SLC7A10*	100126273	protein-coding	2.10557384	-0.613095	16.6512435	0.00025876	0.00692467
ENSBTAG00000013227	*SNAI2*	520631	protein-coding	0.64479246	2.76947924	16.6356035	0.0002602	0.00694974
ENSBTAG00000025450	*SYNE2*	540504	protein-coding	0.55380968	7.18544583	16.6166643	0.00026194	0.00696985
ENSBTAG00000040590				0.98722295	1.66161323	16.6076023	0.00026278	0.00697897
ENSBTAG00000038141	*ADGRG1*	509703	protein-coding	0.52074705	4.97816518	16.5879407	0.00026461	0.00701365
ENSBTAG00000006954	*ZNF432*	539365	protein-coding	-0.6615482	2.05613979	16.5828823	0.00026508	0.00701365
ENSBTAG00000016908	*METTL1*	511197	protein-coding	-0.745567	1.96895207	16.5698832	0.0002663	0.00703269
ENSBTAG00000023551	*MOB3C*	614275	protein-coding	0.67702218	2.538149	16.5181055	0.00027121	0.00713573
ENSBTAG00000003152	*IFI27*	507138	protein-coding	1.38470681	4.69779431	16.4936487	0.00027357	0.00718427
ENSBTAG00000010682	*DDR1*	534092	protein-coding	0.75273996	3.83069288	16.4817093	0.00027473	0.00718786
ENSBTAG00000025400	*PARP4*	615359	protein-coding	-0.5329338	3.79952043	16.4729126	0.00027558	0.00719689
ENSBTAG00000008079	*NRBP2*	504664	protein-coding	0.55264746	3.11743051	16.4494472	0.00027788	0.00723008
ENSBTAG00000010366	*HCRTR1*	282248	protein-coding	-1.3486883	1.42769624	16.4377225	0.00027904	0.00723424
ENSBTAG00000007114	*POC5*	533555	protein-coding	-0.6741021	1.96234747	16.4374008	0.00027907	0.00723424
ENSBTAG00000037794	*MYH4*	408020	protein-coding	-0.6255469	11.4082534	16.4086338	0.00028193	0.0072949
ENSBTAG00000010111	*TCF7L1*	515303	protein-coding	0.54312611	3.27322643	16.3581827	0.00028702	0.00741294
ENSBTAG00000013530	*DDAH2*	540386	protein-coding	0.664069	3.46924992	16.3518556	0.00028766	0.00741599
ENSBTAG00000004679	*WARS1*	281576	protein-coding	0.60952214	6.92421122	16.3167529	0.00029127	0.00749286
ENSBTAG00000032292	*CCDC159*	785202	protein-coding	0.8152237	3.2834362	16.3112548	0.00029184	0.00749286
ENSBTAG00000000510	*ATG101*	513049	protein-coding	0.72420674	4.64676997	16.3073706	0.00029224	0.00749286
ENSBTAG00000020698	*MTHFR*	497032	protein-coding	0.53560979	5.25338353	16.2803612	0.00029506	0.00753765
ENSBTAG00000020626	*ERCC6L2*	508357	protein-coding	-0.562201	3.28398104	16.2617419	0.00029702	0.00756221
ENSBTAG00000011659	*PIGZ*	104968672	protein-coding	0.88952819	3.13506158	16.2610141	0.00029709	0.00756221
ENSBTAG00000014336	*WBP1L*	782651	protein-coding	-0.7566764	3.78474597	16.0241102	0.00032328	0.0081258
ENSBTAG00000009637	*SLC12A2*	286845	protein-coding	0.67452999	5.03905596	15.9647268	0.00033022	0.00821274
ENSBTAG00000051794				0.55204084	2.47074638	15.9486382	0.00033213	0.00821274
ENSBTAG00000046218	*KLF11*	616730	protein-coding	-0.9490947	6.22676254	15.944898	0.00033257	0.00821274
ENSBTAG00000018682	*SETD4*	540440	protein-coding	-0.5802064	2.7012355	15.930195	0.00033433	0.00824165
ENSBTAG00000014649	*CPT2*	504502	protein-coding	-0.5593965	4.06524667	15.8866816	0.00033958	0.00832745
ENSBTAG00000008013	*LRRC2*	541113	protein-coding	-0.6887736	6.46025742	15.8334092	0.00034614	0.00844313
ENSBTAG00000021182	*DOCK4*	534227	protein-coding	0.55216353	4.68749692	15.8288405	0.0003467	0.00844313

ENSBTAG00000008654	*HOOK2*	526516	protein-coding	0.56344738	3.79960886	15.8240723	0.0003473	0.00844313
ENSBTAG00000051298	*PVR*	526865	protein-coding	0.72224667	5.46565481	15.8064386	0.00034951	0.00845298
ENSBTAG00000016801	*RXRG*	509500	protein-coding	0.73680784	3.78627101	15.7928703	0.00035121	0.00847581
ENSBTAG00000012143	*TECPR2*	530121	protein-coding	-0.5249413	3.03223542	15.7818961	0.0003526	0.00847747
ENSBTAG00000047680	*IRF7*	100125591	protein-coding	1.24913559	2.14021719	15.7755574	0.00035341	0.00847747
ENSBTAG00000046503	*NCOA7*	541071	protein-coding	-0.6246369	2.79107035	15.7217615	0.00036031	0.00862543
ENSBTAG00000007666	*IGF2BP2*	519028	protein-coding	-0.741507	2.69332976	15.6881731	0.0003647	0.00869046
ENSBTAG00000004248	*MLYCD*	512341	protein-coding	-0.7310091	5.24311698	15.6820821	0.0003655	0.00869046
ENSBTAG00000003825	*PTPN12*	531877	protein-coding	0.50100169	4.56690443	15.6704526	0.00036703	0.00871224
ENSBTAG00000008953	*TAP1*	524959	protein-coding	0.63472859	5.24287952	15.6569856	0.00036882	0.00873302
ENSBTAG00000012216	*MLKL*	790225	protein-coding	0.9926757	1.19225542	15.6498475	0.00036977	0.00873302
ENSBTAG00000010416	*RIN3*	539951	protein-coding	0.57465653	4.0379086	15.637008	0.00037149	0.00875886
ENSBTAG00000050737				-1.9704357	0.36199981	15.5889157	0.00037799	0.00886362
ENSBTAG00000018213	*TBCEL*	540781	protein-coding	-0.5611266	2.8972829	15.5829112	0.00037881	0.00886362
ENSBTAG00000003049	*MICALL2*	510644	protein-coding	0.56716062	3.48722919	15.5516475	0.00038311	0.00889885
ENSBTAG00000051225				1.0798032	1.11617226	15.5435843	0.00038423	0.00891012
ENSBTAG00000006160	*SUOX*	509837	protein-coding	-0.627066	3.05671684	15.5292291	0.00038623	0.00894176
ENSBTAG00000021808	*CACNB3*	282160	protein-coding	0.51971158	3.79363895	15.51859	0.00038772	0.00894819
ENSBTAG00000037072	*LOC112441908*	112441908	snRNA	2.83321753	-0.6322429	15.5110189	0.00038878	0.00894819
ENSBTAG00000001510	*CDK17*	539655	protein-coding	0.50095538	3.94293606	15.5092339	0.00038903	0.00894819
ENSBTAG00000015479	*ZFPL1*	613442	protein-coding	-0.5136269	2.93655405	15.5091119	0.00038905	0.00894819
ENSBTAG00000016112	*CCDC197*	617443	protein-coding	1.27529589	2.06613673	15.4625049	0.00039567	0.00908558
ENSBTAG00000011482	*SLC43A2*	511955	protein-coding	-1.600128	6.1487181	15.42987	0.00040038	0.00915482
ENSBTAG00000014270	*UNC5B*	524942	protein-coding	0.74385159	4.10529465	15.428591	0.00040056	0.00915482
ENSBTAG00000004290	*DDX28*	539411	protein-coding	-0.5391337	2.57288303	15.4280756	0.00040064	0.00915482
ENSBTAG00000020084	*LONRF1*	504489	protein-coding	0.71439939	2.82533009	15.3870492	0.00040664	0.00923209
ENSBTAG00000033315	*DNAJC1*	781036	protein-coding	-0.5308336	3.28778936	15.3642223	0.00041003	0.0092939
ENSBTAG00000019552	*PGRMC1*	317706	protein-coding	0.59150649	4.41651097	15.3424575	0.00041328	0.0093027
ENSBTAG00000030499	*DIAPH1*	786565	protein-coding	-0.8288405	6.11678716	15.3412988	0.00041345	0.0093027
ENSBTAG00000001567	*LRRK1*	513994	protein-coding	0.73719036	3.32303653	15.3395319	0.00041372	0.0093027
ENSBTAG00000008132	*SOX13*	788459	protein-coding	0.63429011	3.32140482	15.3200393	0.00041666	0.00935387
ENSBTAG00000010943	*SLC22A23*	614701	protein-coding	-0.6895848	1.88023617	15.305369	0.00041889	0.00935909
ENSBTAG00000038048	*MRC1*	787578	protein-coding	1.40892834	2.74002993	15.2895953	0.00042129	0.009398
ENSBTAG00000046862	*MARCKSL1*	539555	protein-coding	0.79328023	2.25932219	15.266486	0.00042485	0.00944103
ENSBTAG00000021508	*LMOD3*	509978	protein-coding	0.68802982	8.16729715	15.2640001	0.00042524	0.00944103
ENSBTAG00000010760	*REV3L*	517422	protein-coding	-0.6863795	6.76598349	15.2565209	0.00042639	0.00945185
ENSBTAG00000012262	*FBXO10*	781854	protein-coding	-0.9254864	1.76832675	15.2338125	0.00042993	0.0095062
ENSBTAG00000007390	*VAT1*	510698	protein-coding	0.53582117	4.38337851	15.2033605	0.00043473	0.00957628
ENSBTAG00000027320	*KCNB1*	539528	protein-coding	-0.9325967	3.89286834	15.1895529	0.00043692	0.00959456
ENSBTAG00000016377	*IPP*	785293	protein-coding	0.59205623	4.12038361	15.0970578	0.00045192	0.00986238
ENSBTAG00000020219	*MSS51*	508577	protein-coding	0.96214157	7.80728409	15.0834637	0.00045417	0.00988085
ENSBTAG00000006846	*LGALS9*	510813	protein-coding	0.70319495	4.79185545	15.0733809	0.00045585	0.00989525
ENSBTAG00000050535	*ZNF385D*	789528	protein-coding	-1.1351157	3.26500537	15.071034	0.00045624	0.00989525

ENSBTAG00000012106	*ADCK1*	533372	protein-coding	-0.6845966	1.53646317	15.0507355	0.00045964	0.00993833
ENSBTAG00000045789	*TNNT2*	286816	protein-coding	0.88542632	1.56139266	15.0276048	0.00046354	0.00998409
ENSBTAG00000007415	*SLC7A8*	536818	protein-coding	-1.2340511	6.46668273	15.0256095	0.00046388	0.00998409
ENSBTAG00000031476	*DLL1*	788775	protein-coding	0.54257146	3.59346851	14.9667982	0.00047398	0.01013942
ENSBTAG00000010652	*PDE4C*	524624	protein-coding	0.70060817	5.66632179	14.9429432	0.00047814	0.01021294
ENSBTAG00000009493	*BCL3*	531942	protein-coding	0.61202396	2.84844112	14.9198304	0.00048221	0.0102532
ENSBTAG00000053762				1.92656897	-0.7390427	14.911643	0.00048366	0.01025576
ENSBTAG00000003836	*ADAM19*	510907	protein-coding	-0.61901	4.24696451	14.8851735	0.00048838	0.01032208
ENSBTAG00000027694	*TTC30A*	510160	protein-coding	-0.8409003	1.26288876	14.8446218	0.00049571	0.01046128
ENSBTAG00000017514	*CCND1*	524530	protein-coding	1.24805491	1.36815991	14.835155	0.00049744	0.01046684
ENSBTAG00000031135	*CTXN3*	100125915	protein-coding	1.20427388	3.61735092	14.8350292	0.00049746	0.01046684
ENSBTAG00000013556	*UNC13D*	506146	protein-coding	0.94885039	1.68277576	14.820275	0.00050017	0.01050806
ENSBTAG00000002615	*LONRF3*	508189	protein-coding	0.88367739	3.20913706	14.8108601	0.00050191	0.0105288
ENSBTAG00000011257	*ASAH1*	510620	protein-coding	0.50167209	3.67634782	14.8052404	0.00050294	0.01053488
ENSBTAG00000001814	*PLXND1*	781625	protein-coding	0.71103069	5.70506784	14.7909209	0.0005056	0.01057479
ENSBTAG00000010348	*GATD1*	513454	protein-coding	0.50206901	3.51564226	14.7779283	0.00050802	0.01060972
ENSBTAG00000039740	*LY6G5B*	539236	protein-coding	0.6157189	2.38949006	14.7223417	0.00051853	0.0107812
ENSBTAG00000010361	*DLL4*	518746	protein-coding	0.84027057	3.94863489	14.666396	0.00052934	0.01095742
ENSBTAG00000020130	*CLCN3*	522719	protein-coding	-0.5431836	4.27540639	14.6421513	0.0005341	0.0110397
ENSBTAG00000040361	*LFNG*	516209	protein-coding	0.74792369	3.41263755	14.6193695	0.00053862	0.01108417
ENSBTAG00000016496	*ZNF641*	538714	protein-coding	-0.785232	2.11264936	14.5791163	0.00054669	0.01123392
ENSBTAG00000007334	*NFKBID*	614198	protein-coding	0.53958696	2.40503132	14.5747075	0.00054758	0.01123555
ENSBTAG00000032933	*TTC23L*	783234	protein-coding	1.97481717	-0.1440398	14.5682312	0.0005489	0.01123555
ENSBTAG00000012636	*IKZF4*	540671	protein-coding	-0.9303004	1.41357308	14.5460601	0.00055342	0.01130614
ENSBTAG00000010455	*INTS5*	514150	protein-coding	-0.5308665	3.11271223	14.5413536	0.00055438	0.01130944
ENSBTAG00000037533	*C4A*	280678	protein-coding	0.87323178	3.49603122	14.5224423	0.00055828	0.01136356
ENSBTAG00000045593	*EARS2*	100300732	protein-coding	-0.8830649	0.69477218	14.5206461	0.00055865	0.01136356
ENSBTAG00000008240	*GALNT15*	618078	protein-coding	-1.270443	0.61817506	14.5045966	0.00056198	0.01139838
ENSBTAG00000015115	*ACP4*	788015	protein-coding	0.58365115	2.2920015	14.4948433	0.00056402	0.01140694
ENSBTAG00000020713	*BACH2*	538296	protein-coding	-1.353692	0.92906432	14.4948051	0.00056403	0.01140694
ENSBTAG00000007704	*ELOVL7*	614096	protein-coding	0.91443482	1.72423526	14.4711589	0.00056899	0.01149086
ENSBTAG00000001864	*NR4A3*	528877	protein-coding	3.4457912	5.28162809	14.4466433	0.00057419	0.01157922
ENSBTAG00000009407	*MBTD1*	511415	protein-coding	-0.5270185	3.35660529	14.4236336	0.00057911	0.01164516
ENSBTAG00000013317	*RNF168*	613812	protein-coding	-0.6643922	4.07449386	14.4179141	0.00058035	0.01165327
ENSBTAG00000012966	*SCRN3*	511983	protein-coding	-0.5727412	3.31120247	14.4040875	0.00058333	0.01167692
ENSBTAG00000049641	*ENOX2*	100848511	protein-coding	0.96876133	3.38608241	14.4009564	0.00058401	0.01167692
ENSBTAG00000024826	*TECRL*	616643	protein-coding	0.65119611	3.2244313	14.3637491	0.00059214	0.01178546
ENSBTAG00000005604	*POPDC2*	540535	protein-coding	0.60575133	3.58952985	14.3561008	0.00059383	0.01178546
ENSBTAG00000014885	*MYOM3*	532872	protein-coding	-0.8134698	7.59871706	14.3556393	0.00059393	0.01178546
ENSBTAG00000013391	*ANKH*	511800	protein-coding	0.67871745	4.37102308	14.3485748	0.00059549	0.01178546
ENSBTAG00000021791	*PARP9*	510532	protein-coding	0.67622455	3.33799024	14.3456642	0.00059614	0.01178546
ENSBTAG00000046724	*CBX6*	513830	protein-coding	-0.5995126	2.2403054	14.3403572	0.00059732	0.01179219
ENSBTAG00000001938	*CKS2*	767985	protein-coding	-0.9471078	1.80683198	14.3358705	0.00059831	0.01179535

ENSBTAG00000020166	*ZNFX1*	539807	protein-coding	0.79767922	5.54559903	14.3251018	0.00060072	0.01182614
ENSBTAG00000050196				-2.7441997	-0.3189963	14.3118213	0.00060369	0.01185159
ENSBTAG00000022580	*INKA2*	616754	protein-coding	0.77540014	2.10574074	14.2551661	0.00061657	0.01205395
ENSBTAG00000001274	*PPM1L*	541235	protein-coding	-0.609583	4.9351489	14.2407473	0.00061989	0.01207588
ENSBTAG00000015402	*GREB1*	508204	protein-coding	-1.36044	1.72010374	14.2391384	0.00062027	0.01207588
ENSBTAG00000032092	*CDH11*	785475	protein-coding	0.57692488	2.81054066	14.2230251	0.000624	0.01211516
ENSBTAG00000002922	*GAB2*	529346	protein-coding	-0.5062326	4.88507347	14.2157778	0.00062569	0.01213123
ENSBTAG00000021582	*NCAPG*	531234	protein-coding	0.87240521	1.50348325	14.1863694	0.0006326	0.01224827
ENSBTAG00000000080	*GRWD1*	540739	protein-coding	-0.520365	3.63268438	14.1694128	0.00063662	0.01230915
ENSBTAG00000014358	*EVA1B*	618094	protein-coding	0.69445852	2.30882917	14.1628208	0.00063819	0.01232257
ENSBTAG00000021829	*GM2A*	504524	protein-coding	1.20954242	1.20218432	14.1522237	0.00064072	0.01234821
ENSBTAG00000032301	*SLC26A7*	617196	protein-coding	0.90356524	1.66705973	14.1499268	0.00064127	0.01234821
ENSBTAG00000015405	*DCHS1*	515576	protein-coding	0.58437715	4.70760952	14.1422983	0.0006431	0.01236655
ENSBTAG00000004995	*C1GALT1*	539417	protein-coding	-0.5098319	2.82431655	14.129891	0.00064609	0.0123902
ENSBTAG00000024091	*MALL*	512289	protein-coding	0.75615921	2.81468492	14.1218817	0.00064803	0.01239426
ENSBTAG00000003366	*DDX58*	504760	protein-coding	0.7840645	3.76685446	14.1069662	0.00065166	0.01244603
ENSBTAG00000012035	*EXOC3L1*	526306	protein-coding	1.27604102	1.47108358	14.0966016	0.00065419	0.01247746
ENSBTAG00000030425	*ID3*	538690	protein-coding	0.57671351	4.79100566	14.0407497	0.00066802	0.01264635
ENSBTAG00000007890	*SEC14L5*	790103	protein-coding	1.15192443	1.25480478	14.0390788	0.00066843	0.01264635
ENSBTAG00000031998	*CXCL16*	511671	protein-coding	0.64552941	2.84426125	14.0287557	0.00067103	0.01264906
ENSBTAG00000002669	*RASSF4*	510549	protein-coding	0.74797321	2.70492613	13.992266	0.00068027	0.01278061
ENSBTAG00000004165				0.5001196	4.00840941	13.9894961	0.00068098	0.01278061
ENSBTAG00000015008	*CPA3*	528454	protein-coding	1.86001415	0.02468314	13.9791377	0.00068363	0.01281294
ENSBTAG00000046122	*NRARP*	100299743	protein-coding	1.10991952	0.73020767	13.9645064	0.0006874	0.01282385
ENSBTAG00000007374	*LHFPL2*	616131	protein-coding	0.67771765	3.01676629	13.9522298	0.00069057	0.01284081
ENSBTAG00000006732	*NRCAM*	534500	protein-coding	-2.2706336	-0.8017306	13.9379168	0.00069429	0.01286666
ENSBTAG00000002902	*ANO6*	521784	protein-coding	-0.5559193	5.70308477	13.8672011	0.000713	0.01310215
ENSBTAG00000005622	*LITAF*	520564	protein-coding	0.59076686	3.84343843	13.853152	0.00071679	0.01314788
ENSBTAG00000014286	*IFT88*	514177	protein-coding	-0.6980049	2.23439684	13.8510197	0.00071736	0.01314788
ENSBTAG00000011146	*RAB8B*	614242	protein-coding	0.61059795	4.09006882	13.8184425	0.00072622	0.0132693
ENSBTAG00000015290	*BAIAP2L2*	617924	protein-coding	1.27477411	-0.106606	13.8123553	0.00072788	0.0132693
ENSBTAG00000015200	*L2HGDH*	514230	protein-coding	0.52940346	4.30358056	13.8093893	0.0007287	0.0132693
ENSBTAG00000050809	*GIPC3*	618265	protein-coding	1.2175041	-0.0205345	13.8043368	0.00073009	0.01327742
ENSBTAG00000007881	*LOC100139670*	100139670	protein-coding	1.33568677	3.38914507	13.7919979	0.00073349	0.01332211
ENSBTAG00000030913	*MX1*	280872	protein-coding	1.42163977	5.19128531	13.7718824	0.00073908	0.01340627
ENSBTAG00000019412				-0.5473968	2.46129951	13.7638275	0.00074133	0.01341659
ENSBTAG00000000434	*CRYAB*	281719	protein-coding	-0.8620074	9.86607098	13.7630201	0.00074155	0.01341659
ENSBTAG00000000727	*RNF169*	525756	protein-coding	-0.5780223	2.46728535	13.7503386	0.00074511	0.0134321
ENSBTAG00000003212	*NNAT*	353114	protein-coding	0.87852759	2.79808946	13.7497822	0.00074527	0.0134321
ENSBTAG00000018810	*THBS2*	338092	protein-coding	-0.8823973	2.78373409	13.7422753	0.00074738	0.01343242
ENSBTAG00000011345	*SFMBT1*	511984	protein-coding	-0.7541656	3.0554576	13.7343148	0.00074963	0.01343242
ENSBTAG00000005198	*FGF1*	281160	protein-coding	-1.2115504	3.92153075	13.7336591	0.00074982	0.01343242
ENSBTAG00000016332	*ING3*	513000	protein-coding	-0.5061511	3.04611364	13.7020213	0.00075883	0.01356621

ENSBTAG00000017086	*GRB10*	407210	protein-coding	0.69272228	6.31152936	13.6998313	0.00075946	0.01356621
ENSBTAG00000019175	*SOBP*	537433	protein-coding	0.59453001	2.82825597	13.6856829	0.00076353	0.01361675
ENSBTAG00000044160	*MCCC2*	783065	protein-coding	-0.6354122	2.70300257	13.6738459	0.00076696	0.01363094
ENSBTAG00000005034	*CAPN5*	536988	protein-coding	0.64064282	3.76435378	13.6273984	0.00078056	0.01381808
ENSBTAG00000008552				0.5750931	2.09737144	13.6244966	0.00078141	0.01381808
ENSBTAG00000006784	*PLA2G4B*	505485	protein-coding	0.65208694	2.62231481	13.608483	0.00078617	0.0138673
ENSBTAG00000049742				1.51871949	-0.0148054	13.596985	0.0007896	0.01390166
ENSBTAG00000051609	*PLPPR3*	100337214	protein-coding	1.11151314	0.891031	13.5953429	0.00079009	0.01390166
ENSBTAG00000002640	*KALRN*	540675	protein-coding	-0.905888	1.74350017	13.5816212	0.00079421	0.01395669
ENSBTAG00000048459	*CARHSP1*	506760	protein-coding	0.697032	3.1222739	13.565928	0.00079895	0.01402247
ENSBTAG00000021773	*ERCC4*	516371	protein-coding	-0.5017006	3.26041107	13.5609623	0.00080046	0.01403141
ENSBTAG00000016752	*ASB10*	616286	protein-coding	0.53154074	3.90349079	13.5467737	0.00080478	0.01408958
ENSBTAG00000002755	*ANKRD12*	534739	protein-coding	0.50849404	6.58602628	13.5375507	0.0008076	0.01411369
ENSBTAG00000012251	*MTR*	280869	protein-coding	-0.5230081	4.20727826	13.5357198	0.00080816	0.01411369
ENSBTAG00000016533	*FOXP1*	515903	protein-coding	0.66074906	4.34933802	13.4799394	0.00082546	0.01427057
ENSBTAG00000046104				0.66646795	2.12060396	13.4728543	0.00082768	0.01427057
ENSBTAG00000003946	*NECAB3*	514695	protein-coding	0.93992171	1.96430622	13.4725093	0.00082779	0.01427057
ENSBTAG00000019146	*OSBP2*	510311	protein-coding	-1.1571001	1.73808192	13.4687693	0.00082897	0.01427057
ENSBTAG00000038755				-1.0088906	0.16932544	13.4646311	0.00083028	0.01427057
ENSBTAG00000015635	*TNFRSF4*	528782	protein-coding	0.79356641	1.01434578	13.4617805	0.00083118	0.01427057
ENSBTAG00000020737	*SOX8*	530317	protein-coding	0.79300392	2.07290476	13.4506516	0.0008347	0.01429349
ENSBTAG00000051586	*ECE2*	281134	protein-coding	-0.7410282	1.48493962	13.4403217	0.00083799	0.01433232
ENSBTAG00000047706	*ING2*	404141	protein-coding	0.63958079	2.72132722	13.4341544	0.00083996	0.01434756
ENSBTAG00000039335	*ARRDC2*	523285	protein-coding	-1.231785	6.12542447	13.431148	0.00084092	0.01434756
ENSBTAG00000016559	*NUDT22*	533578	protein-coding	0.50542552	2.96416583	13.4197811	0.00084456	0.01439231
ENSBTAG00000003191	*FSCN1*	507342	protein-coding	0.71366747	4.74108412	13.3663593	0.00086192	0.01460158
ENSBTAG00000020923	*GDF1*	104968455	protein-coding	0.66939129	2.19690066	13.3506765	0.00086709	0.01463445
ENSBTAG00000016155	*GDPD3*	767913	protein-coding	0.73771156	1.35367648	13.2909611	0.00088707	0.01484709
ENSBTAG00000020359	*PLEKHM1*	523424	protein-coding	-0.5288477	4.29670662	13.2647552	0.00089599	0.01493476
ENSBTAG00000006743	*RBBP8*	512977	protein-coding	-0.5542996	3.3796788	13.263125	0.00089655	0.01493476
ENSBTAG00000021899	*PDE9A*	511665	protein-coding	-0.5468443	2.12869839	13.2505129	0.00090088	0.01495382
ENSBTAG00000017697	*CTF1*	514803	protein-coding	0.57793369	3.21977452	13.2406205	0.0009043	0.0149928
ENSBTAG00000006069	*CAPS*	616482	protein-coding	0.6640603	1.10759411	13.1117883	0.00095003	0.01560398
ENSBTAG00000021298	*NTMT2*	521303	protein-coding	1.1134182	2.76284192	13.078374	0.00096229	0.01578041
ENSBTAG00000020536	*HERC6*	527520	protein-coding	1.00102353	4.14027269	13.0387636	0.00097704	0.01593608
ENSBTAG00000005433	*SSUH2*	787021	protein-coding	-1.2735738	0.27401203	13.0322502	0.00097949	0.01595753
ENSBTAG00000005299	*EDNRB*	281750	protein-coding	0.68888933	3.43012656	12.9862765	0.00099696	0.01620466
ENSBTAG00000009863	*BHLHE40*	506945	protein-coding	0.83623779	6.30054403	12.9698487	0.00100329	0.01628863
ENSBTAG00000013569	*CD38*	327677	protein-coding	0.93314151	1.6931239	12.9345649	0.00101701	0.01640464
ENSBTAG00000022714	*HSPB3*	616007	protein-coding	0.54544533	3.17538342	12.9335159	0.00101742	0.01640464
ENSBTAG00000016656	*PARP14*	540789	protein-coding	0.83123487	4.73850283	12.9196028	0.00102289	0.01645514
ENSBTAG00000038735	*LHB*	280839	protein-coding	0.84824326	1.76103061	12.8912607	0.00103413	0.01657912
ENSBTAG00000054239	*KIF1B*	100337161	protein-coding	-0.5409145	7.70308633	12.8796625	0.00103877	0.016613

ENSBTAG00000006941	*ATAT1*	786491	protein-coding	0.60944598	1.81288724	12.8784291	0.00103927	0.016613
ENSBTAG00000035995	*TNFAIP2*	786672	protein-coding	0.85393308	2.58083245	12.8720781	0.00104182	0.016613
ENSBTAG00000008471	*MX2*	280873	protein-coding	1.66188378	2.38059015	12.8712523	0.00104215	0.016613
ENSBTAG00000017661	*RFX2*	534475	protein-coding	-1.1877472	2.70338079	12.8647905	0.00104475	0.01661519
ENSBTAG00000015304	*ANXA9*	528055	protein-coding	-1.234827	0.35021432	12.8488209	0.00105121	0.01668192
ENSBTAG00000002688	*ATP1B1*	519758	protein-coding	-0.534137	7.00629004	12.8127478	0.00106597	0.01689699
ENSBTAG00000014171	*NAPEPLD*	541291	protein-coding	-0.5215538	3.48044802	12.8059405	0.00106877	0.01692245
ENSBTAG00000011343	*XAF1*	509740	protein-coding	0.95143948	2.62223063	12.799861	0.00107129	0.01694322
ENSBTAG00000030317	*PPP1R26*	618652	protein-coding	-0.5223876	2.4970219	12.7634809	0.00108647	0.01712562
ENSBTAG00000017228	*TP53I11*	515682	protein-coding	0.61863645	3.73684329	12.7301918	0.00110057	0.01730901
ENSBTAG00000039722	*LOC782061*	782061	protein-coding	1.06824454	0.70774198	12.7050994	0.00111132	0.01743725
ENSBTAG00000039015	*TMEM145*	513015	protein-coding	0.54512173	2.86170607	12.6756626	0.00112408	0.01756104
ENSBTAG00000021316	*COL4A3*	317711	protein-coding	1.25140148	1.37878317	12.6657594	0.0011284	0.01757014
ENSBTAG00000033685	*CCNJ*	523293	protein-coding	-0.6456353	1.72219492	12.659788	0.00113102	0.01758777
ENSBTAG00000019138	*KIF3C*	777770	protein-coding	0.59788535	2.71523774	12.6412548	0.00113919	0.0176599
ENSBTAG00000010346	*ENAM*	527075	protein-coding	-2.2588078	-0.6174627	12.6333275	0.0011427	0.01769485
ENSBTAG00000021942	*HPS6*	511792	protein-coding	-0.861635	1.06542908	12.5928582	0.00116081	0.01789652
ENSBTAG00000051933				-1.1636103	1.34720511	12.5583334	0.0011765	0.01805932
ENSBTAG00000015140	*RUNDC1*	539525	protein-coding	-0.5673674	2.83741841	12.5519222	0.00117944	0.01808471
ENSBTAG00000010736	*BBS5*	528191	protein-coding	-0.9157735	1.49807359	12.5439286	0.00118311	0.01810162
ENSBTAG00000050453	*ZNF835*	101908474	protein-coding	-0.6275995	2.3020589	12.5280685	0.00119044	0.0181836
ENSBTAG00000049158	*ZNF596*	511959	protein-coding	-0.5644583	2.44314964	12.5253429	0.0011917	0.0181836
ENSBTAG00000046256	*TMEM132C*	512602	protein-coding	1.51735891	-0.0043319	12.5239603	0.00119235	0.0181836
ENSBTAG00000016236	*SLC12A6*	521217	protein-coding	-0.5457286	3.50687468	12.4539124	0.00122535	0.0186281
ENSBTAG00000045010	*MIR2350*	100313332	ncRNA	0.78181105	1.33185144	12.4536778	0.00122546	0.0186281
ENSBTAG00000018151	*HELQ*	539372	protein-coding	-0.6171382	2.23273733	12.4411067	0.00123149	0.01869951
ENSBTAG00000018799	*GPSM1*	100139799	protein-coding	0.76932219	1.57397564	12.4137559	0.00124471	0.01883929
ENSBTAG00000008142	*IFIH1*	535490	protein-coding	0.69780565	3.45337499	12.4022562	0.00125031	0.01884269
ENSBTAG00000009336	*LXN*	781644	protein-coding	1.11544793	1.70046122	12.3924372	0.00125512	0.01885493
ENSBTAG00000003986	*CXXC5*	538485	protein-coding	0.5944792	4.98874261	12.3736989	0.00126435	0.01893325
ENSBTAG00000024633	*PIK3C2B*	537396	protein-coding	0.50671072	4.66011476	12.3558979	0.00127318	0.0189844
ENSBTAG00000008808	*CCDC17*	515836	protein-coding	0.67663811	3.29774244	12.3333345	0.00128447	0.01909151
ENSBTAG00000009476	*VEGFD*	286799	protein-coding	1.09643839	0.23403135	12.2832496	0.00130991	0.01932825
ENSBTAG00000045868				-0.7489509	1.09978183	12.2825895	0.00131025	0.01932825
ENSBTAG00000019979	*CMPK2*	784304	protein-coding	0.72434355	2.99097438	12.2813295	0.0013109	0.01932825
ENSBTAG00000014218	*DKK1*	504445	protein-coding	1.64171181	1.93860436	12.2804642	0.00131134	0.01932825
ENSBTAG00000048982				0.57280968	3.86743951	12.2745382	0.00131439	0.0193419
ENSBTAG00000049218				1.00190411	1.91725337	12.2525667	0.00132577	0.01941882
ENSBTAG00000003806	*ECM1*	524222	protein-coding	0.94973567	3.15322273	12.189613	0.00135894	0.01982219
ENSBTAG00000031441	*FXYD5*	505584	protein-coding	0.60754959	3.02110822	12.1799956	0.00136408	0.01984073
ENSBTAG00000054218	*IGFBP5*	404185	protein-coding	1.02505752	6.51071927	12.1767065	0.00136585	0.01984073
ENSBTAG00000050495	*GREM1*	539079	protein-coding	2.0697017	-0.6246929	12.1684742	0.00137027	0.01985947
ENSBTAG00000010587	*SH3BGRL*	614007	protein-coding	0.55300553	3.15536933	12.1559472	0.00137704	0.01990063

ENSBTAG00000001537	*TSKU*	529661	protein-coding	0.64993552	3.23722666	12.1389762	0.00138626	0.01996837
ENSBTAG00000015132	*RXFP2*	507053	protein-coding	1.19992795	0.82097364	12.1370803	0.0013873	0.01996837
ENSBTAG00000050620				0.60654582	1.9464876	12.1342907	0.00138882	0.01996837
ENSBTAG00000051560				1.17131024	-0.1691287	12.1292466	0.00139158	0.01998762
ENSBTAG00000044172	*LMLN*	100337404	protein-coding	-0.962236	0.64786578	12.1209921	0.00139611	0.02001182
ENSBTAG00000007186	*ARHGAP39*	532363	protein-coding	0.61316294	1.80538751	12.1153084	0.00139923	0.02003624
ENSBTAG00000000717	*PLPP2*	504545	protein-coding	1.42590252	0.24681179	12.0958086	0.00141002	0.02009234
ENSBTAG00000055286	*MACIR*	538782	protein-coding	0.62839456	1.71283293	12.0884464	0.00141411	0.02010207
ENSBTAG00000052957	*MON1B*	510379	protein-coding	-0.5591058	1.79505245	12.0321858	0.00144583	0.02043323
ENSBTAG00000017856	*TSTD1*	615465	protein-coding	0.56347811	1.46623598	12.0212846	0.00145206	0.02048018
ENSBTAG00000005275	*PKIG*	404169	protein-coding	0.571475	4.31198511	12.0012626	0.00146358	0.02060737
ENSBTAG00000039356	*LOC782061*	782061	protein-coding	1.35943838	-0.9051686	11.9953618	0.001467	0.02062884
ENSBTAG00000014465	*SERPINE1*	281375	protein-coding	1.39733322	2.86159554	11.9584317	0.00148856	0.02082802
ENSBTAG00000012082	*TNFRSF12A*	617439	protein-coding	-1.5009526	5.32991376	11.9443897	0.00149685	0.02086103
ENSBTAG00000009933	*DTX3L*	515051	protein-coding	0.6915114	3.6863916	11.9335891	0.00150326	0.02090893
ENSBTAG00000008124	*GK*	505987	protein-coding	-0.7704687	2.44542433	11.9202984	0.00151118	0.0209795
ENSBTAG00000006752	*PFKFB4*	534928	protein-coding	-0.6101769	7.78644331	11.884216	0.00153292	0.02119592
ENSBTAG00000003919	*KIAA0408*	100140348	protein-coding	-0.8896531	4.35569949	11.8757861	0.00153805	0.02124595
ENSBTAG00000030599	*SMOC1*	508379	protein-coding	1.02136106	2.65901673	11.8642803	0.00154508	0.02129711
ENSBTAG00000008216	*RRM2*	508167	protein-coding	1.2349805	1.37976363	11.8622982	0.00154629	0.02129711
ENSBTAG00000018773	*RND1*	508869	protein-coding	0.79943596	1.97465556	11.8562485	0.00155001	0.02130654
ENSBTAG00000021377	*S100A14*	618250	protein-coding	0.56832307	5.01649121	11.848311	0.00155489	0.02134963
ENSBTAG00000013674	*EDNRA*	281749	protein-coding	-0.5258812	2.7693045	11.827604	0.00156771	0.02144306
ENSBTAG00000053050	*SAMD9*	514205	protein-coding	0.77387317	4.30755611	11.8265111	0.00156839	0.02144306
ENSBTAG00000053476	*WDR36*	514267	protein-coding	-0.7259049	1.0444628	11.8187909	0.0015732	0.02145893
ENSBTAG00000014628	*OAS2*	529660	protein-coding	1.73760475	0.84100795	11.8186563	0.00157329	0.02145893
ENSBTAG00000040001	*MARVELD2*	541110	protein-coding	0.79287997	1.98504984	11.7826955	0.00159591	0.02166252
ENSBTAG00000051975				1.16714327	-0.5446057	11.7587324	0.00161118	0.02184868
ENSBTAG00000016676	*PTER*	782020	protein-coding	0.61507592	3.81295909	11.724645	0.00163316	0.02204067
ENSBTAG00000051541				1.63370968	-0.1819366	11.7017903	0.00164808	0.02217563
ENSBTAG00000054727				0.61676885	1.98811623	11.6943522	0.00165297	0.02220156
ENSBTAG00000010082	*COL15A1*	100139730	protein-coding	0.50972711	8.00886228	11.6841846	0.00165968	0.02227037
ENSBTAG00000047836	*LOC785629*	785629	pseudo	0.51711322	2.27718264	11.6612983	0.00167488	0.02239655
ENSBTAG00000050114	*SOX11*	107132954	protein-coding	1.52026613	-0.0734387	11.6580673	0.00167704	0.02239655
ENSBTAG00000000917	*BMP1*	510360	protein-coding	0.54025609	3.72075833	11.6280684	0.00169721	0.02258033
ENSBTAG00000010180	*URB2*	514623	protein-coding	-0.5596394	2.73915595	11.6181681	0.00170393	0.02261648
ENSBTAG00000029926	*MIR1-2*	100312988	ncRNA	0.78368109	3.96583347	11.6169607	0.00170475	0.02261648
ENSBTAG00000019244	*P2RX6*	618262	protein-coding	0.78353212	2.69734894	11.6034665	0.00171396	0.02271718
ENSBTAG00000010091	*ARL6*	519014	protein-coding	-0.7370225	1.77461908	11.583349	0.00172778	0.02286565
ENSBTAG00000002024	*ASB18*	100141009	protein-coding	0.79085813	1.98804944	11.5777679	0.00173163	0.02288681
ENSBTAG00000021202	*ACOT7*	514788	protein-coding	0.56984392	2.82385266	11.545324	0.00175423	0.02314199
ENSBTAG00000000655	*MIPOL1*	528380	protein-coding	0.71912086	1.01746846	11.5156299	0.00177518	0.02328749
ENSBTAG00000005787	*IFT57*	531436	protein-coding	-0.5419445	2.31708533	11.4941188	0.00179053	0.02343386

ENSBTAG00000017566	*TUFT1*	282104	protein-coding	0.96679095	1.18295181	11.4809102	0.00180002	0.02349561
ENSBTAG00000013048	*NIPAL3*	616724	protein-coding	-0.5773465	1.76440455	11.4771574	0.00180273	0.02349561
ENSBTAG00000008060	*SNRNP35*	512142	protein-coding	-0.5836914	1.79743444	11.4588253	0.00181602	0.02362504
ENSBTAG00000007778				-1.3433148	0.23260287	11.4511001	0.00182165	0.02365198
ENSBTAG00000048717				-0.5517037	2.78903402	11.4259077	0.00184014	0.02374139
ENSBTAG00000054179				1.51656874	-0.8198561	11.4217786	0.00184319	0.02375896
ENSBTAG00000052504	*KLF2*	520939	protein-coding	0.80773149	5.03704227	11.4030246	0.00185711	0.02387282
ENSBTAG00000013300	*KCNMA1*	282573	protein-coding	-0.5056703	4.21324849	11.3841774	0.00187122	0.02403217
ENSBTAG00000009400	*FASTKD3*	513260	protein-coding	-0.5817777	1.9958143	11.3500343	0.00189706	0.02425341
ENSBTAG00000002524	*MRI1*	534734	protein-coding	-0.6555107	1.68614517	11.3274672	0.00191435	0.02443008
ENSBTAG00000006731	*SLC7A5*	282369	protein-coding	1.11203098	0.91617045	11.3205171	0.00191971	0.02445412
ENSBTAG00000006030	*MYOG*	281343	protein-coding	0.57266181	5.1041901	11.2923816	0.00194156	0.02462114
ENSBTAG00000001110	*SLC16A5*	531531	protein-coding	-0.5632761	4.61218298	11.2841306	0.00194802	0.02466498
ENSBTAG00000010987	*NFKBIZ*	282713	protein-coding	0.53507814	3.07211145	11.2672186	0.00196134	0.02478258
ENSBTAG00000011586	*HABP4*	514807	protein-coding	-0.6041582	3.99065356	11.2355605	0.00198652	0.02496631
ENSBTAG00000048350				-1.2540093	3.26038179	11.2039092	0.00201204	0.02519381
ENSBTAG00000045547				0.52753829	2.44897494	11.2020292	0.00201357	0.02519381
ENSBTAG00000002880	*SORBS1*	504625	protein-coding	-0.6829286	7.13026993	11.1911199	0.00202246	0.0252204
ENSBTAG00000016103	*CBFB*	614678	protein-coding	-0.5631078	3.36363591	11.1906217	0.00202286	0.0252204
ENSBTAG00000030333	*AOC3*	281002	protein-coding	0.5497111	6.20685887	11.1715812	0.00203848	0.0253477
ENSBTAG00000040151	*GCH1*	286815	protein-coding	0.74497466	1.89417175	11.1529914	0.00205384	0.02547128
ENSBTAG00000039951	*PRMT6*	540228	protein-coding	-0.563399	1.62972065	11.1171601	0.00208381	0.02572017
ENSBTAG00000020050	*MLEC*	515309	protein-coding	-0.5420791	3.68438585	11.1151235	0.00208553	0.02572017
ENSBTAG00000003889	*PER1*	516318	protein-coding	0.73698446	7.31822563	11.1137337	0.0020867	0.02572017
ENSBTAG00000020469	*TMED8*	614106	protein-coding	-1.2916208	-0.1350338	11.0862923	0.00211001	0.02591662
ENSBTAG00000002647	*RGS10*	614550	protein-coding	1.34898706	0.05248999	11.0812341	0.00211433	0.02594711
ENSBTAG00000011662	*SOX18*	519439	protein-coding	0.79195259	3.51656241	11.0760104	0.00211881	0.02597941
ENSBTAG00000011554	*SURF6*	511610	protein-coding	-0.6131838	2.23811684	11.0493355	0.00214183	0.02623882
ENSBTAG00000021329	*ZBTB3*	508118	protein-coding	-0.8772161	0.18199232	11.0419557	0.00214825	0.02629454
ENSBTAG00000009685	*ERI1*	540728	protein-coding	-0.5367198	1.82537591	11.0297482	0.00215891	0.02640204
ENSBTAG00000000095	*CD274*	533834	protein-coding	1.36120539	0.5729203	11.0139464	0.00217279	0.02651142
ENSBTAG00000000928	*SPECC1*	506538	protein-coding	-0.669385	3.52573504	11.0072318	0.00217871	0.02652903
ENSBTAG00000001748	*TRIM45*	539091	protein-coding	0.50208041	4.23992508	10.9962821	0.00218842	0.02660116
ENSBTAG00000011316	*ZC3H12A*	535344	protein-coding	0.52496748	2.18024656	10.9703633	0.00221157	0.02679003
ENSBTAG00000003312	*CHST15*	534506	protein-coding	0.75078556	2.99107974	10.953749	0.00222655	0.02686321
ENSBTAG00000007156	*AGAP2*	504833	protein-coding	-0.5325991	2.32408207	10.9493071	0.00223057	0.0268814
ENSBTAG00000051604				1.59103296	1.2380366	10.9443628	0.00223506	0.02691243
ENSBTAG00000008583	*PALD1*	524694	protein-coding	0.99605106	2.71604666	10.940112	0.00223892	0.02693594
ENSBTAG00000052721				-0.9459571	0.98911927	10.9091896	0.00226725	0.02716075
ENSBTAG00000002127	*TCP11L2*	541012	protein-coding	-0.5424717	4.44354686	10.9004495	0.00227533	0.0272112
ENSBTAG00000011680	*PAPLN*	614084	protein-coding	0.98655449	0.52419699	10.8581665	0.00231485	0.02750309
ENSBTAG00000046153				-0.7990038	3.7611653	10.8463917	0.00232598	0.0275826
ENSBTAG00000021035	*CTSK*	513038	protein-coding	0.51991707	4.68509393	10.8238737	0.00234744	0.027697

ENSBTAG00000012991				-1.1313131	3.15965656	10.8018828	0.00236859	0.02789984
ENSBTAG00000019900	*LNPEP*	521633	protein-coding	-0.6178799	5.23950175	10.7682588	0.00240133	0.02821467
ENSBTAG00000012180	*BMF*	541141	protein-coding	-0.6043167	2.18783614	10.7550539	0.00241432	0.02825216
ENSBTAG00000019949	*DBR1*	530188	protein-coding	-0.5442603	2.5885408	10.7468382	0.00242244	0.02827346
ENSBTAG00000018020	*GNAT1*	281794	protein-coding	1.61191536	0.02143067	10.7419053	0.00242733	0.02828407
ENSBTAG00000014058	*LDB2*	617976	protein-coding	0.79496301	2.04181433	10.7278286	0.00244135	0.0283653
ENSBTAG00000031777	*GDPD5*	532719	protein-coding	0.61832854	1.88682201	10.7268075	0.00244237	0.0283653
ENSBTAG00000016185	*ENAH*	513095	protein-coding	-0.5767998	4.64171817	10.7208103	0.00244836	0.02841152
ENSBTAG00000045625	*NTN3*	787961	protein-coding	-1.858443	-0.9914379	10.7074464	0.00246179	0.02854375
ENSBTAG00000013152	*NIPSNAP1*	614972	protein-coding	0.64174916	3.29634852	10.6873188	0.00248215	0.0287562
ENSBTAG00000015779	*PML*	100138545	protein-coding	0.62900358	3.56667643	10.6787084	0.00249092	0.02876357
ENSBTAG00000009037	*GALNT12*	508488	protein-coding	0.66128765	2.01990125	10.6732692	0.00249647	0.02878
ENSBTAG00000008611	*IGFBP4*	282262	protein-coding	0.65263223	4.50166041	10.6711887	0.0024986	0.02878096
ENSBTAG00000038938	*LOC781710*	781710	protein-coding	1.4816329	1.66740092	10.6622139	0.00250781	0.02886336
ENSBTAG00000051176				1.30043748	1.04324025	10.6376928	0.00253314	0.02905987
ENSBTAG00000020835	*KIAA0513*	507534	protein-coding	-0.5033395	3.83507669	10.6154946	0.00255631	0.02919804
ENSBTAG00000001879	*PER2*	512237	protein-coding	0.62130995	4.19762175	10.600619	0.00257196	0.02928286
ENSBTAG00000054241	*LOC100300896*	100300896	protein-coding	-1.0322212	3.18806079	10.597305	0.00257546	0.02928286
ENSBTAG00000030979	*TUBA3E*	534900	protein-coding	-1.1130934	-0.3575374	10.5722809	0.00260206	0.0295138
ENSBTAG00000003763	*MYO5C*	532574	protein-coding	1.43961396	1.40242847	10.5610438	0.0026141	0.02960267
ENSBTAG00000016486	*SORBS2*	614391	protein-coding	-0.8496461	2.87615934	10.535099	0.00264213	0.02984576
ENSBTAG00000008165	*ITGA2B*	515011	protein-coding	0.50723206	2.55352218	10.524254	0.00265394	0.02990215
ENSBTAG00000012681	*CORO2B*	615274	protein-coding	0.53376769	3.51890604	10.4994216	0.0026812	0.03014596
ENSBTAG00000008545	*ATF3*	515266	protein-coding	2.43618874	6.55148002	10.4791561	0.00270367	0.03032418
ENSBTAG00000014529	*GBP4*	613313	protein-coding	1.23844536	1.93163188	10.4756697	0.00270755	0.03034358
ENSBTAG00000001029	*RASGRP2*	512056	protein-coding	0.743557	3.30023401	10.4720821	0.00271156	0.03036428
ENSBTAG00000008250	*SPRY4*	504593	protein-coding	0.99419283	3.34599571	10.4661456	0.0027182	0.03036617
ENSBTAG00000008625	*PLEKHF1*	782309	protein-coding	0.5550919	2.55304353	10.4567935	0.0027287	0.03041102
ENSBTAG00000019015	*IFITM3*	282255	protein-coding	0.83267929	2.63132864	10.4231023	0.00276687	0.03069066
ENSBTAG00000000256				0.5228658	4.31768973	10.4128756	0.00277857	0.03077194
ENSBTAG00000013405	*CIBAR1*	614070	protein-coding	-0.510162	2.27673391	10.3738767	0.00282367	0.0311478
ENSBTAG00000005416	*PPIL3*	615703	protein-coding	-0.5876903	1.99273363	10.3525917	0.00284862	0.03127707
ENSBTAG00000018227	*SLC4A7*	510209	protein-coding	-0.5456991	5.09823308	10.3100156	0.00289924	0.03170912
ENSBTAG00000006035	*PPP1R1B*	282459	protein-coding	0.84106886	1.5244466	10.3040501	0.0029064	0.03176284
ENSBTAG00000006155	*CCRL2*	515976	protein-coding	-0.7236449	3.00190403	10.2918492	0.00292112	0.03188525
ENSBTAG00000009101	*SH2B3*	614726	protein-coding	0.54471925	2.95389118	10.2910136	0.00292213	0.03188525
ENSBTAG00000015086	*HSD11B1*	282589	protein-coding	0.89726959	0.52241061	10.2558422	0.00296503	0.03215613
ENSBTAG00000011446	*SEMA7A*	614707	protein-coding	0.61578462	4.15133137	10.2534039	0.00296803	0.03215613
ENSBTAG00000029828	*MIR421*	100313263	ncRNA	0.62686278	1.83739103	10.212532	0.00301878	0.03250397
ENSBTAG00000026408				0.72610049	1.0133231	10.2121558	0.00301925	0.03250397
ENSBTAG00000037938	*LOC613444*	613444	protein-coding	-0.5005055	2.26518349	10.1959931	0.00303958	0.03258582
ENSBTAG00000002037	*ATXN7L1*	534448	protein-coding	-0.7456549	2.2856034	10.1770305	0.00306362	0.03279356
ENSBTAG00000009080	*ITGB6*	282644	protein-coding	0.63865763	5.03286262	10.1674066	0.0030759	0.03289805

ENSBTAG00000052037	*LOC104973830*	104973830	ncRNA	0.82458899	1.86590821	10.1524811	0.00309504	0.03295803
ENSBTAG00000010146	*PLXNB3*	504294	protein-coding	1.05767487	0.42451239	10.1522232	0.00309538	0.03295803
ENSBTAG00000004908	*CHRNE*	281688	protein-coding	0.59152354	5.15487875	10.0587347	0.00321825	0.03385155
ENSBTAG00000046101	*ZNF470*	444866	protein-coding	0.73472146	1.5690481	10.0486059	0.00323187	0.03392381
ENSBTAG00000010419	*CYP2R1*	541302	protein-coding	-0.7351356	2.33097806	10.0310414	0.00325564	0.03412242
ENSBTAG00000045943	*CHRNG*	281689	protein-coding	1.0061452	2.11684608	10.008045	0.00328705	0.03440034
ENSBTAG00000007689	*LPIN1*	537224	protein-coding	-0.9953517	6.73340231	9.98896154	0.00331335	0.03457283
ENSBTAG00000027081	*ATP10A*	510723	protein-coding	0.79562042	1.30310245	9.98105688	0.00332432	0.03461025
ENSBTAG00000032051	*SULT1C4*	783020	protein-coding	-0.9358036	1.110502	9.9751244	0.00333257	0.03464493
ENSBTAG00000007780	*CAMK1*	520498	protein-coding	0.71139829	1.3710448	9.92075047	0.00340924	0.03525973
ENSBTAG00000000199	*PDP1*	280891	protein-coding	0.52055167	6.00687632	9.90951792	0.00342532	0.03539995
ENSBTAG00000017371	*RELL2*	529327	protein-coding	0.74982337	0.90182582	9.90187596	0.0034363	0.03543174
ENSBTAG00000016424	*MMP25*	531092	protein-coding	1.311437	0.25569705	9.9017314	0.00343651	0.03543174
ENSBTAG00000009854	*MPHOSPH10*	531149	protein-coding	-0.5475049	3.28262742	9.90037399	0.00343846	0.03543174
ENSBTAG00000004085	*ASF1B*	510538	protein-coding	0.89874611	0.79312629	9.89741363	0.00344273	0.0354438
ENSBTAG00000010030				-0.8896121	1.25571839	9.89443161	0.00344703	0.0354438
ENSBTAG00000020272	*SIK2*	539570	protein-coding	-0.5406398	4.36393658	9.84122832	0.0035248	0.03608369
ENSBTAG00000011869	*CSRP3*	540407	protein-coding	-1.0834757	9.50158273	9.81633258	0.00356183	0.03640984
ENSBTAG00000004272	*ISG12(B)*	617420	protein-coding	2.87533338	-0.8251973	9.80314075	0.00358163	0.03653258
ENSBTAG00000007492	*ADAMTSL2*	526780	protein-coding	0.65326795	2.2193413	9.80028357	0.00358593	0.03654997
ENSBTAG00000005359	*TGFB2*	534069	protein-coding	0.52474265	3.57844759	9.75880184	0.00364902	0.03687549
ENSBTAG00000012074	*MYB*	317776	protein-coding	1.88784395	-1.0030062	9.75861682	0.0036493	0.03687549
ENSBTAG00000003525	*STXBP1*	282378	protein-coding	0.63171646	4.17738893	9.73104122	0.0036919	0.03714154
ENSBTAG00000013631	*GLUL*	281199	protein-coding	-0.6500844	9.65137594	9.72885615	0.0036953	0.03714154
ENSBTAG00000012566	*MAP10*	781199	protein-coding	-0.7829572	0.72161869	9.72116187	0.00370729	0.03714154
ENSBTAG00000021837	*RGS19*	539036	protein-coding	0.84765919	1.08974977	9.71420972	0.00371817	0.03722398
ENSBTAG00000010068	*REEP2*	532432	protein-coding	0.6772289	2.80902789	9.70139598	0.0037383	0.03734581
ENSBTAG00000016661	*USP18*	515202	protein-coding	1.00884909	2.02830642	9.69856643	0.00374276	0.03735942
ENSBTAG00000031178	*SLC9A9*	535743	protein-coding	0.68124156	1.52008046	9.69716559	0.00374497	0.03735942
ENSBTAG00000015032	*CD14*	281048	protein-coding	0.7599715	1.71932428	9.67822095	0.003775	0.03758876
ENSBTAG00000030667	*ODAD1*	617306	protein-coding	0.86326552	0.93671953	9.67761244	0.00377597	0.03758876
ENSBTAG00000015434	*DSTN*	513267	protein-coding	-0.6298322	3.94658062	9.65871513	0.00380619	0.0378361
ENSBTAG00000002425	*SCN9A*	533065	protein-coding	1.30223662	0.19367975	9.65308681	0.00381524	0.03789931
ENSBTAG00000030556	*ZNF217*	507766	protein-coding	-0.5649272	2.0204835	9.62808972	0.00385572	0.0382109
ENSBTAG00000022886	*RYR2*	537376	protein-coding	-1.0926267	0.58354842	9.61659317	0.00387449	0.03835255
ENSBTAG00000005278	*WEE1*	540322	protein-coding	0.50113207	3.11620928	9.59049697	0.00391746	0.03864207
ENSBTAG00000048625				-0.626593	1.28804681	9.57569153	0.00394207	0.03876041
ENSBTAG00000009489	*CACNA2D2*	785909	protein-coding	0.80273683	4.33401354	9.55962754	0.00396896	0.03890464
ENSBTAG00000000892	*CGAS*	782472	protein-coding	1.25511354	-0.0668296	9.55243244	0.00398107	0.03899615
ENSBTAG00000033961	*RNF217*	541287	protein-coding	-0.554362	3.68276877	9.53967119	0.00400263	0.03912575
ENSBTAG00000018366	*SLC15A3*	513984	protein-coding	0.92965226	0.47378785	9.53359092	0.00401296	0.03915935
ENSBTAG00000009358	*MTSS2*	521855	protein-coding	0.53342822	3.11627201	9.52431688	0.00402875	0.03917463
ENSBTAG00000007449	*HS3ST1*	538691	protein-coding	1.08381339	0.03431955	9.5236453	0.0040299	0.03917463

ENSBTAG00000051212	*TMEM86B*	518466	protein-coding	1.0681033	-0.1098123	9.50896642	0.00405505	0.03936472
ENSBTAG00000050932				-0.8016513	0.97747847	9.50677208	0.00405882	0.0393742
ENSBTAG00000005445	*SLC35D1*	613734	protein-coding	-0.559224	2.03365397	9.50096787	0.00406882	0.03939735
ENSBTAG00000017542	*PPARD*	353106	protein-coding	0.81026895	4.68360399	9.50066605	0.00406934	0.03939735
ENSBTAG00000005748	*SOX17*	534010	protein-coding	0.61157355	2.66129697	9.50051439	0.0040696	0.03939735
ENSBTAG00000052673				-0.5512971	2.49828316	9.45731603	0.00414488	0.03987925
ENSBTAG00000015080	*PHACTR3*	515071	protein-coding	1.46945812	-0.6493593	9.45183722	0.00415453	0.03994483
ENSBTAG00000012073	*VOPP1*	767861	protein-coding	-0.678079	2.37987641	9.44848452	0.00416045	0.03994944
ENSBTAG00000010954	*ART3*	407147	protein-coding	-0.5838534	6.55115896	9.44606678	0.00416473	0.03994944
ENSBTAG00000048237	*FGF9*	613731	protein-coding	-0.9111063	0.44401166	9.40769932	0.00423319	0.04047988
ENSBTAG00000009984	*SGPL1*	522515	protein-coding	0.55566537	3.14053494	9.39913153	0.00424864	0.04059003
ENSBTAG00000013118	*CD86*	414345	protein-coding	0.86687726	1.03969374	9.39408049	0.00425778	0.04063221
ENSBTAG00000050186				0.78432975	2.31209194	9.37414469	0.00429405	0.04086754
ENSBTAG00000003521	*KIAA0895L*	512420	protein-coding	1.17312613	-0.3048805	9.36862501	0.00430415	0.04093599
ENSBTAG00000019459	*CHAC2*	511605	protein-coding	-0.5678938	2.13697312	9.33729736	0.00436197	0.0413565
ENSBTAG00000026067	*SH2D1A*	613356	protein-coding	1.29797781	-0.4966977	9.33629296	0.00436384	0.0413565
ENSBTAG00000039129	*LOC510798*	510798	protein-coding	0.81778108	1.2192289	9.31310308	0.00440719	0.04158516
ENSBTAG00000014829	*MYRFL*	781109	protein-coding	1.23011044	-0.4951223	9.31274726	0.00440786	0.04158516
ENSBTAG00000001324	*SLCO2A1*	282468	protein-coding	-0.6611118	2.67015976	9.29255468	0.00444599	0.04183276
ENSBTAG00000009979	*HOXB2*	616129	protein-coding	0.6413051	1.04313998	9.27063464	0.00448779	0.04204494
ENSBTAG00000008499	*TROAP*	505791	protein-coding	1.0151652	0.6877321	9.2641285	0.00450028	0.04211828
ENSBTAG00000001979	*TAF7L*	615711	protein-coding	0.93662651	0.86924192	9.25283449	0.00452204	0.04224241
ENSBTAG00000030208	*ERFE*	101905782	protein-coding	1.22650839	0.56443188	9.24262895	0.0045418	0.0423661
ENSBTAG00000015104	*CCDC28B*	507607	protein-coding	0.58014812	1.59858428	9.23143468	0.00456359	0.04251296
ENSBTAG00000011234	*FOXO3*	535530	protein-coding	-0.6522105	4.33612861	9.21690442	0.00459203	0.04266558
ENSBTAG00000014048	*SHLD1*	615129	protein-coding	-0.8397138	0.93598068	9.2159985	0.00459381	0.04266558
ENSBTAG00000010948	*CLIC2*	532777	protein-coding	0.57300365	3.66305256	9.19321749	0.00463881	0.04298612
ENSBTAG00000002083	*AUTS2*	615936	protein-coding	-0.5057872	5.56097847	9.17569494	0.00467374	0.04323164
ENSBTAG00000004641	*PLEKHA6*	107133475	protein-coding	0.6240319	1.53331356	9.17489935	0.00467534	0.04323164
ENSBTAG00000011511	*IFI16*	506759	protein-coding	0.70747391	2.82040754	9.16161889	0.00470201	0.04342881
ENSBTAG00000011578	*CD44*	281057	protein-coding	0.65335234	4.0944273	9.15552988	0.0047143	0.043489
ENSBTAG00000020764	*CNN2*	539019	protein-coding	0.64368035	3.44031124	9.15483594	0.0047157	0.043489
ENSBTAG00000010977	*FRZB*	281170	protein-coding	0.84141029	5.05143446	9.15379739	0.0047178	0.043489
ENSBTAG00000010866	*BMPER*	534101	protein-coding	1.25191172	-0.3137957	9.13803856	0.00474977	0.04373884
ENSBTAG00000019708	*ACSL6*	506059	protein-coding	0.94361702	0.4118797	9.13613108	0.00475366	0.04373884
ENSBTAG00000014069	*PDK4*	507367	protein-coding	-1.5244738	10.9012435	9.12582965	0.00477471	0.04388545
ENSBTAG00000017639	*RPS6KA3*	511190	protein-coding	-0.6320713	6.55603102	9.12057347	0.00478549	0.04392302
ENSBTAG00000007783	*MYBL2*	510420	protein-coding	0.64090523	1.38883039	9.099823	0.00482829	0.04418924
ENSBTAG00000010549	*IFRD1*	541283	protein-coding	0.81584003	5.68647851	9.08906762	0.00485064	0.04436492
ENSBTAG00000023632	*FBXL8*	614765	protein-coding	0.69361486	0.88514107	9.07865336	0.00487239	0.04453487
ENSBTAG00000039662	*LOC515551*	515551	protein-coding	-0.6767877	5.2715306	9.07364219	0.00488289	0.04460189
ENSBTAG00000001104	*CMTM4*	540247	protein-coding	-1.1042824	-0.6016284	9.05635812	0.00491929	0.04480465
ENSBTAG00000005364	*HMBS*	515614	protein-coding	0.61918687	4.99919998	9.05115258	0.00493031	0.04486043

ENSBTAG00000005150	*HMGB3*	613729	protein-coding	0.91860116	0.43875777	9.03550564	0.0049636	0.04513413
ENSBTAG00000013645	*RFFL*	530263	protein-coding	-0.7537936	1.3519845	9.02570226	0.00498458	0.04529563
ENSBTAG00000032617	*FBXO44*	785057	protein-coding	0.62446879	2.64010839	9.02125849	0.00499412	0.04532381
ENSBTAG00000017188	*ISM1*	506902	protein-coding	-0.6254414	1.57768077	9.01889671	0.0049992	0.04532641
ENSBTAG00000034501	*CFI*	513197	protein-coding	1.71426029	-0.7042897	9.01657064	0.00500421	0.04532641
ENSBTAG00000018920	*TRIM47*	618638	protein-coding	0.54377658	2.83495855	9.00145029	0.00503689	0.04547737
ENSBTAG00000006471	*OSBPL11*	538576	protein-coding	-0.5610707	4.8109195	8.99776624	0.00504488	0.04552038
ENSBTAG00000007020	*ZNF502*	539265	protein-coding	-0.7449861	0.93645098	8.9934484	0.00505427	0.04554672
ENSBTAG00000006025	*AHSA2*	531017	protein-coding	0.54752114	1.05015964	8.98100215	0.00508145	0.0457623
ENSBTAG00000052708	*RC3H1*	100336754	protein-coding	-0.5306711	5.25408786	8.97298983	0.00509902	0.04580523
ENSBTAG00000013486	*KICS2*	535460	protein-coding	-0.8855446	0.47509749	8.97141517	0.00510249	0.04580523
ENSBTAG00000009686	*FBXO15*	505376	protein-coding	-0.7290609	1.03219422	8.95234814	0.00514461	0.04606582
ENSBTAG00000016683	*NFKBIA*	282291	protein-coding	-0.7082118	5.6868504	8.94693391	0.00515663	0.04614417
ENSBTAG00000023372	*MYCN*	616888	protein-coding	1.59058612	-0.3502266	8.93431612	0.00518478	0.04615953
ENSBTAG00000002574	*MYOZ2*	540487	protein-coding	-0.526481	8.12023852	8.93381891	0.00518589	0.04615953
ENSBTAG00000021130	*MFSD10*	507194	protein-coding	0.53029271	3.10506996	8.92511918	0.0052054	0.04621534
ENSBTAG00000003845	*CSRNP1*	515406	protein-coding	1.09109396	3.66261395	8.91933246	0.00521842	0.046228
ENSBTAG00000051625				1.64346824	-0.1550817	8.90162544	0.00525848	0.04643562
ENSBTAG00000008739	*SAMD11*	100140500	protein-coding	0.63163005	3.04453909	8.89901236	0.00526442	0.04645892
ENSBTAG00000031614	*BOLL*	527841	protein-coding	0.61434556	1.67039193	8.89707245	0.00526883	0.04646874
ENSBTAG00000018300	*TNFRSF19*	768037	protein-coding	-0.6834239	3.03948596	8.8914321	0.00528169	0.04655295
ENSBTAG00000019772	*OXTR*	281371	protein-coding	1.18881297	-0.4839143	8.8865774	0.00529278	0.04659237
ENSBTAG00000046752	*PRRT2*	112444325	protein-coding	0.79608456	1.20700324	8.85551399	0.00536435	0.04701658
ENSBTAG00000004780	*CDKL1*	523900	protein-coding	-1.4527835	-0.8683909	8.83964775	0.00540131	0.04720388
ENSBTAG00000001176	*LRRN1*	539619	protein-coding	0.74352329	3.55349631	8.83279126	0.00541736	0.04727515
ENSBTAG00000019814	*PGBD2*	101907155	protein-coding	-0.9574767	0.68434631	8.81780906	0.00545262	0.04755337
ENSBTAG00000011912	*SLC25A25*	527786	protein-coding	0.60840786	7.35727945	8.81060633	0.00546966	0.04767244
ENSBTAG00000003749	*KCNMB4*	520448	protein-coding	-0.654477	3.0055431	8.79559845	0.00550535	0.04791985
ENSBTAG00000053310	*C1QTNF9*	523616	protein-coding	-0.5426943	4.74671839	8.79437793	0.00550826	0.04791985
ENSBTAG00000013923	*QPCT*	281437	protein-coding	0.68008617	1.52042334	8.77558724	0.00555332	0.04810375
ENSBTAG00000027151	*ARHGAP26*	538219	protein-coding	0.64818743	2.7385088	8.75801789	0.00559582	0.04837183
ENSBTAG00000009355	*SNAP91*	516917	protein-coding	-1.7179235	-0.7019148	8.75569637	0.00560146	0.04837183
ENSBTAG00000025308	*STON2*	785664	protein-coding	-0.5916584	3.85421473	8.74762311	0.00562112	0.04848215
ENSBTAG00000003253	*NPPC*	281356	protein-coding	1.51696917	0.82246778	8.73232108	0.00565859	0.04868602
ENSBTAG00000019993	*APLN*	282143	protein-coding	1.22412947	-0.1518939	8.72807155	0.00566904	0.04874617
ENSBTAG00000022986	*TAFA5*	767927	protein-coding	-1.2080972	0.04124251	8.70302046	0.00573109	0.04918957
ENSBTAG00000050339				1.39739024	-0.4090534	8.68460583	0.00577717	0.04943433
ENSBTAG00000008088	*KIF18B*	511170	protein-coding	1.16791653	-0.0660283	8.67516781	0.00580094	0.04947174
ENSBTAG00000001786	*HYKK*	530270	protein-coding	0.53194788	2.06436908	8.6727167	0.00580713	0.04947174
ENSBTAG00000021393	*FLRT2*	539905	protein-coding	0.55399776	2.73060362	8.67095562	0.00581158	0.04947174
ENSBTAG00000009786	*SCIN*	281478	protein-coding	0.94018749	2.24209935	8.66527386	0.00582597	0.04950627
ENSBTAG00000005073	*ZNHIT2*	539138	protein-coding	-0.8907213	0.3126492	8.65431182	0.00585384	0.04960786
ENSBTAG00000011921	*TIGIT*	100140801	protein-coding	2.21743855	-0.6351425	8.63211369	0.00591071	0.04996949

4. Prospected DEGs to T2 vs T1 in Early Weaning group

ENSEMBL	SYMBOL	ENTREZID	GENETYPE	logFC	logCPM	F	PValue	FDR
ENSBTAG00000025848	*RBM3*	509771	protein-coding	1.34013659	5.12694798	110.094328	3.58E-12	5.04E-08
ENSBTAG00000013926	*FCGRT*	338062	protein-coding	-1.2226647	7.08418064	102.451542	9.09E-12	6.39E-08
ENSBTAG00000005754	*PPM1K*	540329	protein-coding	2.05408662	5.35383935	89.7855003	4.80E-11	2.25E-07
ENSBTAG00000010786	*TACC2*	533768	protein-coding	-1.0230238	8.37452628	74.4270842	4.65E-10	1.56E-06
ENSBTAG00000015038	*HADHA*	281810	protein-coding	-0.7644256	7.31435248	72.2267162	6.60E-10	1.56E-06
ENSBTAG00000022058	*UNG*	511381	protein-coding	-1.4118851	7.9015851	71.4696569	7.46E-10	1.56E-06
ENSBTAG00000003512	*MYH7B*	521764	protein-coding	1.3417343	7.71129595	71.2164579	7.78E-10	1.56E-06
ENSBTAG00000014197	*SLC38A4*	527476	protein-coding	-2.4017923	1.31931627	68.5251331	1.21E-09	2.14E-06
ENSBTAG00000003514	*HSF4*	614788	protein-coding	1.00513833	4.74972679	66.4790531	1.72E-09	2.68E-06
ENSBTAG00000005012	*HSPH1*	507165	protein-coding	-1.3114046	5.28098267	63.5651354	2.84E-09	4.00E-06
ENSBTAG00000001879	*PER2*	512237	protein-coding	1.55766992	4.19762175	62.8193536	3.24E-09	4.15E-06
ENSBTAG00000048271	*MICAL3*	532244	protein-coding	-1.0006601	6.4044722	61.9576067	3.78E-09	4.44E-06
ENSBTAG00000051759				-0.7706957	4.09858522	55.6023285	1.23E-08	1.33E-05
ENSBTAG00000013152	*NIPSNAP1*	614972	protein-coding	1.34531785	3.29634852	55.1669746	1.34E-08	1.35E-05
ENSBTAG00000007480	*CIRBP*	507120	protein-coding	1.04839177	6.35886071	53.9715329	1.69E-08	1.58E-05
ENSBTAG00000016048	*CPT1B*	509459	protein-coding	-0.8171802	6.07723072	52.451342	2.28E-08	2.01E-05
ENSBTAG00000003120	*ZNF385B*	613328	protein-coding	1.86494407	1.85641527	51.5582965	2.73E-08	2.19E-05
ENSBTAG00000007484	*ACADS*	511222	protein-coding	-0.6392981	5.90510455	51.106766	2.99E-08	2.19E-05
ENSBTAG00000021912	*DUSP7*	785016	protein-coding	-0.6661809	5.18556464	51.004337	3.05E-08	2.19E-05
ENSBTAG00000014831	*PPP1R3C*	539466	protein-coding	1.346988	7.20342178	50.8982563	3.12E-08	2.19E-05
ENSBTAG00000003072	*ACADVL*	282130	protein-coding	-0.7504063	8.04272919	49.3637854	4.27E-08	2.77E-05
ENSBTAG00000022294	*FADS1*	533107	protein-coding	1.31799008	3.28921006	49.2997038	4.33E-08	2.77E-05
ENSBTAG00000021970	*EYA4*	538786	protein-coding	-0.9746681	6.82448232	47.8665096	5.84E-08	3.57E-05
ENSBTAG00000002185	*RNF7*	515595	protein-coding	0.70386991	5.13247922	47.6140786	6.16E-08	3.61E-05
ENSBTAG00000046841	*IRF2BP2*	781665	protein-coding	-0.85875	5.85808179	47.1724854	6.76E-08	3.81E-05
ENSBTAG00000015119	*SIK3*	506539	protein-coding	-0.797981	5.50892163	46.3212299	8.11E-08	4.39E-05
ENSBTAG00000018258	*SRSF2*	508312	protein-coding	0.52035258	7.27590453	45.9145813	8.86E-08	4.47E-05
ENSBTAG00000016679	*ETFDH*	768074	protein-coding	-0.6008874	6.39359375	45.786221	9.11E-08	4.47E-05
ENSBTAG00000004248	*MLYCD*	512341	protein-coding	-1.2734098	5.24311698	45.7371817	9.20E-08	4.47E-05
ENSBTAG00000010002	*IRF2*	337916	protein-coding	-0.6424887	4.11367125	44.9566642	1.09E-07	5.12E-05
ENSBTAG00000017697	*CTF1*	514803	protein-coding	1.08352365	3.21977452	44.6427254	1.17E-07	5.31E-05
ENSBTAG00000018248	*MGLL*	505290	protein-coding	-0.7516607	6.39316835	44.0873057	1.32E-07	5.81E-05
ENSBTAG00000046664	*TSC22D1*	510359	protein-coding	-0.8166006	5.72630658	43.2765504	1.58E-07	6.75E-05
ENSBTAG00000012370	*MGP*	282660	protein-coding	-0.99595	5.65136816	42.764061	1.78E-07	7.35E-05
ENSBTAG00000001851	*LOC508916*	508916	protein-coding	2.50259242	2.41501041	42.2252139	2.01E-07	8.07E-05
ENSBTAG00000008940	*NPTX1*	513538	protein-coding	-2.6860904	2.238817	41.5351897	2.35E-07	9.18E-05
ENSBTAG00000054844	*HBA1*	100140149	protein-coding	-3.9328313	-0.0496511	41.3905889	2.43E-07	9.23E-05
ENSBTAG00000013306	*ZBTB7C*	614110	protein-coding	1.14857357	1.85832752	40.8341754	2.76E-07	0.00010071
ENSBTAG00000054680	*ST3GAL2*	444879	protein-coding	-0.8416521	5.79325056	40.6912225	2.85E-07	0.00010071
ENSBTAG00000017071	*C1QTNF3*	531659	protein-coding	1.05927623	4.53285159	40.6752132	2.86E-07	0.00010071
ENSBTAG00000054250				1.04072585	2.68683351	40.460766	3.01E-07	0.00010071

ENSBTAG00000014158	*CCNT2*	521089	protein-coding	0.58731569	5.60589022	40.3816485	3.07E-07	0.00010071
ENSBTAG00000007884	*SREBF1*	539361	protein-coding	1.08865126	6.48331898	40.3663992	3.08E-07	0.00010071
ENSBTAG00000052213	*ADIPOQ*	282865	protein-coding	5.82323943	1.51973568	40.1581902	3.23E-07	0.00010312
ENSBTAG00000017360	*HDAC3*	404125	protein-coding	0.52584349	4.56541259	38.8873941	4.36E-07	0.00012781
ENSBTAG00000016532	*MAPKAPK3*	615215	protein-coding	-0.5971949	5.59402952	38.0264059	5.36E-07	0.00015386
ENSBTAG00000053162				-0.6173936	5.73037353	37.6202447	5.91E-07	0.00016525
ENSBTAG00000000251	*ALAD*	510679	protein-coding	-0.8334022	5.51508332	37.4692897	6.13E-07	0.00016525
ENSBTAG00000006370	*CCT3*	504735	protein-coding	-0.613183	6.44522562	37.4344559	6.18E-07	0.00016525
ENSBTAG00000032292	*CCDC159*	785202	protein-coding	1.18373734	3.2834362	37.4073194	6.22E-07	0.00016525
ENSBTAG00000055207	*SCD*	280924	protein-coding	1.53029367	6.29933393	37.1192354	6.67E-07	0.00017398
ENSBTAG00000000342	*PLOD3*	514996	protein-coding	-0.6389802	3.95286196	36.5506676	7.67E-07	0.00019466
ENSBTAG00000038464	*PLIN5*	531199	protein-coding	-1.0728858	4.62063681	36.5129622	7.74E-07	0.00019466
ENSBTAG00000021508	*LMOD3*	509978	protein-coding	1.08387167	8.16729715	36.3251366	8.11E-07	0.00020032
ENSBTAG00000001417	*ACSM1*	282576	protein-coding	6.41836068	-0.1313018	35.7607177	9.33E-07	0.00022584
ENSBTAG00000011879	*B4GALT2*	100125390	protein-coding	0.50128668	4.62132255	35.7029806	9.47E-07	0.00022584
ENSBTAG00000025458	*DPF3*	787359	protein-coding	-1.0636372	2.72668686	35.4844495	1.00E-06	0.00023453
ENSBTAG00000023891	*RBM20*	525419	protein-coding	0.851454	4.99513763	35.4142898	1.02E-06	0.00023478
ENSBTAG00000037526	*FABP4*	281759	protein-coding	4.85687042	2.1718995	35.1879302	1.08E-06	0.00024378
ENSBTAG00000003952	*FBXL2*	616212	protein-coding	1.12264175	1.19474151	35.1357504	1.09E-06	0.00024378
ENSBTAG00000011067	*IL17RA*	525040	protein-coding	-0.7397106	3.47343039	34.9781147	1.14E-06	0.00024753
ENSBTAG00000052909	*C8G*	517356	protein-coding	0.78665396	3.42797568	34.1553609	1.40E-06	0.00029833
ENSBTAG00000021962	*BCLAF3*	511003	protein-coding	0.81271015	3.94918414	33.8776917	1.50E-06	0.00031316
ENSBTAG00000019379	*TRARG1*	527604	protein-coding	6.17617152	0.84803778	33.849138	1.51E-06	0.00031316
ENSBTAG00000014151	*RCSD1*	512205	protein-coding	0.65155764	6.41129436	33.6610862	1.59E-06	0.00032389
ENSBTAG00000013666	*SLC25A29*	507686	protein-coding	0.93237067	3.0706802	33.4636832	1.67E-06	0.00033593
ENSBTAG00000010389	*STBD1*	513376	protein-coding	1.45879953	6.7890088	33.2560949	1.76E-06	0.00034945
ENSBTAG00000008192	*PLPPR2*	520247	protein-coding	1.08054217	4.57685553	32.819122	1.97E-06	0.00037794
ENSBTAG00000019554	*FBP2*	514066	protein-coding	-0.6368541	9.20058468	32.7742896	2.00E-06	0.00037794
ENSBTAG00000016265	*DNAJA1*	528862	protein-coding	-0.664111	4.60442937	32.5528768	2.12E-06	0.00039205
ENSBTAG00000037644	*HBB*	280813	protein-coding	-2.6716753	1.38130493	32.4126187	2.20E-06	0.00039756
ENSBTAG00000038523	*ATP1A4*	537960	protein-coding	1.49995095	3.18901863	32.4005361	2.20E-06	0.00039756
ENSBTAG00000007993	*CORO1C*	515798	protein-coding	-0.5691867	4.32832386	31.9032142	2.51E-06	0.00044748
ENSBTAG00000052243				-0.6417944	4.20973111	31.7044604	2.65E-06	0.00046003
ENSBTAG00000054053				0.59092146	2.85079911	31.4372632	2.84E-06	0.00048201
ENSBTAG00000016580	*TEF*	616076	protein-coding	0.76510693	4.84639851	31.0313441	3.17E-06	0.00053087
ENSBTAG00000008409	*MYC*	511077	protein-coding	-1.2657862	3.20001555	30.9501541	3.24E-06	0.00053619
ENSBTAG00000002644	*KCNQ4*	528741	protein-coding	-0.7846478	5.7925016	30.8139373	3.36E-06	0.0005449
ENSBTAG00000015509	*NAMPT*	520472	protein-coding	-1.1021045	6.65944432	30.8027743	3.37E-06	0.0005449
ENSBTAG00000006754	*DBP*	503577	protein-coding	1.15988881	2.43657003	30.7612895	3.41E-06	0.0005449
ENSBTAG00000016139	*TMEM120A*	520173	protein-coding	-1.0889404	4.32015572	30.4936824	3.66E-06	0.00057916
ENSBTAG00000019302	*BCL2*	281020	protein-coding	-1.2227831	2.14262997	30.3426965	3.81E-06	0.00059371
ENSBTAG00000015543	*ARHGAP35*	540310	protein-coding	-0.617735	6.83932883	30.3201426	3.84E-06	0.00059371
ENSBTAG00000051176				2.08106884	1.04324025	30.2143483	3.95E-06	0.00060438

ENSBTAG00000007969	*CIDEC*	534607	protein-coding	4.80171821	1.00241546	30.1034159	4.07E-06	0.00060677
ENSBTAG00000013961	*MYSM1*	515332	protein-coding	0.55825102	5.33863332	30.0633185	4.12E-06	0.00060677
ENSBTAG00000002348	*SLC4A4*	282360	protein-coding	-1.2616923	3.64431913	30.0115927	4.17E-06	0.00060677
ENSBTAG00000004471	*ST3GAL6*	444860	protein-coding	0.71974889	5.04121997	29.9563944	4.24E-06	0.00060677
ENSBTAG00000054661	*MIR2887-1*	100498812	ncRNA	5.01360141	-0.992082	29.9441847	4.25E-06	0.00060677
ENSBTAG00000007564	*BBS12*	516376	protein-coding	-0.7251157	2.48690731	29.9307554	4.27E-06	0.00060677
ENSBTAG00000017528	*SNAI3*	526694	protein-coding	2.22183073	2.72648565	29.7912889	4.43E-06	0.00062406
ENSBTAG00000024605	*DYNLL1*	404151	protein-coding	-0.7146738	3.19108492	29.6848776	4.56E-06	0.00063123
ENSBTAG00000031564	*GNL3L*	507679	protein-coding	0.51823831	6.0373147	29.6636603	4.59E-06	0.00063123
ENSBTAG00000020125	*RBFOX2*	514951	protein-coding	0.70957597	6.10246605	29.6418225	4.62E-06	0.00063123
ENSBTAG00000007943	*AZIN1*	532835	protein-coding	0.56242271	6.59589846	29.3147499	5.05E-06	0.00068405
ENSBTAG00000001842	*GSTM3*	615507	protein-coding	0.98647541	3.70858117	29.1972534	5.22E-06	0.00069989
ENSBTAG00000002382	*DDX10*	528483	protein-coding	-0.7620425	1.93088253	29.0945314	5.37E-06	0.00071328
ENSBTAG00000020030	*FITM2*	518159	protein-coding	-0.8398911	4.86539871	29.0201728	5.48E-06	0.00072133
ENSBTAG00000014724	*EIF4A2*	286819	protein-coding	-0.5026442	8.79584183	28.885926	5.69E-06	0.00074179
ENSBTAG00000014828	*CACNA1A*	282648	protein-coding	0.88195617	2.0494841	28.7425993	5.92E-06	0.0007649
ENSBTAG00000055085				-0.5756706	3.9672797	28.6013535	6.16E-06	0.00078841
ENSBTAG00000014484	*TMEM26*	100125281	protein-coding	-1.1608298	1.73828823	28.5529812	6.25E-06	0.00079194
ENSBTAG00000001305	*ATP2B2*	613800	protein-coding	1.22780793	4.28088822	28.3579289	6.60E-06	0.00082894
ENSBTAG00000019818	*CASZ1*	536765	protein-coding	-0.7509619	4.8855454	28.2513919	6.80E-06	0.00084655
ENSBTAG00000052420	*MIR12060*	114482212	ncRNA	1.31896233	0.64064534	28.1633136	6.97E-06	0.00085269
ENSBTAG00000018936	*LSS*	615906	protein-coding	0.71531286	2.83633693	28.0906317	7.11E-06	0.00086113
ENSBTAG00000014459	*MAP6D1*	614904	protein-coding	0.66223847	2.79279205	28.067131	7.16E-06	0.00086113
ENSBTAG00000000111	*UGP2*	281565	protein-coding	-0.7148354	7.98697257	28.0000877	7.29E-06	0.00087013
ENSBTAG00000020869	*DDC*	280762	protein-coding	-0.8465146	2.27247965	27.9510412	7.40E-06	0.00087485
ENSBTAG00000046990	*GON7*	505131	protein-coding	0.64491605	3.20779031	27.7755095	7.77E-06	0.0009042
ENSBTAG00000045762	*SCAMP4*	538819	protein-coding	-0.5599034	3.43330316	27.7193052	7.90E-06	0.00090686
ENSBTAG00000047441				-0.6389342	4.77257622	27.3251211	8.84E-06	0.00099236
ENSBTAG00000046263	*ADAMTSL5*	107131164	protein-coding	0.72416871	6.64836203	27.3061571	8.88E-06	0.00099236
ENSBTAG00000052115	*LOC100848869*	100848869	protein-coding	0.51222434	8.61175081	27.2593114	9.00E-06	0.00099781
ENSBTAG00000009637	*SLC12A2*	286845	protein-coding	0.83243291	5.03905596	27.0367828	9.60E-06	0.00105518
ENSBTAG00000014215	*CCDC86*	540913	protein-coding	-0.730682	3.39966444	26.9929027	9.72E-06	0.00106027
ENSBTAG00000047265	*PPP1R14B*	617154	protein-coding	0.61755558	5.63721345	26.9022895	9.97E-06	0.00107773
ENSBTAG00000020591	*VAMP4*	616923	protein-coding	0.56090383	2.81868342	26.860393	1.01E-05	0.00107773
ENSBTAG00000019225	*SLC39A14*	515437	protein-coding	-0.7287271	3.87696975	26.7883442	1.03E-05	0.00109074
ENSBTAG00000007187	*INF2*	538493	protein-coding	0.56132806	4.46094331	26.6838179	1.06E-05	0.00111472
ENSBTAG00000013615	*CHORDC1*	505144	protein-coding	-0.6146665	5.44973779	26.661251	1.07E-05	0.00111472
ENSBTAG00000048350				-1.8114139	3.26038179	26.5951545	1.09E-05	0.00112787
ENSBTAG00000046104				0.90594212	2.12060396	26.5096292	1.12E-05	0.00113939
ENSBTAG00000009042	*ATXN7L2*	541097	protein-coding	0.84856531	3.91236319	26.3746992	1.16E-05	0.00116793
ENSBTAG00000015578	*SAP30*	781150	protein-coding	-0.8292972	1.5790817	26.3290404	1.18E-05	0.00117513
ENSBTAG00000000191	*SLC25A20*	518758	protein-coding	-0.5168249	5.63244504	26.0032418	1.29E-05	0.00128309
ENSBTAG00000033315	*DNAJC1*	781036	protein-coding	-0.6769056	3.28778936	25.8275492	1.36E-05	0.00132866

ENSBTAG00000052678	*MIR29C*	791043	ncRNA	1.34791705	1.60037987	25.8099669	1.37E-05	0.00132866
ENSBTAG00000016768	*SCN3B*	540925	protein-coding	-1.6813728	4.03859128	25.4583191	1.52E-05	0.00145461
ENSBTAG00000005630	*VWA7*	505555	protein-coding	0.53618812	4.34954841	25.4163563	1.54E-05	0.0014628
ENSBTAG00000003985	*CCDC15*	512305	protein-coding	0.75755914	3.7772687	25.3924198	1.55E-05	0.0014633
ENSBTAG00000013855	*ORMDL3*	615368	protein-coding	0.53386215	4.99591042	25.3555054	1.57E-05	0.00146949
ENSBTAG00000009788	*ACOT2*	785383	protein-coding	-1.5252053	0.22037739	25.2547181	1.61E-05	0.00148418
ENSBTAG00000016033	*HOXD9*	100295338	protein-coding	-0.6293738	3.70957492	25.2343264	1.62E-05	0.00148418
ENSBTAG00000015214	*CA3*	513212	protein-coding	0.72416816	10.8927957	25.2324843	1.62E-05	0.00148418
ENSBTAG00000011617	*MAMSTR*	505540	protein-coding	-1.0380797	5.28737126	25.2113421	1.63E-05	0.00148418
ENSBTAG00000038316	*GPATCH8*	534759	protein-coding	0.51808068	6.58935871	25.2112075	1.63E-05	0.00148418
ENSBTAG00000013226	*HUS1*	514142	protein-coding	0.68302736	3.15874904	25.083576	1.70E-05	0.00153161
ENSBTAG00000005436	*SHQ1*	507486	protein-coding	-0.6173892	2.27519569	25.0340774	1.72E-05	0.00154442
ENSBTAG00000015350	*PLIN1*	520598	protein-coding	2.5989417	2.26855604	25.0061393	1.74E-05	0.00154746
ENSBTAG00000000459	*POMGNT2*	497030	protein-coding	-0.8206604	2.72282232	24.9264837	1.78E-05	0.00155861
ENSBTAG00000039190	*SLC9A5*	512686	protein-coding	1.11929109	5.27380074	24.9166898	1.78E-05	0.00155861
ENSBTAG00000011666	*THRSP*	515940	protein-coding	3.97143991	0.45878257	24.9138252	1.79E-05	0.00155861
ENSBTAG00000016801	*RXRG*	509500	protein-coding	0.85325906	3.78627101	24.8968125	1.79E-05	0.00155861
ENSBTAG00000016775	*SLC27A1*	513787	protein-coding	-0.5219339	6.47760353	24.8774861	1.80E-05	0.00155861
ENSBTAG00000013029	*ARNTL*	530705	protein-coding	-0.7553011	3.25967994	24.8376722	1.83E-05	0.00156557
ENSBTAG00000004556	*SLC2A3*	282358	protein-coding	-0.8683995	3.60675024	24.8194862	1.84E-05	0.00156557
ENSBTAG00000052788				1.50083064	0.20854223	24.8014807	1.85E-05	0.00156557
ENSBTAG00000038186	*GLRX*	515416	protein-coding	-0.6188612	4.58225966	24.7445445	1.88E-05	0.0015829
ENSBTAG00000053346	*SPSB2*	613651	protein-coding	0.71327101	3.5438641	24.6878782	1.91E-05	0.00160039
ENSBTAG00000049882				2.66322203	-0.4536464	24.6393184	1.94E-05	0.00161423
ENSBTAG00000003836	*ADAM19*	510907	protein-coding	-0.7948212	4.24696451	24.5162818	2.01E-05	0.00165544
ENSBTAG00000050008				0.60249415	4.7384194	24.5160726	2.01E-05	0.00165544
ENSBTAG00000018024	*NR1D2*	532076	protein-coding	0.5681516	5.84103026	24.2467957	2.18E-05	0.00178481
ENSBTAG00000019091	*RNPC3*	768060	protein-coding	0.54209645	4.43856048	24.1227833	2.26E-05	0.0018212
ENSBTAG00000012844	*LRSAM1*	507008	protein-coding	0.64608581	2.67085815	24.0471791	2.32E-05	0.00183484
ENSBTAG00000024240	*ACADM*	505968	protein-coding	-0.6045961	5.99086422	24.0148031	2.34E-05	0.00183484
ENSBTAG00000031295	*HSDL2*	404131	protein-coding	-0.5949123	5.39009294	24.0051158	2.35E-05	0.00183484
ENSBTAG00000004367	*COQ10A*	538821	protein-coding	-0.5408475	7.45325023	23.9267931	2.40E-05	0.00186859
ENSBTAG00000012896	*METTL7B*	506952	protein-coding	-1.3414956	1.17649973	23.8165548	2.48E-05	0.001912
ENSBTAG00000012147	*ARRDC1*	786098	protein-coding	0.804518	3.32233375	23.7323192	2.55E-05	0.00195015
ENSBTAG00000037729	*CERS1*	615296	protein-coding	0.53250312	3.00000023	23.6413215	2.62E-05	0.0019942
ENSBTAG00000047930	*UBE2E2*	784326	protein-coding	0.65559009	4.04758072	23.5091576	2.73E-05	0.00206524
ENSBTAG00000002024	*ASB18*	100141009	protein-coding	1.11002247	1.98804944	23.4272721	2.80E-05	0.00209516
ENSBTAG00000046282	*VGLL2*	541260	protein-coding	0.8698794	6.47862384	23.3614737	2.86E-05	0.00212656
ENSBTAG00000012969	*DKK2*	541161	protein-coding	1.14803999	1.69430926	23.2542076	2.95E-05	0.00217479
ENSBTAG00000002883	*RPTOR*	507056	protein-coding	-0.5021311	4.45086576	23.2284199	2.97E-05	0.0021807
ENSBTAG00000001133	*VWA8*	524159	protein-coding	-0.6637419	4.56655003	23.1687246	3.03E-05	0.00219824
ENSBTAG00000000778	*HSP90AB1*	767874	protein-coding	-0.5213675	8.56561306	23.0760965	3.12E-05	0.00225032
ENSBTAG00000010502	*SERTAD3*	767935	protein-coding	-0.5337064	4.42123942	23.0422807	3.15E-05	0.00226233

ENSBTAG00000037456	*AHDC1*	522105	protein-coding	-0.6709208	5.27092127	22.9631691	3.23E-05	0.0022949
ENSBTAG00000050785				1.0686348	0.77431249	22.9328101	3.26E-05	0.00230491
ENSBTAG00000021537	*RWDD3*	614557	protein-coding	0.95764521	1.61409099	22.8359154	3.36E-05	0.00236325
ENSBTAG00000012178	*NR1D1*	768225	protein-coding	0.89674562	4.73723832	22.806942	3.39E-05	0.00237272
ENSBTAG00000019792	*SLC16A9*	539514	protein-coding	-1.674479	0.3167394	22.7047976	3.50E-05	0.00241315
ENSBTAG00000047339	*BCOR*	784529	protein-coding	-0.6902483	4.26889381	22.6140614	3.60E-05	0.00247012
ENSBTAG00000014649	*CPT2*	504502	protein-coding	-0.6353239	4.06524667	22.5960603	3.62E-05	0.00247194
ENSBTAG00000001609	*MAP2K6*	286883	protein-coding	-0.7389156	6.11989122	22.5420916	3.68E-05	0.00250172
ENSBTAG00000025898	*TBC1D8*	527162	protein-coding	-0.6476234	3.82474485	22.5128898	3.71E-05	0.00251246
ENSBTAG00000054572	*PEG3*	444864	protein-coding	-0.8398857	3.7269962	22.4799797	3.75E-05	0.00252625
ENSBTAG00000047420	*BTNL9*	100125313	protein-coding	-0.9336577	5.22809644	22.3880096	3.86E-05	0.00257524
ENSBTAG00000016109	*DHRS12*	507276	protein-coding	0.72403034	3.53497834	22.304331	3.96E-05	0.00261875
ENSBTAG00000018838	*IQCA1L*	101903592	protein-coding	1.85682815	3.93250363	22.2720227	4.00E-05	0.00263303
ENSBTAG00000046814	*ACOT2*	511033	protein-coding	-1.1972726	2.129367	22.1968176	4.10E-05	0.00268333
ENSBTAG00000050737				-2.2774328	0.36199981	22.1735584	4.13E-05	0.00269047
ENSBTAG00000044058	*MFSD8*	515944	protein-coding	0.52196038	4.33987992	22.0426816	4.30E-05	0.00276508
ENSBTAG00000025550	*PRR22*	616551	protein-coding	1.05018051	1.16540463	22.0073427	4.35E-05	0.00278331
ENSBTAG00000007245	*IFT140*	100139697	protein-coding	-1.0155087	0.87936705	21.9646278	4.41E-05	0.00280827
ENSBTAG00000007988	*STX2*	519148	protein-coding	0.51190397	3.0384753	21.9126726	4.48E-05	0.00282907
ENSBTAG00000005565	*CLN6*	617615	protein-coding	0.59206631	3.04015851	21.8663425	4.55E-05	0.00285794
ENSBTAG00000013401	*ARHGEF40*	527362	protein-coding	-0.7041264	4.50836702	21.651736	4.87E-05	0.00303176
ENSBTAG00000003822	*CROCC*	530641	protein-coding	0.58104977	3.75775442	21.4443	5.20E-05	0.00319906
ENSBTAG00000002012	*CIAO2A*	512871	protein-coding	0.50994667	3.84774155	21.4412741	5.20E-05	0.00319906
ENSBTAG00000021678	*SLC31A1*	518976	protein-coding	0.73893337	4.63152827	21.4063304	5.26E-05	0.00322081
ENSBTAG00000003808	*HDAC9*	535415	protein-coding	1.26498068	3.02290874	21.3831143	5.30E-05	0.00323069
ENSBTAG00000012070	*INTS2*	534471	protein-coding	0.61501289	3.51666479	21.3598617	5.34E-05	0.00324072
ENSBTAG00000044022	*KIAA1671*	533883	protein-coding	-1.0089477	3.98969782	21.2391306	5.55E-05	0.00333935
ENSBTAG00000048643				1.0234771	1.50977597	21.2033172	5.62E-05	0.00336346
ENSBTAG00000037649	*VIPR2*	790124	protein-coding	0.91591137	1.10523412	21.1256941	5.76E-05	0.00343352
ENSBTAG00000020218	*ANXA7*	533360	protein-coding	0.57150403	5.63626823	20.9336617	6.12E-05	0.00357389
ENSBTAG00000051191				0.98943355	8.05496575	20.7391655	6.52E-05	0.00373021
ENSBTAG00000013162	*HSPA8*	281831	protein-coding	-0.5965628	10.0077127	20.7002209	6.60E-05	0.00374699
ENSBTAG00000050076	*MRLN*	101902281	protein-coding	-0.9353925	4.52002419	20.6559313	6.70E-05	0.0037634
ENSBTAG00000010351	*SNX25*	533174	protein-coding	-0.6255503	2.57563236	20.6211038	6.77E-05	0.00378312
ENSBTAG00000050186				1.14581598	2.31209194	20.5751092	6.87E-05	0.00382474
ENSBTAG00000004432	*CHRDL1*	100336905	protein-coding	0.95505843	3.55297895	20.3879159	7.31E-05	0.00403256
ENSBTAG00000003100	*SMTN*	528013	protein-coding	0.51257069	5.93969459	20.2855968	7.55E-05	0.00410481
ENSBTAG00000003015	*SESN1*	538226	protein-coding	-0.8339011	5.9984065	20.2605483	7.61E-05	0.00412256
ENSBTAG00000020984	*RAPGEF4*	537312	protein-coding	-0.8773369	1.7884974	20.1645895	7.86E-05	0.0042213
ENSBTAG00000032617	*FBXO44*	785057	protein-coding	0.89578048	2.64010839	20.095023	8.04E-05	0.0042788
ENSBTAG00000053238	*KALRN*	540675	protein-coding	-0.7456958	3.41858537	20.0938276	8.04E-05	0.0042788
ENSBTAG00000021021	*C21H15orf39*	784903	protein-coding	-0.7772594	1.14393336	20.0857059	8.06E-05	0.0042788
ENSBTAG00000020252	*NLGN2*	787986	protein-coding	0.8144396	1.18792062	20.0769098	8.09E-05	0.0042788

ENSBTAG00000004908	*CHRNE*	281688	protein-coding	0.8524249	5.15487875	20.0495521	8.16E-05	0.00430114
ENSBTAG00000016401	*OPTN*	534150	protein-coding	0.54573529	8.43556556	20.0247986	8.23E-05	0.00431792
ENSBTAG00000043961	*MPDZ*	536863	protein-coding	0.64192598	6.99785008	20.0149043	8.25E-05	0.00431792
ENSBTAG00000013869	*SH3BP4*	520462	protein-coding	-0.6735441	4.11701964	19.839264	8.74E-05	0.00450729
ENSBTAG00000017597	*ESCO1*	535035	protein-coding	-0.5187372	4.40303912	19.7024634	9.14E-05	0.00468081
ENSBTAG00000001938	*CKS2*	767985	protein-coding	-1.0654242	1.80683198	19.6232967	9.39E-05	0.00477629
ENSBTAG00000008376	*SMRP1*	540767	protein-coding	-0.5268596	4.65772917	19.6193743	9.40E-05	0.00477629
ENSBTAG00000020584	*IFT81*	519602	protein-coding	0.77871989	2.31630783	19.5235126	9.70E-05	0.00491246
ENSBTAG00000010284	*ETNPPL*	515186	protein-coding	0.50056602	5.90488622	19.4836855	9.83E-05	0.00495987
ENSBTAG00000002764	*IGFLR1*	617594	protein-coding	0.64370021	3.25199621	19.3699143	0.00010209	0.005071
ENSBTAG00000010843	*PGRMC2*	534021	protein-coding	0.52936621	6.16404876	19.324931	0.00010363	0.005071
ENSBTAG00000009376	*DPH1*	534099	protein-coding	-0.5408845	3.05676175	19.3166751	0.00010391	0.005071
ENSBTAG00000027425	*CCDC190*	617478	protein-coding	1.40091395	2.14871309	19.3117226	0.00010409	0.005071
ENSBTAG00000001450	*APBB3*	525278	protein-coding	0.55897858	3.24614646	19.3102937	0.00010414	0.005071
ENSBTAG00000019593	*KNOP1*	511442	protein-coding	-0.6025466	2.70135167	19.270131	0.00010554	0.00509373
ENSBTAG00000018520	*SCN1A*	529590	protein-coding	2.43798948	1.78598967	19.258254	0.00010596	0.00509373
ENSBTAG00000016444	*RETREG1*	540068	protein-coding	1.18109915	5.55079677	19.2514369	0.0001062	0.00509373
ENSBTAG00000003719	*TDRD7*	506702	protein-coding	0.55637463	4.01679011	19.2382354	0.00010666	0.00509373
ENSBTAG00000004005	*WDR7*	536469	protein-coding	-0.5367852	4.15446097	19.2357437	0.00010675	0.00509373
ENSBTAG00000022036	*ZNRF3*	535441	protein-coding	-0.9171652	0.97933264	19.1932663	0.00010827	0.00514132
ENSBTAG00000004893	*ACOX3*	510065	protein-coding	-0.6098714	4.3671705	19.1339947	0.00011044	0.0052165
ENSBTAG00000037449	*ACSM5*	100298888	protein-coding	0.61054631	4.93712704	19.0490998	0.00011362	0.00534867
ENSBTAG00000019315	*PLA2G7*	282311	protein-coding	-0.8422755	3.71616605	19.0101327	0.00011511	0.0054008
ENSBTAG00000000671	*PARP3*	507426	protein-coding	1.62728334	2.06730287	18.9895012	0.0001159	0.00542017
ENSBTAG00000021026	*PJA1*	786612	protein-coding	0.56468083	2.52864606	18.9067894	0.00011916	0.00555045
ENSBTAG00000008101	*PCSK5*	528098	protein-coding	-0.7622141	2.5153843	18.8988913	0.00011948	0.00555045
ENSBTAG00000016220	*WIPF3*	786606	protein-coding	-0.6079387	4.21942509	18.8748582	0.00012045	0.005577
ENSBTAG00000001865	*SGCG*	506155	protein-coding	0.7674015	6.34002917	18.8482309	0.00012153	0.00560864
ENSBTAG00000020272	*SIK2*	539570	protein-coding	-0.6927205	4.36393658	18.691532	0.00012811	0.00587376
ENSBTAG00000012106	*ADCK1*	533372	protein-coding	-0.7301133	1.53646317	18.6399717	0.00013035	0.00591894
ENSBTAG00000043302	*LOC112444957*	112444957	snoRNA	0.846688	0.86597993	18.5811238	0.00013297	0.00599545
ENSBTAG00000010392	*ESRRG*	536732	protein-coding	-1.4742124	1.19031337	18.5734057	0.00013332	0.00599545
ENSBTAG00000019402	*CFAP100*	519687	protein-coding	2.56939575	-0.5078763	18.5446926	0.00013462	0.0060267
ENSBTAG00000013303	*ACSS2*	506459	protein-coding	0.6110495	4.65844526	18.539178	0.00013487	0.0060267
ENSBTAG00000014336	*WBP1L*	782651	protein-coding	-0.8153053	3.78474597	18.3526265	0.00014367	0.00635933
ENSBTAG00000015310	*SLC25A36*	510392	protein-coding	0.54403012	6.12507486	18.3184903	0.00014534	0.00638846
ENSBTAG00000030208	*ERFE*	101905782	protein-coding	1.76507283	0.56443188	18.1802492	0.00015234	0.00659806
ENSBTAG00000019805	*RHBDF1*	529167	protein-coding	0.60819078	4.49216785	18.0119664	0.00016135	0.00683718
ENSBTAG00000013863	*DUSP1*	539175	protein-coding	-0.9304521	7.02159698	17.9263624	0.00016615	0.00698269
ENSBTAG00000000087	*HSD17B12*	789567	protein-coding	0.51245621	4.05966415	17.9257222	0.00016618	0.00698269
ENSBTAG00000010637	*ARSG*	513067	protein-coding	-1.274859	2.75580936	17.8937299	0.00016802	0.00701777
ENSBTAG00000005710	*NCAM1*	281941	protein-coding	0.975457	3.60690849	17.8622153	0.00016984	0.00705218
ENSBTAG00000036297	*RBFA*	510927	protein-coding	-0.5383366	3.07591559	17.8288444	0.0001718	0.00710828

ENSBTAG00000032281	*LOC515547*	515547	pseudo	-0.8137887	1.89698811	17.7727809	0.00017514	0.00720835
ENSBTAG00000001511	*BCL6*	539020	protein-coding	-0.6930964	6.65085065	17.7595586	0.00017594	0.00722008
ENSBTAG00000008394	*MYL3*	618352	protein-coding	-0.8029023	9.51870221	17.71811	0.00017846	0.00726194
ENSBTAG00000030599	*SMOC1*	508379	protein-coding	1.22885709	2.65901673	17.7174426	0.0001785	0.00726194
ENSBTAG00000011150	*PFN2*	539034	protein-coding	0.58673596	5.94133233	17.6825187	0.00018066	0.00728953
ENSBTAG00000008370				0.57969106	2.33478752	17.6813545	0.00018074	0.00728953
ENSBTAG00000003051	*FER*	536759	protein-coding	0.61960065	2.71098591	17.6520549	0.00018257	0.00731143
ENSBTAG00000006035	*PPP1R1B*	282459	protein-coding	1.06411792	1.5244466	17.647805	0.00018284	0.00731143
ENSBTAG00000007434	*CD300LG*	615388	protein-coding	-0.5494638	5.79431266	17.5628074	0.00018828	0.00750769
ENSBTAG00000040569	*NLRX1*	539974	protein-coding	0.62275378	2.37986557	17.4815759	0.00019364	0.00763496
ENSBTAG00000004599	*TAGLN3*	515562	protein-coding	0.93559104	2.34319065	17.389004	0.00019995	0.00786167
ENSBTAG00000038132	*AIFM3*	526295	protein-coding	2.58596163	-0.7677141	17.3736372	0.00020102	0.00788165
ENSBTAG00000020000	*RAP1GAP2*	786649	protein-coding	-1.1212355	1.75337173	17.3531842	0.00020245	0.00788408
ENSBTAG00000019733	*ADGRL4*	535066	protein-coding	-0.6124451	4.54302831	17.3472483	0.00020287	0.00788408
ENSBTAG00000030593	*RFTN1*	506782	protein-coding	-0.587713	4.14117188	17.3408071	0.00020332	0.00788408
ENSBTAG00000004256	*ODC1*	281365	protein-coding	-0.683307	6.86521458	17.3225105	0.00020461	0.00791251
ENSBTAG00000016959	*LAPTM4B*	404155	protein-coding	-0.5205902	5.33512296	17.2813316	0.00020756	0.0080045
ENSBTAG00000002402	*IGF2R*	281849	protein-coding	-0.5804229	7.03267572	17.2608664	0.00020904	0.00803962
ENSBTAG00000015505	*FADS2*	521822	protein-coding	0.74209264	4.2397256	17.2467189	0.00021007	0.00804634
ENSBTAG00000017201	*AP4S1*	618267	protein-coding	0.65675617	3.06648591	17.2349863	0.00021093	0.00804634
ENSBTAG00000020417	*DAPK3*	525506	protein-coding	-0.5387016	5.34154406	17.0690614	0.00022349	0.0083666
ENSBTAG00000002028	*DIPK1A*	541171	protein-coding	0.56971399	3.71539476	17.0552317	0.00022457	0.0083848
ENSBTAG00000013557	*ERAP1*	514617	protein-coding	-0.539222	4.43092609	17.0171459	0.00022758	0.00847464
ENSBTAG00000020570	*PRDM8*	507890	protein-coding	0.90345553	0.68416478	17.0086101	0.00022826	0.00847753
ENSBTAG00000000387	*NICN1*	614730	protein-coding	0.51488071	4.32998045	16.9469001	0.00023324	0.00861707
ENSBTAG00000007012	*ZNF3*	618141	protein-coding	0.58331337	3.2383988	16.8873712	0.00023816	0.00875462
ENSBTAG00000020698	*MTHFR*	497032	protein-coding	0.52010151	5.25338353	16.8855046	0.00023831	0.00875462
ENSBTAG00000016520	*LOC786948*	786948	protein-coding	1.27816667	0.87389908	16.8718928	0.00023945	0.00875462
ENSBTAG00000000160	*CBS*	514525	protein-coding	0.92969206	1.95583763	16.8527215	0.00024107	0.00879085
ENSBTAG00000047286				-0.577434	2.58249591	16.7581875	0.00024921	0.00904076
ENSBTAG00000006432	*KCNE4*	615761	protein-coding	-1.0817176	0.32801146	16.7213365	0.00025246	0.00909176
ENSBTAG00000006320	*DBT*	280759	protein-coding	0.60071422	4.25959176	16.6808631	0.00025608	0.00919532
ENSBTAG00000021527	*IGF1R*	281848	protein-coding	-0.7517978	3.90748974	16.5976028	0.00026371	0.00932744
ENSBTAG00000007415	*SLC7A8*	536818	protein-coding	-1.2422296	6.46668273	16.5770689	0.00026562	0.00934731
ENSBTAG00000017746	*SLIT3*	615883	protein-coding	-0.8993946	2.31142709	16.5540451	0.00026779	0.00937673
ENSBTAG00000012885	*ACAT1*	511082	protein-coding	-0.5005675	6.81952426	16.536855	0.00026942	0.00939779
ENSBTAG00000015361	*CNTFR*	539548	protein-coding	-1.0892623	3.62384674	16.5284701	0.00027022	0.00939779
ENSBTAG00000011482	*SLC43A2*	511955	protein-coding	-1.6372112	6.1487181	16.5231789	0.00027073	0.00939779
ENSBTAG00000021919	*NAV1*	531974	protein-coding	0.56760207	3.83814362	16.519672	0.00027106	0.00939779
ENSBTAG00000011847	*ASPN*	507990	protein-coding	-0.7274427	2.98185789	16.5024707	0.00027272	0.00943188
ENSBTAG00000021999	*CPT1A*	506812	protein-coding	-0.9344202	5.09246463	16.478094	0.00027508	0.00949024
ENSBTAG00000017832	*PDK3*	510841	protein-coding	-0.5365754	2.50662438	16.4547693	0.00027736	0.00953258
ENSBTAG00000022520	*BRCA1*	353120	protein-coding	-0.5821835	2.14186316	16.3971913	0.00028307	0.00969477

ENSBTAG00000015805	*SIPA1L1*	787248	protein-coding	-0.5251353	3.38450321	16.3861537	0.00028418	0.00970916
ENSBTAG00000013910	*POLR3GL*	505641	protein-coding	0.56282022	2.12265933	16.3539419	0.00028745	0.00979313
ENSBTAG00000007785	*PKD2L2*	521683	protein-coding	1.66653518	-0.6806019	16.3465713	0.0002882	0.00979313
ENSBTAG00000009655	*TNS3*	516555	protein-coding	-0.5707035	3.5730957	16.3346419	0.00028942	0.00979313
ENSBTAG00000012139	*SIX1*	511371	protein-coding	-0.5032058	5.4528354	16.3021618	0.00029278	0.00988297
ENSBTAG00000011734	*ANKRD1*	510376	protein-coding	1.27023004	9.29023716	16.295297	0.0002935	0.0098834
ENSBTAG00000020469	*TMED8*	614106	protein-coding	-1.5230286	-0.1350338	16.2841388	0.00029466	0.00989898
ENSBTAG00000019052	*ANK3*	511203	protein-coding	0.61836754	7.03864575	16.2457883	0.00029871	0.00996353
ENSBTAG00000007053	*ZFHX2*	539758	protein-coding	-0.6291358	2.47944382	16.2170313	0.00030178	0.01004219
ENSBTAG00000039593	*FAM98C*	508569	protein-coding	0.8463025	1.8848027	16.1814271	0.00030563	0.01011754
ENSBTAG00000029926	*MIR1-2*	100312988	ncRNA	0.90814088	3.96583347	16.17619	0.0003062	0.01011754
ENSBTAG00000010954	*ART3*	407147	protein-coding	-0.7051891	6.55115896	16.1527615	0.00030877	0.01016682
ENSBTAG00000053952				-0.9781205	3.40408693	16.1494096	0.00030914	0.01016682
ENSBTAG00000003510	*MMRN2*	512308	protein-coding	0.5835573	4.47623494	16.1089734	0.00031363	0.01029045
ENSBTAG00000039740	*LY6G5B*	539236	protein-coding	0.6436634	2.38949006	16.0957924	0.0003151	0.01031387
ENSBTAG00000015614	*LNX2*	526424	protein-coding	-0.5310346	4.29110534	16.0895709	0.00031581	0.01031387
ENSBTAG00000011548	*AMPD1*	512748	protein-coding	0.56917493	9.017041	16.0733341	0.00031764	0.01034981
ENSBTAG00000044202	*CNKSR2*	534112	protein-coding	-0.615419	3.71216559	16.0062408	0.00032535	0.01052785
ENSBTAG00000017567	*ACACA*	281590	protein-coding	0.58427879	3.87684792	15.9407603	0.00033306	0.01074692
ENSBTAG00000008363	*EXD1*	617602	protein-coding	2.52172874	-0.829105	15.9307364	0.00033426	0.01074692
ENSBTAG00000020219	*MSS51*	508577	protein-coding	0.95332295	7.80728409	15.9295008	0.00033441	0.01074692
ENSBTAG00000049747				1.16876144	2.32422844	15.9025832	0.00033765	0.01078165
ENSBTAG00000017540	*FAM13C*	540918	protein-coding	-0.8745749	2.75913939	15.8707752	0.00034152	0.0108517
ENSBTAG00000005259	*UCP3*	281563	protein-coding	-1.1338553	8.34374545	15.8621522	0.00034258	0.01085683
ENSBTAG00000022920	*RNF128*	535869	protein-coding	0.63784473	3.86572927	15.8569057	0.00034323	0.01085683
ENSBTAG00000021294	*VWA1*	505917	protein-coding	0.60229936	3.1358257	15.8302259	0.00034653	0.01093673
ENSBTAG00000020073	*NCEH1*	534212	protein-coding	-0.6725861	3.06442967	15.806243	0.00034953	0.01100667
ENSBTAG00000032450				0.98452269	1.12569747	15.7983082	0.00035053	0.01100968
ENSBTAG00000049410	*PIERCE1*	618654	protein-coding	-0.5776734	2.80552584	15.7930579	0.00035119	0.01100968
ENSBTAG00000000125	*SPATA5*	100297992	protein-coding	-0.6568635	2.50259028	15.762943	0.00035501	0.01110479
ENSBTAG00000054514				0.97792506	3.48053497	15.742948	0.00035758	0.01116016
ENSBTAG00000014143	*ASB5*	516722	protein-coding	0.76335923	8.05611205	15.7075494	0.00036216	0.01123266
ENSBTAG00000044106	*SPIDR*	512910	protein-coding	-0.8763887	2.23003416	15.7065407	0.00036229	0.01123266
ENSBTAG00000002151	*ASB15*	282591	protein-coding	0.79995357	5.6004104	15.6436287	0.0003706	0.01140167
ENSBTAG00000003212	*NNAT*	353114	protein-coding	0.92238902	2.79808946	15.6398554	0.0003711	0.01140167
ENSBTAG00000031439	*CDC14A*	614777	protein-coding	-0.5770846	3.89863622	15.6390454	0.00037121	0.01140167
ENSBTAG00000026394	*UBALD1*	618672	protein-coding	-0.5954113	5.69202964	15.6286798	0.0003726	0.01140167
ENSBTAG00000013530	*DDAH2*	540386	protein-coding	0.63316944	3.46924992	15.5077094	0.00038925	0.01180303
ENSBTAG00000010939	*CXHXorf58*	515411	protein-coding	1.10670653	0.74383298	15.4540203	0.00039689	0.01193718
ENSBTAG00000050010				-0.5720664	2.49980686	15.352722	0.00041174	0.01230501
ENSBTAG00000002626	*ARHGAP6*	100140016	protein-coding	0.54925803	2.47029394	15.3258749	0.00041578	0.01237811
ENSBTAG00000001344	*METTL21E*	513822	protein-coding	-1.3759457	5.31052369	15.230454	0.00043046	0.01272931
ENSBTAG00000007917	*TSHZ2*	515677	protein-coding	-0.5984158	3.26719905	15.2128506	0.00043323	0.01275758

ENSBTAG00000019065	*EEPD1*	511767	protein-coding	0.60790442	5.10692572	15.2027747	0.00043482	0.01277777
ENSBTAG00000018453	*ANKRD37*	509139	protein-coding	1.50295808	0.60362597	15.1247587	0.00044737	0.01302195
ENSBTAG00000050809	*GIPC3*	618265	protein-coding	1.18894932	-0.0205345	15.1198407	0.00044818	0.01302195
ENSBTAG00000010564	*ELOVL6*	533333	protein-coding	1.51114087	1.28516024	15.1111107	0.00044961	0.01302195
ENSBTAG00000000571	*ARHGAP27*	789296	protein-coding	-0.6278144	3.29154012	15.0923142	0.0004527	0.01308474
ENSBTAG00000017218	*ACER3*	615110	protein-coding	0.92948738	0.90439203	15.0636652	0.00045747	0.01316201
ENSBTAG00000005002	*GALE*	523154	protein-coding	0.80548755	0.95312793	15.0586757	0.0004583	0.01316201
ENSBTAG00000008313	*KLF15*	407241	protein-coding	0.87390021	3.58758725	15.049385	0.00045986	0.01316201
ENSBTAG00000020900	*RPUSD4*	516934	protein-coding	-0.5305843	3.13461415	15.0292362	0.00046327	0.01316201
ENSBTAG00000011461	*ADORA1*	282133	protein-coding	-1.6796608	-0.4277579	15.0289057	0.00046332	0.01316201
ENSBTAG00000018070	*SCNM1*	505644	protein-coding	0.50592063	3.93241281	15.0222138	0.00046446	0.01316201
ENSBTAG00000025329	*IRF2BPL*	613845	protein-coding	-0.5193616	4.70441572	15.020624	0.00046473	0.01316201
ENSBTAG00000005716	*CRABP2*	493998	protein-coding	1.56242407	0.00095383	14.9973462	0.00046871	0.01322413
ENSBTAG00000020701	*MEF2C*	512254	protein-coding	0.50475136	7.24861409	14.9967898	0.0004688	0.01322413
ENSBTAG00000047801	*CRELD2*	515222	protein-coding	-0.7487925	1.77290842	14.9760104	0.00047238	0.01327197
ENSBTAG00000013492	*PRKAG3*	511961	protein-coding	0.96719497	5.86943544	14.9508398	0.00047676	0.01336827
ENSBTAG00000005359	*TGFB2*	534069	protein-coding	0.59645379	3.57844759	14.9146141	0.00048314	0.0134891
ENSBTAG00000029906	*MIR133A-2*	100313253	ncRNA	0.73039264	4.54766606	14.8232307	0.00049963	0.01375362
ENSBTAG00000048486	*IQANK1*	506831	protein-coding	0.70856837	2.86251512	14.8217948	0.00049989	0.01375362
ENSBTAG00000017666	*ABCC5*	511769	protein-coding	0.60110266	6.71263389	14.8192168	0.00050037	0.01375362
ENSBTAG00000011680	*PAPLN*	614084	protein-coding	1.15974795	0.52419699	14.8041673	0.00050314	0.01377867
ENSBTAG00000009378				0.86583407	0.92177626	14.7915897	0.00050548	0.0138157
ENSBTAG00000004034	*SESN3*	515914	protein-coding	0.70823981	3.01017209	14.7648013	0.00051049	0.01389863
ENSBTAG00000033137	*PSD3*	531459	protein-coding	-0.7293191	3.02782466	14.7582442	0.00051172	0.01390533
ENSBTAG00000007553	*KCNA5*	508960	protein-coding	-0.8496914	2.58344716	14.7376035	0.00051562	0.01397582
ENSBTAG00000034113	*GALNT10*	787200	protein-coding	-0.6526922	3.08935294	14.7139338	0.00052014	0.01400784
ENSBTAG00000012943	*PPARGC1B*	514750	protein-coding	-0.816504	3.80131788	14.7122404	0.00052047	0.01400784
ENSBTAG00000020266	*SLC19A2*	532860	protein-coding	1.03011254	2.38841177	14.6241772	0.00053766	0.01430646
ENSBTAG00000013981	*SPTSSA*	615641	protein-coding	0.75782774	5.6040813	14.5999763	0.00054249	0.01438062
ENSBTAG00000004625	*PYGB*	505560	protein-coding	-0.5079384	2.37866001	14.5716777	0.0005482	0.01450458
ENSBTAG00000046162	*ZNF385C*	100298918	protein-coding	-1.729483	0.49763085	14.5629595	0.00054997	0.01452414
ENSBTAG00000016377	*IPP*	785293	protein-coding	0.56026925	4.12038361	14.5238714	0.00055798	0.01468074
ENSBTAG00000014642	*NAPRT*	399559	protein-coding	0.60249182	3.1527675	14.5160767	0.0005596	0.01469571
ENSBTAG00000009835	*CACNA1G*	282411	protein-coding	1.7434124	0.54983531	14.4715123	0.00056892	0.01487492
ENSBTAG00000016819	*FABP3*	281758	protein-coding	-0.6185128	6.80099552	14.4683448	0.00056959	0.01487492
ENSBTAG00000014030	*FGD6*	539481	protein-coding	-0.6343589	3.45467517	14.4683144	0.00056959	0.01487492
ENSBTAG00000020831	*CHD1L*	524787	protein-coding	0.68812348	3.46533169	14.4490185	0.00057368	0.01495403
ENSBTAG00000006039	*ARHGDIB*	327676	protein-coding	-0.5028834	5.03801484	14.4035997	0.00058344	0.01512428
ENSBTAG00000013860	*GADD45A*	505463	protein-coding	-0.8282569	4.8485276	14.3652389	0.00059182	0.01528512
ENSBTAG00000001745	*LUM*	280847	protein-coding	0.83316386	4.97876499	14.3202377	0.00060181	0.01531539
ENSBTAG00000049331				-1.372738	2.78942815	14.3165021	0.00060264	0.01531539
ENSBTAG00000049058	*INHBB*	530430	protein-coding	0.91743716	1.90727396	14.3158919	0.00060278	0.01531539
ENSBTAG00000020074	*NKPD1*	789234	protein-coding	1.31486957	0.05946712	14.2535611	0.00061694	0.01559073

ENSBTAG00000012675	*MYO19*	508229	protein-coding	0.65386129	3.28679816	14.2176724	0.00062525	0.01574762
ENSBTAG00000011403	*RUSC2*	507809	protein-coding	0.57047059	5.83345465	14.2091917	0.00062723	0.01576433
ENSBTAG00000003084	*GPATCH2L*	509393	protein-coding	0.50828579	5.24919139	14.2046923	0.00062829	0.01576433
ENSBTAG00000001176	*LRRN1*	539619	protein-coding	0.91610887	3.55349631	14.1938564	0.00063083	0.01580006
ENSBTAG00000019675	*ATXN1*	616399	protein-coding	-0.5808947	4.42152109	14.1633868	0.00063805	0.0159525
ENSBTAG00000012558	*ADAMTS12*	525276	protein-coding	-0.9241649	2.77290496	14.1521483	0.00064074	0.01597242
ENSBTAG00000019672	*LOC574091*	574091	protein-coding	-0.5094925	3.9682515	14.1505566	0.00064112	0.01597242
ENSBTAG00000005244	*RASL11A*	101903585	protein-coding	-1.4091718	0.10640729	14.1402785	0.00064359	0.01600037
ENSBTAG00000018807	*MYBPHL*	538875	protein-coding	0.90751414	1.16666981	14.1364262	0.00064452	0.01600037
ENSBTAG00000019409	*GNPTAB*	509610	protein-coding	-0.6656306	4.8256365	14.1151852	0.00064966	0.01608965
ENSBTAG00000001455	*HOXA7*	615851	protein-coding	-0.6334077	1.93005311	14.0833307	0.00065745	0.01620704
ENSBTAG00000000981	*KLHDC8B*	527321	protein-coding	0.57722895	2.41840362	14.0280806	0.0006712	0.01645949
ENSBTAG00000014602	*CAMTA1*	504811	protein-coding	1.02940576	1.75906963	14.00743	0.00067641	0.01655858
ENSBTAG00000031573	*NMRK2*	780788	protein-coding	-0.6899515	10.6292892	13.9800802	0.00068339	0.01670032
ENSBTAG00000051412	*HBA*	512439	protein-coding	-3.037222	-0.3871582	13.9635201	0.00068765	0.01676452
ENSBTAG00000000442	*RBP4*	281444	protein-coding	1.48351862	1.71506556	13.9606263	0.0006884	0.01676452
ENSBTAG00000019194	*SHLD2*	508335	protein-coding	0.61413597	2.40620371	13.944475	0.00069259	0.01683739
ENSBTAG00000001244	*PLAT*	281407	protein-coding	0.57374531	4.58343806	13.9327304	0.00069565	0.01688269
ENSBTAG00000014915	*ETV5*	541166	protein-coding	0.94468918	3.0435645	13.8529667	0.00071684	0.01717288
ENSBTAG00000048906	*AHNAK2*	527701	protein-coding	0.98118846	1.15698947	13.8445999	0.0007191	0.01717288
ENSBTAG00000040128	*FZD4*	445416	protein-coding	-0.5538308	5.09176055	13.8200615	0.00072577	0.01725676
ENSBTAG00000051586	*ECE2*	281134	protein-coding	-0.7959879	1.48493962	13.8083082	0.000729	0.01730413
ENSBTAG00000002473	*ANGPTL4*	509963	protein-coding	-1.3815104	5.91865452	13.7904393	0.00073392	0.01736253
ENSBTAG00000006200	*SLC25A17*	506631	protein-coding	0.52758044	3.61520097	13.7647812	0.00074106	0.01745937
ENSBTAG00000029954				1.70364279	-0.6832214	13.7623599	0.00074174	0.01745937
ENSBTAG00000047997	*PRPS2*	537688	protein-coding	0.85809444	0.98686106	13.7240588	0.00075254	0.01759596
ENSBTAG00000012247	*MXRA8*	522392	protein-coding	0.74855735	2.81045731	13.7019107	0.00075886	0.01767466
ENSBTAG00000052085				-0.8661477	0.9451822	13.6697344	0.00076815	0.01784242
ENSBTAG00000014614	*ACTA2*	515610	protein-coding	-0.7389112	6.28317246	13.663659	0.00076992	0.017854
ENSBTAG00000017860	*HIPK2*	510158	protein-coding	-0.7394424	3.58659217	13.6324807	0.00077906	0.01803618
ENSBTAG00000017677	*SCG3*	525941	protein-coding	-1.2549303	0.26887383	13.6180883	0.00078331	0.01808129
ENSBTAG00000051359				1.01643337	3.07581741	13.6081159	0.00078628	0.01808129
ENSBTAG00000051307				0.81964762	4.18718981	13.5757343	0.00079599	0.01824802
ENSBTAG00000021447	*SIM2*	100337422	protein-coding	-1.4232072	1.05656055	13.5171501	0.00081387	0.01852589
ENSBTAG00000012976	*WLS*	528123	protein-coding	-0.5469122	3.96541084	13.5145225	0.00081469	0.01852589
ENSBTAG00000012079	*MYLK4*	518025	protein-coding	-0.906945	3.38755201	13.4819575	0.00082483	0.01863604
ENSBTAG00000001920	*POLQ*	535323	protein-coding	-1.2501231	0.6987064	13.4199699	0.0008445	0.01892865
ENSBTAG00000011059	*CDKN2C*	505691	protein-coding	0.59428471	3.01341586	13.4138045	0.00084648	0.01894296
ENSBTAG00000032517	*BCL7A*	616240	protein-coding	-0.7625262	1.22458451	13.3802196	0.00085738	0.0190957
ENSBTAG00000000395	*ADAMTS20*	536137	protein-coding	0.60521048	4.78258603	13.3486962	0.00086774	0.01929597
ENSBTAG00000014972	*PTGER4*	282331	protein-coding	-0.532863	3.79451936	13.3421954	0.0008699	0.01931335
ENSBTAG00000004641	*PLEKHA6*	107133475	protein-coding	0.72511128	1.53331356	13.3326151	0.00087308	0.01935352
ENSBTAG00000046153				-0.838	3.7611653	13.3217817	0.0008767	0.01936412

ENSBTAG00000013943	*ZNF703*	782419	protein-coding	-0.7041336	3.02461949	13.2834926	0.0008896	0.01947602
ENSBTAG00000020958	*LRRC8D*	539204	protein-coding	-0.7062318	1.98115162	13.2639019	0.00089629	0.01949942
ENSBTAG00000007732	*ARPP21*	618648	protein-coding	-0.5229754	5.59048772	13.2594609	0.00089781	0.01950239
ENSBTAG00000021316	*COL4A3*	317711	protein-coding	1.25139588	1.37878317	13.2224852	0.00091059	0.01961436
ENSBTAG00000054931	*CACNG4*	519331	protein-coding	0.80455434	3.58805924	13.2223367	0.00091064	0.01961436
ENSBTAG00000016496	*ZNF641*	538714	protein-coding	-0.7057077	2.11264936	13.2203805	0.00091132	0.01961436
ENSBTAG00000007665	*NPR3*	281358	protein-coding	1.81094455	3.4388718	13.1984475	0.000919	0.01971955
ENSBTAG00000048460				-0.6674679	2.29705121	13.1946192	0.00092035	0.01971955
ENSBTAG00000051102	*KCNK3*	519188	protein-coding	-2.1852701	-0.0224362	13.1874492	0.00092288	0.01974235
ENSBTAG00000006253	*FLNC*	528415	protein-coding	-0.8320193	11.8151321	13.1794644	0.00092571	0.01976185
ENSBTAG00000012338	*FAH*	508724	protein-coding	0.9021463	2.16376037	13.1769436	0.0009266	0.01976185
ENSBTAG00000000448	*BDH1*	534090	protein-coding	1.25118893	1.13645041	13.1711017	0.00092868	0.01977615
ENSBTAG00000038978				-0.9137276	1.19629878	13.1651925	0.00093078	0.01979102
ENSBTAG00000002081	*BMPR1B*	407128	protein-coding	1.01459416	2.54150695	13.1523471	0.00093537	0.01985866
ENSBTAG00000026502	*CYP2D14*	282211	protein-coding	1.0128816	1.71095472	13.1187091	0.00094751	0.02005589
ENSBTAG00000012738	*ZNF827*	104974573	protein-coding	-0.6961363	2.10814873	13.0565063	0.0009704	0.02043559
ENSBTAG00000026972	*MYF5*	281335	protein-coding	0.79548877	1.27195954	13.0425691	0.00097561	0.02046608
ENSBTAG00000018165	*RIPOR2*	539635	protein-coding	-0.7586501	3.58515419	13.0323008	0.00097947	0.0204947
ENSBTAG00000049287	*TSPYL5*	615997	protein-coding	-0.810532	1.30009911	13.0225613	0.00098314	0.0204947
ENSBTAG00000045086				0.68432865	1.99442037	13.0196175	0.00098426	0.0204947
ENSBTAG00000019039	*ZNF239*	100848535	protein-coding	0.63716061	2.51178892	12.9832289	0.00099813	0.02069782
ENSBTAG00000003935	*TMBIM1*	404134	protein-coding	0.51445313	5.95765241	12.9119445	0.00102592	0.02105076
ENSBTAG00000012036	*HR*	785815	protein-coding	-0.6505358	3.32636105	12.8928354	0.00103351	0.02111412
ENSBTAG00000011310	*NALF2*	613918	protein-coding	-0.92738	1.93709675	12.7974971	0.00107227	0.02168571
ENSBTAG00000010196	*NUP43*	520174	protein-coding	0.53394896	4.85081217	12.7361562	0.00109803	0.02197331
ENSBTAG00000015780	*RCN2*	512717	protein-coding	0.60731786	3.87814243	12.7326976	0.0010995	0.02197331
ENSBTAG00000023947	*AVPI1*	614488	protein-coding	-0.5486154	4.10496271	12.7309158	0.00110026	0.02197331
ENSBTAG00000031583	*NUPR2*	614047	protein-coding	0.79131875	0.62635	12.7266283	0.00110209	0.02197331
ENSBTAG00000049066	*LOC616957*	616957	protein-coding	2.47871744	-0.8907132	12.7245408	0.00110298	0.02197331
ENSBTAG00000015140	*RUNDC1*	539525	protein-coding	-0.5567273	2.83741841	12.7042143	0.0011117	0.02207093
ENSBTAG00000048660	*C19H17orf113*	116601118	protein-coding	-1.4612866	-0.1607789	12.6950433	0.00111566	0.02210559
ENSBTAG00000019807	*COL27A1*	513668	protein-coding	0.6972931	2.30239179	12.6632266	0.00112951	0.02229877
ENSBTAG00000007922	*RNF24*	767991	protein-coding	-1.1819249	-0.1950703	12.6432154	0.00113832	0.02237847
ENSBTAG00000046333	*C6H4orf54*	101904203	protein-coding	-0.6320811	7.09234027	12.6138559	0.00115137	0.02254066
ENSBTAG00000008466	*CCNB1IP1*	615932	protein-coding	0.7197951	1.9074892	12.6082875	0.00115387	0.02255749
ENSBTAG00000030247	*NSMF*	615854	protein-coding	0.53355871	3.7767503	12.6036791	0.00115594	0.02255749
ENSBTAG00000045879	*CALHM5*	101903708	protein-coding	-0.6059509	2.1657913	12.6012196	0.00115704	0.02255749
ENSBTAG00000016881	*ANPEP*	404191	protein-coding	1.44042958	1.23743535	12.5339078	0.00118774	0.02312391
ENSBTAG00000011908	*CPQ*	540923	protein-coding	1.32442823	-0.328091	12.5268222	0.00119102	0.02315576
ENSBTAG00000051090	*KLF13*	789710	protein-coding	-0.7228879	2.01089512	12.5153676	0.00119634	0.02322721
ENSBTAG00000031135	*CTXN3*	100125915	protein-coding	1.13806132	3.61735092	12.5013464	0.0012029	0.02332227
ENSBTAG00000003504	*GSS*	525059	protein-coding	0.57934475	2.18021262	12.4951479	0.00120581	0.02334652
ENSBTAG00000014069	*PDK4*	507367	protein-coding	-1.749787	10.9012435	12.4913876	0.00120757	0.02334865

ENSBTAG00000037072	*LOC112441908*	112441908	snRNA	3.39882245	-0.6322429	12.44678	0.00122877	0.02361245
ENSBTAG00000046542	*FAM174C*	616355	protein-coding	-0.6662247	1.72451006	12.4450299	0.00122961	0.02361245
ENSBTAG00000005240				0.60521603	3.28345429	12.439191	0.00123241	0.02363407
ENSBTAG00000017566	*TUFT1*	282104	protein-coding	0.9443267	1.18295181	12.3792645	0.0012616	0.02396527
ENSBTAG00000018214	*SHISA2*	617336	protein-coding	0.9372947	5.82186987	12.3590509	0.00127161	0.02412289
ENSBTAG00000021487	*CIART*	104968463	protein-coding	1.02339622	2.63510368	12.3375196	0.00128237	0.02426156
ENSBTAG00000011896	*GRIP2*	509537	protein-coding	0.75783552	4.13042848	12.2892566	0.00130683	0.0245968
ENSBTAG00000017103	*MAP3K13*	505369	protein-coding	-0.8343116	0.22843952	12.2887831	0.00130708	0.0245968
ENSBTAG00000046724	*CBX6*	513830	protein-coding	-0.5328032	2.2403054	12.1976927	0.00135463	0.02532243
ENSBTAG00000019900	*LNPEP*	521633	protein-coding	-0.6320284	5.23950175	12.1899059	0.00135878	0.02536633
ENSBTAG00000008541	*MGST1*	493719	protein-coding	0.85250778	2.14198421	12.0913394	0.0014125	0.02602408
ENSBTAG00000037778	*CXCL3*	613667	protein-coding	-1.1610354	2.52684074	12.0795163	0.0014191	0.02611141
ENSBTAG00000015343	*DYNC2I2*	531489	protein-coding	-0.5385146	2.23120486	12.0462679	0.00143782	0.0262841
ENSBTAG00000025718	*HMGCLL1*	535508	protein-coding	1.05758495	0.92488909	11.9973924	0.00146582	0.02658881
ENSBTAG00000002361	*TMCC2*	768080	protein-coding	-0.8418045	0.47478958	11.9628843	0.00148594	0.0268845
ENSBTAG00000011959	*GPR19*	540647	protein-coding	-1.2498739	-0.2748239	11.9409641	0.00149888	0.02704418
ENSBTAG00000019596	*GPRC5B*	516334	protein-coding	0.56739273	3.36006856	11.9226857	0.00150976	0.02717559
ENSBTAG00000038286	*PPDPFL*	100126183	protein-coding	-1.2255252	5.34822814	11.8961544	0.00152569	0.02732533
ENSBTAG00000020853	*SLC16A13*	538855	protein-coding	0.78466278	1.92831836	11.8830338	0.00153364	0.02739537
ENSBTAG00000005102	*PHTF2*	520774	protein-coding	0.51057421	5.74431166	11.8355706	0.00156277	0.02780801
ENSBTAG00000013406	*CSRP2*	539381	protein-coding	-0.6431341	2.20165045	11.8302787	0.00156605	0.02780801
ENSBTAG00000018737	*GLMN*	504211	protein-coding	0.51458557	2.58435496	11.8261857	0.0015686	0.02780801
ENSBTAG00000013341	*GYG2*	505258	protein-coding	0.56060869	3.67676767	11.8074683	0.00158029	0.02789591
ENSBTAG00000052977	*LOC112445076*	112445076	pseudo	-0.6370103	1.71805197	11.8024138	0.00158346	0.02789591
ENSBTAG00000001390	*B4GALT4*	511328	protein-coding	0.5391079	1.94026812	11.780095	0.00159756	0.02803897
ENSBTAG00000010047	*TIAM2*	100139161	protein-coding	0.7715401	2.26837298	11.7655053	0.00160685	0.02816682
ENSBTAG00000051920	*MIR29E*	100313099	ncRNA	1.2752639	0.15571823	11.7623749	0.00160885	0.02816682
ENSBTAG00000038733	*EFS*	508559	protein-coding	0.81496438	1.03205561	11.7561892	0.00161281	0.02820108
ENSBTAG00000005021	*SEMA5A*	506636	protein-coding	-0.508217	2.75037792	11.7327422	0.00162791	0.02832447
ENSBTAG00000020679	*CNTN1*	281705	protein-coding	1.05086702	0.096394	11.6667364	0.00167125	0.02876884
ENSBTAG00000001773				-0.5193151	3.73216048	11.6606873	0.00167529	0.02876884
ENSBTAG00000007629	*THAP1*	538615	protein-coding	-0.642914	1.78447528	11.6597166	0.00167593	0.02876884
ENSBTAG00000030456	*SPATA7*	510352	protein-coding	0.50857267	3.67281325	11.6453064	0.00168559	0.02886416
ENSBTAG00000016716	*RRS1*	510956	protein-coding	-0.5687327	2.88541911	11.6315461	0.00169486	0.02890536
ENSBTAG00000012992	*CDH6*	537946	protein-coding	-0.8954714	0.18039205	11.6261877	0.00169849	0.02890536
ENSBTAG00000044071	*HRH2*	521257	protein-coding	0.97645268	2.72014047	11.6196771	0.0017029	0.02890536
ENSBTAG00000027064	*BTBD11*	539986	protein-coding	-1.2356406	1.96439829	11.6110021	0.00170881	0.02890536
ENSBTAG00000014879	*FAM185A*	531996	protein-coding	-0.6726545	2.0535871	11.5899773	0.00172321	0.02904899
ENSBTAG00000048059	*TMEM86A*	532018	protein-coding	-0.6997292	1.81052864	11.5682182	0.00173825	0.02925628
ENSBTAG00000011934	*PCK2*	282856	protein-coding	1.26682619	0.65082787	11.5639401	0.00174122	0.02925628
ENSBTAG00000008739	*SAMD11*	100140500	protein-coding	0.71290425	3.04453909	11.5631954	0.00174174	0.02925628
ENSBTAG00000005678	*AACS*	505842	protein-coding	0.52558074	2.35320392	11.5438214	0.00175528	0.02941879
ENSBTAG00000005574	*CLU*	280750	protein-coding	0.52167091	5.41613567	11.5433708	0.0017556	0.02941879

ENSBTAG00000009434	*PAQR6*	516498	protein-coding	0.51679648	2.96503774	11.4370272	0.00183196	0.03058911
ENSBTAG00000006548	*MCCC1*	513504	protein-coding	0.58696285	3.4560447	11.4101561	0.00185181	0.03075023
ENSBTAG00000048699				0.93685308	2.5492909	11.4091849	0.00185253	0.03075023
ENSBTAG00000018431	*NAV2*	100139508	protein-coding	-0.7622789	3.7346623	11.4033998	0.00185683	0.03075095
ENSBTAG00000014538	*HPCAL4*	531339	protein-coding	-0.8844611	3.00739696	11.3624735	0.0018876	0.03118529
ENSBTAG00000004475	*CDK5R1*	282173	protein-coding	-0.7400381	0.59977375	11.3572823	0.00189154	0.03121377
ENSBTAG00000046178				0.52082664	2.20203815	11.3401119	0.00190464	0.0313564
ENSBTAG00000025929	*MTAP*	782907	protein-coding	-0.596957	1.87394661	11.3371039	0.00190695	0.03135767
ENSBTAG00000051406				0.72344053	0.76471168	11.3293606	0.00191289	0.03141875
ENSBTAG00000014885	*MYOM3*	532872	protein-coding	-0.7050698	7.59871706	11.3212028	0.00191918	0.03145057
ENSBTAG00000009050	*ADIPOR2*	407234	protein-coding	-0.5203634	6.01592529	11.3210475	0.0019193	0.03145057
ENSBTAG00000015942	*DNAJA4*	528615	protein-coding	-0.7034224	6.00964718	11.3127487	0.00192572	0.03146302
ENSBTAG00000013048	*NIPAL3*	616724	protein-coding	-0.5647419	1.76440455	11.2982963	0.00193695	0.03155611
ENSBTAG00000047605	*LOC100337457*	100337457	protein-coding	-0.6295174	4.24304455	11.2493204	0.00197553	0.0319962
ENSBTAG00000003721	*CHST1*	506932	protein-coding	0.51294083	3.43501149	11.2412942	0.00198193	0.0319962
ENSBTAG00000012849	*COL4A1*	282191	protein-coding	-0.5846816	9.10889617	11.2260012	0.00199419	0.03205849
ENSBTAG00000019138	*KIF3C*	777770	protein-coding	0.5305604	2.71523774	11.2256136	0.0019945	0.03205849
ENSBTAG00000000588	*CHODL*	613942	protein-coding	0.68954691	2.63761058	11.2248554	0.00199511	0.03205849
ENSBTAG00000005525	*LHX6*	541261	protein-coding	-0.7779189	2.86775258	11.1729555	0.00203735	0.03250771
ENSBTAG00000007142	*CPPED1*	537938	protein-coding	-0.629528	0.89196638	11.1718568	0.00203825	0.03250771
ENSBTAG00000009620				0.8306725	1.08889657	11.1535346	0.00205339	0.03265938
ENSBTAG00000004249	*TANC2*	529494	protein-coding	0.62037743	3.99008263	11.1495889	0.00205667	0.03267458
ENSBTAG00000017660	*LOC100297302*	100297302	pseudo	0.5203139	3.02911427	11.1418642	0.0020631	0.0326904
ENSBTAG00000010877	*ARMC12*	540812	protein-coding	-1.6711347	2.65864154	11.1235531	0.00207843	0.03282932
ENSBTAG00000001074	*SAYSD1*	618888	protein-coding	-0.7608401	0.86018103	11.121209	0.0020804	0.03282932
ENSBTAG00000015274	*PRL*	280901	protein-coding	-1.8189585	-0.6367476	11.1044366	0.00209457	0.03294336
ENSBTAG00000048774	*ALKAL2*	616690	protein-coding	1.10738398	-0.7169816	11.0952438	0.00210237	0.03296765
ENSBTAG00000045981	*DIRAS1*	618387	protein-coding	1.18062863	-0.4372124	11.085046	0.00211107	0.03302028
ENSBTAG00000010809	*HID1*	540436	protein-coding	-0.5841042	3.69059026	11.0825656	0.00211319	0.03302028
ENSBTAG00000048655	*NT5E*	281363	protein-coding	0.79106896	0.97843135	11.0633159	0.00212973	0.03318253
ENSBTAG00000009907	*MAPK4*	529183	protein-coding	1.32406614	1.65155812	11.0539055	0.00213787	0.03321482
ENSBTAG00000007702	*RPP38*	511723	protein-coding	-0.5488783	1.73321535	11.0318749	0.00215704	0.03340215
ENSBTAG00000005857	*SLC6A1*	407118	protein-coding	0.61844989	4.86457922	11.0221952	0.00216553	0.03349667
ENSBTAG00000010164	*ESPNL*	524380	protein-coding	-0.6363105	1.63754721	10.9893854	0.00219455	0.03377483
ENSBTAG00000012861	*KIF4A*	537127	protein-coding	0.66014977	2.63058813	10.9877468	0.00219601	0.03377483
ENSBTAG00000010123	*APOE*	281004	protein-coding	0.72746319	3.06335681	10.978834	0.00220397	0.03379424
ENSBTAG00000011682	*GLI2*	510255	protein-coding	0.60812997	1.63899362	10.975012	0.0022074	0.0338099
ENSBTAG00000017819	*PMVK*	513533	protein-coding	0.58309501	1.70630084	10.9472986	0.00223239	0.03415556
ENSBTAG00000016676	*PTER*	782020	protein-coding	0.59743702	3.81295909	10.9344438	0.00224409	0.03426003
ENSBTAG00000014252	*SPTLC3*	100336940	protein-coding	-0.6033229	3.05607668	10.9223751	0.00225513	0.03439128
ENSBTAG00000007804	*RILPL2*	533228	protein-coding	-0.6848657	2.53913805	10.9083867	0.002268	0.03447549
ENSBTAG00000001385	*ZSCAN2*	539320	protein-coding	-0.6780914	1.5662292	10.8938527	0.00228145	0.03456802
ENSBTAG00000050978				1.08046464	0.16418346	10.8707742	0.00230299	0.03470759

ENSBTAG00000012774	*RAB7B*	538050	protein-coding	-0.517385	3.11049423	10.8628932	0.0023104	0.03478195
ENSBTAG00000006587	*ZNF367*	540823	protein-coding	0.58563571	1.81322445	10.8419175	0.00233023	0.03500519
ENSBTAG00000050814	*CFAP70*	529891	protein-coding	0.62345779	1.92829515	10.7980548	0.0023723	0.03544844
ENSBTAG00000048717				-0.5337216	2.78903402	10.7877419	0.0023823	0.03556024
ENSBTAG00000036099	*LOC104968656*	104968656	protein-coding	0.55273976	2.32492234	10.7837785	0.00238616	0.0355801
ENSBTAG00000010008	*TTC12*	515758	protein-coding	1.24427011	-0.4435512	10.7399116	0.00242931	0.03598448
ENSBTAG00000003165	*ADAMTS9*	537051	protein-coding	0.57193887	4.86348459	10.7344138	0.00243478	0.03599997
ENSBTAG00000046307	*CEBPD*	281678	protein-coding	-1.2461796	5.19570904	10.7110714	0.00245814	0.03623115
ENSBTAG00000019347	*PLXDC1*	536989	protein-coding	0.76571035	3.1863339	10.7061508	0.00246309	0.0362662
ENSBTAG00000003130	*CHRNA3*	282702	protein-coding	0.90032353	1.52878788	10.6928741	0.00247651	0.03640408
ENSBTAG00000020979	*NGFR*	353110	protein-coding	1.15637533	-0.0600276	10.6831482	0.00248639	0.03645676
ENSBTAG00000017661	*RFX2*	534475	protein-coding	-1.0869111	2.70338079	10.6681203	0.00250175	0.03660558
ENSBTAG00000048873				0.63438446	3.22917443	10.6610743	0.00250898	0.03666113
ENSBTAG00000051298	*PVR*	526865	protein-coding	0.57132875	5.46565481	10.6587997	0.00251132	0.03666113
ENSBTAG00000034147	*OLFML2B*	513053	protein-coding	0.59471303	3.36032047	10.5828846	0.00259075	0.03759532
ENSBTAG00000002092	*PI16*	507058	protein-coding	0.73004129	3.83812259	10.5763912	0.00259767	0.03761815
ENSBTAG00000003457	*ATF5*	515654	protein-coding	0.89765906	2.45912669	10.5599508	0.00261528	0.0376978
ENSBTAG00000036363	*MIR133A-1*	100313361	ncRNA	0.83988935	1.47350077	10.5588932	0.00261641	0.0376978
ENSBTAG00000015535	*NEK8*	532332	protein-coding	0.92101607	0.17346703	10.5569678	0.00261849	0.0376978
ENSBTAG00000009080	*ITGB6*	282644	protein-coding	0.62250479	5.03286262	10.556265	0.00261924	0.0376978
ENSBTAG00000006507	*ADAMTS3*	534082	protein-coding	-1.4182339	-0.2576698	10.5509159	0.00262501	0.03774218
ENSBTAG00000003345	*FAT4*	781683	protein-coding	-0.5697304	4.4381335	10.5323939	0.00264507	0.03795316
ENSBTAG00000019761	*MANBA*	281909	protein-coding	0.81839347	2.13821889	10.5179249	0.00266086	0.03810203
ENSBTAG00000001992	*CYP51A1*	505060	protein-coding	0.66133855	2.53955486	10.5073844	0.00267243	0.03822876
ENSBTAG00000001156	*ST3GAL1*	282351	protein-coding	-0.9474894	0.98141361	10.4980226	0.00268275	0.03829852
ENSBTAG00000001462	*FLT4*	338031	protein-coding	1.27917263	0.3836233	10.4753316	0.00270793	0.03853857
ENSBTAG00000010682	*DDR1*	534092	protein-coding	0.62380831	3.83069288	10.4716861	0.002712	0.03853857
ENSBTAG00000018563	*SFRP2*	510821	protein-coding	-0.9911533	0.71274472	10.4684353	0.00271564	0.03853857
ENSBTAG00000006067	*VMAC*	515212	protein-coding	0.73408362	1.20228556	10.4658533	0.00271853	0.03853857
ENSBTAG00000047088				-0.6172104	2.30738847	10.4632315	0.00272147	0.03853857
ENSBTAG00000003815	*EDEM2*	513253	protein-coding	-0.5221897	2.62610235	10.4561235	0.00272945	0.03855464
ENSBTAG00000005278	*WEE1*	540322	protein-coding	0.51507294	3.11620928	10.4480595	0.00273854	0.03856359
ENSBTAG00000014693	*TMEM88*	507172	protein-coding	-0.5231667	3.07941132	10.4432396	0.00274398	0.03856359
ENSBTAG00000002817	*SLC46A1*	511097	protein-coding	0.71633442	1.37462991	10.4408284	0.00274671	0.03856359
ENSBTAG00000006703	*PTGDR*	515331	protein-coding	-0.5819958	4.48617569	10.410649	0.00278112	0.03887496
ENSBTAG00000035174	*THOC3*	529231	protein-coding	-0.5209727	2.52598897	10.3907361	0.00280408	0.03904077
ENSBTAG00000008836				-0.7563844	1.99481962	10.3650731	0.00283396	0.03937895
ENSBTAG00000006187	*MFAP4*	286766	protein-coding	0.71196314	1.72003074	10.3511592	0.00285031	0.03952804
ENSBTAG00000003946	*NECAB3*	514695	protein-coding	0.76782824	1.96430622	10.2952489	0.00291701	0.04004443
ENSBTAG00000014390	*MTMR9*	521746	protein-coding	-0.5030502	2.88182564	10.2619032	0.00295759	0.04045778
ENSBTAG00000010050				0.65543079	2.05834288	10.2482531	0.00297438	0.04060845
ENSBTAG00000001446	*ABRACL*	505914	protein-coding	-1.1077189	0.35404299	10.240037	0.00298453	0.04070757
ENSBTAG00000019615	*PHTF1*	536504	protein-coding	-0.5199501	2.28825969	10.1895813	0.00304769	0.04122999

ENSBTAG00000045633	*AGTR1*	281607	protein-coding	-0.5352201	3.10262403	10.1871977	0.00305071	0.04122999
ENSBTAG00000031433	*TEX9*	518432	protein-coding	0.90959895	1.10586888	10.1860833	0.00305212	0.04122999
ENSBTAG00000018795	*PMS1*	529491	protein-coding	-0.5110289	3.66141999	10.1827661	0.00305633	0.04124726
ENSBTAG00000051340				-1.4551872	1.24945426	10.1631556	0.00308134	0.04146551
ENSBTAG00000025812				-1.2075067	-0.163781	10.159877	0.00308554	0.04148241
ENSBTAG00000024826	*TECRL*	616643	protein-coding	0.5470014	3.2244313	10.1184623	0.00313916	0.04212278
ENSBTAG00000010419	*CYP2R1*	541302	protein-coding	-0.7359976	2.33097806	10.089377	0.00317741	0.04235343
ENSBTAG00000006563	*KLHL40*	514526	protein-coding	-0.8344872	7.52348913	10.0840655	0.00318445	0.04236199
ENSBTAG00000009199	*GLIS2*	527935	protein-coding	0.55204225	3.14673212	10.0701804	0.00320293	0.04241245
ENSBTAG00000016206	*MAOA*	281293	protein-coding	-0.5428366	3.34844178	10.043109	0.00323929	0.04267893
ENSBTAG00000015609	*PREX1*	527410	protein-coding	-0.6290102	3.10795063	10.0416623	0.00324125	0.04267893
ENSBTAG00000006326	*ALDH1L2*	516864	protein-coding	0.71784329	1.5910066	10.0371379	0.00324737	0.04271961
ENSBTAG00000005431	*LMCD1*	540474	protein-coding	0.54820474	6.87788339	10.0265967	0.00326169	0.04286788
ENSBTAG00000018088	*SETBP1*	617265	protein-coding	0.70182592	4.15076456	10.0129192	0.00328036	0.04303297
ENSBTAG00000015980	*FASN*	281152	protein-coding	0.81174616	5.18927207	9.99954576	0.00329873	0.04319486
ENSBTAG00000044150	*UCK2*	541028	protein-coding	-0.6567515	1.34428754	9.97460997	0.00333329	0.04352442
ENSBTAG00000048916	*LIPG*	509808	protein-coding	1.14339868	1.71640806	9.94250984	0.00337833	0.04403096
ENSBTAG00000005691	*FGF2*	281161	protein-coding	0.53729945	2.13927607	9.92725186	0.00339998	0.04419026
ENSBTAG00000012940	*TMEM25*	504393	protein-coding	1.0962433	0.88975131	9.91693621	0.00341469	0.04434058
ENSBTAG00000000679	*PGM3*	505648	protein-coding	0.50149461	2.25690044	9.90193369	0.00343621	0.04450803
ENSBTAG00000015104	*CCDC28B*	507607	protein-coding	0.59254835	1.59858428	9.90115224	0.00343734	0.04450803
ENSBTAG00000051880				-0.7893303	0.61813597	9.89914238	0.00344023	0.04450803
ENSBTAG00000054037				1.23888889	-0.2143434	9.88846534	0.00345566	0.04458466
ENSBTAG00000050436	*SPAAR*	112441516	protein-coding	-0.7322867	1.26533086	9.8705577	0.0034817	0.04476277
ENSBTAG00000053608				0.53972155	2.61102353	9.87022452	0.00348218	0.04476277
ENSBTAG00000003958	*TMEM220*	504970	protein-coding	0.59243921	1.07803636	9.8647041	0.00349025	0.04482557
ENSBTAG00000011590	*GLIPR1*	767905	protein-coding	-1.5000614	-0.6684518	9.85704725	0.00350148	0.04492877
ENSBTAG00000000240	*AKAP7*	613606	protein-coding	0.77229693	1.83829989	9.80752164	0.00357504	0.04568677
ENSBTAG00000006937				0.619534	1.23699335	9.80420085	0.00358003	0.04568677
ENSBTAG00000050919				-1.441311	-0.7342829	9.79832218	0.00358888	0.04575827
ENSBTAG00000001864	*NR4A3*	528877	protein-coding	2.60264441	5.28162809	9.76601942	0.00363796	0.04609168
ENSBTAG00000005351	*RFXAP*	540760	protein-coding	-0.6247196	1.58589826	9.72791016	0.00369677	0.04651115
ENSBTAG00000047426	*LYPD6*	100337491	protein-coding	0.9419129	0.95966068	9.71914783	0.00371044	0.04651115
ENSBTAG00000003304	*HOXC4*	613740	protein-coding	0.9495482	0.31356831	9.71642395	0.0037147	0.04651115
ENSBTAG00000008732	*ZC3H12C*	512586	protein-coding	0.59673575	3.32073935	9.7122244	0.00372128	0.04651927
ENSBTAG00000021364	*TNK2*	280710	protein-coding	-0.5222474	5.64282608	9.70911891	0.00372615	0.04653885
ENSBTAG00000025903	*GLDC*	507688	protein-coding	1.65897499	-0.697718	9.69268162	0.00375205	0.04673794
ENSBTAG00000009302	*RCAN2*	525636	protein-coding	-0.6538017	2.52094954	9.681881	0.00376918	0.0468465
ENSBTAG00000053699	*ZFP64*	539018	protein-coding	-0.6698123	0.53515364	9.65769754	0.00380782	0.04719873
ENSBTAG00000008763	*PRR32*	783116	protein-coding	1.3107929	3.2923167	9.65501409	0.00381214	0.04719873
ENSBTAG00000007196	*TAGLN*	513463	protein-coding	-0.6598435	5.49308854	9.65478036	0.00381251	0.04719873
ENSBTAG00000014967	*LOC536229*	536229	protein-coding	0.75309408	1.1770692	9.63691188	0.00384138	0.04743095
ENSBTAG00000013317	*RNF168*	613812	protein-coding	-0.5326289	4.07449386	9.63352185	0.00384688	0.04745726

ENSBTAG00000049610				0.57536164	1.82849249	9.61762673	0.0038728	0.04773396
ENSBTAG00000010647	*NTRK2*	505824	protein-coding	0.92779235	0.86562738	9.61561347	0.00387609	0.04773396
ENSBTAG00000015369	*MLLT11*	539519	protein-coding	0.67334391	3.09885591	9.60310113	0.00389665	0.04790321
ENSBTAG00000011659	*PIGZ*	104968672	protein-coding	0.6467162	3.13506158	9.58967513	0.00391883	0.04813385
ENSBTAG00000051975				1.01469521	-0.5446057	9.57880249	0.00393689	0.0481875
ENSBTAG00000039340	*SCN4B*	538100	protein-coding	-0.6314247	7.11337639	9.55899404	0.00397002	0.04850872
ENSBTAG00000053524				1.09909601	-0.4141162	9.55644378	0.00397431	0.04851899
ENSBTAG00000053714	*LOC100296900*	100296900	ncRNA	0.71638545	0.44224493	9.52633128	0.00402532	0.04897178
ENSBTAG00000001153	*TLE2*	533595	protein-coding	0.52647153	3.01829034	9.51225354	0.0040494	0.049126
ENSBTAG00000000042	*PYCR1*	539606	protein-coding	0.71418383	1.86516794	9.50291208	0.00406547	0.0491781
ENSBTAG00000000853				-0.5157208	2.45463152	9.49157622	0.00408506	0.04927273
ENSBTAG00000000271	*MNS1*	532884	protein-coding	0.59951067	1.98911734	9.48756099	0.00409202	0.04931446
ENSBTAG00000018399	*MYH15*	526674	protein-coding	1.54428692	-0.0417643	9.45525083	0.00414852	0.04978221
ENSBTAG00000009614	*NRROS*	510688	protein-coding	-0.5780401	2.26168164	9.4424538	0.00417112	0.04999899
ENSBTAG00000008175	*ZNF713*	100126443	protein-coding	0.69250921	0.48966153	9.44100794	0.00417369	0.04999899
