# Supplementary material for: Early Weaning Possibly Increases the Activity of Lipogenic and Adipogenic Pathways in Intramuscular Adipose Tissue of Nellore Calves
Source: Metabolites. 2023 Sep 21;13(9):1028. doi: 10.3390/metabo13091028 (PMC10536964; doi:10.3390/metabo13091028)
Supplement: Supplementary file 1 [file metabolites-13-01028-s001.zip › Supplementary 3 - GO terms.pdf]

1. Enriched Biological Process of Gene Ontology terms to DEG prospect in EW vs CW into time 1

ID	Description	count	terms	BgRatio	pvalue	p.adjust	qvalue	Count.UpReg	geneID.UpReg	Count.DownReg	geneID.DownReg	GeneRatio.Up	GeneRatio.Down	GeneRatio
GO:0045597	positive regulation of cell differentiation	4	8	117/3649	6.36e-05	6.36e-05	1.65e-02	4	*MAMSTR; MYOD1; RAB7B; ZFP36*	0	0	4/8	0/8	4/8
GO:0051094	positive regulation of developmental process	4	8	174/3649	3.01e-04	3.01e-04	2.81e-02	4	*MAMSTR; MYOD1; RAB7B; ZFP36*	0	0	4/8	0/8	4/8
GO:0051149	positive regulation of muscle cell differentiation	2	8	13/3649	3.24e-04	3.24e-04	2.81e-02	2	*MAMSTR; MYOD1*	0	0	2/8	0/8	2/8
GO:0045595	regulation of cell differentiation	4	8	199/3649	5.05e-04	5.05e-04	3.28e-02	4	*MAMSTR; MYOD1; RAB7B; ZFP36*	0	0	4/8	0/8	4/8
GO:0051147	regulation of muscle cell differentiation	2	8	19/3649	7.06e-04	7.06e-04	3.67e-02	2	*MAMSTR; MYOD1*	0	0	2/8	0/8	2/8
GO:0045637	regulation of myeloid cell differentiation	2	8	35/3649	2.41e-03	2.41e-03	1.05e-01	2	*RAB7B; ZFP36*	0	0	2/8	0/8	2/8
GO:0050793	regulation of developmental process	4	8	338/3649	3.74e-03	3.74e-03	1.32e-01	4	*MAMSTR; MYOD1; RAB7B; ZFP36*	0	0	4/8	0/8	4/8
GO:0051146	striated muscle cell differentiation	2	8	47/3649	4.33e-03	4.33e-03	1.32e-01	2	*MAMSTR; MYOD1*	0	0	2/8	0/8	2/8
GO:2000026	regulation of multicellular organismal development	3	8	182/3649	5.68e-03	5.68e-03	1.32e-01	3	*MYOD1; RAB7B; ZFP36*	0	0	3/8	0/8	3/8
GO:1903311	regulation of mRNA metabolic process	2	8	55/3649	5.89e-03	5.89e-03	1.32e-01	2	*MYOD1; ZFP36*	0	0	2/8	0/8	2/8
GO:0030099	myeloid cell differentiation	2	8	62/3649	7.45e-03	7.45e-03	1.32e-01	2	*RAB7B; ZFP36*	0	0	2/8	0/8	2/8
GO:1903706	regulation of hemopoiesis	2	8	62/3649	7.45e-03	7.45e-03	1.32e-01	2	*RAB7B; ZFP36*	0	0	2/8	0/8	2/8
GO:0042692	muscle cell differentiation	2	8	65/3649	8.16e-03	8.16e-03	1.32e-01	2	*MAMSTR; MYOD1*	0	0	2/8	0/8	2/8
GO:0071396	cellular response to lipid	2	8	79/3649	1.19e-02	1.19e-02	1.32e-01	2	*MYOD1; ZFP36*	0	0	2/8	0/8	2/8
GO:0070887	cellular response to chemical stimulus	4	8	483/3649	1.37e-02	1.37e-02	1.32e-01	4	*HBB; MYOD1; RAB7B; ZFP36*	0	0	4/8	0/8	4/8
GO:0051252	regulation of RNA metabolic process	4	8	484/3649	1.38e-02	1.38e-02	1.32e-01	4	*MAMSTR; MYOD1; RAB7B; ZFP36*	0	0	4/8	0/8	4/8
GO:0071407	cellular response to organic cyclic compound	2	8	86/3649	1.40e-02	1.40e-02	1.32e-01	2	*MYOD1; ZFP36*	0	0	2/8	0/8	2/8
GO:0051254	positive regulation of RNA metabolic process	3	8	253/3649	1.42e-02	1.42e-02	1.32e-01	3	*MAMSTR; MYOD1; ZFP36*	0	0	3/8	0/8	3/8
GO:0014070	response to organic cyclic compound	2	8	104/3649	2.01e-02	2.01e-02	1.32e-01	2	*MYOD1; ZFP36*	0	0	2/8	0/8	2/8
GO:0033993	response to lipid	2	8	106/3649	2.09e-02	2.09e-02	1.32e-01	2	*MYOD1; ZFP36*	0	0	2/8	0/8	2/8
GO:0009081	branched-chain amino acid metabolic process	1	8	10/3649	2.17e-02	2.17e-02	1.32e-01	0	*0*	1	DBT	0/8	1/8	1/8
GO:0030219	megakaryocyte differentiation	1	8	10/3649	2.17e-02	2.17e-02	1.32e-01	1	*RAB7B*	0	0	1/8	0/8	1/8
GO:0031960	response to corticosteroid	1	8	10/3649	2.17e-02	2.17e-02	1.32e-01	1	*ZFP36*	0	0	1/8	0/8	1/8
GO:0032623	interleukin-2 production	1	8	10/3649	2.17e-02	2.17e-02	1.32e-01	1	*ZFP36*	0	0	1/8	0/8	1/8
GO:0032663	regulation of interleukin-2 production	1	8	10/3649	2.17e-02	2.17e-02	1.32e-01	1	*ZFP36*	0	0	1/8	0/8	1/8
GO:0045646	regulation of erythrocyte differentiation	1	8	10/3649	2.17e-02	2.17e-02	1.32e-01	1	*ZFP36*	0	0	1/8	0/8	1/8
GO:0051155	positive regulation of striated muscle cell differentiation	1	8	10/3649	2.17e-02	2.17e-02	1.32e-01	1	*MAMSTR*	0	0	1/8	0/8	1/8
GO:0051384	response to glucocorticoid	1	8	10/3649	2.17e-02	2.17e-02	1.32e-01	1	*ZFP36*	0	0	1/8	0/8	1/8
GO:0060966	regulation of gene silencing by RNA	1	8	10/3649	2.17e-02	2.17e-02	1.32e-01	1	*ZFP36*	0	0	1/8	0/8	1/8
GO:0098781	ncRNA transcription	1	8	10/3649	2.17e-02	2.17e-02	1.32e-01	1	*MYOD1*	0	0	1/8	0/8	1/8
GO:0061061	muscle structure development	2	8	109/3649	2.20e-02	2.20e-02	1.32e-01	2	*MAMSTR; MYOD1*	0	0	2/8	0/8	2/8
GO:0045935	positive regulation of nucleobase-containing compound metabolic process	3	8	298/3649	2.22e-02	2.22e-02	1.32e-01	3	*MAMSTR; MYOD1; ZFP36*	0	0	3/8	0/8	3/8
GO:0019233	sensory perception of pain	1	8	11/3649	2.39e-02	2.39e-02	1.32e-01	0	*0*	1	RETREG1	0/8	1/8	1/8
GO:0030216	keratinocyte differentiation	1	8	11/3649	2.39e-02	2.39e-02	1.32e-01	1	*ZFP36*	0	0	1/8	0/8	1/8
GO:0038066	p38MAPK cascade	1	8	11/3649	2.39e-02	2.39e-02	1.32e-01	1	*ZFP36*	0	0	1/8	0/8	1/8
GO:0043403	skeletal muscle tissue regeneration	1	8	11/3649	2.39e-02	2.39e-02	1.32e-01	1	*MYOD1*	0	0	1/8	0/8	1/8
GO:1904019	epithelial cell apoptotic process	1	8	11/3649	2.39e-02	2.39e-02	1.32e-01	1	*ZFP36*	0	0	1/8	0/8	1/8
GO:0001817	regulation of cytokine production	2	8	116/3649	2.47e-02	2.47e-02	1.32e-01	2	*RAB7B; ZFP36*	0	0	2/8	0/8	2/8

GO:0001816	cytokine production	2	8	118/3649	2.56e-02	2.56e-02	1.32e-01	2	*RAB7B; ZFP36*	0	0	2/8	0/8	2/8
GO:0043966	histone H3 acetylation	1	8	12/3649	2.60e-02	2.60e-02	1.32e-01	1	*MYOD1*	0	0	1/8	0/8	1/8
GO:0048525	negative regulation of viral process	1	8	12/3649	2.60e-02	2.60e-02	1.32e-01	1	*ZFP36*	0	0	1/8	0/8	1/8
GO:0051153	regulation of striated muscle cell differentiation	1	8	12/3649	2.60e-02	2.60e-02	1.32e-01	1	*MAMSTR*	0	0	1/8	0/8	1/8
GO:0000381	regulation of alternative mRNA splicing, via spliceosome	1	8	13/3649	2.82e-02	2.82e-02	1.32e-01	1	*MYOD1*	0	0	1/8	0/8	1/8
GO:0019083	viral transcription	1	8	13/3649	2.82e-02	2.82e-02	1.32e-01	1	*ZFP36*	0	0	1/8	0/8	1/8
GO:0071346	cellular response to interferon-gamma	1	8	13/3649	2.82e-02	2.82e-02	1.32e-01	1	*RAB7B*	0	0	1/8	0/8	1/8
GO:0071345	cellular response to cytokine stimulus	2	8	125/3649	2.85e-02	2.85e-02	1.32e-01	2	*RAB7B; ZFP36*	0	0	2/8	0/8	2/8
GO:0043967	histone H4 acetylation	1	8	14/3649	3.03e-02	3.03e-02	1.32e-01	1	*MYOD1*	0	0	1/8	0/8	1/8
GO:0061157	mRNA destabilization	1	8	14/3649	3.03e-02	3.03e-02	1.32e-01	1	*ZFP36*	0	0	1/8	0/8	1/8
GO:1903312	negative regulation of mRNA metabolic process	1	8	14/3649	3.03e-02	3.03e-02	1.32e-01	1	*ZFP36*	0	0	1/8	0/8	1/8
GO:0030097	hemopoiesis	2	8	130/3649	3.06e-02	3.06e-02	1.32e-01	2	*RAB7B; ZFP36*	0	0	2/8	0/8	2/8
GO:0044344	cellular response to fibroblast growth factor stimulus	1	8	15/3649	3.24e-02	3.24e-02	1.32e-01	1	*ZFP36*	0	0	1/8	0/8	1/8
GO:0045638	negative regulation of myeloid cell differentiation	1	8	15/3649	3.24e-02	3.24e-02	1.32e-01	1	*ZFP36*	0	0	1/8	0/8	1/8
GO:0050779	RNA destabilization	1	8	15/3649	3.24e-02	3.24e-02	1.32e-01	1	*ZFP36*	0	0	1/8	0/8	1/8
GO:0071774	response to fibroblast growth factor	1	8	15/3649	3.24e-02	3.24e-02	1.32e-01	1	*ZFP36*	0	0	1/8	0/8	1/8
GO:0071310	cellular response to organic substance	3	8	346/3649	3.30e-02	3.30e-02	1.32e-01	3	*MYOD1; RAB7B; ZFP36*	0	0	3/8	0/8	3/8
GO:0034097	response to cytokine	2	8	136/3649	3.33e-02	3.33e-02	1.32e-01	2	*RAB7B; ZFP36*	0	0	2/8	0/8	2/8
GO:0048534	hematopoietic or lymphoid organ development	2	8	136/3649	3.33e-02	3.33e-02	1.32e-01	2	*RAB7B; ZFP36*	0	0	2/8	0/8	2/8
GO:0042246	tissue regeneration	1	8	16/3649	3.46e-02	3.46e-02	1.32e-01	1	*MYOD1*	0	0	1/8	0/8	1/8
GO:0045445	myoblast differentiation	1	8	16/3649	3.46e-02	3.46e-02	1.32e-01	1	*MYOD1*	0	0	1/8	0/8	1/8
GO:0045944	positive regulation of transcription by RNA polymerase II	2	8	142/3649	3.61e-02	3.61e-02	1.32e-01	2	*MAMSTR; MYOD1*	0	0	2/8	0/8	2/8
GO:0002520	immune system development	2	8	143/3649	3.66e-02	3.66e-02	1.32e-01	2	*RAB7B; ZFP36*	0	0	2/8	0/8	2/8
GO:0032755	positive regulation of interleukin-6 production	1	8	17/3649	3.67e-02	3.67e-02	1.32e-01	1	*RAB7B*	0	0	1/8	0/8	1/8
GO:0034121	regulation of toll-like receptor signaling pathway	1	8	17/3649	3.67e-02	3.67e-02	1.32e-01	1	*RAB7B*	0	0	1/8	0/8	1/8
GO:0034341	response to interferon-gamma	1	8	17/3649	3.67e-02	3.67e-02	1.32e-01	1	*RAB7B*	0	0	1/8	0/8	1/8
GO:0042147	retrograde transport, endosome to Golgi	1	8	17/3649	3.67e-02	3.67e-02	1.32e-01	1	*RAB7B*	0	0	1/8	0/8	1/8
GO:0045600	positive regulation of fat cell differentiation	1	8	17/3649	3.67e-02	3.67e-02	1.32e-01	1	*ZFP36*	0	0	1/8	0/8	1/8
GO:0045639	positive regulation of myeloid cell differentiation	1	8	17/3649	3.67e-02	3.67e-02	1.32e-01	1	*RAB7B*	0	0	1/8	0/8	1/8
GO:0061014	positive regulation of mRNA catabolic process	1	8	17/3649	3.67e-02	3.67e-02	1.32e-01	1	*ZFP36*	0	0	1/8	0/8	1/8
GO:0031099	regeneration	1	8	18/3649	3.88e-02	3.88e-02	1.32e-01	1	*MYOD1*	0	0	1/8	0/8	1/8
GO:0032635	interleukin-6 production	1	8	18/3649	3.88e-02	3.88e-02	1.32e-01	1	*RAB7B*	0	0	1/8	0/8	1/8
GO:0032675	regulation of interleukin-6 production	1	8	18/3649	3.88e-02	3.88e-02	1.32e-01	1	*RAB7B*	0	0	1/8	0/8	1/8
GO:0000380	alternative mRNA splicing, via spliceosome	1	8	19/3649	4.09e-02	4.09e-02	1.32e-01	1	*MYOD1*	0	0	1/8	0/8	1/8
GO:0009913	epidermal cell differentiation	1	8	19/3649	4.09e-02	4.09e-02	1.32e-01	1	*ZFP36*	0	0	1/8	0/8	1/8
GO:0048639	positive regulation of developmental growth	1	8	19/3649	4.09e-02	4.09e-02	1.32e-01	1	*MYOD1*	0	0	1/8	0/8	1/8
GO:0019080	viral gene expression	1	8	20/3649	4.31e-02	4.31e-02	1.32e-01	1	*ZFP36*	0	0	1/8	0/8	1/8
GO:0030856	regulation of epithelial cell differentiation	1	8	20/3649	4.31e-02	4.31e-02	1.32e-01	1	*ZFP36*	0	0	1/8	0/8	1/8
GO:0032640	tumor necrosis factor production	1	8	20/3649	4.31e-02	4.31e-02	1.32e-01	1	*ZFP36*	0	0	1/8	0/8	1/8
GO:0032680	regulation of tumor necrosis factor production	1	8	20/3649	4.31e-02	4.31e-02	1.32e-01	1	*ZFP36*	0	0	1/8	0/8	1/8

GO:0035195	miRNA-mediated gene silencing	1	8	20/3649	4.31e-02	4.31e-02	1.32e-01	1	*ZFP36*	0	0	1/8	0/8	1/8
GO:0048024	regulation of mRNA splicing, via spliceosome	1	8	20/3649	4.31e-02	4.31e-02	1.32e-01	1	*MYOD1*	0	0	1/8	0/8	1/8
GO:0071706	tumor necrosis factor superfamily cytokine production	1	8	20/3649	4.31e-02	4.31e-02	1.32e-01	1	*ZFP36*	0	0	1/8	0/8	1/8
GO:1903555	regulation of tumor necrosis factor superfamily cytokine production	1	8	20/3649	4.31e-02	4.31e-02	1.32e-01	1	*ZFP36*	0	0	1/8	0/8	1/8
GO:0035194	post-transcriptional gene silencing by RNA	1	8	21/3649	4.52e-02	4.52e-02	1.32e-01	1	*ZFP36*	0	0	1/8	0/8	1/8
GO:0043488	regulation of mRNA stability	1	8	21/3649	4.52e-02	4.52e-02	1.32e-01	1	*ZFP36*	0	0	1/8	0/8	1/8
GO:0043524	negative regulation of neuron apoptotic process	1	8	21/3649	4.52e-02	4.52e-02	1.32e-01	0	*0*	1	RETREG1	0/8	1/8	1/8
GO:0051239	regulation of multicellular organismal process	3	8	393/3649	4.60e-02	4.60e-02	1.32e-01	3	*MYOD1; RAB7B; ZFP36*	0	0	3/8	0/8	3/8
GO:0016441	post-transcriptional gene silencing	1	8	22/3649	4.73e-02	4.73e-02	1.32e-01	1	*ZFP36*	0	0	1/8	0/8	1/8
GO:0030218	erythrocyte differentiation	1	8	22/3649	4.73e-02	4.73e-02	1.32e-01	1	*ZFP36*	0	0	1/8	0/8	1/8
GO:0062207	regulation of pattern recognition receptor signaling pathway	1	8	22/3649	4.73e-02	4.73e-02	1.32e-01	1	*RAB7B*	0	0	1/8	0/8	1/8
GO:0034101	erythrocyte homeostasis	1	8	23/3649	4.94e-02	4.94e-02	1.32e-01	1	*ZFP36*	0	0	1/8	0/8	1/8

2. Enriched Biological Process of Gene Ontology terms to DEG prospect in EW vs CW into time 2

ID	Description	count	terms	BgRatio	pvalue	p.adjust	qvalue	Count.UpReg	geneID.UpReg	Count.DownReg	geneID.DownReg	GeneRatio.Up	GeneRatio.Down	GeneRatio
GO:0032787	monocarboxylic acid metabolic process	12	42	142/3649	3.08e-08	3.08e-08	2.71e-05	7	*ACACA; ADIPOQ; FABP4; FASN; INSIG1; NR1D1; SCD*	5	*CPT1B; FADS3; HPGD; PFKFB2; PRL*	7/42	5/42	12/42
GO:0006631	fatty acid metabolic process	10	42	95/3649	6.56e-08	6.56e-08	2.89e-05	6	*ACACA; ADIPOQ; FABP4; FASN; INSIG1; SCD*	4	*CPT1B; FADS3; HPGD; PRL*	6/42	4/42	10/42
GO:0072330	monocarboxylic acid biosynthetic process	7	42	55/3649	2.15e-06	2.15e-06	6.29e-04	5	*ACACA; FASN; INSIG1; NR1D1; SCD*	2	*FADS3; PRL*	5/42	2/42	7/42
GO:0019752	carboxylic acid metabolic process	12	42	232/3649	6.58e-06	6.58e-06	9.05e-04	7	*ACACA; ADIPOQ; FABP4; FASN; INSIG1; NR1D1; SCD*	5	*CPT1B; FADS3; HPGD; PFKFB2; PRL*	7/42	5/42	12/42
GO:0043436	oxoacid metabolic process	12	42	232/3649	6.58e-06	6.58e-06	9.05e-04	7	*ACACA; ADIPOQ; FABP4; FASN; INSIG1; NR1D1; SCD*	5	*CPT1B; FADS3; HPGD; PFKFB2; PRL*	7/42	5/42	12/42
GO:0044255	cellular lipid metabolic process	12	42	233/3649	6.89e-06	6.89e-06	9.05e-04	8	*ACACA; ADIPOQ; FABP4; FASN; INSIG1; LDLR; LSS; SCD*	4	*CPT1B; FADS3; HPGD; PRL*	8/42	4/42	12/42
GO:0006082	organic acid metabolic process	12	42	234/3649	7.20e-06	7.20e-06	9.05e-04	7	*ACACA; ADIPOQ; FABP4; FASN; INSIG1; NR1D1; SCD*	5	*CPT1B; FADS3; HPGD; PFKFB2; PRL*	7/42	5/42	12/42
GO:0006633	fatty acid biosynthetic process	6	42	48/3649	1.38e-05	1.38e-05	1.52e-03	4	*ACACA; FASN; INSIG1; SCD*	2	*FADS3; PRL*	4/42	2/42	6/42
GO:0044281	small molecule metabolic process	16	42	458/3649	2.14e-05	2.14e-05	2.10e-03	9	*ACACA; ADIPOQ; FABP4; FASN; INSIG1; LDLR; LSS; NR1D1; SCD*	7	*CPT1B; DMAC2L; FADS3; HPGD; PFKFB1; PFKFB2; PRL*	9/42	7/42	16/42
GO:0042632	cholesterol homeostasis	4	42	17/3649	3.24e-05	3.24e-05	2.55e-03	4	*FABP4; INSIG1; LDLR; NR1D1*	0	*0*	4/42	0/42	4/42
GO:0055092	sterol homeostasis	4	42	17/3649	3.24e-05	3.24e-05	2.55e-03	4	*FABP4; INSIG1; LDLR; NR1D1*	0	*0*	4/42	0/42	4/42
GO:0044283	small molecule biosynthetic process	9	42	149/3649	3.48e-05	3.48e-05	2.55e-03	7	*ACACA; ADIPOQ; FASN; INSIG1; LSS; NR1D1; SCD*	2	*FADS3; PRL*	7/42	2/42	9/42
GO:0006629	lipid metabolic process	13	42	325/3649	4.12e-05	4.12e-05	2.74e-03	9	*ACACA; ADIPOQ; FABP4; FASN; INSIG1; LDLR; LSS; NR1D1; SCD*	4	*CPT1B; FADS3; HPGD; PRL*	9/42	4/42	13/42
GO:0046394	carboxylic acid biosynthetic process	7	42	86/3649	4.37e-05	4.37e-05	2.74e-03	5	*ACACA; FASN; INSIG1; NR1D1; SCD*	2	*FADS3; PRL*	5/42	2/42	7/42
GO:0016053	organic acid biosynthetic process	7	42	87/3649	4.71e-05	4.71e-05	2.76e-03	5	*ACACA; FASN; INSIG1; NR1D1; SCD*	2	*FADS3; PRL*	5/42	2/42	7/42
GO:0008610	lipid biosynthetic process	9	42	156/3649	5.02e-05	5.02e-05	2.76e-03	7	*ACACA; FASN; INSIG1; LDLR; LSS; NR1D1; SCD*	2	*FADS3; PRL*	7/42	2/42	9/42
GO:0010565	regulation of cellular ketone metabolic process	4	42	23/3649	1.15e-04	1.15e-04	5.93e-03	3	*ADIPOQ; INSIG1; NR1D1*	1	*PRL*	3/42	1/42	4/42
GO:0050873	brown fat cell differentiation	3	42	10/3649	1.61e-04	1.61e-04	7.87e-03	2	*ADIPOQ; FABP4*	1	*ADRB2*	2/42	1/42	3/42
GO:0045444	fat cell differentiation	5	42	48/3649	1.88e-04	1.88e-04	8.71e-03	4	*ADIPOQ; FABP4; INSIG1; NR1D1*	1	*ADRB2*	4/42	1/42	5/42
GO:0055088	lipid homeostasis	4	42	29/3649	2.92e-04	2.92e-04	1.29e-02	4	*FABP4; INSIG1; LDLR; NR1D1*	0	*0*	4/42	0/42	4/42
GO:0045834	positive regulation of lipid metabolic process	4	42	30/3649	3.35e-04	3.35e-04	1.40e-02	3	*ADIPOQ; LDLR; NR1D1*	1	*PRL*	3/42	1/42	4/42
GO:0062012	regulation of small molecule metabolic process	5	42	58/3649	4.62e-04	4.62e-04	1.85e-02	4	*ADIPOQ; INSIG1; LDLR; NR1D1*	1	*PRL*	4/42	1/42	5/42

GO:0046890	regulation of lipid biosynthetic process	4	42	34/3649	5.48e-04	5.48e-04	2.10e-02	3	*INSIG1; LDLR; NR1D1*	1	*PRL*	3/42	1/42	4/42
GO:0019217	regulation of fatty acid metabolic process	3	42	17/3649	8.62e-04	8.62e-04	3.16e-02	2	*ADIPOQ; INSIG1*	1	*PRL*	2/42	1/42	3/42
GO:2000026	regulation of multicellular organismal development	8	42	182/3649	8.99e-04	8.99e-04	3.16e-02	4	*ADIPOQ; LDLR; NR1D1; PRKCA*	4	*ADRB2; ANGPT2; GJA1; PRL*	4/42	4/42	8/42
GO:0019218	regulation of steroid metabolic process	3	42	18/3649	1.03e-03	1.03e-03	3.34e-02	3	*INSIG1; LDLR; NR1D1*	0	*0*	3/42	0/42	3/42
GO:0046889	positive regulation of lipid biosynthetic process	3	42	18/3649	1.03e-03	1.03e-03	3.34e-02	2	*LDLR; NR1D1*	1	*PRL*	2/42	1/42	3/42
GO:0060348	bone development	4	42	41/3649	1.13e-03	1.13e-03	3.55e-02	1	*INSIG1*	3	*GJA1; SERPINH1; TRIM45*	1/42	3/42	4/42
GO:0019216	regulation of lipid metabolic process	5	42	72/3649	1.25e-03	1.25e-03	3.80e-02	4	*ADIPOQ; INSIG1; LDLR; NR1D1*	1	*PRL*	4/42	1/42	5/42
GO:0050728	negative regulation of inflammatory response	3	42	20/3649	1.41e-03	1.41e-03	4.01e-02	3	*ADIPOQ; LDLR; NR1D1*	0	*0*	3/42	0/42	3/42
GO:0062013	positive regulation of small molecule metabolic process	3	42	20/3649	1.41e-03	1.41e-03	4.01e-02	2	*ADIPOQ; NR1D1*	1	*PRL*	2/42	1/42	3/42
GO:0042180	cellular ketone metabolic process	4	42	46/3649	1.74e-03	1.74e-03	4.76e-02	3	*ADIPOQ; INSIG1; NR1D1*	1	*PRL*	3/42	1/42	4/42
GO:0051093	negative regulation of developmental process	6	42	115/3649	1.78e-03	1.78e-03	4.76e-02	4	*ADIPOQ; INSIG1; LDLR; NR1D1*	2	*ADRB2; ANGPT2*	4/42	2/42	6/42
GO:2000241	regulation of reproductive process	3	42	23/3649	2.14e-03	2.14e-03	5.54e-02	1	*RNASE10*	2	*GJA1; PRL*	1/42	2/42	3/42
GO:0001659	temperature homeostasis	3	42	26/3649	3.07e-03	3.07e-03	7.71e-02	1	*NR1D1*	2	*ADRB2; GJA1*	1/42	2/42	3/42
GO:0032102	negative regulation of response to external stimulus	4	42	54/3649	3.16e-03	3.16e-03	7.73e-02	3	*ADIPOQ; LDLR; NR1D1*	1	*ANGPT2*	3/42	1/42	4/42
GO:0015833	peptide transport	3	42	27/3649	3.42e-03	3.42e-03	7.93e-02	1	*NR1D1*	2	*GJA1; PRL*	1/42	2/42	3/42
GO:0046879	hormone secretion	3	42	27/3649	3.42e-03	3.42e-03	7.93e-02	1	*NR1D1*	2	*GJA1; PRL*	1/42	2/42	3/42
GO:0009653	anatomical structure morphogenesis	11	42	390/3649	3.60e-03	3.60e-03	8.11e-02	5	*ADIPOQ; ALCAM; INSIG1; LDLR; PRKCA*	6	*ANGPT2; FHL2; GJA1; HPGD; PRL; SERPINH1*	5/42	6/42	11/42
GO:0009914	hormone transport	3	42	28/3649	3.80e-03	3.80e-03	8.25e-02	1	*NR1D1*	2	*GJA1; PRL*	1/42	2/42	3/42
GO:0008202	steroid metabolic process	4	42	57/3649	3.85e-03	3.85e-03	8.25e-02	4	*INSIG1; LDLR; LSS; NR1D1*	0	*0*	4/42	0/42	4/42
GO:1901700	response to oxygen-containing compound	8	42	230/3649	4.01e-03	4.01e-03	8.25e-02	5	*ADIPOQ; LDLR; NR1D1; PRKCA; SCD*	3	*ADRB2; GJA1; PRL*	5/42	3/42	8/42
GO:0050793	regulation of developmental process	10	42	338/3649	4.03e-03	4.03e-03	8.25e-02	5	*ADIPOQ; INSIG1; LDLR; NR1D1; PRKCA*	5	*ADRB2; ANGPT2; GJA1; MAMSTR; PRL*	5/42	5/42	10/42
GO:0033559	unsaturated fatty acid metabolic process	3	42	29/3649	4.21e-03	4.21e-03	8.42e-02	1	*SCD*	2	*FADS3; HPGD*	1/42	2/42	3/42
GO:0051241	negative regulation of multicellular organismal process	6	42	138/3649	4.48e-03	4.48e-03	8.63e-02	3	*ADIPOQ; LDLR; NR1D1*	3	*ADRB2; ANGPT2; GJA1*	3/42	3/42	6/42
GO:0050727	regulation of inflammatory response	4	42	60/3649	4.64e-03	4.64e-03	8.63e-02	4	*ADIPOQ; FABP4; LDLR; NR1D1*	0	*0*	4/42	0/42	4/42
GO:0006694	steroid biosynthetic process	3	42	30/3649	4.64e-03	4.64e-03	8.63e-02	3	*INSIG1; LSS; NR1D1*	0	*0*	3/42	0/42	3/42
GO:0045598	regulation of fat cell differentiation	3	42	31/3649	5.10e-03	5.10e-03	8.63e-02	3	*ADIPOQ; INSIG1; NR1D1*	0	*0*	3/42	0/42	3/42
GO:0010629	negative regulation of gene expression	6	42	143/3649	5.33e-03	5.33e-03	8.63e-02	3	*ADIPOQ; FABP4; LDLR*	3	*EIF4EBP1; GJA1; PRL*	3/42	3/42	6/42
GO:0034381	plasma lipoprotein particle clearance	2	42	10/3649	5.49e-03	5.49e-03	8.63e-02	2	*ADIPOQ; LDLR*	0	*0*	2/42	0/42	2/42
GO:0042116	macrophage activation	2	42	10/3649	5.49e-03	5.49e-03	8.63e-02	2	*LDLR; NR1D1*	0	*0*	2/42	0/42	2/42
GO:0045599	negative regulation of fat cell differentiation	2	42	10/3649	5.49e-03	5.49e-03	8.63e-02	2	*ADIPOQ; INSIG1*	0	*0*	2/42	0/42	2/42
GO:0060079	excitatory postsynaptic potential	2	42	10/3649	5.49e-03	5.49e-03	8.63e-02	1	*CHRNA3*	1	*ADRB2*	1/42	1/42	2/42

GO:0060840	artery development	2	42	10/3649	5.49e-03	5.49e-03	8.63e-02	1	LDLR	1	HPGD	1/42	1/42	2/42
GO:0120162	positive regulation of cold-induced thermogenesis	2	42	10/3649	5.49e-03	5.49e-03	8.63e-02	0	0	2	ADRB2; GJA1	0/42	2/42	2/42
GO:0150076	neuroinflammatory response	2	42	10/3649	5.49e-03	5.49e-03	8.63e-02	2	LDLR; NR1D1	0	0	2/42	0/42	2/42
GO:0044262	cellular carbohydrate metabolic process	4	42	64/3649	5.85e-03	5.85e-03	9.03e-02	2	ADIPOQ; NR1D1	2	PFKFB1; PFKFB2	2/42	2/42	4/42
GO:0042886	amide transport	3	42	34/3649	6.62e-03	6.62e-03	9.61e-02	1	NR1D1	2	GJA1; PRL	1/42	2/42	3/42
GO:0042304	regulation of fatty acid biosynthetic process	2	42	11/3649	6.66e-03	6.66e-03	9.61e-02	1	INSIG1	1	PRL	1/42	1/42	2/42
GO:0060078	regulation of postsynaptic membrane potential	2	42	11/3649	6.66e-03	6.66e-03	9.61e-02	1	CHRNA3	1	ADRB2	1/42	1/42	2/42
GO:0099565	chemical synaptic transmission, postsynaptic	2	42	11/3649	6.66e-03	6.66e-03	9.61e-02	1	CHRNA3	1	ADRB2	1/42	1/42	2/42
GO:0045595	regulation of cell differentiation	7	42	199/3649	6.82e-03	6.82e-03	9.68e-02	5	ADIPOQ; INSIG1; LDLR; NR1D1; PRKCA	2	GJA1; MAMSTR	5/42	2/42	7/42
GO:0048468	cell development	8	42	253/3649	7.14e-03	7.14e-03	9.88e-02	4	ADIPOQ; ALCAM; LDLR; NR1D1	4	FHL2; GJA1; PRL; SERPINH1	4/42	4/42	8/42
GO:0043122	regulation of I-kappaB kinase/NF-kappaB signaling	3	42	35/3649	7.19e-03	7.19e-03	9.88e-02	2	ADIPOQ; NR1D1	1	GJA1	2/42	1/42	3/42
GO:0090317	negative regulation of intracellular protein transport	2	42	12/3649	7.94e-03	7.94e-03	1.07e-01	2	ADIPOQ; INSIG1	0	0	2/42	0/42	2/42
GO:0032101	regulation of response to external stimulus	6	42	156/3649	8.11e-03	8.11e-03	1.08e-01	5	ADIPOQ; FABP4; LDLR; NR1D1; PRKCA	1	ANGPT2	5/42	1/42	6/42
GO:0031348	negative regulation of defense response	3	42	37/3649	8.40e-03	8.40e-03	1.09e-01	3	ADIPOQ; LDLR; NR1D1	0	0	3/42	0/42	3/42
GO:0009719	response to endogenous stimulus	7	42	207/3649	8.42e-03	8.42e-03	1.09e-01	3	ADIPOQ; LDLR; NR1D1	4	ADRB2; FHL2; GJA1; HPGD	3/42	4/42	7/42
GO:0048514	blood vessel morphogenesis	5	42	112/3649	8.56e-03	8.56e-03	1.09e-01	2	LDLR; PRKCA	3	ANGPT2; GJA1; HPGD	2/42	3/42	5/42
GO:0048708	astrocyte differentiation	2	42	13/3649	9.31e-03	9.31e-03	1.16e-01	2	LDLR; NR1D1	0	0	2/42	0/42	2/42
GO:0010033	response to organic substance	11	42	442/3649	9.34e-03	9.34e-03	1.16e-01	5	ADIPOQ; LDLR; NR1D1; PRKCA; SCD	6	ACKR4; ADRB2; FHL2; GJA1; HPGD; PRL	5/42	6/42	11/42
GO:0007267	cell-cell signaling	6	42	162/3649	9.70e-03	9.70e-03	1.19e-01	3	ADIPOQ; CHRNA3; NR1D1	3	ADRB2; GJA1; PRL	3/42	3/42	6/42
GO:0030500	regulation of bone mineralization	2	42	14/3649	1.08e-02	1.08e-02	1.28e-01	0	0	2	ADRB2; GJA1	0/42	2/42	2/42
GO:0097006	regulation of plasma lipoprotein particle levels	2	42	14/3649	1.08e-02	1.08e-02	1.28e-01	2	ADIPOQ; LDLR	0	0	2/42	0/42	2/42
GO:0023057	negative regulation of signaling	7	42	219/3649	1.13e-02	1.13e-02	1.33e-01	3	ADIPOQ; LDLR; NR1D1	4	ADRB2; FHL2; GJA1; PRL	3/42	4/42	7/42
GO:0001501	skeletal system development	4	42	79/3649	1.22e-02	1.22e-02	1.36e-01	1	INSIG1	3	GJA1; SERPINH1; TRIM45	1/42	3/42	4/42
GO:0002695	negative regulation of leukocyte activation	2	42	15/3649	1.24e-02	1.24e-02	1.36e-01	2	LDLR; NR1D1	0	0	2/42	0/42	2/42
GO:0070167	regulation of biomineral tissue development	2	42	15/3649	1.24e-02	1.24e-02	1.36e-01	0	0	2	ADRB2; GJA1	0/42	2/42	2/42
GO:0110149	regulation of biomineralization	2	42	15/3649	1.24e-02	1.24e-02	1.36e-01	0	0	2	ADRB2; GJA1	0/42	2/42	2/42
GO:0044057	regulation of system process	4	42	81/3649	1.33e-02	1.33e-02	1.36e-01	2	ADIPOQ; PRKCA	2	ADRB2; GJA1	2/42	2/42	4/42
GO:0051094	positive regulation of developmental process	6	42	174/3649	1.35e-02	1.35e-02	1.36e-01	2	ADIPOQ; PRKCA	4	ADRB2; GJA1; MAMSTR; PRL	2/42	4/42	6/42
GO:0023061	signal release	3	42	44/3649	1.35e-02	1.35e-02	1.36e-01	1	NR1D1	2	GJA1; PRL	1/42	2/42	3/42
GO:0007589	body fluid secretion	2	42	16/3649	1.40e-02	1.40e-02	1.36e-01	0	0	2	GJA1; PRL	0/42	2/42	2/42
GO:0032387	negative regulation of intracellular transport	2	42	16/3649	1.40e-02	1.40e-02	1.36e-01	2	ADIPOQ; INSIG1	0	0	2/42	0/42	2/42
GO:0048659	smooth muscle cell proliferation	2	42	16/3649	1.40e-02	1.40e-02	1.36e-01	1	ADIPOQ	1	GJA1	1/42	1/42	2/42

GO:0048660	regulation of smooth muscle cell proliferation	2	42	16/3649	1.40e-02	1.40e-02	1.36e-01	1	*ADIPOQ*	1	*GJA1*	1/42	1/42	2/42
GO:0050768	negative regulation of neurogenesis	2	42	16/3649	1.40e-02	1.40e-02	1.36e-01	2	*LDLR; NR1D1*	0	*0*	2/42	0/42	2/42
GO:0051961	negative regulation of nervous system development	2	42	16/3649	1.40e-02	1.40e-02	1.36e-01	2	*LDLR; NR1D1*	0	*0*	2/42	0/42	2/42
GO:1901701	cellular response to oxygen-containing compound	6	42	176/3649	1.43e-02	1.43e-02	1.36e-01	4	*ADIPOQ; LDLR; NR1D1; PRKCA*	2	*ADRB2; GJA1*	4/42	2/42	6/42
GO:0009896	positive regulation of catabolic process	4	42	84/3649	1.50e-02	1.50e-02	1.36e-01	1	*LDLR*	3	*ADRB2; ATG101; GJA1*	1/42	3/42	4/42
GO:0007249	I-kappaB kinase/NF-kappaB signaling	3	42	46/3649	1.53e-02	1.53e-02	1.36e-01	2	*ADIPOQ; NR1D1*	1	*GJA1*	2/42	1/42	3/42
GO:0030336	negative regulation of cell migration	3	42	46/3649	1.53e-02	1.53e-02	1.36e-01	1	*ADIPOQ*	2	*ANGPT2; GJA1*	1/42	2/42	3/42
GO:0042391	regulation of membrane potential	3	42	46/3649	1.53e-02	1.53e-02	1.36e-01	1	*CHRNA3*	2	*ADRB2; GJA1*	1/42	2/42	3/42
GO:0045596	negative regulation of cell differentiation	4	42	85/3649	1.57e-02	1.57e-02	1.36e-01	4	*ADIPOQ; INSIG1; LDLR; NR1D1*	0	*0*	4/42	0/42	4/42
GO:0032879	regulation of localization	10	42	411/3649	1.57e-02	1.57e-02	1.36e-01	5	*ADIPOQ; INSIG1; NR1D1; PRKCA; RNASE10*	5	*ADRB2; ANGPT2; GJA1; PRL; TSPAN13*	5/42	5/42	10/42
GO:0001568	blood vessel development	5	42	130/3649	1.57e-02	1.57e-02	1.36e-01	2	*LDLR; PRKCA*	3	*ANGPT2; GJA1; HPGD*	2/42	3/42	5/42
GO:0002761	regulation of myeloid leukocyte differentiation	2	42	17/3649	1.58e-02	1.58e-02	1.36e-01	2	*ADIPOQ; PRKCA*	0	*0*	2/42	0/42	2/42
GO:0006636	unsaturated fatty acid biosynthetic process	2	42	17/3649	1.58e-02	1.58e-02	1.36e-01	1	*SCD*	1	*FADS3*	1/42	1/42	2/42
GO:0030879	mammary gland development	2	42	17/3649	1.58e-02	1.58e-02	1.36e-01	0	*0*	2	*GJA1; PRL*	0/42	2/42	2/42
GO:0046849	bone remodeling	2	42	17/3649	1.58e-02	1.58e-02	1.36e-01	0	*0*	2	*ADRB2; GJA1*	0/42	2/42	2/42
GO:0060349	bone morphogenesis	2	42	17/3649	1.58e-02	1.58e-02	1.36e-01	1	*INSIG1*	1	*SERPINH1*	1/42	1/42	2/42
GO:0072359	circulatory system development	6	42	180/3649	1.58e-02	1.58e-02	1.36e-01	2	*LDLR; PRKCA*	4	*ANGPT2; FHL2; GJA1; HPGD*	2/42	4/42	6/42
GO:0044242	cellular lipid catabolic process	3	42	47/3649	1.62e-02	1.62e-02	1.38e-01	2	*ADIPOQ; LDLR*	1	*CPT1B*	2/42	1/42	3/42
GO:2000146	negative regulation of cell motility	3	42	48/3649	1.71e-02	1.71e-02	1.41e-01	1	*ADIPOQ*	2	*ANGPT2; GJA1*	1/42	2/42	3/42
GO:0009605	response to external stimulus	10	42	418/3649	1.75e-02	1.75e-02	1.41e-01	6	*ADIPOQ; ALCAM; FABP4; LDLR; NR1D1; PRKCA*	4	*ADRB2; ANGPT2; GJA1; PRL*	6/42	4/42	10/42
GO:0007565	female pregnancy	2	42	18/3649	1.76e-02	1.76e-02	1.41e-01	0	*0*	2	*GJA1; HPGD*	0/42	2/42	2/42
GO:0032091	negative regulation of protein binding	2	42	18/3649	1.76e-02	1.76e-02	1.41e-01	1	*ADIPOQ*	1	*ADRB2*	1/42	1/42	2/42
GO:0032411	positive regulation of transporter activity	2	42	18/3649	1.76e-02	1.76e-02	1.41e-01	1	*ADIPOQ*	1	*ADRB2*	1/42	1/42	2/42
GO:0044703	multi-organism reproductive process	2	42	18/3649	1.76e-02	1.76e-02	1.41e-01	0	*0*	2	*GJA1; HPGD*	0/42	2/42	2/42
GO:0001944	vasculature development	5	42	134/3649	1.77e-02	1.77e-02	1.41e-01	2	*LDLR; PRKCA*	3	*ANGPT2; GJA1; HPGD*	2/42	3/42	5/42
GO:0040013	negative regulation of locomotion	3	42	49/3649	1.81e-02	1.81e-02	1.41e-01	1	*ADIPOQ*	2	*ANGPT2; GJA1*	1/42	2/42	3/42
GO:0051271	negative regulation of cellular component movement	3	42	49/3649	1.81e-02	1.81e-02	1.41e-01	1	*ADIPOQ*	2	*ANGPT2; GJA1*	1/42	2/42	3/42
GO:0031667	response to nutrient levels	3	42	50/3649	1.91e-02	1.91e-02	1.41e-01	1	*LDLR*	2	*ADRB2; PRL*	1/42	2/42	3/42
GO:1901653	cellular response to peptide	3	42	50/3649	1.91e-02	1.91e-02	1.41e-01	1	*ADIPOQ*	2	*ADRB2; GJA1*	1/42	2/42	3/42
GO:0031327	negative regulation of cellular biosynthetic process	6	42	188/3649	1.92e-02	1.92e-02	1.41e-01	4	*ADIPOQ; FABP4; INSIG1; NR1D1*	2	*EIF4EBP1; FHL2*	4/42	2/42	6/42
GO:0010243	response to organonitrogen compound	5	42	137/3649	1.93e-02	1.93e-02	1.41e-01	2	*ADIPOQ; LDLR*	3	*ADRB2; GJA1; PRL*	2/42	3/42	5/42
GO:0006635	fatty acid beta-oxidation	2	42	19/3649	1.95e-02	1.95e-02	1.41e-01	1	*ADIPOQ*	1	*CPT1B*	1/42	1/42	2/42
GO:0044706	multi-multicellular organism process	2	42	19/3649	1.95e-02	1.95e-02	1.41e-01	0	*0*	2	*GJA1; HPGD*	0/42	2/42	2/42

GO:0046883	regulation of hormone secretion	2	42	19/3649	1.95e-02	1.95e-02	1.41e-01	1	*NR1D1*	1	*GJA1*	1/42	1/42	2/42
GO:0050866	negative regulation of cell activation	2	42	19/3649	1.95e-02	1.95e-02	1.41e-01	2	*LDLR; NR1D1*	0	*0*	2/42	0/42	2/42
GO:0106106	cold-induced thermogenesis	2	42	19/3649	1.95e-02	1.95e-02	1.41e-01	0	*0*	2	*ADRB2; GJA1*	0/42	2/42	2/42
GO:0120161	regulation of cold-induced thermogenesis	2	42	19/3649	1.95e-02	1.95e-02	1.41e-01	0	*0*	2	*ADRB2; GJA1*	0/42	2/42	2/42
GO:0051049	regulation of transport	7	42	246/3649	2.05e-02	2.05e-02	1.46e-01	3	*ADIPOQ; INSIG1; NR1D1*	4	*ADRB2; GJA1; PRL; TSPAN13*	3/42	4/42	7/42
GO:0009746	response to hexose	2	42	20/3649	2.16e-02	2.16e-02	1.46e-01	2	*ADIPOQ; NR1D1*	0	*0*	2/42	0/42	2/42
GO:0009749	response to glucose	2	42	20/3649	2.16e-02	2.16e-02	1.46e-01	2	*ADIPOQ; NR1D1*	0	*0*	2/42	0/42	2/42
GO:0030278	regulation of ossification	2	42	20/3649	2.16e-02	2.16e-02	1.46e-01	0	*0*	2	*ADRB2; GJA1*	0/42	2/42	2/42
GO:0034284	response to monosaccharide	2	42	20/3649	2.16e-02	2.16e-02	1.46e-01	2	*ADIPOQ; NR1D1*	0	*0*	2/42	0/42	2/42
GO:0045931	positive regulation of mitotic cell cycle	2	42	20/3649	2.16e-02	2.16e-02	1.46e-01	1	*PRKCA*	1	*EIF4EBP1*	1/42	1/42	2/42
GO:0062014	negative regulation of small molecule metabolic process	2	42	20/3649	2.16e-02	2.16e-02	1.46e-01	2	*ADIPOQ; INSIG1*	0	*0*	2/42	0/42	2/42
GO:0098742	cell-cell adhesion via plasma-membrane adhesion molecules	2	42	20/3649	2.16e-02	2.16e-02	1.46e-01	2	*ADIPOQ; ALCAM*	0	*0*	2/42	0/42	2/42
GO:0035239	tube morphogenesis	5	42	143/3649	2.28e-02	2.28e-02	1.53e-01	2	*LDLR; PRKCA*	3	*ANGPT2; GJA1; HPGD*	2/42	3/42	5/42
GO:0019318	hexose metabolic process	3	42	54/3649	2.34e-02	2.34e-02	1.54e-01	1	*ADIPOQ*	2	*PFKFB1; PFKFB2*	1/42	2/42	3/42
GO:0009062	fatty acid catabolic process	2	42	21/3649	2.37e-02	2.37e-02	1.54e-01	1	*ADIPOQ*	1	*CPT1B*	1/42	1/42	2/42
GO:0009743	response to carbohydrate	2	42	21/3649	2.37e-02	2.37e-02	1.54e-01	2	*ADIPOQ; NR1D1*	0	*0*	2/42	0/42	2/42
GO:0009890	negative regulation of biosynthetic process	6	42	197/3649	2.37e-02	2.37e-02	1.54e-01	4	*ADIPOQ; FABP4; INSIG1; NR1D1*	2	*EIF4EBP1; FHL2*	4/42	2/42	6/42
GO:0009991	response to extracellular stimulus	3	42	55/3649	2.46e-02	2.46e-02	1.58e-01	1	*LDLR*	2	*ADRB2; PRL*	1/42	2/42	3/42
GO:0006897	endocytosis	4	42	98/3649	2.51e-02	2.51e-02	1.58e-01	1	*LDLR*	3	*ACKR4; ADRB2; PRL*	1/42	3/42	4/42
GO:0007186	G protein-coupled receptor signaling pathway	4	42	98/3649	2.51e-02	2.51e-02	1.58e-01	1	*PRKCA*	3	*ACKR4; ADRB2; HPGD*	1/42	3/42	4/42
GO:1902532	negative regulation of intracellular signal transduction	4	42	98/3649	2.51e-02	2.51e-02	1.58e-01	2	*ADIPOQ; NR1D1*	2	*FHL2; PRL*	2/42	2/42	4/42
GO:0002683	negative regulation of immune system process	3	42	56/3649	2.58e-02	2.58e-02	1.58e-01	3	*ADIPOQ; LDLR; NR1D1*	0	*0*	3/42	0/42	3/42
GO:0006869	lipid transport	3	42	56/3649	2.58e-02	2.58e-02	1.58e-01	3	*ADIPOQ; FABP4; LDLR*	0	*0*	3/42	0/42	3/42
GO:0008277	regulation of G protein-coupled receptor signaling pathway	2	42	22/3649	2.58e-02	2.58e-02	1.58e-01	1	*PRKCA*	1	*ADRB2*	1/42	1/42	2/42
GO:0021782	glial cell development	2	42	22/3649	2.58e-02	2.58e-02	1.58e-01	2	*LDLR; NR1D1*	0	*0*	2/42	0/42	2/42
GO:0030072	peptide hormone secretion	2	42	22/3649	2.58e-02	2.58e-02	1.58e-01	1	*NR1D1*	1	*PRL*	1/42	1/42	2/42
GO:0048585	negative regulation of response to stimulus	7	42	259/3649	2.64e-02	2.64e-02	1.58e-01	3	*ADIPOQ; LDLR; NR1D1*	4	*ADRB2; ANGPT2; FHL2; PRL*	3/42	4/42	7/42
GO:0060284	regulation of cell development	3	42	57/3649	2.70e-02	2.70e-02	1.58e-01	3	*ADIPOQ; LDLR; NR1D1*	0	*0*	3/42	0/42	3/42
GO:2000145	regulation of cell motility	5	42	150/3649	2.74e-02	2.74e-02	1.58e-01	3	*ADIPOQ; PRKCA; RNASE10*	2	*ANGPT2; GJA1*	3/42	2/42	5/42
GO:0042127	regulation of cell population proliferation	6	42	204/3649	2.76e-02	2.76e-02	1.58e-01	4	*ADIPOQ; FABP4; NR1D1; PRKCA*	2	*ATG101; GJA1*	4/42	2/42	6/42
GO:0048513	animal organ development	10	42	450/3649	2.81e-02	2.81e-02	1.58e-01	3	*ADIPOQ; INSIG1; PRKCA*	7	*ADRB2; ARID5B; FHL2; GJA1; PRL; SERPINH1; TRIM45*	3/42	7/42	10/42
GO:0002790	peptide secretion	2	42	23/3649	2.81e-02	2.81e-02	1.58e-01	1	*NR1D1*	1	*PRL*	1/42	1/42	2/42

GO:0003014	renal system process	2	42	23/3649	2.81e-02	2.81e-02	1.58e-01	2	*ADIPOQ; CHRNA3*	0	*0*	2/42	0/42	2/42
GO:0006641	triglyceride metabolic process	2	42	23/3649	2.81e-02	2.81e-02	1.58e-01	2	*INSIG1; LDLR*	0	*0*	2/42	0/42	2/42
GO:1990845	adaptive thermogenesis	2	42	23/3649	2.81e-02	2.81e-02	1.58e-01	0	*0*	2	*ADRB2; GJA1*	0/42	2/42	2/42
GO:0005996	monosaccharide metabolic process	3	42	58/3649	2.82e-02	2.82e-02	1.58e-01	1	*ADIPOQ*	2	*PFKFB1; PFKFB2*	1/42	2/42	3/42
GO:0007268	chemical synaptic transmission	3	42	58/3649	2.82e-02	2.82e-02	1.58e-01	2	*ADIPOQ; CHRNA3*	1	*ADRB2*	2/42	1/42	3/42
GO:0098916	anterograde trans-synaptic signaling	3	42	58/3649	2.82e-02	2.82e-02	1.58e-01	2	*ADIPOQ; CHRNA3*	1	*ADRB2*	2/42	1/42	3/42
GO:1901652	response to peptide	3	42	58/3649	2.82e-02	2.82e-02	1.58e-01	1	*ADIPOQ*	2	*ADRB2; GJA1*	1/42	2/42	3/42
GO:0030155	regulation of cell adhesion	4	42	102/3649	2.86e-02	2.86e-02	1.58e-01	3	*ADIPOQ; PRKCA; RNASE10*	1	*ANGPT2*	3/42	1/42	4/42
GO:0098609	cell-cell adhesion	4	42	102/3649	2.86e-02	2.86e-02	1.58e-01	4	*ADIPOQ; ALCAM; PRKCA; RNASE10*	0	*0*	4/42	0/42	4/42
GO:0031347	regulation of defense response	4	42	103/3649	2.95e-02	2.95e-02	1.60e-01	4	*ADIPOQ; FABP4; LDLR; NR1D1*	0	*0*	4/42	0/42	4/42
GO:0099537	trans-synaptic signaling	3	42	59/3649	2.95e-02	2.95e-02	1.60e-01	2	*ADIPOQ; CHRNA3*	1	*ADRB2*	2/42	1/42	3/42
GO:0040012	regulation of locomotion	5	42	154/3649	3.03e-02	3.03e-02	1.60e-01	3	*ADIPOQ; PRKCA; RNASE10*	2	*ANGPT2; GJA1*	3/42	2/42	5/42
GO:0007292	female gamete generation	2	42	24/3649	3.04e-02	3.04e-02	1.60e-01	0	*0*	2	*HPGD; PRL*	0/42	2/42	2/42
GO:0007369	gastrulation	2	42	24/3649	3.04e-02	3.04e-02	1.60e-01	1	*ADIPOQ*	1	*GJA1*	1/42	1/42	2/42
GO:0019395	fatty acid oxidation	2	42	24/3649	3.04e-02	3.04e-02	1.60e-01	1	*ADIPOQ*	1	*CPT1B*	1/42	1/42	2/42
GO:0051100	negative regulation of binding	2	42	24/3649	3.04e-02	3.04e-02	1.60e-01	1	*ADIPOQ*	1	*ADRB2*	1/42	1/42	2/42
GO:0072329	monocarboxylic acid catabolic process	2	42	24/3649	3.04e-02	3.04e-02	1.60e-01	1	*ADIPOQ*	1	*CPT1B*	1/42	1/42	2/42
GO:0010817	regulation of hormone levels	3	42	61/3649	3.22e-02	3.22e-02	1.63e-01	1	*NR1D1*	2	*GJA1; PRL*	1/42	2/42	3/42
GO:0050878	regulation of body fluid levels	3	42	61/3649	3.22e-02	3.22e-02	1.63e-01	1	*PRKCA*	2	*GJA1; PRL*	1/42	2/42	3/42
GO:0051270	regulation of cellular component movement	5	42	157/3649	3.25e-02	3.25e-02	1.63e-01	3	*ADIPOQ; PRKCA; RNASE10*	2	*ANGPT2; GJA1*	3/42	2/42	5/42
GO:0010508	positive regulation of autophagy	2	42	25/3649	3.28e-02	3.28e-02	1.63e-01	0	*0*	2	*ADRB2; ATG101*	0/42	2/42	2/42
GO:0010721	negative regulation of cell development	2	42	25/3649	3.28e-02	3.28e-02	1.63e-01	2	*LDLR; NR1D1*	0	*0*	2/42	0/42	2/42
GO:0033002	muscle cell proliferation	2	42	25/3649	3.28e-02	3.28e-02	1.63e-01	1	*ADIPOQ*	1	*GJA1*	1/42	1/42	2/42
GO:0034440	lipid oxidation	2	42	25/3649	3.28e-02	3.28e-02	1.63e-01	1	*ADIPOQ*	1	*CPT1B*	1/42	1/42	2/42
GO:0043123	positive regulation of I-kappaB kinase/NF-kappaB signaling	2	42	25/3649	3.28e-02	3.28e-02	1.63e-01	1	*ADIPOQ*	1	*GJA1*	1/42	1/42	2/42
GO:0046777	protein autophosphorylation	2	42	25/3649	3.28e-02	3.28e-02	1.63e-01	1	*ADIPOQ*	1	*ATG101*	1/42	1/42	2/42
GO:0048469	cell maturation	2	42	25/3649	3.28e-02	3.28e-02	1.63e-01	0	*0*	2	*GJA1; PRL*	0/42	2/42	2/42
GO:0022407	regulation of cell-cell adhesion	3	42	62/3649	3.35e-02	3.35e-02	1.65e-01	3	*ADIPOQ; PRKCA; RNASE10*	0	*0*	3/42	0/42	3/42
GO:0051051	negative regulation of transport	3	42	62/3649	3.35e-02	3.35e-02	1.65e-01	2	*ADIPOQ; INSIG1*	1	*GJA1*	2/42	1/42	3/42
GO:0042592	homeostatic process	7	42	273/3649	3.40e-02	3.40e-02	1.66e-01	5	*ADIPOQ; FABP4; INSIG1; LDLR; NR1D1*	2	*ADRB2; GJA1*	5/42	2/42	7/42
GO:1901698	response to nitrogen compound	5	42	159/3649	3.41e-02	3.41e-02	1.66e-01	2	*ADIPOQ; LDLR*	3	*ADRB2; GJA1; PRL*	2/42	3/42	5/42
GO:0016192	vesicle-mediated transport	7	42	274/3649	3.46e-02	3.46e-02	1.67e-01	4	*ADIPOQ; COG7; INSIG1; LDLR*	3	*ACKR4; ADRB2; PRL*	4/42	3/42	7/42
GO:0099536	synaptic signaling	3	42	63/3649	3.49e-02	3.49e-02	1.67e-01	2	*ADIPOQ; CHRNA3*	1	*ADRB2*	2/42	1/42	3/42
GO:1902533	positive regulation of intracellular signal transduction	5	42	160/3649	3.49e-02	3.49e-02	1.67e-01	2	*ADIPOQ; PRKCA*	3	*ADRB2; GJA1; PRL*	2/42	3/42	5/42
GO:0051224	negative regulation of protein transport	2	42	26/3649	3.53e-02	3.53e-02	1.68e-01	2	*ADIPOQ; INSIG1*	0	*0*	2/42	0/42	2/42

GO:0010648	negative regulation of cell communication	6	42	218/3649	3.66e-02	3.66e-02	1.73e-01	2	*ADIPOQ; NR1D1*	4	*ADRB2; FHL2; GJA1; PRL*	2/42	4/42	6/42
GO:0003008	system process	6	42	219/3649	3.73e-02	3.73e-02	1.75e-01	4	*ADIPOQ; CHRNA3; LDLR; PRKCA*	2	*ADRB2; GJA1*	4/42	2/42	6/42
GO:0015748	organophosphate ester transport	2	42	27/3649	3.79e-02	3.79e-02	1.75e-01	1	*LDLR*	1	*GJA1*	1/42	1/42	2/42
GO:0030282	bone mineralization	2	42	27/3649	3.79e-02	3.79e-02	1.75e-01	0	*0*	2	*ADRB2; GJA1*	0/42	2/42	2/42
GO:1904950	negative regulation of establishment of protein localization	2	42	27/3649	3.79e-02	3.79e-02	1.75e-01	2	*ADIPOQ; INSIG1*	0	*0*	2/42	0/42	2/42
GO:1902531	regulation of intracellular signal transduction	7	42	280/3649	3.83e-02	3.83e-02	1.77e-01	3	*ADIPOQ; NR1D1; PRKCA*	4	*ADRB2; FHL2; GJA1; PRL*	3/42	4/42	7/42
GO:0009894	regulation of catabolic process	5	42	165/3649	3.91e-02	3.91e-02	1.79e-01	1	*LDLR*	4	*ADRB2; ATG101; GJA1; HPGD*	1/42	4/42	5/42
GO:0019953	sexual reproduction	4	42	113/3649	3.95e-02	3.95e-02	1.80e-01	1	*RNASE10*	3	*GJA1; HPGD; PRL*	1/42	3/42	4/42
GO:0048771	tissue remodeling	2	42	28/3649	4.05e-02	4.05e-02	1.82e-01	0	*0*	2	*ADRB2; GJA1*	0/42	2/42	2/42
GO:0071310	cellular response to organic substance	8	42	346/3649	4.05e-02	4.05e-02	1.82e-01	4	*ADIPOQ; LDLR; NR1D1; PRKCA*	4	*ACKR4; ADRB2; GJA1; HPGD*	4/42	4/42	8/42
GO:0007610	behavior	3	42	67/3649	4.08e-02	4.08e-02	1.82e-01	3	*CHRNA3; LDLR; NR1D1*	0	*0*	3/42	0/42	3/42
GO:0048871	multicellular organismal homeostasis	3	42	67/3649	4.08e-02	4.08e-02	1.82e-01	1	*NR1D1*	2	*ADRB2; GJA1*	1/42	2/42	3/42
GO:0001503	ossification	3	42	68/3649	4.24e-02	4.24e-02	1.88e-01	0	*0*	3	*ADRB2; FHL2; GJA1*	0/42	3/42	3/42
GO:0001649	osteoblast differentiation	2	42	29/3649	4.32e-02	4.32e-02	1.88e-01	0	*0*	2	*FHL2; GJA1*	0/42	2/42	2/42
GO:0034764	positive regulation of transmembrane transport	2	42	29/3649	4.32e-02	4.32e-02	1.88e-01	1	*ADIPOQ*	1	*ADRB2*	1/42	1/42	2/42
GO:0050729	positive regulation of inflammatory response	2	42	29/3649	4.32e-02	4.32e-02	1.88e-01	2	*FABP4; LDLR*	0	*0*	2/42	0/42	2/42
GO:0071356	cellular response to tumor necrosis factor	2	42	29/3649	4.32e-02	4.32e-02	1.88e-01	2	*ADIPOQ; NR1D1*	0	*0*	2/42	0/42	2/42
GO:0035295	tube development	5	42	170/3649	4.36e-02	4.36e-02	1.89e-01	2	*LDLR; PRKCA*	3	*ANGPT2; GJA1; HPGD*	2/42	3/42	5/42
GO:0045597	positive regulation of cell differentiation	4	42	117/3649	4.40e-02	4.40e-02	1.90e-01	2	*ADIPOQ; PRKCA*	2	*GJA1; MAMSTR*	2/42	2/42	4/42
GO:0048646	anatomical structure formation involved in morphogenesis	5	42	172/3649	4.55e-02	4.55e-02	1.94e-01	1	*PRKCA*	4	*ANGPT2; FHL2; GJA1; PRL*	1/42	4/42	5/42
GO:0016042	lipid catabolic process	3	42	70/3649	4.56e-02	4.56e-02	1.94e-01	2	*ADIPOQ; LDLR*	1	*CPT1B*	2/42	1/42	3/42
GO:0008203	cholesterol metabolic process	2	42	30/3649	4.59e-02	4.59e-02	1.94e-01	2	*LDLR; LSS*	0	*0*	2/42	0/42	2/42
GO:0048705	skeletal system morphogenesis	2	42	30/3649	4.59e-02	4.59e-02	1.94e-01	1	*INSIG1*	1	*SERPINH1*	1/42	1/42	2/42
GO:0048598	embryonic morphogenesis	3	42	71/3649	4.72e-02	4.72e-02	1.98e-01	2	*ADIPOQ; INSIG1*	1	*GJA1*	2/42	1/42	3/42
GO:0051046	regulation of secretion	3	42	71/3649	4.72e-02	4.72e-02	1.98e-01	1	*NR1D1*	2	*GJA1; PRL*	1/42	2/42	3/42
GO:0035556	intracellular signal transduction	9	42	423/3649	4.75e-02	4.75e-02	1.98e-01	3	*ADIPOQ; NR1D1; PRKCA*	6	*ADRB2; EIF4EBP1; FHL2; GJA1; PRL; RHOF*	3/42	6/42	9/42
GO:0006355	regulation of transcription, DNA-templated	9	42	425/3649	4.87e-02	4.87e-02	1.99e-01	4	*ADIPOQ; CEBPG; FABP4; NR1D1*	5	*ADRB2; ARID5B; FHL2; MAMSTR; PRL*	4/42	5/42	9/42
GO:0002274	myeloid leukocyte activation	2	42	31/3649	4.87e-02	4.87e-02	1.99e-01	2	*LDLR; NR1D1*	0	*0*	2/42	0/42	2/42
GO:0006639	acylglycerol metabolic process	2	42	31/3649	4.87e-02	4.87e-02	1.99e-01	2	*INSIG1; LDLR*	0	*0*	2/42	0/42	2/42
GO:0016125	sterol metabolic process	2	42	31/3649	4.87e-02	4.87e-02	1.99e-01	2	*LDLR; LSS*	0	*0*	2/42	0/42	2/42
GO:0050767	regulation of neurogenesis	2	42	31/3649	4.87e-02	4.87e-02	1.99e-01	2	*LDLR; NR1D1*	0	*0*	2/42	0/42	2/42
GO:1903506	regulation of nucleic acid-templated transcription	9	42	426/3649	4.93e-02	4.93e-02	2.00e-01	4	*ADIPOQ; CEBPG; FABP4; NR1D1*	5	*ADRB2; ARID5B; FHL2; MAMSTR; PRL*	4/42	5/42	9/42
GO:0051726	regulation of cell cycle	5	42	176/3649	4.95e-02	4.95e-02	2.00e-01	1	*PRKCA*	4	*EIF4EBP1; HPGD; KLHL21; PRL*	1/42	4/42	5/42

3. Enriched Biological Process of Gene Ontology terms to DEG prospect in T2 vs T1 into conventional weaning group

ID	Description	count	terms	BgRatio	pvalue	p.adjust	qvalue	Count.UpReg	geneID.UpReg	Count.DownReg	geneID.DownReg	GeneRatio.Up	GeneRatio.Down	GeneRatio
GO:0009888	tissue development	40	210	276/3649	1.75e-08	1.75e-08	2.88e-05	29	*ACTB; ACTG1; ASAH1; CD44; CTSK; EDNRB; FLCN; FRZB; GJA1; GPR4; HEYL; ID3; IGFBP5; ISG15; LFNG; MSTN; MYOD1; MYOG; NPPC; NR4A1; PAK1; PTER; SCIN; SERPINH1; SNAI2; TGFB1; TGFB2; TMEM100; TUFT1*	11	*ACVR2A; C1GALT1; CITED2; CSRP3; EDN1; EDNRA; FGF1; KCNMA1; MGP; MYOZ2; RAPGEF2*	29/210	11/210	40/210
GO:0007166	cell surface receptor signaling pathway	45	210	373/3649	5.67e-07	5.67e-07	4.68e-04	30	*ANGPT2; CD14; CD44; CHRNE; CHRNG; EDNRB; FLCN; FRZB; HEYL; IFI6; IGFBP5; INHBB; IRF1; ISG15; LFNG; MSTN; MX1; NFKBIZ; NOS3; NPPC; OAS2; PAK1; RHBDF1; SH2D1A; SNAI2; TGFB1; TGFB2; TMEM100; WFIKKN2; ZC3H12A*	15	*ACVR2A; ARL6; CCRL2; CIBAR1; CITED2; CSRP3; CYLD; EDN1; EDNRA; FGF1; HSPB1; NCEH1; RAPGEF2; RNF34; WDR12*	30/210	15/210	45/210
GO:0050793	regulation of developmental process	41	210	338/3649	1.76e-06	1.76e-06	9.68e-04	29	*ANGPT2; CD44; CTSK; EDNRB; FLCN; FRZB; GAMT; GJA1; GPR4; HEYL; ID3; IGFBP5; IRF1; ISG15; LRRN1; MAMSTR; MSTN; MYOD1; MYOG; NFKBIZ; OAS2; SCIN; SNAI2; SPRY4; TGFB1; TGFB2; TMEM100; WARS1; ZC3H12A*	12	*ACVR2A; CITED2; EDN1; FGF1; FITM2; GLUL; HSPB1; MGP; NAPEPLD; RAPGEF2; RIOX1; THBS2*	29/210	12/210	41/210
GO:0051239	regulation of multicellular organismal process	45	210	393/3649	2.54e-06	2.54e-06	9.97e-04	32	*ANGPT2; APLN; CD14; CGAS; CTSK; EDNRB; FLCN; FRZB; GAMT; GJA1; GPR4; IGFBP5; INHBB; IRF1; ISG15; ITGB6; LRRN1; MYOD1; MYOG; NFKBIZ; NR4A2; OAS2; ORMDL3; OXTR; SCIN; SNAI2; TGFB1; TGFB2; TMEM100; TNNT2; WARS1; ZC3H12A*	13	*ACVR2A; CITED2; CSRP3; EDN1; EDNRA; FGF1; GLUL; HSPB1; KCNMA1; MAP2K6; MGP; RAPGEF2; THBS2*	32/210	13/210	45/210
GO:0071310	cellular response to organic substance	41	210	346/3649	3.26e-06	3.26e-06	9.97e-04	31	*ACTB; ACTG1; ASAH1; CD14; CD44; FLCN; GJA1; HEYL; ID3; IGFBP5; INHBB; IRF1; ISG15; MSTN; MX1; MYOD1; MYOG; NR4A1; NR4A2; NSG2; OAS2; PAK1; RBFOX2; RXRG; SNAI2; SPRY4; TGFB1; TGFB2; TMEM100; WFIKKN2; ZC3H12A*	10	*ACVR2A; CCRL2; CITED2; CSRP3; CYLD; EDN1; EDNRA; FGF1; HSPB1; RAPGEF2*	31/210	10/210	41/210
GO:0048513	animal organ development	49	210	450/3649	3.62e-06	3.62e-06	9.97e-04	36	*APLN; ASAH1; CNN2; CTSK; EDNRB; ETV6; FLCN; FRZB; GAMT; GJA1; GNAT1; GPR4; HEYL; ID3; IGFBP5; IRF1; ISG15; LFNG; MSTN; MYOD1; MYOG; NFKBIZ; NPPC; NR4A1; NR4A2; OAS2; SCIN; SERPINH1; SNAI2; SOBP; TGFB1; TGFB2; TMEM100; TRIM45; TUFT1; ZC3H12A*	14	*ACVR2A; C1GALT1; CITED2; CSRP3; ECE2; ECE2; EDN1; EDNRA; FGF1; KCNMA1; MAP2K6; MGP; MYOZ2; RAPGEF2*	36/210	14/210	49/210
GO:0060429	epithelium development	24	210	155/3649	5.43e-06	5.43e-06	1.28e-03	17	*ACTB; ACTG1; ASAH1; CD44; EDNRB; FRZB; GJA1; GPR4; HEYL; ID3; IGFBP5; LFNG; PAK1; PTER; TGFB1; TGFB2; TMEM100*	7	*C1GALT1; CITED2; EDN1; EDNRA; FGF1; KCNMA1; RAPGEF2*	17/210	7/210	24/210
GO:0060537	muscle tissue development	15	210	71/3649	7.87e-06	7.87e-06	1.62e-03	10	*FLCN; GJA1; HEYL; IGFBP5; MSTN; MYOD1; MYOG; NR4A1; TGFB1; TGFB2*	5	*CITED2; CSRP3; EDN1; EDNRA; MYOZ2*	10/210	5/210	15/210
GO:2000026	regulation of multicellular organismal development	26	210	182/3649	9.89e-06	9.89e-06	1.81e-03	18	*ANGPT2; CTSK; FRZB; GJA1; GPR4; IRF1; ISG15; LRRN1; MYOD1; NFKBIZ; OAS2; SCIN; SNAI2; TGFB1; TGFB2; TMEM100; WARS1; ZC3H12A*	8	*ACVR2A; EDN1; FGF1; GLUL; HSPB1; MGP; RAPGEF2; THBS2*	18/210	8/210	26/210
GO:0010033	response to organic substance	47	210	442/3649	1.18e-05	1.18e-05	1.94e-03	34	*ACTB; ACTG1; ASAH1; CD14; CD44; CXCL16; EDNRB; FLCN; GJA1;*	13	*ACVR2A; CCRL2; CITED2;*	34/210	13/210	47/210

									HEYL; ID3; IGFBP5; INHBB; IRF1; ISG15; MSTN; MX1; MYOD1; MYOG; NR4A1; NR4A2; NSG2; OAS2; PAK1; RBFOX2; RGS10; RXRG; SNAI2; SPRY4; TGFB1; TGFB2; TMEM100; WFIKKN2; ZC3H12A		*CSRP3; CYLD; EDN1; EDNRA; FGF1; HSPB1; KCNMA1; MAPKAPK3; RAPGEF2; USP13*			
GO:0007167	enzyme linked receptor protein signaling pathway	20	210	126/3649	2.39e-05	2.39e-05	3.58e-03	13	*ANGPT2; FLCN; IFI6; IGFBP5; INHBB; MSTN; NPPC; PAK1; RHBDF1; TGFB1; TGFB2; TMEM100; WFIKKN2*	7	*ACVR2A; CITED2; CSRP3; FGF1; HSPB1; NCEH1; RAPGEF2*	13/210	7/210	20/210
GO:0009653	anatomical structure morphogenesis	42	210	390/3649	2.83e-05	2.83e-05	3.89e-03	28	*ACTB; ACTG1; ANGPT2; APLN; CD44; FRZB; GAMT; GJA1; GNAT1; GPR4; HEYL; ID3; IGFBP5; LFNG; MYOG; NPPC; NR4A1; NR4A2; PAK1; SERPINH1; SOBP; SPRY4; STXBP1; TGFB1; TGFB2; TMEM100; WARS1; ZC3H12A*	14	*C1GALT1; CIBAR1; CITED2; CSRP3; EDN1; EDNRA; FGF1; FITM2; GLUL; HSPB1; MYOZ2; RAPGEF2; RFX2; THBS2*	28/210	14/210	42/210
GO:0001501	skeletal system development	15	210	79/3649	3.07e-05	3.07e-05	3.90e-03	10	*CTSK; FRZB; GJA1; NPPC; SCIN; SERPINH1; SNAI2; TGFB1; TRIM45; WFIKKN2*	5	*CITED2; EDN1; EDNRA; MAP2K6; MGP*	10/210	5/210	15/210
GO:0071495	cellular response to endogenous stimulus	25	210	184/3649	3.59e-05	3.59e-05	3.97e-03	18	*ACTB; FLCN; GJA1; HEYL; IGFBP5; INHBB; MSTN; NR4A1; NR4A2; NSG2; PAK1; RBFOX2; RXRG; SNAI2; TGFB1; TGFB2; TMEM100; WFIKKN2*	7	*ACVR2A; CITED2; CSRP3; EDN1; EDNRA; FGF1; RAPGEF2*	18/210	7/210	25/210
GO:0061061	muscle structure development	18	210	109/3649	3.60e-05	3.60e-05	3.97e-03	13	*ACTG1; EDNRB; GJA1; HEYL; ID3; IGFBP5; MAMSTR; MSTN; MYOD1; MYOG; NR4A1; TGFB1; WFIKKN2*	5	*CITED2; CSRP3; EDN1; EDNRA; MYOZ2*	13/210	5/210	18/210
GO:0044057	regulation of system process	15	210	81/3649	4.18e-05	4.18e-05	4.32e-03	11	*APLN; EDNRB; GJA1; IGFBP5; INHBB; MYOG; ORMDL3; TGFB2; TMEM100; TNNT2; ZC3H12A*	4	*CSRP3; EDN1; EDNRA; KCNMA1*	11/210	4/210	15/210
GO:0048469	cell maturation	8	210	25/3649	4.87e-05	4.87e-05	4.55e-03	5	*EDNRB; GJA1; NR4A2; PGRMC1; RND1*	3	*EDN1; EDNRA; KCNMA1*	5/210	3/210	8/210
GO:0045595	regulation of cell differentiation	26	210	199/3649	4.96e-05	4.96e-05	4.55e-03	21	*EDNRB; FLCN; FRZB; GJA1; HEYL; ID3; IGFBP5; IRF1; ISG15; MAMSTR; MSTN; MYOD1; MYOG; NFKBIZ; SCIN; SNAI2; SPRY4; TGFB1; TGFB2; TMEM100; ZC3H12A*	5	*ACVR2A; EDN1; NAPEPLD; RAPGEF2; RIOX1*	21/210	5/210	26/210
GO:0048514	blood vessel morphogenesis	18	210	112/3649	5.24e-05	5.24e-05	4.56e-03	9	*ACTG1; ANGPT2; APLN; GJA1; GPR4; NR4A1; TMEM100; WARS1; ZC3H12A*	9	*C1GALT1; CITED2; EDN1; EDNRA; FGF1; GLUL; HSPB1; RAPGEF2; THBS2*	9/210	9/210	18/210
GO:0048762	mesenchymal cell differentiation	10	210	40/3649	5.94e-05	5.94e-05	4.91e-03	7	*EDNRB; FRZB; HEYL; SNAI2; TGFB1; TGFB2; TMEM100*	3	*CITED2; EDN1; EDNRA*	7/210	3/210	10/210
GO:0045934	negative regulation of nucleobase-containing compound metabolic process	24	210	179/3649	6.49e-05	6.49e-05	5.10e-03	16	*BHLHE40; CCND1; CGAS; CXXC5; EDNRB; ETV6; FLCN; FOXP1; HEYL; ID3; IRF1; NR4A2; RBFOX2; SNAI2; TGFB1; ZC3H12A*	8	*CITED2; CRYAB; EDN1; MYOZ2; PRMT6; RIOX1; RNF168; RNF169*	16/210	8/210	24/210
GO:0009605	response to external stimulus	43	210	418/3649	6.88e-05	6.88e-05	5.16e-03	33	*ACTG1; ANGPT2; BHLHE40; C4A; CD14; CD40; CGAS; CNN2; CXCL16; EDNRB; FLCN; GJA1; GNAT1; GPR4; HMGB3; IFI6; INHBB; IRF1; ISG15; MACIR; MSTN; MX1; MX2; MYOD1; NFKBIZ; NR4A1; NR4A2; OAS2; S100A14; SH2D1A; SNAI2; TGFB1; ZC3H12A*	10	*ABCC1; CSRP3; CYLD; EDN1; EDNRA; FGF1; HSPB1; KCNMA1; MAPKAPK3; NAPEPLD*	33/210	10/210	43/210
GO:0035295	tube development	23	210	170/3649	8.07e-05	8.07e-05	5.43e-03	14	*ACTG1; ANGPT2; APLN; EDNRB; GJA1; GPR4; HEYL; IGFBP5; NR4A1; PAK1; TGFB2; TMEM100; WARS1; ZC3H12A*	9	*C1GALT1; CITED2; EDN1; EDNRA; FGF1; GLUL; HSPB1; RAPGEF2; THBS2*	14/210	9/210	23/210
GO:0045892	negative regulation of	21	210	148/3649	8.33e-05	8.33e-05	5.43e-03	14	*BHLHE40; CCND1; CXXC5; EDNRB; ETV6;*	7	*CITED2; CRYAB; EDN1;*	14/210	7/210	21/210

	transcription, DNA-templated								*FLCN; FOXP1; HEYL; ID3; IRF1; NR4A2; RBFOX2; SNAI2; TGFB1*		*MYOZ2; PRMT6; RIOX1; RNF168*			
GO:0071407	cellular response to organic cyclic compound	15	210	86/3649	8.64e-05	8.64e-05	5.43e-03	14	*ACTB; HEYL; ID3; IGFBP5; MSTN; MYOD1; MYOG; NSG2; PAK1; RBFOX2; RXRG; SNAI2; TGFB1; ZC3H12A*	1	*RAPGEF2*	14/210	1/210	15/210
GO:0051216	cartilage development	9	210	34/3649	8.66e-05	8.66e-05	5.43e-03	7	*CTSK; FRZB; NPPC; SCIN; SERPINH1; SNAI2; TGFB1*	2	*EDN1; MGP*	7/210	2/210	9/210
GO:1902679	negative regulation of RNA biosynthetic process	21	210	149/3649	9.21e-05	9.21e-05	5.43e-03	14	*BHLHE40; CCND1; CXXC5; EDNRB; ETV6; FLCN; FOXP1; HEYL; ID3; IRF1; NR4A2; RBFOX2; SNAI2; TGFB1*	7	*CITED2; CRYAB; EDN1; MYOZ2; PRMT6; RIOX1; RNF168*	14/210	7/210	21/210
GO:1903507	negative regulation of nucleic acid-templated transcription	21	210	149/3649	9.21e-05	9.21e-05	5.43e-03	14	*BHLHE40; CCND1; CXXC5; EDNRB; ETV6; FLCN; FOXP1; HEYL; ID3; IRF1; NR4A2; RBFOX2; SNAI2; TGFB1*	7	*CITED2; CRYAB; EDN1; MYOZ2; PRMT6; RIOX1; RNF168*	14/210	7/210	21/210
GO:0048646	anatomical structure formation involved in morphogenesis	23	210	172/3649	9.71e-05	9.71e-05	5.53e-03	12	*ACTG1; ANGPT2; APLN; GJA1; GPR4; HEYL; LFNG; MYOG; NR4A1; TMEM100; WARS1; ZC3H12A*	11	*C1GALT1; CITED2; CSRP3; EDN1; EDNRA; FGF1; GLUL; HSPB1; MYOZ2; RFX2; THBS2*	12/210	11/210	23/210
GO:0007517	muscle organ development	12	210	60/3649	1.16e-04	1.16e-04	6.37e-03	8	*GJA1; HEYL; ID3; MSTN; MYOD1; MYOG; NR4A1; TGFB1*	4	*CITED2; CSRP3; EDNRA; MYOZ2*	8/210	4/210	12/210
GO:0031324	negative regulation of cellular metabolic process	37	210	348/3649	1.22e-04	1.22e-04	6.52e-03	25	*BHLHE40; CCND1; CGAS; CXXC5; EDNRB; ETV6; FLCN; FOXP1; HEYL; ID3; IFI6; IGFBP5; IRF1; ISG15; MSTN; NR4A1; NR4A2; PKIG; RBFOX2; SNAI2; TGFB1; TGFB2; WARS1; WFIKKN2; ZC3H12A*	12	*BCL2; CITED2; CRYAB; EDN1; HSPB1; MYOZ2; PRMT6; RAPGEF2; RIOX1; RNF168; RNF169; RNF34*	25/210	12/210	37/210
GO:0021700	developmental maturation	10	210	44/3649	1.42e-04	1.42e-04	7.17e-03	7	*EDNRB; GJA1; NR4A2; PGRMC1; RND1; STXBP1; TGFB2*	3	*EDN1; EDNRA; KCNMA1*	7/210	3/210	10/210
GO:0031327	negative regulation of cellular biosynthetic process	24	210	188/3649	1.43e-04	1.43e-04	7.17e-03	16	*BHLHE40; CCND1; CXXC5; EDNRB; ETV6; FLCN; FOXP1; HEYL; ID3; IGFBP5; IRF1; NR4A2; RBFOX2; SNAI2; TGFB1; ZC3H12A*	8	*CITED2; CRYAB; EDN1; MYOZ2; PRMT6; RAPGEF2; RIOX1; RNF168*	16/210	8/210	24/210
GO:0003012	muscle system process	13	210	71/3649	1.55e-04	1.55e-04	7.55e-03	6	*EDNRB; IGFBP5; MYOG; ORMDL3; TNNT2; ZC3H12A*	7	*CSRP3; EDN1; EDNRA; KCNMA1; MAP2K6; MYOZ2; ROCK2*	6/210	7/210	13/210
GO:0014031	mesenchymal cell development	6	210	16/3649	1.67e-04	1.67e-04	7.67e-03	3	*EDNRB; HEYL; SNAI2*	3	*CITED2; EDN1; EDNRA*	3/210	3/210	6/210
GO:0061448	connective tissue development	10	210	45/3649	1.73e-04	1.73e-04	7.67e-03	8	*CTSK; FRZB; GPR4; NPPC; SCIN; SERPINH1; SNAI2; TGFB1*	2	*EDN1; MGP*	8/210	2/210	10/210
GO:0071695	anatomical structure maturation	9	210	37/3649	1.77e-04	1.77e-04	7.67e-03	6	*EDNRB; GJA1; NR4A2; PGRMC1; RND1; TGFB2*	3	*EDN1; EDNRA; KCNMA1*	6/210	3/210	9/210
GO:0071902	positive regulation of protein serine/threonine kinase activity	9	210	37/3649	1.77e-04	1.77e-04	7.67e-03	4	*CCND1; CD40; PAK1; TGFB1*	5	*CAB39; CKS2; EDN1; MAP2K6; RAPGEF2*	4/210	5/210	9/210
GO:0045860	positive regulation of protein kinase activity	12	210	63/3649	1.89e-04	1.89e-04	7.83e-03	6	*CCND1; CD40; MOB3C; PAK1; TGFB1; TGFB2*	6	*CAB39; CKS2; EDN1; FGF1; MAP2K6; RAPGEF2*	6/210	6/210	12/210
GO:0051253	negative regulation of RNA metabolic process	22	210	168/3649	1.90e-04	1.90e-04	7.83e-03	15	*BHLHE40; CCND1; CXXC5; EDNRB; ETV6; FLCN; FOXP1; HEYL; ID3; IRF1; NR4A2; RBFOX2; SNAI2; TGFB1; ZC3H12A*	7	*CITED2; CRYAB; EDN1; MYOZ2; PRMT6; RIOX1; RNF168*	15/210	7/210	22/210
GO:0072359	circulatory system development	23	210	180/3649	1.96e-04	1.96e-04	7.90e-03	12	*ACTG1; ANGPT2; APLN; GJA1; GPR4; HEYL; ID3; NR4A1; TGFB2; TMEM100; WARS1; ZC3H12A*	12	*C1GALT1; CITED2; CSRP3; ECE2; ECE2; EDN1; EDNRA; FGF1; GLUL; HSPB1; RAPGEF2; THBS2*	12/210	12/210	23/210
GO:1901861	regulation of muscle tissue development	5	210	11/3649	2.09e-04	2.09e-04	8.01e-03	5	*FLCN; GJA1; IGFBP5; MYOG; TGFB1*	0	*0*	5/210	0/210	5/210

GO:0060485	mesenchyme development	10	210	46/3649	2.11e-04	2.11e-04	8.01e-03	7	*EDNRB; FRZB; HEYL; SNAI2; TGFB1; TGFB2; TMEM100*	3	*CITED2; EDN1; EDNRA*	7/210	3/210	10/210
GO:0010558	negative regulation of macromolecule biosynthetic process	23	210	181/3649	2.13e-04	2.13e-04	8.01e-03	16	*BHLHE40; CCND1; CXXC5; EDNRB; ETV6; FLCN; FOXP1; HEYL; ID3; IGFBP5; IRF1; NR4A2; RBFOX2; SNAI2; TGFB1; ZC3H12A*	7	*CITED2; CRYAB; EDN1; MYOZ2; PRMT6; RIOX1; RNF168*	16/210	7/210	23/210
GO:0014070	response to organic cyclic compound	16	210	104/3649	2.35e-04	2.35e-04	8.61e-03	15	*ACTB; HEYL; ID3; IGFBP5; MSTN; MYOD1; MYOG; NSG2; PAK1; RBFOX2; RXRG; SNAI2; TGFB1; TGFB2; ZC3H12A*	1	*RAPGEF2*	15/210	1/210	16/210
GO:0051093	negative regulation of developmental process	17	210	115/3649	2.46e-04	2.46e-04	8.73e-03	14	*ANGPT2; CTSK; EDNRB; FLCN; FRZB; GPR4; ID3; IGFBP5; IRF1; MSTN; SNAI2; SPRY4; TGFB1; ZC3H12A*	3	*RAPGEF2; RIOX1; THBS2*	14/210	3/210	17/210
GO:0003008	system process	26	210	219/3649	2.51e-04	2.51e-04	8.73e-03	18	*APLN; CHRNE; CHRNG; EDNRB; GJA1; GNAT1; GPR4; IGFBP5; INHBB; MYOG; ORMDL3; OXTR; SNAI2; SOBP; TGFB2; TMEM100; TNNT2; ZC3H12A*	8	*ABCC1; CSRP3; EDN1; EDNRA; KCNMA1; MAP2K6; MYOZ2; ROCK2*	18/210	8/210	26/210
GO:0009719	response to endogenous stimulus	25	210	207/3649	2.54e-04	2.54e-04	8.73e-03	18	*ACTB; FLCN; GJA1; HEYL; IGFBP5; INHBB; MSTN; NR4A1; NR4A2; NSG2; PAK1; RBFOX2; RXRG; SNAI2; TGFB1; TGFB2; TMEM100; WFIKKN2*	7	*ACVR2A; CITED2; CSRP3; EDN1; EDNRA; FGF1; RAPGEF2*	18/210	7/210	25/210
GO:0009890	negative regulation of biosynthetic process	24	210	197/3649	2.97e-04	2.97e-04	1.00e-02	16	*BHLHE40; CCND1; CXXC5; EDNRB; ETV6; FLCN; FOXP1; HEYL; ID3; IGFBP5; IRF1; NR4A2; RBFOX2; SNAI2; TGFB1; ZC3H12A*	8	*CITED2; CRYAB; EDN1; MYOZ2; PRMT6; RAPGEF2; RIOX1; RNF168*	16/210	8/210	24/210
GO:0045597	positive regulation of cell differentiation	17	210	117/3649	3.04e-04	3.04e-04	1.00e-02	13	*FRZB; GJA1; HEYL; ISG15; MAMSTR; MYOD1; MYOG; NFKBIZ; SCIN; TGFB1; TGFB2; TMEM100; ZC3H12A*	4	*ACVR2A; EDN1; NAPEPLD; RAPGEF2*	13/210	4/210	17/210
GO:0051172	negative regulation of nitrogen compound metabolic process	34	210	324/3649	3.10e-04	3.10e-04	1.00e-02	24	*BHLHE40; CCND1; CGAS; CXXC5; EDNRB; ETV6; FLCN; FOXP1; HEYL; ID3; IFI6; IGFBP5; INHBB; IRF1; ISG15; NR4A1; NR4A2; PKIG; RBFOX2; SNAI2; TGFB1; WARS1; WFIKKN2; ZC3H12A*	10	*CITED2; CRYAB; EDN1; HSPB1; MYOZ2; PRMT6; RIOX1; RNF168; RNF169; RNF34*	24/210	10/210	34/210
GO:0001525	angiogenesis	15	210	97/3649	3.48e-04	3.48e-04	1.11e-02	8	*ACTG1; ANGPT2; APLN; GPR4; NR4A1; TMEM100; WARS1; ZC3H12A*	7	*C1GALT1; EDN1; EDNRA; FGF1; GLUL; HSPB1; THBS2*	8/210	7/210	15/210
GO:0008285	negative regulation of cell population proliferation	14	210	87/3649	3.60e-04	3.60e-04	1.12e-02	13	*ATG101; FLCN; FRZB; GJA1; IGFBP5; IRF1; MSTN; MYOG; PAK1; SCIN; SNAI2; TGFB1; TGFB2*	1	*RAPGEF2*	13/210	1/210	14/210
GO:0001568	blood vessel development	18	210	130/3649	3.73e-04	3.73e-04	1.14e-02	9	*ACTG1; ANGPT2; APLN; GJA1; GPR4; NR4A1; TMEM100; WARS1; ZC3H12A*	9	*C1GALT1; CITED2; EDN1; EDNRA; FGF1; GLUL; HSPB1; RAPGEF2; THBS2*	9/210	9/210	18/210
GO:0009725	response to hormone	15	210	98/3649	3.90e-04	3.90e-04	1.17e-02	11	*HEYL; IGFBP5; INHBB; MSTN; NR4A1; NR4A2; PAK1; RBFOX2; RXRG; TGFB1; TGFB2*	4	*CITED2; CSRP3; EDN1; EDNRA*	11/210	4/210	15/210
GO:0001503	ossification	12	210	68/3649	4.00e-04	4.00e-04	1.18e-02	8	*GJA1; ID3; IGFBP5; ISG15; NPPC; SNAI2; TGFB1; TGFB2*	4	*ACVR2A; MAP2K6; MGP; RIOX1*	8/210	4/210	12/210
GO:0008016	regulation of heart contraction	8	210	33/3649	4.18e-04	4.18e-04	1.21e-02	5	*APLN; EDNRB; GJA1; TGFB2; ZC3H12A*	3	*CSRP3; EDN1; EDNRA*	5/210	3/210	8/210
GO:0035239	tube morphogenesis	19	210	143/3649	4.38e-04	4.38e-04	1.25e-02	10	*ACTG1; ANGPT2; APLN; GJA1; GPR4; NR4A1; PAK1; TMEM100; WARS1; ZC3H12A*	9	*C1GALT1; CITED2; EDN1; EDNRA; FGF1; GLUL; HSPB1; RAPGEF2; THBS2*	10/210	9/210	19/210
GO:0001837	epithelial to mesenchymal transition	7	210	26/3649	4.86e-04	4.86e-04	1.32e-02	5	*HEYL; SNAI2; TGFB1; TGFB2; TMEM100*	2	*EDN1; EDNRA*	5/210	2/210	7/210

GO:0045216	cell-cell junction organization	7	210	26/3649	4.86e-04	4.86e-04	1.32e-02	7	*ACTB; ACTG1; FLCN; GJA1; SNAI2; TGFB1; TGFB2*	0	*0*	7/210	0/210	7/210
GO:0014033	neural crest cell differentiation	6	210	19/3649	4.88e-04	4.88e-04	1.32e-02	3	*EDNRB; FRZB; SNAI2*	3	*CITED2; EDN1; EDNRA*	3/210	3/210	6/210
GO:0007162	negative regulation of cell adhesion	8	210	34/3649	5.20e-04	5.20e-04	1.39e-02	8	*ANGPT2; IRF1; NOS3; RND1; SNAI2; SPRY4; TGFB1; ZC3H12A*	0	*0*	8/210	0/210	8/210
GO:0001944	vasculature development	18	210	134/3649	5.44e-04	5.44e-04	1.43e-02	9	*ACTG1; ANGPT2; APLN; GJA1; GPR4; NR4A1; TMEM100; WARS1; ZC3H12A*	9	*C1GALT1; CITED2; EDN1; EDNRA; FGF1; GLUL; HSPB1; RAPGEF2; THBS2*	9/210	9/210	18/210
GO:0010648	negative regulation of cell communication	25	210	218/3649	5.67e-04	5.67e-04	1.46e-02	21	*APLN; FLCN; FRZB; GJA1; GRINA; HEYL; IFI6; IGFBP5; INHBB; ISG15; LFNG; MSTN; NOS3; NR4A2; RASA3; RGS10; RGS19; SH2D1A; SNAI2; SPRY4; ZC3H12A*	4	*CYLD; HSPB1; MYOZ2; RNF34*	21/210	4/210	25/210
GO:0060047	heart contraction	9	210	43/3649	5.92e-04	5.92e-04	1.50e-02	5	*APLN; EDNRB; GJA1; TGFB2; ZC3H12A*	4	*CSRP3; EDN1; EDNRA; MAP2K6*	5/210	4/210	9/210
GO:0007178	transmembrane receptor protein serine/threonine kinase signaling pathway	10	210	52/3649	6.03e-04	6.03e-04	1.50e-02	7	*FLCN; INHBB; MSTN; TGFB1; TGFB2; TMEM100; WFIKKN2*	3	*ACVR2A; CITED2; NCEH1*	7/210	3/210	10/210
GO:0023057	negative regulation of signaling	25	210	219/3649	6.08e-04	6.08e-04	1.50e-02	21	*APLN; FLCN; FRZB; GJA1; GRINA; HEYL; IFI6; IGFBP5; INHBB; ISG15; LFNG; MSTN; NOS3; NR4A2; RASA3; RGS10; RGS19; SH2D1A; SNAI2; SPRY4; ZC3H12A*	4	*CYLD; HSPB1; MYOZ2; RNF34*	21/210	4/210	25/210
GO:0071363	cellular response to growth factor stimulus	14	210	92/3649	6.49e-04	6.49e-04	1.56e-02	9	*FLCN; HEYL; MSTN; NR4A1; SNAI2; TGFB1; TGFB2; TMEM100; WFIKKN2*	5	*ACVR2A; CITED2; FGF1; HSPB1; RAPGEF2*	9/210	5/210	14/210
GO:0006939	smooth muscle contraction	6	210	20/3649	6.63e-04	6.63e-04	1.56e-02	2	*EDNRB; ORMDL3*	4	*EDN1; EDNRA; KCNMA1; ROCK2*	2/210	4/210	6/210
GO:0030278	regulation of ossification	6	210	20/3649	6.63e-04	6.63e-04	1.56e-02	4	*GJA1; ISG15; TGFB1; TGFB2*	2	*ACVR2A; MGP*	4/210	2/210	6/210
GO:0009887	animal organ morphogenesis	17	210	125/3649	6.72e-04	6.72e-04	1.56e-02	13	*FRZB; GAMT; GJA1; GNAT1; HEYL; ID3; IGFBP5; NPPC; SERPINH1; SOBP; TGFB1; TGFB2; TMEM100*	4	*CITED2; EDN1; EDNRA; FGF1*	13/210	4/210	17/210
GO:0003015	heart process	9	210	44/3649	7.07e-04	7.07e-04	1.62e-02	5	*APLN; EDNRB; GJA1; TGFB2; ZC3H12A*	4	*CSRP3; EDN1; EDNRA; MAP2K6*	5/210	4/210	9/210
GO:0009892	negative regulation of metabolic process	41	210	436/3649	7.47e-04	7.47e-04	1.68e-02	27	*BHLHE40; CCND1; CGAS; CXXC5; EDNRB; ETV6; FLCN; FOXP1; GJA1; HEYL; ID3; IFI6; IGFBP5; INHBB; IRF1; ISG15; MSTN; NR4A1; NR4A2; PKIG; RBFOX2; SNAI2; TGFB1; TGFB2; WARS1; WFIKKN2; ZC3H12A*	14	*BCL2; CITED2; CRYAB; EDN1; FASTKD3; HSPB1; MAP2K6; MYOZ2; PRMT6; RAPGEF2; RIOX1; RNF168; RNF169; RNF34*	27/210	14/210	41/210
GO:0003013	circulatory system process	13	210	83/3649	7.64e-04	7.64e-04	1.68e-02	7	*APLN; EDNRB; GJA1; GPR4; OXTR; TGFB2; ZC3H12A*	6	*ABCC1; CSRP3; EDN1; EDNRA; KCNMA1; MAP2K6*	7/210	6/210	13/210
GO:0048729	tissue morphogenesis	13	210	83/3649	7.64e-04	7.64e-04	1.68e-02	9	*ACTB; ACTG1; CD44; FRZB; GJA1; HEYL; IGFBP5; PAK1; TMEM100*	4	*CITED2; EDN1; EDNRA; FGF1*	9/210	4/210	13/210
GO:0033674	positive regulation of kinase activity	12	210	73/3649	7.81e-04	7.81e-04	1.69e-02	6	*CCND1; CD40; MOB3C; PAK1; TGFB1; TGFB2*	6	*CAB39; CKS2; EDN1; FGF1; MAP2K6; RAPGEF2*	6/210	6/210	12/210
GO:0030500	regulation of bone mineralization	5	210	14/3649	7.86e-04	7.86e-04	1.69e-02	3	*GJA1; ISG15; TGFB1*	2	*ACVR2A; MGP*	3/210	2/210	5/210
GO:0051094	positive regulation of developmental process	21	210	174/3649	8.18e-04	8.18e-04	1.73e-02	14	*FRZB; GJA1; HEYL; ISG15; LRRN1; MAMSTR; MYOD1; MYOG; NFKBIZ; SCIN; TGFB1; TGFB2; TMEM100; ZC3H12A*	7	*ACVR2A; CITED2; EDN1; FGF1; HSPB1; NAPEPLD; RAPGEF2*	14/210	7/210	21/210
GO:0002009	morphogenesis of an epithelium	11	210	64/3649	8.79e-04	8.79e-04	1.84e-02	7	*ACTB; ACTG1; CD44; FRZB; GJA1; IGFBP5; PAK1*	4	*CITED2; EDN1; EDNRA; FGF1*	7/210	4/210	11/210

GO:0070887	cellular response to chemical stimulus	44	210	483/3649	9.19e-04	9.19e-04	1.90e-02	33	*ACTB; ACTG1; ASAH1; CD14; CD44; EDNRB; FLCN; GJA1; HEYL; ID3; IGFBP5; INHBB; IRF1; ISG15; MSTN; MX1; MYOD1; MYOG; NR4A1; NR4A2; NSG2; OAS2; PAK1; RBFOX2; RGS10; RXRG; SNAI2; SPRY4; TGFB1; TGFB2; TMEM100; WFIKKN2; ZC3H12A*	11	*ACVR2A; CCRL2; CITED2; CSRP3; CYLD; EDN1; EDNRA; FGF1; HSPB1; NCEH1; RAPGEF2*	33/210	11/210	44/210
GO:0007519	skeletal muscle tissue development	8	210	37/3649	9.53e-04	9.53e-04	1.92e-02	6	*HEYL; MSTN; MYOD1; MYOG; NR4A1; TGFB1*	2	*CITED2; MYOZ2*	6/210	2/210	8/210
GO:0060538	skeletal muscle organ development	8	210	37/3649	9.53e-04	9.53e-04	1.92e-02	6	*HEYL; MSTN; MYOD1; MYOG; NR4A1; TGFB1*	2	*CITED2; MYOZ2*	6/210	2/210	8/210
GO:0045596	negative regulation of cell differentiation	13	210	85/3649	9.63e-04	9.63e-04	1.92e-02	11	*EDNRB; FLCN; FRZB; ID3; IGFBP5; IRF1; MSTN; SNAI2; SPRY4; TGFB1; ZC3H12A*	2	*RAPGEF2; RIOX1*	11/210	2/210	13/210
GO:0001649	osteoblast differentiation	7	210	29/3649	9.95e-04	9.95e-04	1.95e-02	4	*GJA1; ID3; IGFBP5; SNAI2*	3	*ACVR2A; MAP2K6; RIOX1*	4/210	3/210	7/210
GO:0042692	muscle cell differentiation	11	210	65/3649	1.01e-03	1.01e-03	1.95e-02	7	*ACTG1; EDNRB; IGFBP5; MAMSTR; MYOD1; MYOG; WFIKKN2*	4	*CSRP3; EDN1; EDNRA; MYOZ2*	7/210	4/210	11/210
GO:0048468	cell development	27	210	253/3649	1.06e-03	1.06e-03	2.02e-02	18	*ACTG1; ATAT1; EDNRB; FRZB; GJA1; GNAT1; GPR4; HEYL; MYOG; NR4A2; PGRMC1; RND1; SERPINH1; SNAI2; SPRY4; STXBP1; TGFB1; WFIKKN2*	9	*C1GALT1; CITED2; CSRP3; EDN1; EDNRA; KCNMA1; MYOZ2; RAPGEF2; RFX2*	18/210	9/210	27/210
GO:0030334	regulation of cell migration	18	210	142/3649	1.10e-03	1.10e-03	2.02e-02	12	*ACTG1; ANGPT2; CNN2; FLCN; GJA1; IGFBP5; MSTN; PAK1; SNAI2; TGFB1; TGFB2; ZC3H12A*	6	*CITED2; EDN1; FGF1; GLUL; HSPB1; RAPGEF2*	12/210	6/210	18/210
GO:0070848	response to growth factor	14	210	97/3649	1.11e-03	1.11e-03	2.02e-02	9	*FLCN; HEYL; MSTN; NR4A1; SNAI2; TGFB1; TGFB2; TMEM100; WFIKKN2*	5	*ACVR2A; CITED2; FGF1; HSPB1; RAPGEF2*	9/210	5/210	14/210
GO:0014032	neural crest cell development	5	210	15/3649	1.12e-03	1.12e-03	2.02e-02	2	*EDNRB; SNAI2*	3	*CITED2; EDN1; EDNRA*	2/210	3/210	5/210
GO:0043406	positive regulation of MAP kinase activity	5	210	15/3649	1.12e-03	1.12e-03	2.02e-02	3	*CD40; PAK1; TGFB1*	2	*EDN1; MAP2K6*	3/210	2/210	5/210
GO:0070167	regulation of biomineral tissue development	5	210	15/3649	1.12e-03	1.12e-03	2.02e-02	3	*GJA1; ISG15; TGFB1*	2	*ACVR2A; MGP*	3/210	2/210	5/210
GO:0110149	regulation of biomineralization	5	210	15/3649	1.12e-03	1.12e-03	2.02e-02	3	*GJA1; ISG15; TGFB1*	2	*ACVR2A; MGP*	3/210	2/210	5/210
GO:0045859	regulation of protein kinase activity	15	210	109/3649	1.23e-03	1.23e-03	2.19e-02	8	*CCND1; CD40; MOB3C; PAK1; PKIG; TGFB1; TGFB2; WARS1*	7	*CAB39; CKS2; EDN1; FGF1; HSPB1; MAP2K6; RAPGEF2*	8/210	7/210	15/210
GO:0001667	ameboidal-type cell migration	12	210	77/3649	1.27e-03	1.27e-03	2.24e-02	7	*ANGPT2; EDNRB; GJA1; NR4A1; TGFB1; TGFB2; ZC3H12A*	5	*EDN1; EDNRA; FGF1; GLUL; HSPB1*	7/210	5/210	12/210
GO:0016477	cell migration	24	210	218/3649	1.32e-03	1.32e-03	2.29e-02	17	*ACTG1; ANGPT2; CD44; CNN2; EDNRB; FLCN; GJA1; IGFBP5; MSTN; NR4A1; NR4A2; PAK1; RHBDF1; SNAI2; TGFB1; TGFB2; ZC3H12A*	7	*CITED2; EDN1; EDNRA; FGF1; GLUL; HSPB1; RAPGEF2*	17/210	7/210	24/210
GO:0045444	fat cell differentiation	9	210	48/3649	1.37e-03	1.37e-03	2.34e-02	8	*ARL4A; FLCN; FRZB; INHBB; NR4A1; NR4A2; TGFB1; ZC3H12A*	1	*NAPEPLD*	8/210	1/210	9/210
GO:1903522	regulation of blood circulation	8	210	39/3649	1.37e-03	1.37e-03	2.34e-02	5	*APLN; EDNRB; GJA1; TGFB2; ZC3H12A*	3	*CSRP3; EDN1; EDNRA*	5/210	3/210	8/210
GO:0008015	blood circulation	12	210	78/3649	1.43e-03	1.43e-03	2.40e-02	7	*APLN; EDNRB; GJA1; GPR4; OXTR; TGFB2; ZC3H12A*	5	*CSRP3; EDN1; EDNRA; KCNMA1; MAP2K6*	7/210	5/210	12/210
GO:0012501	programmed cell death	35	210	366/3649	1.44e-03	1.44e-03	2.40e-02	23	*ASAH1; BLCAP; ETV6; FLCN; FRZB; GJA1; GRINA; IFI6; IRF1; MFSD10; NOS3; NR4A1; NR4A2; ORMDL3; PAK1; S100A14; SCIN; SHISA5; SNAI2; TGFB1; TGFB2; XAF1; ZC3H12A*	12	*BCL2; CITED2; CRYAB; CYLD; EDNRA; HSPB1; IFT57; KCNMA1; MAP2K6; RAPGEF2; RNF144B; RNF34*	23/210	12/210	35/210

GO:0048585	negative regulation of response to stimulus	27	210	259/3649	1.51e-03	1.51e-03	2.47e-02	22	*ANGPT2; APLN; CGAS; FLCN; FRZB; GRINA; HEYL; IFI6; IGFBP5; ISG15; LFNG; MACIR; MSTN; NOS3; NR4A2; RASA3; RGS10; RGS19; SH2D1A; SNAI2; SPRY4; ZC3H12A*	5	*CYLD; HSPB1; MYOZ2; RNF169; RNF34*	22/210	5/210	27/210
GO:0010605	negative regulation of macromolecule metabolic process	37	210	395/3649	1.52e-03	1.52e-03	2.47e-02	26	*BHLHE40; CCND1; CGAS; CXXC5; EDNRB; ETV6; FLCN; FOXP1; GJA1; HEYL; ID3; IFI6; IGFBP5; INHBB; IRF1; ISG15; NR4A1; NR4A2; PKIG; RBFOX2; SNAI2; TGFB1; TGFB2; WARS1; WFIKKN2; ZC3H12A*	11	*CITED2; CRYAB; EDN1; FASTKD3; HSPB1; MYOZ2; PRMT6; RIOX1; RNF168; RNF169; RNF34*	26/210	11/210	37/210
GO:0009966	regulation of signal transduction	41	210	452/3649	1.55e-03	1.55e-03	2.47e-02	29	*APLN; CD40; FLCN; FRZB; GJA1; GRINA; HEYL; IFI6; IGFBP5; IRF1; ISG15; LFNG; MSTN; NOS3; NR4A2; PAK1; RASA3; RELL2; RGS10; RGS19; RHBDF1; SH2D1A; SNAI2; SPRY4; TGFB1; TGFB2; TMEM100; WFIKKN2; ZC3H12A*	12	*ACVR2A; CIBAR1; CITED2; CYLD; EDN1; FGF1; HCRTR1; HSPB1; MAP2K6; MYOZ2; RAPGEF2; RNF34*	29/210	12/210	41/210
GO:0045445	myoblast differentiation	5	210	16/3649	1.56e-03	1.56e-03	2.47e-02	5	*ID3; MSTN; MYOD1; MYOG; TGFB1*	0	*0*	5/210	0/210	5/210
GO:0048864	stem cell development	5	210	16/3649	1.56e-03	1.56e-03	2.47e-02	2	*EDNRB; SNAI2*	3	*CITED2; EDN1; EDNRA*	2/210	3/210	5/210
GO:0009968	negative regulation of signal transduction	23	210	208/3649	1.57e-03	1.57e-03	2.47e-02	19	*APLN; FLCN; FRZB; GRINA; HEYL; IFI6; IGFBP5; ISG15; LFNG; MSTN; NOS3; NR4A2; RASA3; RGS10; RGS19; SH2D1A; SNAI2; SPRY4; ZC3H12A*	4	*CYLD; HSPB1; MYOZ2; RNF34*	19/210	4/210	23/210
GO:0071396	cellular response to lipid	12	210	79/3649	1.60e-03	1.60e-03	2.50e-02	12	*CD14; HEYL; ID3; MSTN; MYOD1; MYOG; PAK1; RBFOX2; RXRG; SNAI2; TGFB1; ZC3H12A*	0	*0*	12/210	0/210	12/210
GO:0007507	heart development	11	210	69/3649	1.67e-03	1.67e-03	2.58e-02	6	*APLN; GJA1; HEYL; ID3; TGFB2; TMEM100*	6	*CITED2; CSRP3; ECE2; ECE2; EDN1; EDNRA*	6/210	6/210	11/210
GO:0035050	embryonic heart tube development	4	210	10/3649	1.70e-03	1.70e-03	2.60e-02	1	*GJA1*	3	*CITED2; EDN1; EDNRA*	1/210	3/210	4/210
GO:0030335	positive regulation of cell migration	12	210	80/3649	1.79e-03	1.79e-03	2.71e-02	8	*ACTG1; IGFBP5; MSTN; PAK1; SNAI2; TGFB1; TGFB2; ZC3H12A*	4	*EDN1; FGF1; HSPB1; RAPGEF2*	8/210	4/210	12/210
GO:0030155	regulation of cell adhesion	14	210	102/3649	1.83e-03	1.83e-03	2.73e-02	13	*ACTG1; ANGPT2; FLCN; GPR4; IRF1; NFKBIZ; NOS3; RND1; SNAI2; SPRY4; TGFB1; TGFB2; ZC3H12A*	1	*CITED2*	13/210	1/210	14/210
GO:0048732	gland development	9	210	50/3649	1.85e-03	1.85e-03	2.73e-02	6	*FRZB; GJA1; IGFBP5; OAS2; TGFB1; TGFB2*	3	*CITED2; EDN1; EDNRA*	6/210	3/210	9/210
GO:0055001	muscle cell development	7	210	32/3649	1.85e-03	1.85e-03	2.73e-02	3	*ACTG1; MYOG; WFIKKN2*	4	*CSRP3; EDN1; EDNRA; MYOZ2*	3/210	4/210	7/210
GO:0035051	cardiocyte differentiation	6	210	24/3649	1.90e-03	1.90e-03	2.77e-02	1	*TGFB2*	6	*CITED2; CSRP3; ECE2; ECE2; EDN1; EDNRA*	1/210	6/210	6/210
GO:0051241	negative regulation of multicellular organismal process	17	210	138/3649	2.07e-03	2.07e-03	2.97e-02	13	*ANGPT2; CTSK; FLCN; FRZB; GJA1; GPR4; IGFBP5; INHBB; IRF1; SNAI2; TGFB1; TGFB2; ZC3H12A*	4	*KCNMA1; MAP2K6; RAPGEF2; THBS2*	13/210	4/210	17/210
GO:2000145	regulation of cell motility	18	210	150/3649	2.07e-03	2.07e-03	2.97e-02	12	*ACTG1; ANGPT2; CNN2; FLCN; GJA1; IGFBP5; MSTN; PAK1; SNAI2; TGFB1; TGFB2; ZC3H12A*	6	*CITED2; EDN1; FGF1; GLUL; HSPB1; RAPGEF2*	12/210	6/210	18/210
GO:0006936	muscle contraction	9	210	51/3649	2.14e-03	2.14e-03	3.04e-02	4	*EDNRB; ORMDL3; TNNT2; ZC3H12A*	5	*EDN1; EDNRA; KCNMA1; MAP2K6; ROCK2*	4/210	5/210	9/210
GO:0071900	regulation of protein serine/threonine kinase activity	10	210	61/3649	2.18e-03	2.18e-03	3.08e-02	5	*CCND1; CD40; PAK1; PKIG; TGFB1*	5	*CAB39; CKS2; EDN1; MAP2K6; RAPGEF2*	5/210	5/210	10/210
GO:1901701	cellular response to oxygen-containing compound	20	210	176/3649	2.30e-03	2.30e-03	3.22e-02	16	*ACTB; CD14; GJA1; ID3; IGFBP5; INHBB; MSTN; MYOD1; MYOG; NR4A1; NR4A2; NSG2; RGS10; SNAI2; TGFB1; ZC3H12A*	4	*CSRP3; EDN1; EDNRA; RAPGEF2*	16/210	4/210	20/210

GO:0006357	regulation of transcription by RNA polymerase II	25	210	241/3649	2.44e-03	2.44e-03	3.38e-02	16	*BHLHE40; CCND1; CXXC5; EDNRB; ETV6; FLCN; HEYL; IRF1; MAMSTR; MYOD1; MYOG; NR4A1; NR4A2; SNAI2; TGFB1; ZC3H12A*	10	*ACVR2A; CITED2; CKS2; CSRP3; EDN1; FGF1; MYOZ2; RFX2; TSC22D1; TSC22D1*	16/210	10/210	25/210
GO:0061035	regulation of cartilage development	4	210	11/3649	2.55e-03	2.55e-03	3.51e-02	4	*CTSK; FRZB; SCIN; SNAI2*	0	*0*	4/210	0/210	4/210
GO:0033993	response to lipid	14	210	106/3649	2.65e-03	2.65e-03	3.58e-02	13	*CD14; HEYL; ID3; MSTN; MYOD1; MYOG; PAK1; RBFOX2; RXRG; SNAI2; TGFB1; TGFB2; ZC3H12A*	1	*MAPKAPK3*	13/210	1/210	14/210
GO:0048863	stem cell differentiation	8	210	43/3649	2.66e-03	2.66e-03	3.58e-02	4	*EDNRB; FRZB; SNAI2; TGFB2*	5	*CITED2; ECE2; ECE2; EDN1; EDNRA*	4/210	5/210	8/210
GO:0043410	positive regulation of MAPK cascade	11	210	73/3649	2.66e-03	2.66e-03	3.58e-02	6	*CD40; PAK1; RELL2; TGFB1; TGFB2; ZC3H12A*	5	*EDN1; FGF1; HCRTR1; MAP2K6; RAPGEF2*	6/210	5/210	11/210
GO:0040017	positive regulation of locomotion	12	210	84/3649	2.74e-03	2.74e-03	3.59e-02	8	*ACTG1; IGFBP5; MSTN; PAK1; SNAI2; TGFB1; TGFB2; ZC3H12A*	4	*EDN1; FGF1; HSPB1; RAPGEF2*	8/210	4/210	12/210
GO:2000147	positive regulation of cell motility	12	210	84/3649	2.74e-03	2.74e-03	3.59e-02	8	*ACTG1; IGFBP5; MSTN; PAK1; SNAI2; TGFB1; TGFB2; ZC3H12A*	4	*EDN1; FGF1; HSPB1; RAPGEF2*	8/210	4/210	12/210
GO:1901700	response to oxygen-containing compound	24	210	230/3649	2.75e-03	2.75e-03	3.59e-02	17	*ACTB; CD14; GJA1; ID3; IGFBP5; INHBB; MSTN; MYOD1; MYOG; NR4A1; NR4A2; NSG2; RGS10; SNAI2; TGFB1; TGFB2; ZC3H12A*	7	*CSRP3; EDN1; EDNRA; KCNMA1; MAPKAPK3; RAPGEF2; UCP3*	17/210	7/210	24/210
GO:0006915	apoptotic process	33	210	352/3649	2.77e-03	2.77e-03	3.59e-02	22	*BLCAP; ETV6; FLCN; FRZB; GJA1; GRINA; IFI6; IRF1; MFSD10; NOS3; NR4A1; NR4A2; ORMDL3; PAK1; S100A14; SCIN; SHISA5; SNAI2; TGFB1; TGFB2; XAF1; ZC3H12A*	11	*BCL2; CITED2; CRYAB; EDNRA; HSPB1; IFT57; KCNMA1; MAP2K6; RAPGEF2; RNF144B; RNF34*	22/210	11/210	33/210
GO:0040012	regulation of locomotion	18	210	154/3649	2.78e-03	2.78e-03	3.59e-02	12	*ACTG1; ANGPT2; CNN2; FLCN; GJA1; IGFBP5; MSTN; PAK1; SNAI2; TGFB1; TGFB2; ZC3H12A*	6	*CITED2; EDN1; FGF1; GLUL; HSPB1; RAPGEF2*	12/210	6/210	18/210
GO:0043549	regulation of kinase activity	16	210	130/3649	2.81e-03	2.81e-03	3.60e-02	9	*CCND1; CD40; MOB3C; MSTN; PAK1; PKIG; TGFB1; TGFB2; WARS1*	7	*CAB39; CKS2; EDN1; FGF1; HSPB1; MAP2K6; RAPGEF2*	9/210	7/210	16/210
GO:0043408	regulation of MAPK cascade	14	210	107/3649	2.90e-03	2.90e-03	3.68e-02	8	*CD40; FLCN; PAK1; RELL2; SPRY4; TGFB1; TGFB2; ZC3H12A*	6	*CYLD; EDN1; FGF1; HCRTR1; MAP2K6; RAPGEF2*	8/210	6/210	14/210
GO:0048870	cell motility	24	210	233/3649	3.27e-03	3.27e-03	4.09e-02	17	*ACTG1; ANGPT2; CD44; CNN2; EDNRB; FLCN; GJA1; IGFBP5; MSTN; NR4A1; NR4A2; PAK1; RHBDF1; SNAI2; TGFB1; TGFB2; ZC3H12A*	7	*CITED2; EDN1; EDNRA; FGF1; GLUL; HSPB1; RAPGEF2*	17/210	7/210	24/210
GO:0051674	localization of cell	24	210	233/3649	3.27e-03	3.27e-03	4.09e-02	17	*ACTG1; ANGPT2; CD44; CNN2; EDNRB; FLCN; GJA1; IGFBP5; MSTN; NR4A1; NR4A2; PAK1; RHBDF1; SNAI2; TGFB1; TGFB2; ZC3H12A*	7	*CITED2; EDN1; EDNRA; FGF1; GLUL; HSPB1; RAPGEF2*	17/210	7/210	24/210
GO:0051272	positive regulation of cellular component movement	12	210	86/3649	3.35e-03	3.35e-03	4.16e-02	8	*ACTG1; IGFBP5; MSTN; PAK1; SNAI2; TGFB1; TGFB2; ZC3H12A*	4	*EDN1; FGF1; HSPB1; RAPGEF2*	8/210	4/210	12/210
GO:0051270	regulation of cellular component movement	18	210	157/3649	3.44e-03	3.44e-03	4.24e-02	12	*ACTG1; ANGPT2; CNN2; FLCN; GJA1; IGFBP5; MSTN; PAK1; SNAI2; TGFB1; TGFB2; ZC3H12A*	6	*CITED2; EDN1; FGF1; GLUL; HSPB1; RAPGEF2*	12/210	6/210	18/210
GO:0030522	intracellular receptor signaling pathway	8	210	45/3649	3.57e-03	3.57e-03	4.29e-02	6	*HEYL; NR4A1; NR4A2; PAK1; RBFOX2; SNAI2*	2	*CITED2; RNF34*	6/210	2/210	8/210
GO:0010646	regulation of cell communication	42	210	487/3649	3.59e-03	3.59e-03	4.29e-02	30	*APLN; CD40; FLCN; FRZB; GJA1; GRINA; HEYL; IFI6; IGFBP5; INHBB; IRF1; ISG15; LFNG; MSTN; NOS3; NR4A2; PAK1; RASA3; RELL2; RGS10; RGS19;*	12	*ACVR2A; CIBAR1; CITED2; CYLD; EDN1; FGF1; HCRTR1; HSPB1; MAP2K6;*	30/210	12/210	42/210

									RHBDF1; SH2D1A; SNAI2; SPRY4; TGFB1; TGFB2; TMEM100; WFIKKN2; ZC3H12A		*MYOZ2; RAPGEF2; RNF34*			
GO:0010927	cellular component assembly involved in morphogenesis	5	210	19/3649	3.60e-03	3.60e-03	4.29e-02	1	*ACTG1*	4	*CSRP3; EDN1; MYOZ2; RFX2*	1/210	4/210	5/210
GO:0045667	regulation of osteoblast differentiation	5	210	19/3649	3.60e-03	3.60e-03	4.29e-02	3	*ID3; IGFBP5; SNAI2*	2	*ACVR2A; RIOX1*	3/210	2/210	5/210
GO:0048584	positive regulation of response to stimulus	32	210	344/3649	3.64e-03	3.64e-03	4.29e-02	18	*ACTB; ACTG1; C4A; CD40; CGAS; FLCN; GJA1; GPR4; IGFBP5; MSTN; MYOG; NFKBIZ; PAK1; RELL2; SH2D1A; TGFB1; TGFB2; ZC3H12A*	14	*ABCC1; ACVR2A; CIBAR1; CITED2; EDN1; FGF1; HCRTR1; HSPB1; MAP2K6; NAPEPLD; RAPGEF2; RBBP8; RNF168; USP13*	18/210	14/210	32/210
GO:0003151	outflow tract morphogenesis	4	210	12/3649	3.66e-03	3.66e-03	4.29e-02	1	*HEYL*	3	*CITED2; EDN1; EDNRA*	1/210	3/210	4/210
GO:0045778	positive regulation of ossification	4	210	12/3649	3.66e-03	3.66e-03	4.29e-02	3	*ISG15; TGFB1; TGFB2*	1	*ACVR2A*	3/210	1/210	4/210
GO:0008283	cell population proliferation	26	210	262/3649	3.72e-03	3.72e-03	4.32e-02	20	*ATG101; CNN2; ETV6; FLCN; FRZB; GJA1; GNAT1; IGFBP5; IRF1; MARCKSL1; MSTN; MYOG; NPPC; NR4A1; PAK1; RHBDF1; SCIN; SNAI2; TGFB1; TGFB2*	6	*CITED2; CKS2; EDN1; FGF1; RAPGEF2; USP13*	20/210	6/210	26/210
GO:0008219	cell death	35	210	387/3649	3.76e-03	3.76e-03	4.33e-02	23	*ASAH1; BLCAP; ETV6; FLCN; FRZB; GJA1; GRINA; IFI6; IRF1; MFSD10; NOS3; NR4A1; NR4A2; ORMDL3; PAK1; S100A14; SCIN; SHISA5; SNAI2; TGFB1; TGFB2; XAF1; ZC3H12A*	12	*BCL2; CITED2; CRYAB; CYLD; EDNRA; HSPB1; IFT57; KCNMA1; MAP2K6; RAPGEF2; RNF144B; RNF34*	23/210	12/210	35/210
GO:0048545	response to steroid hormone	7	210	36/3649	3.77e-03	3.77e-03	4.33e-02	7	*HEYL; MSTN; PAK1; RBFOX2; RXRG; TGFB1; TGFB2*	0	*0*	7/210	0/210	7/210
GO:0023051	regulation of signaling	42	210	489/3649	3.88e-03	3.88e-03	4.42e-02	30	*APLN; CD40; FLCN; FRZB; GJA1; GRINA; HEYL; IFI6; IGFBP5; INHBB; IRF1; ISG15; LFNG; MSTN; NOS3; NR4A2; PAK1; RASA3; RELL2; RGS10; RGS19; RHBDF1; SH2D1A; SNAI2; SPRY4; TGFB1; TGFB2; TMEM100; WFIKKN2; ZC3H12A*	12	*ACVR2A; CIBAR1; CITED2; CYLD; EDN1; FGF1; HCRTR1; HSPB1; MAP2K6; MYOZ2; RAPGEF2; RNF34*	30/210	12/210	42/210
GO:0007169	transmembrane receptor protein tyrosine kinase signaling pathway	11	210	77/3649	4.08e-03	4.08e-03	4.60e-02	7	*ANGPT2; IFI6; IGFBP5; MSTN; PAK1; RHBDF1; TGFB1*	4	*CSRP3; FGF1; HSPB1; RAPGEF2*	7/210	4/210	11/210
GO:0001822	kidney development	8	210	46/3649	4.12e-03	4.12e-03	4.60e-02	5	*EDNRB; FLCN; GPR4; HEYL; ID3*	3	*C1GALT1; EDNRA; FGF1*	5/210	3/210	8/210
GO:0045765	regulation of angiogenesis	8	210	46/3649	4.12e-03	4.12e-03	4.60e-02	4	*ANGPT2; GPR4; WARS1; ZC3H12A*	4	*FGF1; GLUL; HSPB1; THBS2*	4/210	4/210	8/210
GO:0044092	negative regulation of molecular function	20	210	186/3649	4.40e-03	4.40e-03	4.85e-02	16	*ACTB; BHLHE40; GNAT1; HEYL; ID3; IFI6; INKA2; MSTN; NR4A1; ORMDL3; PKIG; PPP1R1B; TGFB2; WARS1; WFIKKN2; ZC3H12A*	4	*ANXA9; CYLD; HSPB1; RNF34*	16/210	4/210	20/210
GO:0003007	heart morphogenesis	7	210	37/3649	4.43e-03	4.43e-03	4.85e-02	4	*GJA1; HEYL; TGFB2; TMEM100*	3	*CITED2; EDN1; EDNRA*	4/210	3/210	7/210
GO:0001934	positive regulation of protein phosphorylation	14	210	112/3649	4.43e-03	4.43e-03	4.85e-02	7	*CCND1; CD40; FLCN; MOB3C; PAK1; TGFB1; TGFB2*	7	*ACVR2A; CAB39; CKS2; EDN1; FGF1; MAP2K6; RAPGEF2*	7/210	7/210	14/210
GO:0002062	chondrocyte differentiation	5	210	20/3649	4.58e-03	4.58e-03	4.98e-02	5	*NPPC; SCIN; SERPINH1; SNAI2; TGFB1*	0	*0*	5/210	0/210	5/210
GO:0051146	striated muscle cell differentiation	8	210	47/3649	4.72e-03	4.72e-03	5.06e-02	5	*ACTG1; IGFBP5; MAMSTR; MYOD1; MYOG*	3	*CSRP3; EDN1; MYOZ2*	5/210	3/210	8/210
GO:1901342	regulation of vasculature development	8	210	47/3649	4.72e-03	4.72e-03	5.06e-02	4	*ANGPT2; GPR4; WARS1; ZC3H12A*	4	*FGF1; GLUL; HSPB1; THBS2*	4/210	4/210	8/210

GO:0051347	positive regulation of transferase activity	12	210	90/3649	4.89e-03	4.89e-03	5.18e-02	6	*CCND1; CD40; MOB3C; PAK1; TGFB1; TGFB2*	6	*CAB39; CKS2; EDN1; FGF1; MAP2K6; RAPGEF2*	6/210	6/210	12/210
GO:0071417	cellular response to organonitrogen compound	12	210	90/3649	4.89e-03	4.89e-03	5.18e-02	8	*ACTB; GJA1; IGFBP5; INHBB; MSTN; NR4A1; NR4A2; NSG2*	4	*CSRP3; EDN1; EDNRA; RAPGEF2*	8/210	4/210	12/210
GO:0030239	myofibril assembly	4	210	13/3649	5.05e-03	5.05e-03	5.21e-02	1	*ACTG1*	3	*CSRP3; EDN1; MYOZ2*	1/210	3/210	4/210
GO:0045661	regulation of myoblast differentiation	4	210	13/3649	5.05e-03	5.05e-03	5.21e-02	4	*ID3; MSTN; MYOG; TGFB1*	0	*0*	4/210	0/210	4/210
GO:0051149	positive regulation of muscle cell differentiation	4	210	13/3649	5.05e-03	5.05e-03	5.21e-02	3	*MAMSTR; MYOD1; MYOG*	1	*EDN1*	3/210	1/210	4/210
GO:0055002	striated muscle cell development	4	210	13/3649	5.05e-03	5.05e-03	5.21e-02	1	*ACTG1*	3	*CSRP3; EDN1; MYOZ2*	1/210	3/210	4/210
GO:0045926	negative regulation of growth	7	210	38/3649	5.17e-03	5.17e-03	5.31e-02	7	*FLCN; FRZB; GJA1; IGFBP5; MSTN; TGFB1; TGFB2*	0	*0*	7/210	0/210	7/210
GO:1901652	response to peptide	9	210	58/3649	5.27e-03	5.27e-03	5.37e-02	6	*GJA1; IGFBP5; INHBB; MSTN; NR4A1; NR4A2*	3	*CSRP3; EDN1; EDNRA*	6/210	3/210	9/210
GO:0072001	renal system development	8	210	48/3649	5.39e-03	5.39e-03	5.46e-02	5	*EDNRB; FLCN; GPR4; HEYL; ID3*	3	*C1GALT1; EDNRA; FGF1*	5/210	3/210	8/210
GO:0006366	transcription by RNA polymerase II	26	210	271/3649	5.86e-03	5.86e-03	5.90e-02	16	*BHLHE40; CCND1; CXXC5; EDNRB; ETV6; FLCN; HEYL; IRF1; MAMSTR; MYOD1; MYOG; NR4A1; NR4A2; SNAI2; TGFB1; ZC3H12A*	11	*ACVR2A; CITED2; CKS2; CSRP3; EDN1; FGF1; MYOZ2; RFX2; RNF168; TSC22D1; TSC22D1*	16/210	11/210	26/210
GO:0032870	cellular response to hormone stimulus	11	210	81/3649	6.05e-03	6.05e-03	6.06e-02	8	*HEYL; INHBB; MSTN; NR4A1; NR4A2; PAK1; RBFOX2; RXRG*	3	*CSRP3; EDN1; EDNRA*	8/210	3/210	11/210
GO:0043434	response to peptide hormone	8	210	49/3649	6.13e-03	6.13e-03	6.10e-02	5	*IGFBP5; INHBB; MSTN; NR4A1; NR4A2*	3	*CSRP3; EDN1; EDNRA*	5/210	3/210	8/210
GO:0000122	negative regulation of transcription by RNA polymerase II	12	210	93/3649	6.39e-03	6.39e-03	6.28e-02	9	*BHLHE40; CCND1; CXXC5; EDNRB; ETV6; FLCN; HEYL; NR4A2; SNAI2*	3	*CITED2; EDN1; MYOZ2*	9/210	3/210	12/210
GO:0030855	epithelial cell differentiation	12	210	93/3649	6.39e-03	6.39e-03	6.28e-02	8	*ASAH1; EDNRB; FRZB; GJA1; GPR4; ID3; PTER; TMEM100*	4	*C1GALT1; EDNRA; KCNMA1; RAPGEF2*	8/210	4/210	12/210
GO:0006672	ceramide metabolic process	4	210	14/3649	6.75e-03	6.75e-03	6.58e-02	3	*ASAH1; ORMDL3; PLPP2*	1	*NEU3*	3/210	1/210	4/210
GO:0045944	positive regulation of transcription by RNA polymerase II	16	210	142/3649	6.78e-03	6.78e-03	6.58e-02	11	*ETV6; FLCN; HEYL; IRF1; MAMSTR; MYOD1; MYOG; NR4A1; NR4A2; TGFB1; ZC3H12A*	5	*ACVR2A; CITED2; CSRP3; EDN1; FGF1*	11/210	5/210	16/210
GO:1901653	cellular response to peptide	8	210	50/3649	6.95e-03	6.95e-03	6.71e-02	5	*GJA1; INHBB; MSTN; NR4A1; NR4A2*	3	*CSRP3; EDN1; EDNRA*	5/210	3/210	8/210
GO:1901699	cellular response to nitrogen compound	13	210	106/3649	7.09e-03	7.09e-03	6.81e-02	9	*ACTB; GJA1; IGFBP5; INHBB; MSTN; NR4A1; NR4A2; NSG2; RGS10*	4	*CSRP3; EDN1; EDNRA; RAPGEF2*	9/210	4/210	13/210
GO:0032101	regulation of response to external stimulus	17	210	156/3649	7.43e-03	7.43e-03	7.09e-02	12	*ANGPT2; CGAS; GPR4; ISG15; MACIR; MSTN; MYOD1; NFKBIZ; SH2D1A; SNAI2; TGFB1; ZC3H12A*	5	*ABCC1; CYLD; FGF1; HSPB1; NAPEPLD*	12/210	5/210	17/210
GO:0051338	regulation of transferase activity	17	210	157/3649	7.91e-03	7.91e-03	7.49e-02	10	*CCND1; CD40; MOB3C; MSTN; ORMDL3; PAK1; PKIG; TGFB1; TGFB2; WARS1*	7	*CAB39; CKS2; EDN1; FGF1; HSPB1; MAP2K6; RAPGEF2*	10/210	7/210	17/210
GO:0071375	cellular response to peptide hormone stimulus	7	210	41/3649	7.96e-03	7.96e-03	7.49e-02	4	*INHBB; MSTN; NR4A1; NR4A2*	3	*CSRP3; EDN1; EDNRA*	4/210	3/210	7/210
GO:0001932	regulation of protein phosphorylation	18	210	170/3649	7.98e-03	7.98e-03	7.49e-02	10	*CCND1; CD40; FLCN; MOB3C; PAK1; PKIG; TGFB1; TGFB2; WARS1; ZC3H12A*	8	*ACVR2A; CAB39; CKS2; EDN1; FGF1; HSPB1; MAP2K6; RAPGEF2*	10/210	8/210	18/210
GO:0007423	sensory organ development	9	210	62/3649	8.22e-03	8.22e-03	7.67e-02	5	*FRZB; GJA1; GNAT1; SOBP; TGFB2*	4	*CITED2; EDN1; EDNRA; KCNMA1*	5/210	4/210	9/210
GO:0007179	transforming growth factor beta receptor	6	210	32/3649	8.69e-03	8.69e-03	7.88e-02	5	*FLCN; MSTN; TGFB1; TGFB2; WFIKKN2*	1	*CITED2*	5/210	1/210	6/210

	signaling pathway													
GO:0090596	sensory organ morphogenesis	6	210	32/3649	8.69e-03	8.69e-03	7.88e-02	3	*FRZB; GNAT1; SOBP*	3	*CITED2; EDN1; EDNRA*	3/210	3/210	6/210
GO:0032835	glomerulus development	4	210	15/3649	8.80e-03	8.80e-03	7.88e-02	3	*EDNRB; GPR4; HEYL*	1	*EDNRA*	3/210	1/210	4/210
GO:0042471	ear morphogenesis	4	210	15/3649	8.80e-03	8.80e-03	7.88e-02	2	*FRZB; SOBP*	2	*EDN1; EDNRA*	2/210	2/210	4/210
GO:0040011	locomotion	24	210	252/3649	8.81e-03	8.81e-03	7.88e-02	17	*ACTG1; ANGPT2; CD44; CNN2; EDNRB; FLCN; GJA1; IGFBP5; MSTN; NR4A1; NR4A2; PAK1; RHBDF1; SNAI2; TGFB1; TGFB2; ZC3H12A*	7	*CITED2; EDN1; EDNRA; FGF1; GLUL; HSPB1; RAPGEF2*	17/210	7/210	24/210
GO:0000165	MAPK cascade	14	210	121/3649	8.82e-03	8.82e-03	7.88e-02	8	*CD40; FLCN; PAK1; RELL2; SPRY4; TGFB1; TGFB2; ZC3H12A*	6	*CYLD; EDN1; FGF1; HCRTR1; MAP2K6; RAPGEF2*	8/210	6/210	14/210
GO:0042327	positive regulation of phosphorylation	14	210	121/3649	8.82e-03	8.82e-03	7.88e-02	7	*CCND1; CD40; FLCN; MOB3C; PAK1; TGFB1; TGFB2*	7	*ACVR2A; CAB39; CKS2; EDN1; FGF1; MAP2K6; RAPGEF2*	7/210	7/210	14/210
GO:0001655	urogenital system development	8	210	52/3649	8.83e-03	8.83e-03	7.88e-02	5	*EDNRB; FLCN; GPR4; HEYL; ID3*	3	*C1GALT1; EDNRA; FGF1*	5/210	3/210	8/210
GO:0010562	positive regulation of phosphorus metabolic process	15	210	134/3649	9.18e-03	9.18e-03	8.11e-02	8	*CCND1; CD40; FLCN; MOB3C; NOS3; PAK1; TGFB1; TGFB2*	7	*ACVR2A; CAB39; CKS2; EDN1; FGF1; MAP2K6; RAPGEF2*	8/210	7/210	15/210
GO:0045937	positive regulation of phosphate metabolic process	15	210	134/3649	9.18e-03	9.18e-03	8.11e-02	8	*CCND1; CD40; FLCN; MOB3C; NOS3; PAK1; TGFB1; TGFB2*	7	*ACVR2A; CAB39; CKS2; EDN1; FGF1; MAP2K6; RAPGEF2*	8/210	7/210	15/210
GO:0051240	positive regulation of multicellular organismal process	20	210	199/3649	9.33e-03	9.33e-03	8.20e-02	15	*APLN; CD14; CGAS; GJA1; INHBB; IRF1; ISG15; LRRN1; MYOG; NFKBIZ; OAS2; OXTR; TGFB1; TGFB2; ZC3H12A*	5	*ACVR2A; CITED2; EDN1; FGF1; HSPB1*	15/210	5/210	20/210
GO:0006952	defense response	25	210	268/3649	9.77e-03	9.77e-03	8.54e-02	20	*ACTG1; AGER; C4A; CD14; CGAS; CXCL16; GPR4; HMGB3; IFI6; IRF1; ISG15; MACIR; MX1; MX2; NFKBIZ; OAS2; S100A14; SH2D1A; TGFB1; ZC3H12A*	5	*ABCC1; CCRL2; CSRP3; CYLD; NAPEPLD*	20/210	5/210	25/210
GO:0002064	epithelial cell development	6	210	33/3649	1.01e-02	1.01e-02	8.74e-02	3	*EDNRB; GJA1; GPR4*	3	*C1GALT1; EDNRA; RAPGEF2*	3/210	3/210	6/210
GO:0014706	striated muscle tissue development	6	210	33/3649	1.01e-02	1.01e-02	8.74e-02	3	*GJA1; TGFB1; TGFB2*	3	*CSRP3; EDN1; EDNRA*	3/210	3/210	6/210
GO:0043067	regulation of programmed cell death	24	210	255/3649	1.02e-02	1.02e-02	8.74e-02	14	*ASAH1; FLCN; FRZB; GJA1; GRINA; IFI6; NOS3; NR4A1; NR4A2; ORMDL3; SCIN; SNAI2; TGFB2; ZC3H12A*	10	*BCL2; CITED2; CRYAB; CYLD; HSPB1; IFT57; KCNMA1; RAPGEF2; RNF144B; RNF34*	14/210	10/210	24/210
GO:0003158	endothelium development	5	210	24/3649	1.04e-02	1.04e-02	8.86e-02	2	*EDNRB; TMEM100*	3	*EDNRA; FGF1; RAPGEF2*	2/210	3/210	5/210
GO:0007292	female gamete generation	5	210	24/3649	1.04e-02	1.04e-02	8.86e-02	3	*ETV6; INHBB; PGRMC1*	2	*EDN1; EDNRA*	3/210	2/210	5/210
GO:0009611	response to wounding	10	210	76/3649	1.09e-02	1.09e-02	9.19e-02	9	*ACTG1; CD44; CNN2; GPR4; INHBB; NOS3; PAK1; TGFB1; TGFB2*	1	*EDNRA*	9/210	1/210	10/210
GO:0016049	cell growth	9	210	65/3649	1.12e-02	1.12e-02	9.36e-02	7	*CXCL16; FLCN; FRZB; GJA1; IGFBP5; TGFB1; TGFB2*	2	*EDN1; EDNRA*	7/210	2/210	9/210
GO:0007589	body fluid secretion	4	210	16/3649	1.12e-02	1.12e-02	9.36e-02	2	*GJA1; OAS2*	2	*EDN1; KCNMA1*	2/210	2/210	4/210
GO:0090100	positive regulation of transmembrane receptor protein serine/threonine kinase signaling pathway	4	210	16/3649	1.12e-02	1.12e-02	9.36e-02	2	*FLCN; TGFB1*	2	*ACVR2A; CITED2*	2/210	2/210	4/210
GO:0033002	muscle cell proliferation	5	210	25/3649	1.24e-02	1.24e-02	1.03e-01	4	*GJA1; IGFBP5; MYOG; TGFB2*	1	*EDN1*	4/210	1/210	5/210
GO:0043583	ear development	5	210	25/3649	1.24e-02	1.24e-02	1.03e-01	2	*FRZB; SOBP*	3	*EDN1; EDNRA; KCNMA1*	2/210	3/210	5/210

GO:0050790	regulation of catalytic activity	34	210	404/3649	1.30e-02	1.30e-02	1.06e-01	23	*CCND1; CD40; GNAT1; GPRC5C; IFI6; INKA2; MOB3C; MSTN; NOS3; NR4A1; NR4A2; ORMDL3; PAK1; PKIG; PPP1R1B; RABEP2; RASA3; RASGRP2; RGS10; TGFB1; TGFB2; WARS1; WFIKKN2*	11	*ANXA9; CAB39; CKS2; EDN1; FGF1; HSPB1; IFT57; MAP2K6; RAPGEF2; RCAN2; RNF34*	23/210	11/210	34/210
GO:0070371	ERK1 and ERK2 cascade	7	210	45/3649	1.32e-02	1.32e-02	1.08e-01	3	*FLCN; SPRY4; TGFB1*	4	*EDN1; FGF1; HCRTR1; RAPGEF2*	3/210	4/210	7/210
GO:0090257	regulation of muscle system process	7	210	45/3649	1.32e-02	1.32e-02	1.08e-01	5	*IGFBP5; MYOG; ORMDL3; TNNT2; ZC3H12A*	2	*EDN1; KCNMA1*	5/210	2/210	7/210
GO:0031032	actomyosin structure organization	6	210	35/3649	1.35e-02	1.35e-02	1.09e-01	3	*ACTG1; CNN2; PAK1*	3	*CSRP3; EDN1; MYOZ2*	3/210	3/210	6/210
GO:0048562	embryonic organ morphogenesis	6	210	35/3649	1.35e-02	1.35e-02	1.09e-01	3	*FRZB; GJA1; SOBP*	3	*CITED2; EDN1; EDNRA*	3/210	3/210	6/210
GO:0001558	regulation of cell growth	8	210	56/3649	1.37e-02	1.37e-02	1.09e-01	7	*CXCL16; FLCN; FRZB; GJA1; IGFBP5; TGFB1; TGFB2*	1	*EDN1*	7/210	1/210	8/210
GO:0048568	embryonic organ development	8	210	56/3649	1.37e-02	1.37e-02	1.09e-01	5	*FRZB; GJA1; ID3; SOBP; TGFB2*	3	*CITED2; EDN1; EDNRA*	5/210	3/210	8/210
GO:0006636	unsaturated fatty acid biosynthetic process	4	210	17/3649	1.40e-02	1.40e-02	1.10e-01	3	*ELOVL5; ELOVL7; FADS3*	1	*EDN1*	3/210	1/210	4/210
GO:0007043	cell-cell junction assembly	4	210	17/3649	1.40e-02	1.40e-02	1.10e-01	4	*ACTB; ACTG1; FLCN; SNAI2*	0	*0*	4/210	0/210	4/210
GO:0030879	mammary gland development	4	210	17/3649	1.40e-02	1.40e-02	1.10e-01	4	*GJA1; IGFBP5; OAS2; TGFB1*	0	*0*	4/210	0/210	4/210
GO:0045639	positive regulation of myeloid cell differentiation	4	210	17/3649	1.40e-02	1.40e-02	1.10e-01	3	*ISG15; SCIN; TGFB1*	1	*ACVR2A*	3/210	1/210	4/210
GO:0140352	export from cell	12	210	103/3649	1.41e-02	1.41e-02	1.10e-01	9	*EDNRB; EXOC3L1; GJA1; INHBB; PAK1; RHBDF1; SCIN; TGFB1; TGFB2*	3	*ABCC1; EDN1; MICAL3*	9/210	3/210	12/210
GO:0032989	cellular component morphogenesis	11	210	91/3649	1.43e-02	1.43e-02	1.11e-01	5	*ACTG1; CD44; GJA1; NR4A2; STXBP1*	6	*CSRP3; EDN1; EDNRA; MYOZ2; RAPGEF2; RFX2*	5/210	6/210	11/210
GO:0001659	temperature homeostasis	5	210	26/3649	1.47e-02	1.47e-02	1.12e-01	3	*FLCN; GJA1; OXTR*	2	*MAP2K6; NAPEPLD*	3/210	2/210	5/210
GO:0006665	sphingolipid metabolic process	5	210	26/3649	1.47e-02	1.47e-02	1.12e-01	4	*ASAH1; FADS3; ORMDL3; PLPP2*	1	*NEU3*	4/210	1/210	5/210
GO:0072006	nephron development	5	210	26/3649	1.47e-02	1.47e-02	1.12e-01	3	*EDNRB; GPR4; HEYL*	2	*EDNRA; FGF1*	3/210	2/210	5/210
GO:0046903	secretion	13	210	116/3649	1.47e-02	1.47e-02	1.12e-01	10	*EDNRB; EXOC3L1; GJA1; INHBB; OAS2; PAK1; RHBDF1; SCIN; TGFB1; TGFB2*	3	*EDN1; KCNMA1; MICAL3*	10/210	3/210	13/210
GO:0006164	purine nucleotide biosynthetic process	7	210	46/3649	1.49e-02	1.49e-02	1.12e-01	7	*ADSS1; ELOVL5; ELOVL7; FLCN; NOS3; NPPC; TGFB1*	0	*0*	7/210	0/210	7/210
GO:0030336	negative regulation of cell migration	7	210	46/3649	1.49e-02	1.49e-02	1.12e-01	6	*ANGPT2; CNN2; FLCN; GJA1; IGFBP5; TGFB1*	1	*CITED2*	6/210	1/210	7/210
GO:0071496	cellular response to external stimulus	7	210	46/3649	1.49e-02	1.49e-02	1.12e-01	7	*CD40; CNN2; FLCN; INHBB; NR4A2; SNAI2; ZC3H12A*	0	*0*	7/210	0/210	7/210
GO:0035556	intracellular signal transduction	35	210	423/3649	1.50e-02	1.50e-02	1.12e-01	24	*CCND1; CD40; CGAS; EDNRB; FLCN; GJA1; GRINA; IFI6; IGFBP5; MSTN; PAK1; PLD2; RASA3; RASGRP2; RELL2; RHOF; RND1; SHISA5; SNAI2; SPRY4; TGFB1; TGFB2; TMEM100; ZC3H12A*	11	*CSRP3; CYLD; EDN1; EDNRA; FGF1; HCRTR1; HSPB1; MAP2K6; MYOZ2; RAPGEF2; RNF34*	24/210	11/210	35/210
GO:0022603	regulation of anatomical structure morphogenesis	14	210	129/3649	1.51e-02	1.51e-02	1.12e-01	7	*ANGPT2; CD44; GJA1; GPR4; SPRY4; WARS1; ZC3H12A*	7	*CITED2; FGF1; FITM2; GLUL; HSPB1; RAPGEF2; THBS2*	7/210	7/210	14/210
GO:0003018	vascular process in circulatory system	6	210	36/3649	1.54e-02	1.54e-02	1.13e-01	2	*EDNRB; GPR4*	4	*ABCC1; EDN1; EDNRA; KCNMA1*	2/210	4/210	6/210
GO:0019932	second-messenger-mediated signaling	6	210	36/3649	1.54e-02	1.54e-02	1.13e-01	3	*CGAS; EDNRB; TMEM100*	3	*EDN1; MYOZ2; RAPGEF2*	3/210	3/210	6/210

GO:0031214	biomineral tissue development	6	210	36/3649	1.54e-02	1.54e-02	1.13e-01	4	*GJA1; ISG15; TGFB1; TUFT1*	2	*ACVR2A; MGP*	4/210	2/210	6/210
GO:0110148	biomineralization	6	210	36/3649	1.54e-02	1.54e-02	1.13e-01	4	*GJA1; ISG15; TGFB1; TUFT1*	2	*ACVR2A; MGP*	4/210	2/210	6/210
GO:0042325	regulation of phosphorylation	19	210	196/3649	1.61e-02	1.61e-02	1.14e-01	11	*CCND1; CD40; FLCN; MOB3C; MSTN; PAK1; PKIG; TGFB1; TGFB2; WARS1; ZC3H12A*	8	*ACVR2A; CAB39; CKS2; EDN1; FGF1; HSPB1; MAP2K6; RAPGEF2*	11/210	8/210	19/210
GO:0006355	regulation of transcription, DNA-templated	35	210	425/3649	1.61e-02	1.61e-02	1.14e-01	20	*BHLHE40; CCND1; CXXC5; EDNRB; ETV6; FLCN; FOXP1; HEYL; ID3; IRF1; MAMSTR; MSTN; MYOD1; MYOG; NR4A1; NR4A2; RBFOX2; SNAI2; TGFB1; ZC3H12A*	16	*ACVR2A; CITED2; CKS2; CRYAB; CSRP3; CYLD; EDN1; FGF1; MYOZ2; PRMT6; RFX2; RIOX1; RNF168; TSC22D1; TSC22D1; USP13*	20/210	16/210	35/210
GO:0006928	movement of cell or subcellular component	25	210	280/3649	1.65e-02	1.65e-02	1.14e-01	18	*ACTG1; ANGPT2; CD44; CNN2; EDNRB; FLCN; GJA1; IGFBP5; KIF3C; MSTN; NR4A1; NR4A2; PAK1; RHBDF1; SNAI2; TGFB1; TGFB2; ZC3H12A*	7	*CITED2; EDN1; EDNRA; FGF1; GLUL; HSPB1; RAPGEF2*	18/210	7/210	25/210
GO:2001234	negative regulation of apoptotic signaling pathway	7	210	47/3649	1.66e-02	1.66e-02	1.14e-01	5	*GRINA; IFI6; NOS3; NR4A2; SNAI2*	2	*HSPB1; RNF34*	5/210	2/210	7/210
GO:1903506	regulation of nucleic acid-templated transcription	35	210	426/3649	1.66e-02	1.66e-02	1.14e-01	20	*BHLHE40; CCND1; CXXC5; EDNRB; ETV6; FLCN; FOXP1; HEYL; ID3; IRF1; MAMSTR; MSTN; MYOD1; MYOG; NR4A1; NR4A2; RBFOX2; SNAI2; TGFB1; ZC3H12A*	16	*ACVR2A; CITED2; CKS2; CRYAB; CSRP3; CYLD; EDN1; FGF1; MYOZ2; PRMT6; RFX2; RIOX1; RNF168; TSC22D1; TSC22D1; USP13*	20/210	16/210	35/210
GO:0009994	oocyte differentiation	3	210	10/3649	1.67e-02	1.67e-02	1.14e-01	1	*PGRMC1*	2	*EDN1; EDNRA*	1/210	2/210	3/210
GO:0048599	oocyte development	3	210	10/3649	1.67e-02	1.67e-02	1.14e-01	1	*PGRMC1*	2	*EDN1; EDNRA*	1/210	2/210	3/210
GO:0050873	brown fat cell differentiation	3	210	10/3649	1.67e-02	1.67e-02	1.14e-01	2	*ARL4A; FLCN*	1	*NAPEPLD*	2/210	1/210	3/210
GO:0051155	positive regulation of striated muscle cell differentiation	3	210	10/3649	1.67e-02	1.67e-02	1.14e-01	2	*MAMSTR; MYOG*	1	*EDN1*	2/210	1/210	3/210
GO:0060389	pathway-restricted SMAD protein phosphorylation	3	210	10/3649	1.67e-02	1.67e-02	1.14e-01	2	*TGFB1; TGFB2*	1	*ACVR2A*	2/210	1/210	3/210
GO:0060840	artery development	3	210	10/3649	1.67e-02	1.67e-02	1.14e-01	0	*0*	3	*CITED2; EDN1; EDNRA*	0/210	3/210	3/210
GO:0070293	renal absorption	3	210	10/3649	1.67e-02	1.67e-02	1.14e-01	1	*EDNRB*	2	*EDN1; EDNRA*	1/210	2/210	3/210
GO:1900744	regulation of p38MAPK cascade	3	210	10/3649	1.67e-02	1.67e-02	1.14e-01	2	*RELL2; ZC3H12A*	1	*CYLD*	2/210	1/210	3/210
GO:1901568	fatty acid derivative metabolic process	3	210	10/3649	1.67e-02	1.67e-02	1.14e-01	2	*ELOVL5; ELOVL7*	1	*FITM2*	2/210	1/210	3/210
GO:1902414	protein localization to cell junction	3	210	10/3649	1.67e-02	1.67e-02	1.14e-01	2	*ACTB; ACTG1*	1	*HSPB1*	2/210	1/210	3/210
GO:0043086	negative regulation of catalytic activity	14	210	131/3649	1.71e-02	1.71e-02	1.16e-01	11	*GNAT1; IFI6; INKA2; MSTN; NR4A1; ORMDL3; PKIG; PPP1R1B; TGFB2; WARS1; WFIKKN2*	3	*ANXA9; HSPB1; RNF34*	11/210	3/210	14/210
GO:0030282	bone mineralization	5	210	27/3649	1.72e-02	1.72e-02	1.16e-01	3	*GJA1; ISG15; TGFB1*	2	*ACVR2A; MGP*	3/210	2/210	5/210
GO:0030308	negative regulation of cell growth	5	210	27/3649	1.72e-02	1.72e-02	1.16e-01	5	*FLCN; FRZB; GJA1; TGFB1; TGFB2*	0	*0*	5/210	0/210	5/210
GO:0019218	regulation of steroid metabolic process	4	210	18/3649	1.72e-02	1.72e-02	1.16e-01	2	*ASAH1; SNAI2*	2	*FGF1; KCNMA1*	2/210	2/210	4/210
GO:2001141	regulation of RNA biosynthetic process	35	210	428/3649	1.78e-02	1.78e-02	1.19e-01	20	*BHLHE40; CCND1; CXXC5; EDNRB; ETV6; FLCN; FOXP1; HEYL; ID3; IRF1; MAMSTR; MSTN; MYOD1; MYOG;*	16	*ACVR2A; CITED2; CKS2; CRYAB; CSRP3; CYLD; EDN1; FGF1; MYOZ2;*	20/210	16/210	35/210

									NR4A1; NR4A2; RBFOX2; SNAI2; TGFB1; ZC3H12A		*PRMT6; RFX2; RIOX1; RNF168; TSC22D1; TSC22D1; USP13*			
GO:2000146	negative regulation of cell motility	7	210	48/3649	1.85e-02	1.85e-02	1.24e-01	6	*ANGPT2; CNN2; FLCN; GJA1; IGFBP5; TGFB1*	1	*CITED2*	6/210	1/210	7/210
GO:0008217	regulation of blood pressure	5	210	28/3649	2.00e-02	2.00e-02	1.32e-01	3	*APLN; EDNRB; OXTR*	2	*EDN1; EDNRA*	3/210	2/210	5/210
GO:0010634	positive regulation of epithelial cell migration	5	210	28/3649	2.00e-02	2.00e-02	1.32e-01	3	*TGFB1; TGFB2; ZC3H12A*	2	*FGF1; HSPB1*	3/210	2/210	5/210
GO:0048771	tissue remodeling	5	210	28/3649	2.00e-02	2.00e-02	1.32e-01	4	*CTSK; GJA1; IGFBP5; TGFB1*	1	*EDNRA*	4/210	1/210	5/210
GO:0010632	regulation of epithelial cell migration	7	210	49/3649	2.06e-02	2.06e-02	1.34e-01	4	*ANGPT2; TGFB1; TGFB2; ZC3H12A*	3	*FGF1; GLUL; HSPB1*	4/210	3/210	7/210
GO:0040013	negative regulation of locomotion	7	210	49/3649	2.06e-02	2.06e-02	1.34e-01	6	*ANGPT2; CNN2; FLCN; GJA1; IGFBP5; TGFB1*	1	*CITED2*	6/210	1/210	7/210
GO:0051271	negative regulation of cellular component movement	7	210	49/3649	2.06e-02	2.06e-02	1.34e-01	6	*ANGPT2; CNN2; FLCN; GJA1; IGFBP5; TGFB1*	1	*CITED2*	6/210	1/210	7/210
GO:0043401	steroid hormone mediated signaling pathway	4	210	19/3649	2.09e-02	2.09e-02	1.34e-01	4	*HEYL; PAK1; RBFOX2; RXRG*	0	*0*	4/210	0/210	4/210
GO:0051147	regulation of muscle cell differentiation	4	210	19/3649	2.09e-02	2.09e-02	1.34e-01	3	*MAMSTR; MYOD1; MYOG*	1	*EDN1*	3/210	1/210	4/210
GO:0106106	cold-induced thermogenesis	4	210	19/3649	2.09e-02	2.09e-02	1.34e-01	3	*FLCN; GJA1; OXTR*	1	*MAP2K6*	3/210	1/210	4/210
GO:0120161	regulation of cold-induced thermogenesis	4	210	19/3649	2.09e-02	2.09e-02	1.34e-01	3	*FLCN; GJA1; OXTR*	1	*MAP2K6*	3/210	1/210	4/210
GO:0002027	regulation of heart rate	3	210	11/3649	2.20e-02	2.20e-02	1.36e-01	1	*EDNRB*	2	*EDN1; EDNRA*	1/210	2/210	3/210
GO:0007585	respiratory gaseous exchange by respiratory system	3	210	11/3649	2.20e-02	2.20e-02	1.36e-01	1	*NR4A2*	2	*EDN1; EDNRA*	1/210	2/210	3/210
GO:0030501	positive regulation of bone mineralization	3	210	11/3649	2.20e-02	2.20e-02	1.36e-01	2	*ISG15; TGFB1*	1	*ACVR2A*	2/210	1/210	3/210
GO:0035914	skeletal muscle cell differentiation	3	210	11/3649	2.20e-02	2.20e-02	1.36e-01	2	*HEYL; NR4A1*	1	*CITED2*	2/210	1/210	3/210
GO:0038066	p38MAPK cascade	3	210	11/3649	2.20e-02	2.20e-02	1.36e-01	2	*RELL2; ZC3H12A*	1	*CYLD*	2/210	1/210	3/210
GO:0043403	skeletal muscle tissue regeneration	3	210	11/3649	2.20e-02	2.20e-02	1.36e-01	3	*GJA1; MSTN; MYOD1*	0	*0*	3/210	0/210	3/210
GO:0061028	establishment of endothelial barrier	3	210	11/3649	2.20e-02	2.20e-02	1.36e-01	1	*EDNRB*	2	*EDNRA; RAPGEF2*	1/210	2/210	3/210
GO:0070169	positive regulation of biomineral tissue development	3	210	11/3649	2.20e-02	2.20e-02	1.36e-01	2	*ISG15; TGFB1*	1	*ACVR2A*	2/210	1/210	3/210
GO:0110151	positive regulation of biomineralization	3	210	11/3649	2.20e-02	2.20e-02	1.36e-01	2	*ISG15; TGFB1*	1	*ACVR2A*	2/210	1/210	3/210
GO:0010817	regulation of hormone levels	8	210	61/3649	2.22e-02	2.22e-02	1.37e-01	5	*CTSK; EDNRB; EXOC3L1; GJA1; INHBB*	4	*ECE2; ECE2; EDN1; KCNMA1*	5/210	4/210	8/210
GO:0007267	cell-cell signaling	16	210	162/3649	2.24e-02	2.24e-02	1.37e-01	11	*CGAS; CHRNE; CHRNG; EDNRB; EXOC3L1; FRZB; GJA1; INHBB; RGS10; SH2D1A; SNAI2*	5	*ARL6; CYLD; EDN1; EDNRA; KCNMA1*	11/210	5/210	16/210
GO:0043491	protein kinase B signaling	6	210	39/3649	2.24e-02	2.24e-02	1.37e-01	6	*CD40; FLCN; IGFBP5; MSTN; TGFB1; TMEM100*	0	*0*	6/210	0/210	6/210
GO:0071560	cellular response to transforming growth factor beta stimulus	6	210	39/3649	2.24e-02	2.24e-02	1.37e-01	5	*FLCN; MSTN; TGFB1; TGFB2; WFIKKN2*	1	*CITED2*	5/210	1/210	6/210
GO:0019220	regulation of phosphate metabolic process	20	210	217/3649	2.27e-02	2.27e-02	1.38e-01	12	*CCND1; CD40; FLCN; MOB3C; MSTN; NOS3; PAK1; PKIG; TGFB1; TGFB2; WARS1; ZC3H12A*	8	*ACVR2A; CAB39; CKS2; EDN1; FGF1; HSPB1;*	12/210	8/210	20/210

											MAP2K6; RAPGEF2			
GO:0051174	regulation of phosphorus metabolic process	20	210	217/3649	2.27e-02	2.27e-02	1.38e-01	12	*CCND1; CD40; FLCN; MOB3C; MSTN; NOS3; PAK1; PKIG; TGFB1; TGFB2; WARS1; ZC3H12A*	8	*ACVR2A; CAB39; CKS2; EDN1; FGF1; HSPB1; MAP2K6; RAPGEF2*	12/210	8/210	20/210
GO:0046942	carboxylic acid transport	5	210	29/3649	2.30e-02	2.30e-02	1.38e-01	2	*GJA1; SLC25A29*	3	*ABCC1; SLC35D1; SLC43A2*	2/210	3/210	5/210
GO:0034097	response to cytokine	14	210	136/3649	2.30e-02	2.30e-02	1.38e-01	10	*ACTG1; ASAH1; CD44; CXCL16; IRF1; ISG15; MX1; OAS2; SPRY4; ZC3H12A*	4	*CCRL2; CYLD; EDN1; MAPKAPK3*	10/210	4/210	14/210
GO:0010941	regulation of cell death	24	210	274/3649	2.31e-02	2.31e-02	1.38e-01	14	*ASAH1; FLCN; FRZB; GJA1; GRINA; IFI6; NOS3; NR4A1; NR4A2; ORMDL3; SCIN; SNAI2; TGFB2; ZC3H12A*	10	*BCL2; CITED2; CRYAB; CYLD; HSPB1; IFT57; KCNMA1; RAPGEF2; RNF144B; RNF34*	14/210	10/210	24/210
GO:0080135	regulation of cellular response to stress	13	210	123/3649	2.31e-02	2.31e-02	1.38e-01	7	*ACTB; CGAS; GRINA; RELL2; SNAI2; TGFB2; ZC3H12A*	6	*CYLD; HSPB1; RBBP8; RNF168; RNF169; USP13*	7/210	6/210	13/210
GO:0042981	regulation of apoptotic process	22	210	246/3649	2.36e-02	2.36e-02	1.40e-01	13	*FLCN; FRZB; GJA1; GRINA; IFI6; NOS3; NR4A1; NR4A2; ORMDL3; SCIN; SNAI2; TGFB2; ZC3H12A*	9	*BCL2; CITED2; CRYAB; HSPB1; IFT57; KCNMA1; RAPGEF2; RNF144B; RNF34*	13/210	9/210	22/210
GO:0042127	regulation of cell population proliferation	19	210	204/3649	2.36e-02	2.36e-02	1.40e-01	16	*ATG101; CNN2; FLCN; FRZB; GJA1; IGFBP5; IRF1; MARCKSL1; MSTN; MYOG; NR4A1; PAK1; SCIN; SNAI2; TGFB1; TGFB2*	3	*EDN1; FGF1; RAPGEF2*	16/210	3/210	19/210
GO:1902532	negative regulation of intracellular signal transduction	11	210	98/3649	2.38e-02	2.38e-02	1.41e-01	7	*FLCN; GRINA; MSTN; RASA3; SNAI2; SPRY4; ZC3H12A*	4	*CYLD; HSPB1; MYOZ2; RNF34*	7/210	4/210	11/210
GO:0031401	positive regulation of protein modification process	15	210	150/3649	2.41e-02	2.41e-02	1.42e-01	8	*CCND1; CD40; FLCN; MOB3C; PAK1; TGFB1; TGFB2; ZC3H12A*	7	*ACVR2A; CAB39; CKS2; EDN1; FGF1; MAP2K6; RAPGEF2*	8/210	7/210	15/210
GO:0032103	positive regulation of response to external stimulus	9	210	74/3649	2.47e-02	2.47e-02	1.43e-01	6	*CGAS; GPR4; MSTN; NFKBIZ; SH2D1A; TGFB1*	3	*ABCC1; HSPB1; NAPEPLD*	6/210	3/210	9/210
GO:0018958	phenol-containing compound metabolic process	4	210	20/3649	2.49e-02	2.49e-02	1.43e-01	2	*CTSK; NR4A2*	2	*EDNRA; RAPGEF2*	2/210	2/210	4/210
GO:0019724	B cell mediated immunity	4	210	20/3649	2.49e-02	2.49e-02	1.43e-01	3	*C4A; CD40; TGFB1*	1	*RNF168*	3/210	1/210	4/210
GO:0032640	tumor necrosis factor production	4	210	20/3649	2.49e-02	2.49e-02	1.43e-01	3	*CD14; OAS2; ZC3H12A*	1	*HSPB1*	3/210	1/210	4/210
GO:0032680	regulation of tumor necrosis factor production	4	210	20/3649	2.49e-02	2.49e-02	1.43e-01	3	*CD14; OAS2; ZC3H12A*	1	*HSPB1*	3/210	1/210	4/210
GO:0045446	endothelial cell differentiation	4	210	20/3649	2.49e-02	2.49e-02	1.43e-01	2	*EDNRB; TMEM100*	2	*EDNRA; RAPGEF2*	2/210	2/210	4/210
GO:0071706	tumor necrosis factor superfamily cytokine production	4	210	20/3649	2.49e-02	2.49e-02	1.43e-01	3	*CD14; OAS2; ZC3H12A*	1	*HSPB1*	3/210	1/210	4/210
GO:1903555	regulation of tumor necrosis factor superfamily cytokine production	4	210	20/3649	2.49e-02	2.49e-02	1.43e-01	3	*CD14; OAS2; ZC3H12A*	1	*HSPB1*	3/210	1/210	4/210
GO:0043542	endothelial cell migration	7	210	51/3649	2.52e-02	2.52e-02	1.44e-01	4	*ANGPT2; NR4A1; TGFB1; ZC3H12A*	3	*FGF1; GLUL; HSPB1*	4/210	3/210	7/210
GO:0072522	purine-containing compound biosynthetic process	7	210	51/3649	2.52e-02	2.52e-02	1.44e-01	7	*ADSS1; ELOVL5; ELOVL7; FLCN; NOS3; NPPC; TGFB1*	0	*0*	7/210	0/210	7/210
GO:0032940	secretion by cell	11	210	99/3649	2.54e-02	2.54e-02	1.44e-01	9	*EDNRB; EXOC3L1; GJA1; INHBB; PAK1;*	2	*EDN1; MICAL3*	9/210	2/210	11/210

									RHBDF1; SCIN; TGFB1; TGFB2					
GO:0031399	regulation of protein modification process	21	210	234/3649	2.58e-02	2.58e-02	1.46e-01	12	*ATG101; CCND1; CD40; FLCN; ISG15; MOB3C; PAK1; PKIG; TGFB1; TGFB2; WARS1; ZC3H12A*	9	*ACVR2A; CAB39; CKS2; EDN1; FGF1; HSPB1; MAP2K6; PRMT6; RAPGEF2*	12/210	9/210	21/210
GO:0043405	regulation of MAP kinase activity	5	210	30/3649	2.64e-02	2.64e-02	1.47e-01	3	*CD40; PAK1; TGFB1*	2	*EDN1; MAP2K6*	3/210	2/210	5/210
GO:0051403	stress-activated MAPK cascade	5	210	30/3649	2.64e-02	2.64e-02	1.47e-01	3	*RELL2; TGFB2; ZC3H12A*	2	*CYLD; MAP2K6*	3/210	2/210	5/210
GO:0071383	cellular response to steroid hormone stimulus	5	210	30/3649	2.64e-02	2.64e-02	1.47e-01	5	*HEYL; MSTN; PAK1; RBFOX2; RXRG*	0	*0*	5/210	0/210	5/210
GO:0010631	epithelial cell migration	8	210	63/3649	2.65e-02	2.65e-02	1.47e-01	5	*ANGPT2; NR4A1; TGFB1; TGFB2; ZC3H12A*	3	*FGF1; GLUL; HSPB1*	5/210	3/210	8/210
GO:0090132	epithelium migration	8	210	63/3649	2.65e-02	2.65e-02	1.47e-01	5	*ANGPT2; NR4A1; TGFB1; TGFB2; ZC3H12A*	3	*FGF1; GLUL; HSPB1*	5/210	3/210	8/210
GO:0080134	regulation of response to stress	21	210	235/3649	2.69e-02	2.69e-02	1.49e-01	13	*ACTB; ACTG1; CGAS; GPR4; GRINA; ISG15; MACIR; NFKBIZ; RELL2; SH2D1A; SNAI2; TGFB2; ZC3H12A*	8	*ABCC1; CYLD; HSPB1; NAPEPLD; RBBP8; RNF168; RNF169; USP13*	13/210	8/210	21/210
GO:0032879	regulation of localization	33	210	411/3649	2.71e-02	2.71e-02	1.49e-01	20	*ACTB; ACTG1; ANGPT2; APLN; CACNB3; CNN2; FLCN; GJA1; IGFBP5; INHBB; MSTN; OAS2; ORMDL3; PAK1; RHBDF1; SCIN; SNAI2; TGFB1; TGFB2; ZC3H12A*	13	*CCT3; CITED2; CRYAB; CSRP3; EDN1; EDNRA; FGF1; FITM2; GLUL; HSPB1; KCNMA1; RAPGEF2; SLC43A2*	20/210	13/210	33/210
GO:0010594	regulation of endothelial cell migration	6	210	41/3649	2.81e-02	2.81e-02	1.49e-01	3	*ANGPT2; TGFB1; ZC3H12A*	3	*FGF1; GLUL; HSPB1*	3/210	3/210	6/210
GO:0060348	bone development	6	210	41/3649	2.81e-02	2.81e-02	1.49e-01	4	*GJA1; NPPC; SERPINH1; TRIM45*	2	*CITED2; MAP2K6*	4/210	2/210	6/210
GO:0070372	regulation of ERK1 and ERK2 cascade	6	210	41/3649	2.81e-02	2.81e-02	1.49e-01	3	*FLCN; SPRY4; TGFB1*	3	*FGF1; HCRTR1; RAPGEF2*	3/210	3/210	6/210
GO:0071559	response to transforming growth factor beta	6	210	41/3649	2.81e-02	2.81e-02	1.49e-01	5	*FLCN; MSTN; TGFB1; TGFB2; WFIKKN2*	1	*CITED2*	5/210	1/210	6/210
GO:0006809	nitric oxide biosynthetic process	3	210	12/3649	2.81e-02	2.81e-02	1.49e-01	3	*DDAH2; NOS3; ZC3H12A*	0	*0*	3/210	0/210	3/210
GO:0021675	nerve development	3	210	12/3649	2.81e-02	2.81e-02	1.49e-01	0	*0*	3	*CITED2; EDN1; EDNRA*	0/210	3/210	3/210
GO:0030042	actin filament depolymerization	3	210	12/3649	2.81e-02	2.81e-02	1.49e-01	1	*SCIN*	2	*DSTN; MICAL3*	1/210	2/210	3/210
GO:0035270	endocrine system development	3	210	12/3649	2.81e-02	2.81e-02	1.49e-01	0	*0*	3	*CITED2; EDN1; EDNRA*	0/210	3/210	3/210
GO:0035850	epithelial cell differentiation involved in kidney development	3	210	12/3649	2.81e-02	2.81e-02	1.49e-01	2	*EDNRB; GPR4*	1	*EDNRA*	2/210	1/210	3/210
GO:0051153	regulation of striated muscle cell differentiation	3	210	12/3649	2.81e-02	2.81e-02	1.49e-01	2	*MAMSTR; MYOG*	1	*EDN1*	2/210	1/210	3/210
GO:1903038	negative regulation of leukocyte cell-cell adhesion	3	210	12/3649	2.81e-02	2.81e-02	1.49e-01	3	*IRF1; NOS3; ZC3H12A*	0	*0*	3/210	0/210	3/210
GO:2001021	negative regulation of response to DNA damage stimulus	3	210	12/3649	2.81e-02	2.81e-02	1.49e-01	2	*CGAS; SNAI2*	1	*RNF169*	2/210	1/210	3/210
GO:0040008	regulation of growth	11	210	101/3649	2.90e-02	2.90e-02	1.52e-01	10	*CXCL16; FLCN; FRZB; GAMT; GJA1; IGFBP5; MSTN; MYOD1; TGFB1; TGFB2*	1	*EDN1*	10/210	1/210	11/210
GO:1902531	regulation of intracellular signal transduction	24	210	280/3649	2.91e-02	2.91e-02	1.52e-01	15	*CD40; FLCN; GJA1; GRINA; IGFBP5; MSTN; PAK1; RASA3; RELL2; SNAI2; SPRY4; TGFB1; TGFB2; TMEM100; ZC3H12A*	9	*CYLD; EDN1; FGF1; HCRTR1; HSPB1; MAP2K6; MYOZ2; RAPGEF2; RNF34*	15/210	9/210	24/210

GO:0097190	apoptotic signaling pathway	12	210	114/3649	2.93e-02	2.93e-02	1.52e-01	9	*FLCN; GRINA; IFI6; NOS3; NR4A2; SHISA5; SNAI2; TGFB1; TGFB2*	3	*BCL2; HSPB1; RNF34*	9/210	3/210	12/210
GO:0000018	regulation of DNA recombination	4	210	21/3649	2.95e-02	2.95e-02	1.52e-01	3	*ACTB; CGAS; TGFB1*	1	*RBBP8*	3/210	1/210	4/210
GO:0009755	hormone-mediated signaling pathway	4	210	21/3649	2.95e-02	2.95e-02	1.52e-01	4	*HEYL; PAK1; RBFOX2; RXRG*	0	*0*	4/210	0/210	4/210
GO:0019722	calcium-mediated signaling	4	210	21/3649	2.95e-02	2.95e-02	1.52e-01	2	*EDNRB; TMEM100*	2	*EDN1; MYOZ2*	2/210	2/210	4/210
GO:0022408	negative regulation of cell-cell adhesion	4	210	21/3649	2.95e-02	2.95e-02	1.52e-01	4	*IRF1; NOS3; TGFB1; ZC3H12A*	0	*0*	4/210	0/210	4/210
GO:0032481	positive regulation of type I interferon production	4	210	21/3649	2.95e-02	2.95e-02	1.52e-01	4	*CGAS; IRF1; ISG15; OAS2*	0	*0*	4/210	0/210	4/210
GO:0072009	nephron epithelium development	4	210	21/3649	2.95e-02	2.95e-02	1.52e-01	2	*EDNRB; HEYL*	2	*EDNRA; FGF1*	2/210	2/210	4/210
GO:0031098	stress-activated protein kinase signaling cascade	5	210	31/3649	3.00e-02	3.00e-02	1.54e-01	3	*RELL2; TGFB2; ZC3H12A*	2	*CYLD; MAP2K6*	3/210	2/210	5/210
GO:0045598	regulation of fat cell differentiation	5	210	31/3649	3.00e-02	3.00e-02	1.54e-01	4	*FLCN; FRZB; TGFB1; ZC3H12A*	1	*NAPEPLD*	4/210	1/210	5/210
GO:0050920	regulation of chemotaxis	5	210	31/3649	3.00e-02	3.00e-02	1.54e-01	3	*ANGPT2; MSTN; TGFB1*	2	*FGF1; HSPB1*	3/210	2/210	5/210
GO:0043085	positive regulation of catalytic activity	16	210	168/3649	3.03e-02	3.03e-02	1.55e-01	9	*CCND1; CD40; MOB3C; NOS3; NR4A2; PAK1; RGS10; TGFB1; TGFB2*	7	*CAB39; CKS2; EDN1; FGF1; IFT57; MAP2K6; RAPGEF2*	9/210	7/210	16/210
GO:0002460	adaptive immune response based on somatic recombination of immune receptors built from immunoglobulin superfamily domains	6	210	42/3649	3.12e-02	3.12e-02	1.58e-01	5	*C4A; CD40; NFKBIZ; TGFB1; ZC3H12A*	1	*RNF168*	5/210	1/210	6/210
GO:0034329	cell junction assembly	6	210	42/3649	3.12e-02	3.12e-02	1.58e-01	5	*ACTB; ACTG1; FLCN; LRRN1; SNAI2*	1	*RAPGEF2*	5/210	1/210	6/210
GO:0001817	regulation of cytokine production	12	210	116/3649	3.30e-02	3.30e-02	1.65e-01	11	*CD14; CGAS; INHBB; IRF1; ISG15; ITGB6; OAS2; SNAI2; TGFB1; TGFB2; ZC3H12A*	1	*HSPB1*	11/210	1/210	12/210
GO:0030036	actin cytoskeleton organization	12	210	116/3649	3.30e-02	3.30e-02	1.65e-01	6	*ACTB; ACTG1; CNN2; PAK1; SCIN; VASP*	6	*CSRP3; DSTN; EDN1; MICAL3; MYOZ2; ROCK2*	6/210	6/210	12/210
GO:0019221	cytokine-mediated signaling pathway	8	210	66/3649	3.39e-02	3.39e-02	1.65e-01	5	*CD44; IRF1; ISG15; MX1; OAS2*	3	*CCRL2; CYLD; EDN1*	5/210	3/210	8/210
GO:0002285	lymphocyte activation involved in immune response	5	210	32/3649	3.40e-02	3.40e-02	1.65e-01	4	*LFNG; NFKBIZ; TGFB1; ZC3H12A*	1	*RNF168*	4/210	1/210	5/210
GO:0006937	regulation of muscle contraction	5	210	32/3649	3.40e-02	3.40e-02	1.65e-01	3	*ORMDL3; TNNT2; ZC3H12A*	2	*EDN1; KCNMA1*	3/210	2/210	5/210
GO:0051321	meiotic cell cycle	5	210	32/3649	3.40e-02	3.40e-02	1.65e-01	1	*PGRMC1*	4	*CKS2; EDN1; EDNRA; RBBP8*	1/210	4/210	5/210
GO:0055067	monovalent inorganic cation homeostasis	5	210	32/3649	3.40e-02	3.40e-02	1.65e-01	1	*EDNRB*	4	*BCL2; EDN1; EDNRA; KCNMA1*	1/210	4/210	5/210
GO:0097191	extrinsic apoptotic signaling pathway	5	210	32/3649	3.40e-02	3.40e-02	1.65e-01	4	*IFI6; NOS3; TGFB1; TGFB2*	1	*RNF34*	4/210	1/210	5/210
GO:0010595	positive regulation of endothelial cell migration	4	210	22/3649	3.44e-02	3.44e-02	1.65e-01	2	*TGFB1; ZC3H12A*	2	*FGF1; HSPB1*	2/210	2/210	4/210
GO:1901888	regulation of cell junction assembly	4	210	22/3649	3.44e-02	3.44e-02	1.65e-01	3	*ACTG1; LRRN1; SNAI2*	1	*RAPGEF2*	3/210	1/210	4/210
GO:1903046	meiotic cell cycle process	4	210	22/3649	3.44e-02	3.44e-02	1.65e-01	1	*PGRMC1*	3	*CKS2; EDN1; EDNRA*	1/210	3/210	4/210
GO:2000779	regulation of double-strand break repair	4	210	22/3649	3.44e-02	3.44e-02	1.65e-01	2	*ACTB; CGAS*	2	*RBBP8; RNF169*	2/210	2/210	4/210

GO:0009152	purine ribonucleotide biosynthetic process	6	210	43/3649	3.46e-02	3.46e-02	1.65e-01	6	*ADSS1; ELOVL5; ELOVL7; FLCN; NPPC; TGFB1*	0	*0*	6/210	0/210	6/210
GO:0001570	vasculogenesis	3	210	13/3649	3.50e-02	3.50e-02	1.65e-01	1	*TMEM100*	2	*CITED2; RAPGEF2*	1/210	2/210	3/210
GO:0002698	negative regulation of immune effector process	3	210	13/3649	3.50e-02	3.50e-02	1.65e-01	3	*TGFB1; TGFB2; ZC3H12A*	0	*0*	3/210	0/210	3/210
GO:0003231	cardiac ventricle development	3	210	13/3649	3.50e-02	3.50e-02	1.65e-01	1	*HEYL*	2	*CITED2; EDNRA*	1/210	2/210	3/210
GO:0032609	interferon-gamma production	3	210	13/3649	3.50e-02	3.50e-02	1.65e-01	3	*CD14; ISG15; ZC3H12A*	0	*0*	3/210	0/210	3/210
GO:0032649	regulation of interferon-gamma production	3	210	13/3649	3.50e-02	3.50e-02	1.65e-01	3	*CD14; ISG15; ZC3H12A*	0	*0*	3/210	0/210	3/210
GO:0032728	positive regulation of interferon-beta production	3	210	13/3649	3.50e-02	3.50e-02	1.65e-01	3	*IRF1; ISG15; OAS2*	0	*0*	3/210	0/210	3/210
GO:0033627	cell adhesion mediated by integrin	3	210	13/3649	3.50e-02	3.50e-02	1.65e-01	3	*ITGB6; SNAI2; TGFB2*	0	*0*	3/210	0/210	3/210
GO:0045911	positive regulation of DNA recombination	3	210	13/3649	3.50e-02	3.50e-02	1.65e-01	2	*ACTB; TGFB1*	1	*RBBP8*	2/210	1/210	3/210
GO:0048477	oogenesis	3	210	13/3649	3.50e-02	3.50e-02	1.65e-01	1	*PGRMC1*	2	*EDN1; EDNRA*	1/210	2/210	3/210
GO:0071772	response to BMP	3	210	13/3649	3.50e-02	3.50e-02	1.65e-01	2	*HEYL; TMEM100*	1	*ACVR2A*	2/210	1/210	3/210
GO:0071773	cellular response to BMP stimulus	3	210	13/3649	3.50e-02	3.50e-02	1.65e-01	2	*HEYL; TMEM100*	1	*ACVR2A*	2/210	1/210	3/210
GO:0009615	response to virus	9	210	79/3649	3.59e-02	3.59e-02	1.69e-01	8	*CGAS; IFI6; IRF1; ISG15; MX1; MX2; OAS2; ZC3H12A*	1	*HSPB1*	8/210	1/210	9/210
GO:0090130	tissue migration	8	210	67/3649	3.67e-02	3.67e-02	1.72e-01	5	*ANGPT2; NR4A1; TGFB1; TGFB2; ZC3H12A*	3	*FGF1; GLUL; HSPB1*	5/210	3/210	8/210
GO:0001816	cytokine production	12	210	118/3649	3.70e-02	3.70e-02	1.73e-01	11	*CD14; CGAS; INHBB; IRF1; ISG15; ITGB6; OAS2; SNAI2; TGFB1; TGFB2; ZC3H12A*	1	*HSPB1*	11/210	1/210	12/210
GO:0040007	growth	14	210	145/3649	3.74e-02	3.74e-02	1.75e-01	11	*CXCL16; FLCN; FRZB; GAMT; GJA1; IGFBP5; MSTN; MYOD1; NPPC; TGFB1; TGFB2*	3	*EDN1; EDNRA; FGF1*	11/210	3/210	14/210
GO:0015711	organic anion transport	6	210	44/3649	3.82e-02	3.82e-02	1.76e-01	3	*GJA1; SLC25A25; SLC25A29*	3	*ABCC1; SLC35D1; SLC43A2*	3/210	3/210	6/210
GO:0007219	Notch signaling pathway	5	210	33/3649	3.83e-02	3.83e-02	1.76e-01	4	*HEYL; LFNG; SNAI2; TMEM100*	1	*WDR12*	4/210	1/210	5/210
GO:0043433	negative regulation of DNA-binding transcription factor activity	5	210	33/3649	3.83e-02	3.83e-02	1.76e-01	4	*BHLHE40; HEYL; ID3; ZC3H12A*	1	*CYLD*	4/210	1/210	5/210
GO:0060070	canonical Wnt signaling pathway	5	210	33/3649	3.83e-02	3.83e-02	1.76e-01	2	*EDNRB; SNAI2*	3	*CYLD; EDN1; EDNRA*	2/210	3/210	5/210
GO:0009628	response to abiotic stimulus	17	210	187/3649	3.85e-02	3.85e-02	1.77e-01	9	*BHLHE40; CCND1; CD40; CNN2; GJA1; GNAT1; GPR4; MYOG; NR4A2*	8	*CITED2; CSRP3; EDN1; EDNRA; FGF1; KCNMA1; RNF168; RNF34*	9/210	8/210	17/210
GO:0002376	immune system process	32	210	408/3649	3.93e-02	3.93e-02	1.80e-01	27	*ACTG1; C4A; CD14; CD40; CGAS; CNN2; CXCL16; EDNRB; ETV6; FLCN; GJA1; HMGB3; IFI6; IRF1; ISG15; LFNG; LGALS9; MSTN; MX1; MX2; NFKBIZ; OAS2; SCIN; SH2D1A; TGFB1; TGFB2; ZC3H12A*	5	*ACVR2A; CITED2; CYLD; MAPKAPK3; RNF168*	27/210	5/210	32/210
GO:0003014	renal system process	4	210	23/3649	3.98e-02	3.98e-02	1.80e-01	1	*EDNRB*	3	*EDN1; EDNRA; KCNMA1*	1/210	3/210	4/210
GO:0043500	muscle adaptation	4	210	23/3649	3.98e-02	3.98e-02	1.80e-01	2	*IGFBP5; MYOG*	2	*EDN1; MYOZ2*	2/210	2/210	4/210
GO:0051781	positive regulation of cell division	4	210	23/3649	3.98e-02	3.98e-02	1.80e-01	2	*TGFB1; TGFB2*	2	*FGF1; MAP10*	2/210	2/210	4/210

GO:1990845	adaptive thermogenesis	4	210	23/3649	3.98e-02	3.98e-02	1.80e-01	3	*FLCN; GJA1; OXTR*	1	*MAP2K6*	3/210	1/210	4/210
GO:2000241	regulation of reproductive process	4	210	23/3649	3.98e-02	3.98e-02	1.80e-01	3	*GJA1; INHBB; PLAT*	1	*CITED2*	3/210	1/210	4/210
GO:0034330	cell junction organization	9	210	81/3649	4.13e-02	4.13e-02	1.85e-01	8	*ACTB; ACTG1; FLCN; GJA1; LRRN1; SNAI2; TGFB1; TGFB2*	1	*RAPGEF2*	8/210	1/210	9/210
GO:0043066	negative regulation of apoptotic process	14	210	147/3649	4.13e-02	4.13e-02	1.85e-01	8	*GRINA; IFI6; NOS3; NR4A1; NR4A2; ORMDL3; SNAI2; ZC3H12A*	6	*BCL2; CITED2; CRYAB; HSPB1; RNF144B; RNF34*	8/210	6/210	14/210
GO:0045087	innate immune response	14	210	147/3649	4.13e-02	4.13e-02	1.85e-01	13	*ACTG1; C4A; CD14; CGAS; CXCL16; HMGB3; IFI6; IRF1; ISG15; MX1; MX2; OAS2; SH2D1A*	1	*CYLD*	13/210	1/210	14/210
GO:0060562	epithelial tube morphogenesis	6	210	45/3649	4.21e-02	4.21e-02	1.85e-01	2	*GJA1; PAK1*	4	*CITED2; EDN1; EDNRA; FGF1*	2/210	4/210	6/210
GO:0001885	endothelial cell development	3	210	14/3649	4.27e-02	4.27e-02	1.85e-01	1	*EDNRB*	2	*EDNRA; RAPGEF2*	1/210	2/210	3/210
GO:0002312	B cell activation involved in immune response	3	210	14/3649	4.27e-02	4.27e-02	1.85e-01	2	*LFNG; TGFB1*	1	*RNF168*	2/210	1/210	3/210
GO:0014896	muscle hypertrophy	3	210	14/3649	4.27e-02	4.27e-02	1.85e-01	1	*IGFBP5*	2	*CSRP3; EDN1*	1/210	2/210	3/210
GO:0014897	striated muscle hypertrophy	3	210	14/3649	4.27e-02	4.27e-02	1.85e-01	1	*IGFBP5*	2	*CSRP3; EDN1*	1/210	2/210	3/210
GO:0032608	interferon-beta production	3	210	14/3649	4.27e-02	4.27e-02	1.85e-01	3	*IRF1; ISG15; OAS2*	0	*0*	3/210	0/210	3/210
GO:0032648	regulation of interferon-beta production	3	210	14/3649	4.27e-02	4.27e-02	1.85e-01	3	*IRF1; ISG15; OAS2*	0	*0*	3/210	0/210	3/210
GO:0032874	positive regulation of stress-activated MAPK cascade	3	210	14/3649	4.27e-02	4.27e-02	1.85e-01	3	*RELL2; TGFB2; ZC3H12A*	0	*0*	3/210	0/210	3/210
GO:0042310	vasoconstriction	3	210	14/3649	4.27e-02	4.27e-02	1.85e-01	1	*EDNRB*	2	*EDN1; EDNRA*	1/210	2/210	3/210
GO:0046209	nitric oxide metabolic process	3	210	14/3649	4.27e-02	4.27e-02	1.85e-01	3	*DDAH2; NOS3; ZC3H12A*	0	*0*	3/210	0/210	3/210
GO:0046634	regulation of alpha-beta T cell activation	3	210	14/3649	4.27e-02	4.27e-02	1.85e-01	3	*IRF1; NFKBIZ; ZC3H12A*	0	*0*	3/210	0/210	3/210
GO:0061005	cell differentiation involved in kidney development	3	210	14/3649	4.27e-02	4.27e-02	1.85e-01	2	*EDNRB; GPR4*	1	*EDNRA*	2/210	1/210	3/210
GO:1990868	response to chemokine	3	210	14/3649	4.27e-02	4.27e-02	1.85e-01	1	*ZC3H12A*	2	*CCRL2; EDN1*	1/210	2/210	3/210
GO:1990869	cellular response to chemokine	3	210	14/3649	4.27e-02	4.27e-02	1.85e-01	1	*ZC3H12A*	2	*CCRL2; EDN1*	1/210	2/210	3/210
GO:0046890	regulation of lipid biosynthetic process	5	210	34/3649	4.28e-02	4.28e-02	1.85e-01	3	*ASAH1; ELOVL5; SNAI2*	2	*FGF1; FITM2*	3/210	2/210	5/210
GO:0006955	immune response	22	210	262/3649	4.38e-02	4.38e-02	1.88e-01	19	*ACTG1; C4A; CD14; CD40; CGAS; CXCL16; HMGB3; IFI6; IRF1; ISG15; LFNG; MX1; MX2; NFKBIZ; OAS2; SH2D1A; TGFB1; TGFB2; ZC3H12A*	3	*CYLD; MAPKAPK3; RNF168*	19/210	3/210	22/210
GO:0006935	chemotaxis	9	210	82/3649	4.41e-02	4.41e-02	1.89e-01	5	*ANGPT2; EDNRB; MSTN; NR4A1; TGFB1*	4	*EDN1; EDNRA; FGF1; HSPB1*	5/210	4/210	9/210
GO:0042330	taxis	9	210	82/3649	4.41e-02	4.41e-02	1.89e-01	5	*ANGPT2; EDNRB; MSTN; NR4A1; TGFB1*	4	*EDN1; EDNRA; FGF1; HSPB1*	5/210	4/210	9/210
GO:0032479	regulation of type I interferon production	4	210	24/3649	4.57e-02	4.57e-02	1.94e-01	4	*CGAS; IRF1; ISG15; OAS2*	0	*0*	4/210	0/210	4/210
GO:0032606	type I interferon production	4	210	24/3649	4.57e-02	4.57e-02	1.94e-01	4	*CGAS; IRF1; ISG15; OAS2*	0	*0*	4/210	0/210	4/210
GO:0048754	branching morphogenesis of an epithelial tube	4	210	24/3649	4.57e-02	4.57e-02	1.94e-01	1	*PAK1*	3	*EDN1; EDNRA; FGF1*	1/210	3/210	4/210
GO:0072073	kidney epithelium development	4	210	24/3649	4.57e-02	4.57e-02	1.94e-01	2	*EDNRB; HEYL*	2	*EDNRA; FGF1*	2/210	2/210	4/210
GO:0007249	I-kappaB kinase/NF-kappaB signaling	6	210	46/3649	4.62e-02	4.62e-02	1.95e-01	3	*EDNRB; GJA1; ZC3H12A*	3	*EDN1; EDNRA; HSPB1*	3/210	3/210	6/210
GO:0042391	regulation of membrane potential	6	210	46/3649	4.62e-02	4.62e-02	1.95e-01	4	*CHRNE; CHRNG; GJA1; IFI6*	2	*BCL2; KCNMA1*	4/210	2/210	6/210

GO:1905114	cell surface receptor signaling pathway involved in cell-cell signaling	9	210	83/3649	4.71e-02	4.71e-02	1.98e-01	5	*CHRNE; CHRNG; EDNRB; FRZB; SNAI2*	4	*ARL6; CYLD; EDN1; EDNRA*	5/210	4/210	9/210
GO:0033554	cellular response to stress	29	210	370/3649	4.91e-02	4.91e-02	2.06e-01	14	*ACTB; CCND1; CGAS; FLCN; GRINA; INHBB; NOS3; NR4A2; PAK1; RELL2; SHISA5; SNAI2; TGFB2; ZC3H12A*	15	*CITED2; CYLD; EDN1; EDNRA; ERCC6L2; FGF1; HSPB1; MAP2K6; PRMT6; RBBP8; RNF168; RNF169; RNF34; SHLD1; USP13*	14/210	15/210	29/210
GO:0051046	regulation of secretion	8	210	71/3649	4.92e-02	4.92e-02	2.06e-01	7	*GJA1; INHBB; OAS2; RHBDF1; SCIN; TGFB1; TGFB2*	1	*EDN1*	7/210	1/210	8/210
GO:0010243	response to organonitrogen compound	13	210	137/3649	4.95e-02	4.95e-02	2.06e-01	8	*ACTB; GJA1; IGFBP5; INHBB; MSTN; NR4A1; NR4A2; NSG2*	5	*CSRP3; EDN1; EDNRA; RAPGEF2; USP13*	8/210	5/210	13/210

4. Enriched Biological Process of Gene Ontology terms to DEG prospect in T2 vs T1 into early weaning group

ID	Description	count	terms	BgRatio	pvalue	p.adjust	qvalue	Count.UpReg	geneID.UpReg	Count.DownReg	geneID.DownReg	GeneRatio.Up	GeneRatio.Down	GeneRatio
GO:0006631	fatty acid metabolic process	19	178	95/3649	8.09e-08	8.09e-08	1.33e-04	9	*ACACA; ACSM1; ADIPOQ; FABP4; FADS2; FASN; HSD17B12; PRKAG3; SCD*	10	*ACADM; ACADVL; ACAT1; BRCA1; CPT1B; CPT2; PLIN5; PRL; SLC27A1; UCP3*	9/178	10/178	19/178
GO:0044255	cellular lipid metabolic process	30	178	233/3649	4.82e-07	4.82e-07	3.95e-04	15	*ACACA; ACSM1; ADIPOQ; FABP4; FADS2; FASN; FGF2; HSD17B12; LSS; ORMDL3; PMVK; PRKAG3; SCD; SPTSSA; ST3GAL6*	15	*ACADM; ACADVL; ACAT1; AGTR1; BRCA1; CASZ1; CPT1B; CPT2; FITM2; NCEH1; PLA2G7; PLIN5; PRL; SLC27A1; UCP3*	15/178	15/178	30/178
GO:0006629	lipid metabolic process	35	178	325/3649	3.69e-06	3.69e-06	2.02e-03	19	*ACACA; ACSM1; ADIPOQ; BDH1; CYP51A1; FABP4; FADS2; FAH; FASN; FGF2; HSD17B12; LSS; NR1D1; ORMDL3; PMVK; PRKAG3; SCD; SPTSSA; ST3GAL6*	16	*ACADM; ACADVL; ACAT1; AGTR1; ANGPTL4; BRCA1; CASZ1; CPT1B; CPT2; FITM2; NCEH1; PLA2G7; PLIN5; PRL; SLC27A1; UCP3*	19/178	16/178	35/178
GO:0044283	small molecule biosynthetic process	21	178	149/3649	7.28e-06	7.28e-06	2.99e-03	15	*ACACA; ACSM1; ADIPOQ; CYP51A1; FADS2; FASN; FGF2; HSD17B12; LSS; NR1D1; PMVK; PRKAG3; PYCR1; SCD; SPTSSA*	6	*ACADM; ACADVL; BRCA1; FBP2; PRL; SLC39A14*	15/178	6/178	21/178
GO:0032787	monocarboxylic acid metabolic process	20	178	142/3649	1.24e-05	1.24e-05	4.06e-03	10	*ACACA; ACSM1; ADIPOQ; FABP4; FADS2; FASN; HSD17B12; NR1D1; PRKAG3; SCD*	10	*ACADM; ACADVL; ACAT1; BRCA1; CPT1B; CPT2; PLIN5; PRL; SLC27A1; UCP3*	10/178	10/178	20/178
GO:0008610	lipid biosynthetic process	21	178	156/3649	1.52e-05	1.52e-05	4.14e-03	14	*ACACA; ACSM1; CYP51A1; FADS2; FASN; FGF2; HSD17B12; LSS; NR1D1; ORMDL3; PMVK; PRKAG3; SCD; SPTSSA*	7	*ACADVL; ACAT1; BRCA1; FITM2; PLIN5; PRL; SLC27A1*	14/178	7/178	21/178
GO:0006635	fatty acid beta-oxidation	7	178	19/3649	1.79e-05	1.79e-05	4.19e-03	1	*ADIPOQ*	6	*ACADM; ACADVL; ACAT1; CPT1B; CPT2; PLIN5*	1/178	6/178	7/178
GO:0009062	fatty acid catabolic process	7	178	21/3649	3.80e-05	3.80e-05	6.53e-03	1	*ADIPOQ*	6	*ACADM; ACADVL; ACAT1; CPT1B; CPT2; PLIN5*	1/178	6/178	7/178
GO:0019752	carboxylic acid metabolic process	26	178	232/3649	3.98e-05	3.98e-05	6.53e-03	14	*ACACA; ACSM1; ADIPOQ; DBT; DDAH2; FABP4; FADS2; FAH; FASN; HSD17B12; NR1D1; PRKAG3; PYCR1; SCD*	12	*ACADM; ACADVL; ACAT1; BRCA1; CPT1B; CPT2; DDC; ODC1; PLIN5; PRL; SLC27A1; UCP3*	14/178	12/178	26/178
GO:0043436	oxoacid metabolic process	26	178	232/3649	3.98e-05	3.98e-05	6.53e-03	14	*ACACA; ACSM1; ADIPOQ; DBT; DDAH2; FABP4; FADS2; FAH; FASN; HSD17B12; NR1D1; PRKAG3; PYCR1; SCD*	12	*ACADM; ACADVL; ACAT1; BRCA1; CPT1B; CPT2; DDC; ODC1; PLIN5; PRL; SLC27A1; UCP3*	14/178	12/178	26/178
GO:0006082	organic acid metabolic process	26	178	234/3649	4.62e-05	4.62e-05	6.83e-03	14	*ACACA; ACSM1; ADIPOQ; DBT; DDAH2; FABP4; FADS2; FAH; FASN; HSD17B12; NR1D1; PRKAG3; PYCR1; SCD*	12	*ACADM; ACADVL; ACAT1; BRCA1; CPT1B; CPT2; DDC; ODC1; PLIN5; PRL; SLC27A1; UCP3*	14/178	12/178	26/178
GO:0072330	monocarboxylic acid biosynthetic process	11	178	55/3649	4.99e-05	4.99e-05	6.83e-03	8	*ACACA; ACSM1; FADS2; FASN; HSD17B12; NR1D1; PRKAG3; SCD*	3	*ACADVL; BRCA1; PRL*	8/178	3/178	11/178
GO:0030258	lipid modification	9	178	38/3649	6.10e-05	6.10e-05	7.70e-03	1	*ADIPOQ*	8	*ACADM; ACADVL; ACAT1; AGTR1; CASZ1; CPT1B; CPT2; PLIN5*	1/178	8/178	9/178
GO:0006633	fatty acid biosynthetic process	10	178	48/3649	7.69e-05	7.69e-05	9.02e-03	7	*ACACA; ACSM1; FADS2; FASN; HSD17B12; PRKAG3; SCD*	3	*ACADVL; BRCA1; PRL*	7/178	3/178	10/178
GO:0033692	cellular polysaccharide biosynthetic process	5	178	11/3649	9.50e-05	9.50e-05	9.63e-03	3	*NR1D1; PPP1R3C; PRKAG3*	2	*ACADM; UGP2*	3/178	2/178	5/178
GO:0019395	fatty acid oxidation	7	178	24/3649	9.98e-05	9.98e-05	9.63e-03	1	*ADIPOQ*	6	*ACADM; ACADVL; ACAT1; CPT1B; CPT2; PLIN5*	1/178	6/178	7/178

GO:0072329	monocarboxylic acid catabolic process	7	178	24/3649	9.98e-05	9.98e-05	9.63e-03	1	*ADIPOQ*	6	*ACADM; ACADVL; ACAT1; CPT1B; CPT2; PLIN5*	1/178	6/178	7/178
GO:0034440	lipid oxidation	7	178	25/3649	1.33e-04	1.33e-04	1.21e-02	1	*ADIPOQ*	6	*ACADM; ACADVL; ACAT1; CPT1B; CPT2; PLIN5*	1/178	6/178	7/178
GO:0000271	polysaccharide biosynthetic process	5	178	13/3649	2.44e-04	2.44e-04	2.11e-02	3	*NR1D1; PPP1R3C; PRKAG3*	2	*ACADM; UGP2*	3/178	2/178	5/178
GO:0016051	carbohydrate biosynthetic process	8	178	37/3649	3.13e-04	3.13e-04	2.57e-02	4	*ADIPOQ; NR1D1; PPP1R3C; PRKAG3*	4	*ACADM; FBP2; SLC39A14; UGP2*	4/178	4/178	8/178
GO:0016054	organic acid catabolic process	10	178	57/3649	3.46e-04	3.46e-04	2.58e-02	4	*ADIPOQ; DBT; DDAH2; FAH*	6	*ACADM; ACADVL; ACAT1; CPT1B; CPT2; PLIN5*	4/178	6/178	10/178
GO:0046395	carboxylic acid catabolic process	10	178	57/3649	3.46e-04	3.46e-04	2.58e-02	4	*ADIPOQ; DBT; DDAH2; FAH*	6	*ACADM; ACADVL; ACAT1; CPT1B; CPT2; PLIN5*	4/178	6/178	10/178
GO:0045834	positive regulation of lipid metabolic process	7	178	30/3649	4.57e-04	4.57e-04	3.26e-02	3	*ADIPOQ; FGF2; NR1D1*	4	*AGTR1; PLIN5; PRL; SLC27A1*	3/178	4/178	7/178
GO:0044281	small molecule metabolic process	38	178	458/3649	5.05e-04	5.05e-04	3.45e-02	21	*ACACA; ACSM1; ADIPOQ; CYP51A1; DBT; DDAH2; FABP4; FADS2; FAH; FASN; FGF2; HSD17B12; LSS; MTHFR; NR1D1; NT5E; PMVK; PRKAG3; PYCR1; SCD; SPTSSA*	17	*ACADM; ACADVL; ACAT1; BRCA1; CPT1B; CPT2; DDC; FBP2; FITM2; NCEH1; ODC1; PLA2G7; PLIN5; PRL; SLC27A1; SLC39A14; UCP3*	21/178	17/178	38/178
GO:0019216	regulation of lipid metabolic process	11	178	72/3649	6.10e-04	6.10e-04	3.94e-02	3	*ADIPOQ; FGF2; NR1D1*	8	*ACADVL; AGTR1; BRCA1; CASZ1; FITM2; PLIN5; PRL; SLC27A1*	3/178	8/178	11/178
GO:0010565	regulation of cellular ketone metabolic process	6	178	23/3649	6.24e-04	6.24e-04	3.94e-02	2	*ADIPOQ; NR1D1*	4	*ACADVL; BRCA1; PLIN5; PRL*	2/178	4/178	6/178
GO:0034637	cellular carbohydrate biosynthetic process	5	178	16/3649	7.35e-04	7.35e-04	4.47e-02	3	*NR1D1; PPP1R3C; PRKAG3*	2	*ACADM; UGP2*	3/178	2/178	5/178
GO:0046394	carboxylic acid biosynthetic process	12	178	86/3649	8.05e-04	8.05e-04	4.72e-02	9	*ACACA; ACSM1; FADS2; FASN; HSD17B12; NR1D1; PRKAG3; PYCR1; SCD*	3	*ACADVL; BRCA1; PRL*	9/178	3/178	12/178
GO:0016053	organic acid biosynthetic process	12	178	87/3649	8.95e-04	8.95e-04	4.99e-02	9	*ACACA; ACSM1; FADS2; FASN; HSD17B12; NR1D1; PRKAG3; PYCR1; SCD*	3	*ACADVL; BRCA1; PRL*	9/178	3/178	12/178
GO:0055017	cardiac muscle tissue growth	4	178	10/3649	9.12e-04	9.12e-04	4.99e-02	4	*FGF2; MEF2C; PI16; TGFB2*	0	*0*	4/178	0/178	4/178
GO:0019217	regulation of fatty acid metabolic process	5	178	17/3649	1.00e-03	1.00e-03	5.25e-02	1	*ADIPOQ*	4	*ACADVL; BRCA1; PLIN5; PRL*	1/178	4/178	5/178
GO:0046890	regulation of lipid biosynthetic process	7	178	34/3649	1.02e-03	1.02e-03	5.25e-02	1	*NR1D1*	6	*ACADVL; BRCA1; FITM2; PLIN5; PRL; SLC27A1*	1/178	6/178	7/178
GO:0060419	heart growth	4	178	11/3649	1.38e-03	1.38e-03	6.86e-02	4	*FGF2; MEF2C; PI16; TGFB2*	0	*0*	4/178	0/178	4/178
GO:0044242	cellular lipid catabolic process	8	178	47/3649	1.68e-03	1.68e-03	7.50e-02	1	*ADIPOQ*	7	*ACADM; ACADVL; ACAT1; CPT1B; CPT2; PLA2G7; PLIN5*	1/178	7/178	8/178
GO:0005977	glycogen metabolic process	5	178	19/3649	1.74e-03	1.74e-03	7.50e-02	3	*NR1D1; PPP1R3C; PRKAG3*	2	*ACADM; UGP2*	3/178	2/178	5/178
GO:0006073	cellular glucan metabolic process	5	178	19/3649	1.74e-03	1.74e-03	7.50e-02	3	*NR1D1; PPP1R3C; PRKAG3*	2	*ACADM; UGP2*	3/178	2/178	5/178
GO:0044042	glucan metabolic process	5	178	19/3649	1.74e-03	1.74e-03	7.50e-02	3	*NR1D1; PPP1R3C; PRKAG3*	2	*ACADM; UGP2*	3/178	2/178	5/178
GO:0071466	cellular response to xenobiotic stimulus	5	178	19/3649	1.74e-03	1.74e-03	7.50e-02	3	*ADIPOQ; ANKRD1; MEF2C*	2	*MYC; NCEH1*	3/178	2/178	5/178
GO:0006112	energy reserve metabolic process	5	178	20/3649	2.22e-03	2.22e-03	9.36e-02	3	*NR1D1; PPP1R3C; PRKAG3*	2	*ACADM; UGP2*	3/178	2/178	5/178
GO:0048738	cardiac muscle tissue development	6	178	29/3649	2.30e-03	2.30e-03	9.43e-02	5	*ANKRD1; FGF2; MEF2C; PI16; TGFB2*	1	*ACADM*	5/178	1/178	6/178
GO:0051241	negative regulation of multicellular organismal process	15	178	138/3649	2.58e-03	2.58e-03	1.03e-01	8	*ADIPOQ; FGF2; INHBB; MEF2C; NR1D1; PFN2; PI16; TGFB2*	7	*ASPN; CDK5R1; GADD45A; HSP90AB1; LAPTM4B; MAP2K6; MYC*	8/178	7/178	15/178
GO:0051403	stress-activated MAPK cascade	6	178	30/3649	2.76e-03	2.76e-03	1.05e-01	1	*TGFB2*	5	*DNAJA1; GADD45A; IGF1R; MAP2K6; MYC*	1/178	5/178	6/178
GO:0044264	cellular polysaccharide metabolic process	5	178	21/3649	2.81e-03	2.81e-03	1.05e-01	3	*NR1D1; PPP1R3C; PRKAG3*	2	*ACADM; UGP2*	3/178	2/178	5/178

GO:2000278	regulation of DNA biosynthetic process	5	178	21/3649	2.81e-03	2.81e-03	1.05e-01	3	*ADIPOQ; ANKRD1; FGF2*	2	*CCT3; MYC*	3/178	2/178	5/178
GO:0031098	stress-activated protein kinase signaling cascade	6	178	31/3649	3.29e-03	3.29e-03	1.20e-01	1	*TGFB2*	5	*DNAJA1; GADD45A; IGF1R; MAP2K6; MYC*	1/178	5/178	6/178
GO:0060191	regulation of lipase activity	4	178	14/3649	3.73e-03	3.73e-03	1.33e-01	1	*FGF2*	3	*AGTR1; ANGPTL4; PLIN5*	1/178	3/178	4/178
GO:0043065	positive regulation of apoptotic process	11	178	91/3649	4.26e-03	4.26e-03	1.44e-01	6	*ADIPOQ; ANKRD1; CLU; MEF2C; MLLT11; TGFB2*	5	*CDK5R1; DNAJA1; GADD45A; MYC; SHQ1*	6/178	5/178	11/178
GO:0005976	polysaccharide metabolic process	5	178	23/3649	4.29e-03	4.29e-03	1.44e-01	3	*NR1D1; PPP1R3C; PRKAG3*	2	*ACADM; UGP2*	3/178	2/178	5/178
GO:0003008	system process	20	178	219/3649	4.30e-03	4.30e-03	1.44e-01	13	*ADIPOQ; CHRNA3; CHRNE; INHBB; LMCD1; LUM; MEF2C; NPR3; ORMDL3; PI16; PRKAG3; RETREG1; TGFB2*	7	*ACTA2; AGTR1; MAP2K6; RIPOR2; SCN3B; SCN4B; TMEM120A*	13/178	7/178	20/178
GO:0009410	response to xenobiotic stimulus	6	178	33/3649	4.56e-03	4.56e-03	1.46e-01	4	*ADIPOQ; ANKRD1; MEF2C; TGFB2*	2	*MYC; NCEH1*	4/178	2/178	6/178
GO:0014706	striated muscle tissue development	6	178	33/3649	4.56e-03	4.56e-03	1.46e-01	5	*ANKRD1; FGF2; MEF2C; PI16; TGFB2*	1	*ACADM*	5/178	1/178	6/178
GO:0043068	positive regulation of programmed cell death	11	178	92/3649	4.63e-03	4.63e-03	1.46e-01	6	*ADIPOQ; ANKRD1; CLU; MEF2C; MLLT11; TGFB2*	5	*CDK5R1; DNAJA1; GADD45A; MYC; SHQ1*	6/178	5/178	11/178
GO:0035051	cardiocyte differentiation	5	178	24/3649	5.20e-03	5.20e-03	1.61e-01	4	*ECE2; MEF2C; PI16; TGFB2*	2	*ACADM; ECE2*	4/178	2/178	5/178
GO:0044057	regulation of system process	10	178	81/3649	5.45e-03	5.45e-03	1.64e-01	7	*ADIPOQ; INHBB; LMCD1; ORMDL3; PI16; PRKAG3; TGFB2*	3	*AGTR1; SCN3B; SCN4B*	7/178	3/178	10/178
GO:1901617	organic hydroxy compound biosynthetic process	7	178	45/3649	5.49e-03	5.49e-03	1.64e-01	6	*CYP51A1; FGF2; LSS; NR1D1; PMVK; SPTSSA*	1	*DDC*	6/178	1/178	7/178
GO:0032872	regulation of stress-activated MAPK cascade	5	178	25/3649	6.25e-03	6.25e-03	1.76e-01	1	*TGFB2*	4	*DNAJA1; GADD45A; IGF1R; MYC*	1/178	4/178	5/178
GO:0006637	acyl-CoA metabolic process	4	178	16/3649	6.28e-03	6.28e-03	1.76e-01	2	*ACACA; PMVK*	2	*ACAT1; FITM2*	2/178	2/178	4/178
GO:0035383	thioester metabolic process	4	178	16/3649	6.28e-03	6.28e-03	1.76e-01	2	*ACACA; PMVK*	2	*ACAT1; FITM2*	2/178	2/178	4/178
GO:0016042	lipid catabolic process	9	178	70/3649	6.32e-03	6.32e-03	1.76e-01	1	*ADIPOQ*	8	*ACADM; ACADVL; ACAT1; CPT1B; CPT2; NCEH1; PLA2G7; PLIN5*	1/178	8/178	9/178
GO:0044282	small molecule catabolic process	10	178	84/3649	7.06e-03	7.06e-03	1.93e-01	4	*ADIPOQ; DBT; DDAH2; FAH*	6	*ACADM; ACADVL; ACAT1; CPT1B; CPT2; PLIN5*	4/178	6/178	10/178
GO:0070302	regulation of stress-activated protein kinase signaling cascade	5	178	26/3649	7.44e-03	7.44e-03	1.98e-01	1	*TGFB2*	4	*DNAJA1; GADD45A; IGF1R; MYC*	1/178	4/178	5/178
GO:0009725	response to hormone	11	178	98/3649	7.49e-03	7.49e-03	1.98e-01	7	*ADIPOQ; INHBB; MEF2C; NR1D1; RBFOX2; RXRG; TGFB2*	4	*AGTR1; DNAJA1; IGF1R; SLC39A14*	7/178	4/178	11/178
GO:0071900	regulation of protein serine/threonine kinase activity	8	178	61/3649	8.77e-03	8.77e-03	2.29e-01	2	*ADIPOQ; PRKAG3*	6	*CDK5R1; CHORDC1; CKS2; DNAJA1; GADD45A; MAP2K6*	2/178	6/178	8/178
GO:0006094	gluconeogenesis	4	178	18/3649	9.78e-03	9.78e-03	2.32e-01	1	*ADIPOQ*	3	*ACADM; FBP2; SLC39A14*	1/178	3/178	4/178
GO:0019319	hexose biosynthetic process	4	178	18/3649	9.78e-03	9.78e-03	2.32e-01	1	*ADIPOQ*	3	*ACADM; FBP2; SLC39A14*	1/178	3/178	4/178
GO:0046889	positive regulation of lipid biosynthetic process	4	178	18/3649	9.78e-03	9.78e-03	2.32e-01	1	*NR1D1*	3	*PLIN5; PRL; SLC27A1*	1/178	3/178	4/178
GO:0061077	chaperone-mediated protein folding	4	178	18/3649	9.78e-03	9.78e-03	2.32e-01	1	*CLU*	3	*CCT3; CHORDC1; HSPH1*	1/178	3/178	4/178
GO:0010942	positive regulation of cell death	11	178	102/3649	1.01e-02	1.01e-02	2.32e-01	6	*ADIPOQ; ANKRD1; CLU; MEF2C; MLLT11; TGFB2*	5	*CDK5R1; DNAJA1; GADD45A; MYC; SHQ1*	6/178	5/178	11/178
GO:0001818	negative regulation of cytokine production	5	178	28/3649	1.03e-02	1.03e-02	2.32e-01	3	*ADIPOQ; INHBB; TGFB2*	2	*HSP90AB1; LAPTM4B*	3/178	2/178	5/178
GO:0046165	alcohol biosynthetic process	5	178	28/3649	1.03e-02	1.03e-02	2.32e-01	5	*CYP51A1; FGF2; LSS; PMVK; SPTSSA*	0	*0*	5/178	0/178	5/178
GO:0071560	cellular response to transforming growth factor beta stimulus	6	178	39/3649	1.05e-02	1.05e-02	2.32e-01	3	*ANKRD1; MEF2C; TGFB2*	3	*ASPN; HSP90AB1; NRROS*	3/178	3/178	6/178
GO:0006084	acetyl-CoA metabolic process	3	178	10/3649	1.06e-02	1.06e-02	2.32e-01	2	*ACACA; PMVK*	1	*ACAT1*	2/178	1/178	3/178

GO:0014855	striated muscle cell proliferation	3	178	10/3649	1.06e-02	1.06e-02	2.32e-01	3	*FGF2; MEF2C; TGFB2*	0	*0*	3/178	0/178	3/178
GO:0033344	cholesterol efflux	3	178	10/3649	1.06e-02	1.06e-02	2.32e-01	3	*ADIPOQ; APOE; EEPD1*	0	*0*	3/178	0/178	3/178
GO:0034389	lipid droplet organization	3	178	10/3649	1.06e-02	1.06e-02	2.32e-01	1	*CIDEC*	2	*FITM2; PLIN5*	1/178	2/178	3/178
GO:0071692	protein localization to extracellular region	6	178	40/3649	1.19e-02	1.19e-02	2.42e-01	5	*ANKRD1; INHBB; NR1D1; RHBDF1; TGFB2*	1	*NRROS*	5/178	1/178	6/178
GO:0033865	nucleoside bisphosphate metabolic process	4	178	19/3649	1.19e-02	1.19e-02	2.42e-01	2	*ACACA; PMVK*	2	*ACAT1; FITM2*	2/178	2/178	4/178
GO:0033875	ribonucleoside bisphosphate metabolic process	4	178	19/3649	1.19e-02	1.19e-02	2.42e-01	2	*ACACA; PMVK*	2	*ACAT1; FITM2*	2/178	2/178	4/178
GO:0034032	purine nucleoside bisphosphate metabolic process	4	178	19/3649	1.19e-02	1.19e-02	2.42e-01	2	*ACACA; PMVK*	2	*ACAT1; FITM2*	2/178	2/178	4/178
GO:0071901	negative regulation of protein serine/threonine kinase activity	4	178	19/3649	1.19e-02	1.19e-02	2.42e-01	1	*ADIPOQ*	3	*CHORDC1; DNAJA1; GADD45A*	1/178	3/178	4/178
GO:0050708	regulation of protein secretion	5	178	29/3649	1.19e-02	1.19e-02	2.42e-01	5	*ANKRD1; INHBB; NR1D1; RHBDF1; TGFB2*	0	*0*	5/178	0/178	5/178
GO:0045892	negative regulation of transcription, DNA-templated	14	178	148/3649	1.21e-02	1.21e-02	2.42e-01	8	*ADIPOQ; ANKRD1; FABP4; LMCD1; MEF2C; NR1D1; NUPR2; RBFOX2*	6	*CDK5R1; GADD45A; HSPA8; MYC; RNF168; THAP1*	8/178	6/178	14/178
GO:1902679	negative regulation of RNA biosynthetic process	14	178	149/3649	1.28e-02	1.28e-02	2.50e-01	8	*ADIPOQ; ANKRD1; FABP4; LMCD1; MEF2C; NR1D1; NUPR2; RBFOX2*	6	*CDK5R1; GADD45A; HSPA8; MYC; RNF168; THAP1*	8/178	6/178	14/178
GO:1903507	negative regulation of nucleic acid-templated transcription	14	178	149/3649	1.28e-02	1.28e-02	2.50e-01	8	*ADIPOQ; ANKRD1; FABP4; LMCD1; MEF2C; NR1D1; NUPR2; RBFOX2*	6	*CDK5R1; GADD45A; HSPA8; MYC; RNF168; THAP1*	8/178	6/178	14/178
GO:0071559	response to transforming growth factor beta	6	178	41/3649	1.34e-02	1.34e-02	2.53e-01	3	*ANKRD1; MEF2C; TGFB2*	3	*ASPN; HSP90AB1; NRROS*	3/178	3/178	6/178
GO:0006694	steroid biosynthetic process	5	178	30/3649	1.38e-02	1.38e-02	2.53e-01	5	*CYP51A1; HSD17B12; LSS; NR1D1; PMVK*	0	*0*	5/178	0/178	5/178
GO:0009166	nucleotide catabolic process	3	178	11/3649	1.41e-02	1.41e-02	2.53e-01	1	*NT5E*	2	*ACAT1; FITM2*	1/178	2/178	3/178
GO:0010866	regulation of triglyceride biosynthetic process	3	178	11/3649	1.41e-02	1.41e-02	2.53e-01	0	*0*	3	*FITM2; PLIN5; SLC27A1*	0/178	3/178	3/178
GO:0042304	regulation of fatty acid biosynthetic process	3	178	11/3649	1.41e-02	1.41e-02	2.53e-01	0	*0*	3	*ACADVL; BRCA1; PRL*	0/178	3/178	3/178
GO:0046328	regulation of JNK cascade	3	178	11/3649	1.41e-02	1.41e-02	2.53e-01	0	*0*	3	*DNAJA1; GADD45A; IGF1R*	0/178	3/178	3/178
GO:0046364	monosaccharide biosynthetic process	4	178	20/3649	1.43e-02	1.43e-02	2.53e-01	1	*ADIPOQ*	3	*ACADM; FBP2; SLC39A14*	1/178	3/178	4/178
GO:0062013	positive regulation of small molecule metabolic process	4	178	20/3649	1.43e-02	1.43e-02	2.53e-01	2	*ADIPOQ; NR1D1*	2	*PLIN5; PRL*	2/178	2/178	4/178
GO:0062014	negative regulation of small molecule metabolic process	4	178	20/3649	1.43e-02	1.43e-02	2.53e-01	1	*ADIPOQ*	3	*ACADVL; BRCA1; PLIN5*	1/178	3/178	4/178
GO:0051253	negative regulation of RNA metabolic process	15	178	168/3649	1.57e-02	1.57e-02	2.75e-01	8	*ADIPOQ; ANKRD1; FABP4; LMCD1; MEF2C; NR1D1; NUPR2; RBFOX2*	7	*CDK5R1; GADD45A; HSPA8; MYC; RNF168; SHQ1; THAP1*	8/178	7/178	15/178
GO:0032870	cellular response to hormone stimulus	9	178	81/3649	1.60e-02	1.60e-02	2.76e-01	5	*ADIPOQ; INHBB; MEF2C; RBFOX2; RXRG*	4	*AGTR1; DNAJA1; IGF1R; SLC39A14*	5/178	4/178	9/178
GO:0071495	cellular response to endogenous stimulus	16	178	184/3649	1.62e-02	1.62e-02	2.76e-01	8	*ADIPOQ; ANKRD1; FGF2; INHBB; MEF2C; RBFOX2; RXRG; TGFB2*	8	*AGTR1; ASPN; COL4A1; DNAJA1; HSP90AB1; IGF1R; NRROS; SLC39A14*	8/178	8/178	16/178
GO:0031324	negative regulation of cellular metabolic process	26	178	348/3649	1.67e-02	1.67e-02	2.82e-01	10	*ADIPOQ; ANKRD1; FABP4; LMCD1; MEF2C; NR1D1; NUPR2; PI16; RBFOX2; TGFB2*	16	*ACADVL; ALAD; BCL2; BRCA1; CDK5R1; CHORDC1; DNAJA1; GADD45A; HSP90AB1; HSPA8; LAPTM4B; MYC; PLIN5; RNF168; SHQ1; THAP1*	10/178	16/178	26/178
GO:0070201	regulation of establishment of protein localization	9	178	82/3649	1.73e-02	1.73e-02	2.83e-01	6	*ADIPOQ; ANKRD1; INHBB; NR1D1; RHBDF1; TGFB2*	3	*CCT3; CDK5R1; DNAJA1*	6/178	3/178	9/178
GO:0009892	negative regulation of metabolic process	31	178	436/3649	1.77e-02	1.77e-02	2.83e-01	13	*ADIPOQ; ANKRD1; APOE; CLU; FABP4; INHBB; LMCD1;*	18	*ACADVL; ALAD; BCL2; BRCA1; CDK5R1;*	13/178	18/178	31/178

								MEF2C; NR1D1; NUPR2; PI16; RBFOX2; TGFB2		*CHORDC1; DNAJA1; GADD45A; HSP90AB1; HSPA8; LAPTM4B; MAP2K6; MYC; PLIN5; PRL; RNF168; SHQ1; THAP1*			
GO:0010921	regulation of phosphatase activity	3	178	12/3649	1.81e-02	1.81e-02	2.83e-01	2	*MEF2C; TGFB2*	1	*CASZ1*	2/178	1/178	3/178
GO:0035850	epithelial cell differentiation involved in kidney development	3	178	12/3649	1.81e-02	1.81e-02	2.83e-01	2	*ADIPOQ; MEF2C*	1	*ACTA2*	2/178	1/178	3/178
GO:0046620	regulation of organ growth	3	178	12/3649	1.81e-02	1.81e-02	2.83e-01	3	*FGF2; MEF2C; PI16*	0	*0*	3/178	0/178	3/178
GO:0051153	regulation of striated muscle cell differentiation	3	178	12/3649	1.81e-02	1.81e-02	2.83e-01	2	*MEF2C; PI16*	1	*MAMSTR*	2/178	1/178	3/178
GO:0060193	positive regulation of lipase activity	3	178	12/3649	1.81e-02	1.81e-02	2.83e-01	1	*FGF2*	2	*AGTR1; PLIN5*	1/178	2/178	3/178
GO:0098739	import across plasma membrane	3	178	12/3649	1.81e-02	1.81e-02	2.83e-01	1	*SLC46A1*	2	*SLC39A14; SLC43A2*	1/178	2/178	3/178
GO:0007167	enzyme linked receptor protein signaling pathway	12	178	126/3649	1.87e-02	1.87e-02	2.89e-01	4	*ADIPOQ; INHBB; RHBDF1; TGFB2*	8	*ASPN; CDK5R1; COL4A1; HSP90AB1; IGF1R; NCEH1; NRROS; SLC39A14*	4/178	8/178	12/178
GO:0031327	negative regulation of cellular biosynthetic process	16	178	188/3649	1.95e-02	1.95e-02	2.98e-01	8	*ADIPOQ; ANKRD1; FABP4; LMCD1; MEF2C; NR1D1; NUPR2; RBFOX2*	8	*ACADVL; BRCA1; CDK5R1; GADD45A; HSPA8; MYC; RNF168; THAP1*	8/178	8/178	16/178
GO:0008202	steroid metabolic process	7	178	57/3649	1.96e-02	1.96e-02	2.98e-01	5	*CYP51A1; HSD17B12; LSS; NR1D1; PMVK*	2	*ACADVL; AGTR1*	5/178	2/178	7/178
GO:0061448	connective tissue development	6	178	45/3649	2.07e-02	2.07e-02	3.11e-01	3	*FGF2; MEF2C; MYF5*	3	*ACTA2; MGP; SLC39A14*	3/178	3/178	6/178
GO:0060537	muscle tissue development	8	178	71/3649	2.10e-02	2.10e-02	3.13e-01	5	*ANKRD1; FGF2; MEF2C; PI16; TGFB2*	3	*ACADM; ACTA2; RIPOR2*	5/178	3/178	8/178
GO:0062012	regulation of small molecule metabolic process	7	178	58/3649	2.14e-02	2.14e-02	3.14e-01	2	*ADIPOQ; NR1D1*	5	*ACADM; ACADVL; BRCA1; PLIN5; PRL*	2/178	5/178	7/178
GO:0009719	response to endogenous stimulus	17	178	207/3649	2.24e-02	2.24e-02	3.14e-01	9	*ADIPOQ; ANKRD1; FGF2; INHBB; MEF2C; NR1D1; RBFOX2; RXRG; TGFB2*	8	*AGTR1; ASPN; COL4A1; DNAJA1; HSP90AB1; IGF1R; NRROS; SLC39A14*	9/178	8/178	17/178
GO:0001676	long-chain fatty acid metabolic process	3	178	13/3649	2.27e-02	2.27e-02	3.14e-01	1	*ACSM1*	2	*CPT2; SLC27A1*	1/178	2/178	3/178
GO:0007254	JNK cascade	3	178	13/3649	2.27e-02	2.27e-02	3.14e-01	0	*0*	3	*DNAJA1; GADD45A; IGF1R*	0/178	3/178	3/178
GO:0090207	regulation of triglyceride metabolic process	3	178	13/3649	2.27e-02	2.27e-02	3.14e-01	0	*0*	3	*FITM2; PLIN5; SLC27A1*	0/178	3/178	3/178
GO:1901292	nucleoside phosphate catabolic process	3	178	13/3649	2.27e-02	2.27e-02	3.14e-01	1	*NT5E*	2	*ACAT1; FITM2*	1/178	2/178	3/178
GO:0030336	negative regulation of cell migration	6	178	46/3649	2.29e-02	2.29e-02	3.14e-01	3	*ADIPOQ; FGF2; PFN2*	3	*GADD45A; PTGER4; RIPOR2*	3/178	3/178	6/178
GO:0042180	cellular ketone metabolic process	6	178	46/3649	2.29e-02	2.29e-02	3.14e-01	2	*ADIPOQ; NR1D1*	4	*ACADVL; BRCA1; PLIN5; PRL*	2/178	4/178	6/178
GO:0042391	regulation of membrane potential	6	178	46/3649	2.29e-02	2.29e-02	3.14e-01	3	*CHRNA3; CHRNE; MLLT11*	3	*BCL2; SCN3B; SCN4B*	3/178	3/178	6/178
GO:0051216	cartilage development	5	178	34/3649	2.30e-02	2.30e-02	3.14e-01	3	*FGF2; MEF2C; MYF5*	2	*MGP; SLC39A14*	3/178	2/178	5/178
GO:0051172	negative regulation of nitrogen compound metabolic process	24	178	324/3649	2.33e-02	2.33e-02	3.14e-01	12	*ADIPOQ; ANKRD1; APOE; CLU; FABP4; INHBB; LMCD1; MEF2C; NR1D1; NUPR2; PI16; RBFOX2*	12	*ALAD; CDK5R1; CHORDC1; DNAJA1; GADD45A; HSP90AB1; HSPA8; LAPTM4B; MYC; RNF168; SHQ1; THAP1*	12/178	12/178	24/178
GO:0098657	import into cell	4	178	23/3649	2.34e-02	2.34e-02	3.14e-01	1	*SLC46A1*	3	*SLC27A1; SLC39A14; SLC43A2*	1/178	3/178	4/178
GO:0051239	regulation of multicellular organismal process	28	178	393/3649	2.35e-02	2.35e-02	3.14e-01	15	*ADIPOQ; APOE; FGF2; INHBB; ITGB6; LMCD1; LRRN1; LUM; MEF2C; NR1D1; ORMDL3; PFN2; PI16; PRKAG3; TGFB2*	13	*AGTR1; ASPN; CDK5R1; GADD45A; HSP90AB1; LAPTM4B; MAP2K6; MGP; MYC; PRL; RAB7B; SCN3B; SCN4B*	15/178	13/178	28/178

GO:0043086	negative regulation of catalytic activity	12	178	131/3649	2.46e-02	2.46e-02	3.26e-01	6	*ADIPOQ; FABP4; ORMDL3; PI16; PPP1R1B; TGFB2*	6	*ANGPTL4; CHORDC1; DNAJA1; GADD45A; PLIN5; SLC39A14*	6/178	6/178	12/178
GO:0051146	striated muscle cell differentiation	6	178	47/3649	2.52e-02	2.52e-02	3.30e-01	2	*MEF2C; PI16*	4	*ACADM; ACTA2; MAMSTR; RIPOR2*	2/178	4/178	6/178
GO:0015849	organic acid transport	5	178	35/3649	2.58e-02	2.58e-02	3.36e-01	3	*FABP4; SLC25A29; SLC46A1*	2	*SLC27A1; SLC43A2*	3/178	2/178	5/178
GO:0045934	negative regulation of nucleobase-containing compound metabolic process	15	178	179/3649	2.65e-02	2.65e-02	3.42e-01	8	*ADIPOQ; ANKRD1; FABP4; LMCD1; MEF2C; NR1D1; NUPR2; RBFOX2*	7	*CDK5R1; GADD45A; HSPA8; MYC; RNF168; SHQ1; THAP1*	8/178	7/178	15/178
GO:0009612	response to mechanical stimulus	4	178	24/3649	2.70e-02	2.70e-02	3.42e-01	1	*ANKRD1*	3	*GADD45A; PRL; TMEM120A*	1/178	3/178	4/178
GO:0010817	regulation of hormone levels	7	178	61/3649	2.75e-02	2.75e-02	3.42e-01	5	*CPQ; ECE2; HSD17B12; INHBB; NR1D1*	3	*ECE2; PRL; SLC39A14*	5/178	3/178	7/178
GO:0072001	renal system development	6	178	48/3649	2.76e-02	2.76e-02	3.42e-01	3	*ADIPOQ; FGF2; MEF2C*	3	*ACTA2; AGTR1; COL4A1*	3/178	3/178	6/178
GO:2000146	negative regulation of cell motility	6	178	48/3649	2.76e-02	2.76e-02	3.42e-01	3	*ADIPOQ; FGF2; PFN2*	3	*GADD45A; PTGER4; RIPOR2*	3/178	3/178	6/178
GO:0014066	regulation of phosphatidylinositol 3-kinase signaling	3	178	14/3649	2.79e-02	2.79e-02	3.42e-01	3	*FBXL2; FGF2; TGFB2*	0	*0*	3/178	0/178	3/178
GO:0019432	triglyceride biosynthetic process	3	178	14/3649	2.79e-02	2.79e-02	3.42e-01	0	*0*	3	*FITM2; PLIN5; SLC27A1*	0/178	3/178	3/178
GO:0061005	cell differentiation involved in kidney development	3	178	14/3649	2.79e-02	2.79e-02	3.42e-01	2	*ADIPOQ; MEF2C*	1	*ACTA2*	2/178	1/178	3/178
GO:0071897	DNA biosynthetic process	5	178	36/3649	2.88e-02	2.88e-02	3.46e-01	3	*ADIPOQ; ANKRD1; FGF2*	2	*CCT3; MYC*	3/178	2/178	5/178
GO:1903828	negative regulation of protein localization	5	178	36/3649	2.88e-02	2.88e-02	3.46e-01	3	*ADIPOQ; INHBB; RHBDF1*	2	*DNAJA1; RIPOR2*	3/178	2/178	5/178
GO:0009890	negative regulation of biosynthetic process	16	178	197/3649	2.89e-02	2.89e-02	3.46e-01	8	*ADIPOQ; ANKRD1; FABP4; LMCD1; MEF2C; NR1D1; NUPR2; RBFOX2*	8	*ACADVL; BRCA1; CDK5R1; GADD45A; HSPA8; MYC; RNF168; THAP1*	8/178	8/178	16/178
GO:0040013	negative regulation of locomotion	6	178	49/3649	3.03e-02	3.03e-02	3.55e-01	3	*ADIPOQ; FGF2; PFN2*	3	*GADD45A; PTGER4; RIPOR2*	3/178	3/178	6/178
GO:0051271	negative regulation of cellular component movement	6	178	49/3649	3.03e-02	3.03e-02	3.55e-01	3	*ADIPOQ; FGF2; PFN2*	3	*GADD45A; PTGER4; RIPOR2*	3/178	3/178	6/178
GO:0051223	regulation of protein transport	8	178	76/3649	3.03e-02	3.03e-02	3.55e-01	6	*ADIPOQ; ANKRD1; INHBB; NR1D1; RHBDF1; TGFB2*	2	*CDK5R1; DNAJA1*	6/178	2/178	8/178
GO:0033002	muscle cell proliferation	4	178	25/3649	3.10e-02	3.10e-02	3.58e-01	4	*ADIPOQ; FGF2; MEF2C; TGFB2*	0	*0*	4/178	0/178	4/178
GO:0035265	organ growth	4	178	25/3649	3.10e-02	3.10e-02	3.58e-01	4	*FGF2; MEF2C; PI16; TGFB2*	0	*0*	4/178	0/178	4/178
GO:0030198	extracellular matrix organization	5	178	37/3649	3.20e-02	3.20e-02	3.65e-01	4	*LUM; MYF5; OLFML2B; TGFB2*	1	*COL4A1*	4/178	1/178	5/178
GO:0045229	external encapsulating structure organization	5	178	37/3649	3.20e-02	3.20e-02	3.65e-01	4	*LUM; MYF5; OLFML2B; TGFB2*	1	*COL4A1*	4/178	1/178	5/178
GO:0006695	cholesterol biosynthetic process	3	178	15/3649	3.37e-02	3.37e-02	3.66e-01	3	*CYP51A1; LSS; PMVK*	0	*0*	3/178	0/178	3/178
GO:0032835	glomerulus development	3	178	15/3649	3.37e-02	3.37e-02	3.66e-01	2	*ADIPOQ; MEF2C*	1	*ACTA2*	2/178	1/178	3/178
GO:0055007	cardiac muscle cell differentiation	3	178	15/3649	3.37e-02	3.37e-02	3.66e-01	2	*MEF2C; PI16*	1	*ACADM*	2/178	1/178	3/178
GO:0061333	renal tubule morphogenesis	3	178	15/3649	3.37e-02	3.37e-02	3.66e-01	2	*FGF2; MEF2C*	1	*COL4A1*	2/178	1/178	3/178
GO:0070167	regulation of biomineral tissue development	3	178	15/3649	3.37e-02	3.37e-02	3.66e-01	1	*MEF2C*	2	*ASPN; MGP*	1/178	2/178	3/178
GO:0110149	regulation of biomineralization	3	178	15/3649	3.37e-02	3.37e-02	3.66e-01	1	*MEF2C*	2	*ASPN; MGP*	1/178	2/178	3/178
GO:1902653	secondary alcohol biosynthetic process	3	178	15/3649	3.37e-02	3.37e-02	3.66e-01	3	*CYP51A1; LSS; PMVK*	0	*0*	3/178	0/178	3/178
GO:0008015	blood circulation	8	178	78/3649	3.47e-02	3.47e-02	3.74e-01	3	*ADIPOQ; NPR3; TGFB2*	5	*ACTA2; AGTR1; MAP2K6; SCN3B; SCN4B*	3/178	5/178	8/178
GO:0072006	nephron development	4	178	26/3649	3.53e-02	3.53e-02	3.78e-01	3	*ADIPOQ; FGF2; MEF2C*	1	*ACTA2*	3/178	1/178	4/178
GO:0043062	extracellular structure organization	5	178	38/3649	3.55e-02	3.55e-02	3.78e-01	4	*LUM; MYF5; OLFML2B; TGFB2*	1	*COL4A1*	4/178	1/178	5/178

GO:0030855	epithelial cell differentiation	9	178	93/3649	3.59e-02	3.59e-02	3.80e-01	5	*ADIPOQ; FGF2; MEF2C; PTER; TDRD7*	4	*ACADVL; ACTA2; RILPL2; TAGLN*	5/178	4/178	9/178
GO:0045859	regulation of protein kinase activity	10	178	109/3649	3.82e-02	3.82e-02	3.91e-01	4	*ADIPOQ; FABP4; PRKAG3; TGFB2*	6	*CDK5R1; CHORDC1; CKS2; DNAJA1; GADD45A; MAP2K6*	4/178	6/178	10/178
GO:0001655	urogenital system development	6	178	52/3649	3.91e-02	3.91e-02	3.91e-01	3	*ADIPOQ; FGF2; MEF2C*	3	*ACTA2; AGTR1; COL4A1*	3/178	3/178	6/178
GO:0007178	transmembrane receptor protein serine/threonine kinase signaling pathway	6	178	52/3649	3.91e-02	3.91e-02	3.91e-01	2	*INHBB; TGFB2*	4	*ASPN; HSP90AB1; NCEH1; NRROS*	2/178	4/178	6/178
GO:0001654	eye development	5	178	39/3649	3.91e-02	3.91e-02	3.91e-01	4	*FGF2; MYF5; TDRD7; TGFB2*	1	*COL4A1*	4/178	1/178	5/178
GO:0009306	protein secretion	5	178	39/3649	3.91e-02	3.91e-02	3.91e-01	5	*ANKRD1; INHBB; NR1D1; RHBDF1; TGFB2*	0	*0*	5/178	0/178	5/178
GO:0031330	negative regulation of cellular catabolic process	5	178	39/3649	3.91e-02	3.91e-02	3.91e-01	0	*0*	5	*ALAD; BCL2; HSP90AB1; LAPTM4B; PLIN5*	0/178	5/178	5/178
GO:0035592	establishment of protein localization to extracellular region	5	178	39/3649	3.91e-02	3.91e-02	3.91e-01	5	*ANKRD1; INHBB; NR1D1; RHBDF1; TGFB2*	0	*0*	5/178	0/178	5/178
GO:0051092	positive regulation of NF-kappaB transcription factor activity	4	178	27/3649	3.99e-02	3.99e-02	3.91e-01	1	*CLU*	3	*MAP3K13; PRL; RAB7B*	1/178	3/178	4/178
GO:1904950	negative regulation of establishment of protein localization	4	178	27/3649	3.99e-02	3.99e-02	3.91e-01	3	*ADIPOQ; INHBB; RHBDF1*	1	*DNAJA1*	3/178	1/178	4/178
GO:0016126	sterol biosynthetic process	3	178	16/3649	4.00e-02	4.00e-02	3.91e-01	3	*CYP51A1; LSS; PMVK*	0	*0*	3/178	0/178	3/178
GO:0035725	sodium ion transmembrane transport	3	178	16/3649	4.00e-02	4.00e-02	3.91e-01	0	*0*	3	*SCN3B; SCN4B; SLC4A4*	0/178	3/178	3/178
GO:0051899	membrane depolarization	3	178	16/3649	4.00e-02	4.00e-02	3.91e-01	1	*MLLT11*	2	*SCN3B; SCN4B*	1/178	2/178	3/178
GO:2000573	positive regulation of DNA biosynthetic process	3	178	16/3649	4.00e-02	4.00e-02	3.91e-01	1	*FGF2*	2	*CCT3; MYC*	1/178	2/178	3/178
GO:0006469	negative regulation of protein kinase activity	5	178	40/3649	4.30e-02	4.30e-02	4.18e-01	2	*ADIPOQ; FABP4*	3	*CHORDC1; DNAJA1; GADD45A*	2/178	3/178	5/178
GO:0016311	dephosphorylation	8	178	82/3649	4.48e-02	4.48e-02	4.23e-01	4	*MEF2C; NT5E; PPM1K; TGFB2*	4	*CASZ1; CPPED1; FBP2; NCEH1*	4/178	4/178	8/178
GO:0009266	response to temperature stimulus	4	178	28/3649	4.48e-02	4.48e-02	4.23e-01	0	*0*	4	*ACADM; ACADVL; CHORDC1; DNAJA1*	0/178	4/178	4/178
GO:0032869	cellular response to insulin stimulus	4	178	28/3649	4.48e-02	4.48e-02	4.23e-01	2	*ADIPOQ; INHBB*	2	*IGF1R; SLC39A14*	2/178	2/178	4/178
GO:0009888	tissue development	20	178	276/3649	4.53e-02	4.53e-02	4.23e-01	10	*ADIPOQ; ANKRD1; FGF2; MEF2C; MYF5; PI16; PTER; TDRD7; TGFB2; TUFT1*	10	*ACADM; ACADVL; ACTA2; ASPN; COL4A1; MGP; RILPL2; RIPOR2; SLC39A14; TAGLN*	10/178	10/178	20/178
GO:0051348	negative regulation of transferase activity	6	178	54/3649	4.58e-02	4.58e-02	4.23e-01	3	*ADIPOQ; FABP4; ORMDL3*	3	*CHORDC1; DNAJA1; GADD45A*	3/178	3/178	6/178
GO:0001503	ossification	7	178	68/3649	4.60e-02	4.60e-02	4.23e-01	4	*FGF2; MEF2C; MYF5; TGFB2*	3	*ASPN; MAP2K6; MGP*	4/178	3/178	7/178
GO:0050673	epithelial cell proliferation	7	178	68/3649	4.60e-02	4.60e-02	4.23e-01	4	*FGF2; MEF2C; NR1D1; TGFB2*	3	*AGTR1; MYC; THAP1*	4/178	3/178	7/178
GO:0045833	negative regulation of lipid metabolic process	3	178	17/3649	4.69e-02	4.69e-02	4.23e-01	0	*0*	3	*ACADVL; BRCA1; PLIN5*	0/178	3/178	3/178
GO:0051261	protein depolymerization	3	178	17/3649	4.69e-02	4.69e-02	4.23e-01	1	*MAP6D1*	2	*HSPA8; MICAL3*	1/178	2/178	3/178
GO:0061326	renal tubule development	3	178	17/3649	4.69e-02	4.69e-02	4.23e-01	2	*FGF2; MEF2C*	1	*COL4A1*	2/178	1/178	3/178
GO:1905954	positive regulation of lipid localization	3	178	17/3649	4.69e-02	4.69e-02	4.23e-01	1	*ADIPOQ*	2	*FITM2; PLIN5*	1/178	2/178	3/178
GO:0048880	sensory system development	5	178	41/3649	4.72e-02	4.72e-02	4.23e-01	4	*FGF2; MYF5; TDRD7; TGFB2*	1	*COL4A1*	4/178	1/178	5/178
GO:0071375	cellular response to peptide hormone stimulus	5	178	41/3649	4.72e-02	4.72e-02	4.23e-01	2	*ADIPOQ; INHBB*	3	*AGTR1; IGF1R; SLC39A14*	2/178	3/178	5/178
GO:0150063	visual system development	5	178	41/3649	4.72e-02	4.72e-02	4.23e-01	4	*FGF2; MYF5; TDRD7; TGFB2*	1	*COL4A1*	4/178	1/178	5/178
GO:0003013	circulatory system process	8	178	83/3649	4.76e-02	4.76e-02	4.24e-01	3	*ADIPOQ; NPR3; TGFB2*	5	*ACTA2; AGTR1; MAP2K6; SCN3B; SCN4B*	3/178	5/178	8/178

GO:0007507	heart development	7	178	69/3649	4.92e-02	4.92e-02	4.36e-01	6	*ANKRD1; ECE2; FGF2; MEF2C; PI16; TGFB2*	2	*ACADM; ECE2*	6/178	2/178	7/178
