# Supplementary material for: Early Weaning Possibly Increases the Activity of Lipogenic and Adipogenic Pathways in Intramuscular Adipose Tissue of Nellore Calves
Source: Metabolites. 2023 Sep 21;13(9):1028. doi: 10.3390/metabo13091028 (PMC10536964; doi:10.3390/metabo13091028)
Supplement: Supplementary file 1 [file metabolites-13-01028-s001.zip › Supplementary 4 - KEGG pathways - cópia.pdf]

1. Enriched KEGG pathways to DEG prospect in EW vs CW into time 1

ID	Description	count	terms	BgRatio	pvalue	p.adjust	qvalue	Count.UpReg	geneID.UpReg	Count.DownReg	geneID.DownReg	GeneRatio.Up	GeneRatio.Down	GeneRatio
bta05144	Malaria	3	14	61/9095	9.91e-05	9.91e-05	3.86e-03	3	*HBA1; HBB; LOC100300510*	0	0	3/14	0/14	3/14
bta05310	Asthma	2	14	39/9095	1.58e-03	1.58e-03	1.92e-02	2	*BOLA-DRB2; LOC100300510*	0	0	2/14	0/14	2/14
bta05143	African trypanosomiasis	2	14	44/9095	2.01e-03	2.01e-03	1.92e-02	2	*HBA1; HBB*	0	0	2/14	0/14	2/14
bta04514	Cell adhesion molecules	3	14	171/9095	2.04e-03	2.04e-03	1.92e-02	3	*BOLA-DRB2; LOC100300510; TIGIT*	0	0	3/14	0/14	3/14
bta04672	Intestinal immune network for IgA production	2	14	58/9095	3.46e-03	3.46e-03	1.92e-02	2	*BOLA-DRB2; LOC100300510*	0	0	2/14	0/14	2/14
bta05330	Allograft rejection	2	14	58/9095	3.46e-03	3.46e-03	1.92e-02	2	*BOLA-DRB2; LOC100300510*	0	0	2/14	0/14	2/14
bta04940	Type I diabetes mellitus	2	14	60/9095	3.70e-03	3.70e-03	1.92e-02	2	*BOLA-DRB2; LOC100300510*	0	0	2/14	0/14	2/14
bta05332	Graft-versus-host disease	2	14	66/9095	4.46e-03	4.46e-03	1.92e-02	2	*BOLA-DRB2; LOC100300510*	0	0	2/14	0/14	2/14
bta05321	Inflammatory bowel disease	2	14	71/9095	5.15e-03	5.15e-03	1.92e-02	2	*BOLA-DRB2; LOC100300510*	0	0	2/14	0/14	2/14
bta05166	Human T-cell leukemia virus 1 infection	3	14	238/9095	5.20e-03	5.20e-03	1.92e-02	3	*BOLA-DRB2; LOC100300510; ZFP36*	0	0	3/14	0/14	3/14
bta05320	Autoimmune thyroid disease	2	14	73/9095	5.43e-03	5.43e-03	1.92e-02	2	*BOLA-DRB2; LOC100300510*	0	0	2/14	0/14	2/14
bta05416	Viral myocarditis	2	14	78/9095	6.18e-03	6.18e-03	2.01e-02	2	*BOLA-DRB2; LOC100300510*	0	0	2/14	0/14	2/14
bta04612	Antigen processing and presentation	2	14	91/9095	8.33e-03	8.33e-03	2.50e-02	2	*BOLA-DRB2; LOC100300510*	0	0	2/14	0/14	2/14
bta04658	Th1 and Th2 cell differentiation	2	14	99/9095	9.80e-03	9.80e-03	2.73e-02	2	*BOLA-DRB2; LOC100300510*	0	0	2/14	0/14	2/14
bta05323	Rheumatoid arthritis	2	14	106/9095	1.12e-02	1.12e-02	2.77e-02	2	*BOLA-DRB2; LOC100300510*	0	0	2/14	0/14	2/14
bta05150	Staphylococcus aureus infection	2	14	107/9095	1.14e-02	1.14e-02	2.77e-02	2	*BOLA-DRB2; LOC100300510*	0	0	2/14	0/14	2/14
bta04659	Th17 cell differentiation	2	14	116/9095	1.33e-02	1.33e-02	3.04e-02	2	*BOLA-DRB2; LOC100300510*	0	0	2/14	0/14	2/14
bta04145	Phagosome	2	14	172/9095	2.79e-02	2.79e-02	6.03e-02	2	*BOLA-DRB2; RAB7B*	0	0	2/14	0/14	2/14
bta05322	Systemic lupus erythematosus	2	14	190/9095	3.35e-02	3.35e-02	6.87e-02	2	*BOLA-DRB2; LOC100300510*	0	0	2/14	0/14	2/14
bta05202	Transcriptional misregulation in cancer	2	14	200/9095	3.68e-02	3.68e-02	7.17e-02	1	*LOC100300510*	1	PER2	1/14	1/14	2/14
bta00640	Propanoate metabolism	1	14	31/9095	4.67e-02	4.67e-02	8.66e-02	0	*0*	1	DBT	0/14	1/14	1/14
bta05169	Epstein-Barr virus infection	2	14	235/9095	4.93e-02	4.93e-02	8.73e-02	2	*BOLA-DRB2; LOC100300510*	0	0	2/14	0/14	2/14

2. Enriched KEGG pathways to DEG prospect in EW vs CW into time 2

ID	Description	count	terms	BgRatio	pvalue	p.adjust	qvalue	Count.UpReg	geneID.UpReg	Count.DownReg	geneID.DownReg	GeneRatio.Up	GeneRatio.Down	GeneRatio
bta01212	*Fatty acid metabolism*	8	68	58/9095	9.12e-09	9.12e-09	1.81e-06	6	*ACACA; ACSL3; ELOVL6; FADS1; FASN; SCD*	2	*ACOX3; CPT1B*	6/68	2/68	8/68
bta03320	*PPAR signaling pathway*	8	68	84/9095	1.78e-07	1.78e-07	1.77e-05	4	*ACSL3; ADIPOQ; FABP4; SCD*	4	*ACOX3; CPT1B; PPARD; SLC27A5*	4/68	4/68	8/68
bta04152	*AMPK signaling pathway*	9	68	124/9095	3.04e-07	3.04e-07	2.02e-05	5	*ACACA; ADIPOQ; FASN; SCD; SREBF1*	4	*CPT1B; EIF4EBP1; PFKFB1; PFKFB2*	5/68	4/68	9/68
bta04936	*Alcoholic liver disease*	8	68	154/9095	1.76e-05	1.76e-05	8.76e-04	6	*ACACA; ADIPOQ; FASN; MAPK9; SCD; SREBF1*	2	*ACOX3; CPT1B*	6/68	2/68	8/68
bta01040	*Biosynthesis of unsaturated fatty acids*	4	68	30/9095	6.77e-05	6.77e-05	2.69e-03	3	*ELOVL6; FADS1; SCD*	1	*ACOX3*	3/68	1/68	4/68
bta00061	*Fatty acid biosynthesis*	3	68	18/9095	3.01e-04	3.01e-04	9.98e-03	3	*ACACA; ACSL3; FASN*	0	*0*	3/68	0/68	3/68
bta04920	*Adipocytokine signaling pathway*	4	68	72/9095	2.01e-03	2.01e-03	5.70e-02	3	*ACSL3; ADIPOQ; MAPK9*	1	*CPT1B*	3/68	1/68	4/68
bta04910	*Insulin signaling pathway*	5	68	138/9095	3.63e-03	3.63e-03	9.02e-02	4	*ACACA; FASN; MAPK9; SREBF1*	1	*EIF4EBP1*	4/68	1/68	5/68
bta00071	*Fatty acid degradation*	3	68	44/9095	4.25e-03	4.25e-03	9.40e-02	1	*ACSL3*	2	*ACOX3; CPT1B*	1/68	2/68	3/68
bta04961	*Endocrine and other factor-regulated calcium reabsorption*	3	68	51/9095	6.44e-03	6.44e-03	1.28e-01	2	*PRKCA; SLC8A3*	1	*DNM3*	2/68	1/68	3/68
bta04931	*Insulin resistance*	4	68	110/9095	9.11e-03	9.11e-03	1.65e-01	2	*MAPK9; SREBF1*	2	*CPT1B; SLC27A5*	2/68	2/68	4/68
bta05145	*Toxoplasmosis*	4	68	114/9095	1.03e-02	1.03e-02	1.71e-01	3	*BOLA-DRB2; LDLR; MAPK9*	1	*TGFB3*	3/68	1/68	4/68
bta05152	*Tuberculosis*	5	68	205/9095	1.83e-02	1.83e-02	2.81e-01	3	*BOLA-DRB2; CEBPG; MAPK9*	2	*RFXANK; TGFB3*	3/68	2/68	5/68
bta00640	*Propanoate metabolism*	2	68	31/9095	2.23e-02	2.23e-02	2.97e-01	1	*ACACA*	1	*ACOX3*	1/68	1/68	2/68
bta05207	*Chemical carcinogenesis - receptor activation*	5	68	216/9095	2.24e-02	2.24e-02	2.97e-01	3	*CHRNA3; KPNA4; PRKCA*	2	*ADRB2; EIF4EBP1*	3/68	2/68	5/68
bta04012	*ErbB signaling pathway*	3	68	84/9095	2.48e-02	2.48e-02	3.08e-01	2	*MAPK9; PRKCA*	1	*EIF4EBP1*	2/68	1/68	3/68
bta00051	*Fructose and mannose metabolism*	2	68	34/9095	2.65e-02	2.65e-02	3.10e-01	0	*0*	2	*PFKFB1; PFKFB2*	0/68	2/68	2/68
bta04211	*Longevity regulating pathway*	3	68	90/9095	2.96e-02	2.96e-02	3.27e-01	1	*ADIPOQ*	2	*ATG101; EIF4EBP1*	1/68	2/68	3/68
bta04925	*Aldosterone synthesis and secretion*	3	68	96/9095	3.49e-02	3.49e-02	3.31e-01	2	*LDLR; PRKCA*	1	*PRKD2*	2/68	1/68	3/68
bta05231	*Choline metabolism in cancer*	3	68	99/9095	3.77e-02	3.77e-02	3.31e-01	2	*MAPK9; PRKCA*	1	*EIF4EBP1*	2/68	1/68	3/68
bta04514	*Cell adhesion molecules*	4	68	171/9095	3.88e-02	3.88e-02	3.31e-01	2	*ALCAM; BOLA-DRB2*	2	*CDH15; LOC616254*	2/68	2/68	4/68
bta04020	*Calcium signaling pathway*	5	68	252/9095	3.98e-02	3.98e-02	3.31e-01	2	*PRKCA; SLC8A3*	3	*ADRB2; CD38; MYLK4*	2/68	3/68	5/68
bta04970	*Salivary secretion*	3	68	102/9095	4.06e-02	4.06e-02	3.31e-01	1	*PRKCA*	2	*ADRB2; CD38*	1/68	2/68	3/68
bta04922	*Glucagon signaling pathway*	3	68	103/9095	4.16e-02	4.16e-02	3.31e-01	1	*ACACA*	2	*CPT1B; PFKFB1*	1/68	2/68	3/68
bta04933	*AGE-RAGE signaling pathway in diabetic complications*	3	68	103/9095	4.16e-02	4.16e-02	3.31e-01	2	*MAPK9; PRKCA*	1	*TGFB3*	2/68	1/68	3/68
bta04930	*Type II diabetes mellitus*	2	68	46/9095	4.62e-02	4.62e-02	3.53e-01	2	*ADIPOQ; MAPK9*	0	*0*	2/68	0/68	2/68
bta04066	*HIF-1 signaling pathway*	3	68	110/9095	4.90e-02	4.90e-02	3.61e-01	1	*PRKCA*	2	*ANGPT2; EIF4EBP1*	1/68	2/68	3/68

3. Enriched KEGG pathways to DEG prospect in T2 vs T1 into conventional weaning group

ID	Description	count	terms	BgRatio	pvalue	p.adjust	qvalue	Count.UpReg	geneID.UpReg	Count.DownReg	geneID.DownReg	GeneRatio.Up	GeneRatio.Down	GeneRatio
bta05410	Hypertrophic cardiomyopathy	17	349	92/9095	5.86e-08	5.86e-08	1.57e-05	14	*ACTB; ACTG1; CACNA2D2; CACNB3; CACNG4; ITGA2B; ITGA6; ITGB6; SGCG; TGFB1; TGFB2; TGFB3; TNNT2; TPM4*	3	*EDN1; MYH7; RYR2*	14/349	3/349	17/349
bta05414	Dilated cardiomyopathy	16	349	99/9095	9.71e-07	9.71e-07	1.30e-04	14	*ACTB; ACTG1; CACNA2D2; CACNB3; CACNG4; ITGA2B; ITGA6; ITGB6; SGCG; TGFB1; TGFB2; TGFB3; TNNT2; TPM4*	2	*MYH7; RYR2*	14/349	2/349	16/349
bta05412	Arrhythmogenic right ventricular cardiomyopathy	12	349	76/9095	2.85e-05	2.85e-05	2.54e-03	11	*ACTB; ACTG1; CACNA2D2; CACNB3; CACNG4; GJA1; ITGA2B; ITGA6; ITGB6; SGCG; TCF7L1*	1	*RYR2*	11/349	1/349	12/349
bta04010	MAPK signaling pathway	25	349	292/9095	1.39e-04	1.39e-04	9.32e-03	17	*ANGPT2; CACNA1A; CACNA2D2; CACNB3; CACNG4; CD14; EPHA2; FGFR1; FGFR4; NR4A1; PAK1; PLA2G4B; RASGRP2; TGFB1; TGFB2; TGFB3; VEGFD*	8	*FGF1; FGF9; FLNC; HSPB1; MAP2K6; MAPKAPK3; RAPGEF2; RPS6KA3*	17/349	8/349	25/349
bta04350	TGF-beta signaling pathway	12	349	95/9095	2.61e-04	2.61e-04	1.40e-02	11	*CHRD; GREM1; ID1; ID3; INHBB; RGMA; SMAD6; SMAD7; TGFB1; TGFB2; TGFB3*	1	*ACVR2A*	11/349	1/349	12/349
bta04933	AGE-RAGE signaling pathway in diabetic complications	12	349	103/9095	5.55e-04	5.55e-04	2.48e-02	9	*AGER; CCND1; COL4A3; NOS3; SERPINE1; TGFB1; TGFB2; TGFB3; VEGFD*	3	*BCL2; DIAPH1; EDN1*	9/349	3/349	12/349
bta04015	Rap1 signaling pathway	19	349	217/9095	6.66e-04	6.66e-04	2.55e-02	14	*ACTB; ACTG1; ANGPT2; ARAP3; DOCK4; EPHA2; FGFR1; FGFR4; ID1; ITGA2B; PRKD2; RASGRP2; VASP; VEGFD*	5	*ENAH; FGF1; FGF9; MAP2K6; RAPGEF2*	14/349	5/349	19/349
bta04510	Focal adhesion	18	349	203/9095	7.92e-04	7.92e-04	2.65e-02	12	*ACTB; ACTG1; CCND1; COL4A3; ITGA2B; ITGA6; ITGB6; LAMA4; MYLK3; PAK1; VASP; VEGFD*	6	*ARHGAP35; BCL2; DIAPH1; FLNC; ROCK2; THBS2*	12/349	6/349	18/349
bta04330	Notch signaling pathway	8	349	58/9095	1.55e-03	1.55e-03	4.61e-02	8	*DLL1; DLL4; DTX3L; HEYL; LFNG; NOTCH1; NOTCH4; TLE3*	0	*0*	8/349	0/349	8/349
bta04921	Oxytocin signaling pathway	14	349	153/9095	2.21e-03	2.21e-03	5.90e-02	12	*ACTB; ACTG1; CACNA2D2; CACNB3; CACNG4; CAMK1; CCND1; CD38; MYLK3; NOS3; OXTR; PLA2G4B*	2	*ROCK2; RYR2*	12/349	2/349	14/349
bta04810	Regulation of actin cytoskeleton	18	349	228/9095	2.93e-03	2.93e-03	6.98e-02	11	*ACTB; ACTG1; C8G; FGFR1; FGFR4; ITGA2B; ITGA6; ITGB6; MYLK3; PAK1; SCIN*	7	*ARHGAP35; DIAPH1; ENAH; FGF1; FGF9; MYH11; ROCK2*	11/349	7/349	18/349
bta05222	Small cell lung cancer	10	349	94/9095	3.13e-03	3.13e-03	6.98e-02	6	*CCND1; COL4A3; ITGA2B; ITGA6; LAMA4; RXRG*	4	*APAF1; BCL2; CKS2; NFKBIA*	6/349	4/349	10/349
bta00062	Fatty acid elongation	5	349	29/9095	4.49e-03	4.49e-03	9.23e-02	3	*ACOT7; ELOVL5; ELOVL7*	4	*ACOT2; ACOT2; ACOT2; ACOT2*	3/349	4/349	5/349
bta01040	Biosynthesis of unsaturated fatty acids	5	349	30/9095	5.22e-03	5.22e-03	9.97e-02	3	*ACOT7; ELOVL5; ELOVL7*	4	*ACOT2; ACOT2; ACOT2; ACOT2*	3/349	4/349	5/349
bta04390	Hippo signaling pathway	13	349	156/9095	6.92e-03	6.92e-03	1.18e-01	12	*ACTB; ACTG1; CCND1; ID1; SERPINE1; SMAD7; SNAI2; TCF7L1; TGFB1; TGFB2; TGFB3; WNT9A*	1	*FGF1*	12/349	1/349	13/349
bta04611	Platelet activation	11	349	122/9095	7.09e-03	7.09e-03	1.18e-01	9	*ACTB; ACTG1; FCGR2A; ITGA2B; MYLK3; NOS3; PLA2G4B; RASGRP2; VASP*	2	*ARHGAP35; ROCK2*	9/349	2/349	11/349
bta04666	Fc gamma R-mediated phagocytosis	9	349	97/9095	1.19e-02	1.19e-02	1.88e-01	8	*FCGR2A; MARCKSL1; PAK1; PLA2G4B; PLD2; PLPP2; SCIN; VASP*	1	*GAB2*	8/349	1/349	9/349
bta05221	Acute myeloid leukemia	7	349	68/9095	1.50e-02	1.50e-02	2.23e-01	6	*CCND1; CD14; PER2; PML; PPARD; TCF7L1*	1	*ZBTB16*	6/349	1/349	7/349
bta00565	Ether lipid metabolism	6	349	54/9095	1.67e-02	1.67e-02	2.27e-01	5	*GDPD3; PLA2G4B; PLD2; PLPP2; TMEM86B*	1	*PLA2G7*	5/349	1/349	6/349
bta04919	Thyroid hormone signaling pathway	10	349	121/9095	1.77e-02	1.77e-02	2.27e-01	7	*ACTB; ACTG1; CCND1; NOTCH1; NOTCH4; PFKFB2; RXRG*	3	*ATP1B1; MYH7; RCAN2*	7/349	3/349	10/349
bta04142	Lysosome	11	349	139/9095	1.78e-02	1.78e-02	2.27e-01	4	*ASAH1; CTSK; GM2A; LITAF*	7	*AP4E1; ARSG; GNPTAB; IGF2R; NCOA7; SORT1; WDR7*	4/349	7/349	11/349
bta04530	Tight junction	13	349	178/9095	1.94e-02	1.94e-02	2.35e-01	8	*ACTB; ACTG1; CCND1; MARVELD2; MICALL2; MPDZ; RAB8B; VASP*	5	*MYH11; RAPGEF2; ROCK2; SYNPO; TUBA3E*	8/349	5/349	13/349
bta04360	Axon guidance	13	349	179/9095	2.02e-02	2.02e-02	2.35e-01	9	*EPHA2; PAK1; PLXNB3; RGMA; RND1; SEMA3F; SEMA4C; SEMA7A; UNC5B*	4	*ENAH; NTN3; PTCH1; ROCK2*	9/349	4/349	13/349

bta05132	Salmonella infection	17	349	261/9095	2.31e-02	2.31e-02	2.52e-01	8	*ACTB; ACTG1; CD14; MLKL; PAK1; PIK3C2B; TCF7L1; TUBB6*	9	*BCL2; FLNC; FYCO1; MAP2K6; NFKBIA; PLEKHM1; ROCK2; TUBA3E; VPS39*	8/349	9/349	17/349
bta05418	Fluid shear stress and atherosclerosis	11	349	145/9095	2.36e-02	2.36e-02	2.52e-01	7	*ACTB; ACTG1; ITGA2B; KLF2; NOS3; NPPC; PLAT*	4	*ACVR2A; BCL2; EDN1; MAP2K6*	7/349	4/349	11/349
bta04260	Cardiac muscle contraction	8	349	93/9095	2.61e-02	2.61e-02	2.68e-01	5	*CACNA2D2; CACNB3; CACNG4; TNNT2; TPM4*	3	*ATP1B1; MYH7; RYR2*	5/349	3/349	8/349
bta05224	Breast cancer	11	349	150/9095	2.93e-02	2.93e-02	2.75e-01	9	*CCND1; DLL1; DLL4; FGFR1; HEYL; NOTCH1; NOTCH4; TCF7L1; WNT9A*	2	*FGF1; FGF9*	9/349	2/349	11/349
bta05416	Viral myocarditis	7	349	78/9095	2.96e-02	2.96e-02	2.75e-01	6	*ACTB; ACTG1; CCND1; CD40; CD86; SGCG*	1	*MYH7*	6/349	1/349	7/349
bta00670	One carbon pool by folate	3	349	18/9095	2.98e-02	2.98e-02	2.75e-01	2	*AMT; MTHFR*	1	*MTR*	2/349	1/349	3/349
bta05145	Toxoplasmosis	9	349	114/9095	3.09e-02	3.09e-02	2.75e-01	6	*CD40; ITGA6; LAMA4; TGFB1; TGFB2; TGFB3*	3	*BCL2; MAP2K6; NFKBIA*	6/349	3/349	9/349
bta04020	Calcium signaling pathway	16	349	252/9095	3.31e-02	3.31e-02	2.79e-01	11	*CACNA1A; CAMK1; CD38; EDNRB; FGFR1; FGFR4; MYLK3; NOS3; OXTR; P2RX6; VEGFD*	5	*EDNRA; FGF1; FGF9; MCU; RYR2*	11/349	5/349	16/349
bta04115	p53 signaling pathway	7	349	80/9095	3.34e-02	3.34e-02	2.79e-01	4	*CCND1; RRM2; SERPINE1; SHISA5*	3	*APAF1; BCL2; SESN1*	4/349	3/349	7/349
bta04621	NOD-like receptor signaling pathway	13	349	194/9095	3.59e-02	3.59e-02	2.91e-01	9	*GBP4; GBP4; GBP5; IFI16; IRF7; LOC512486; LOC781710; OAS1Y; OAS2*	5	*BCL2; MCU; NAMPT; NFKBIA; TXNIP*	9/349	5/349	13/349
bta04071	Sphingolipid signaling pathway	9	349	120/9095	4.09e-02	4.09e-02	3.22e-01	5	*ASAH1; CERS1; NOS3; PLD2; SGPL1*	4	*ABCC1; BCL2; GAB2; ROCK2*	5/349	4/349	9/349
bta04270	Vascular smooth muscle contraction	10	349	140/9095	4.28e-02	4.28e-02	3.25e-01	3	*MYLK3; NPPC; PLA2G4B*	7	*EDN1; EDNRA; KCNMA1; KCNMB4; MYH11; PPP1R14A; ROCK2*	3/349	7/349	10/349
bta05162	Measles	11	349	160/9095	4.38e-02	4.38e-02	3.25e-01	8	*CCND1; DDX58; IFIH1; IRF7; MX1; MX2; OAS1Y; OAS2*	3	*APAF1; BCL2; NFKBIA*	8/349	3/349	11/349
bta04928	Parathyroid hormone synthesis, secretion and action	8	349	105/9095	4.84e-02	4.84e-02	3.43e-01	7	*FGFR1; MMP17; MMP25; NR4A2; PDE4C; PLD2; RXRG*	1	*BCL2*	7/349	1/349	8/349
bta04152	AMPK signaling pathway	9	349	124/9095	4.87e-02	4.87e-02	3.43e-01	3	*CCND1; PFKFB1; PFKFB2*	6	*ADIPOR2; CAB39; FOXO3; MLYCD; PFKFB4; ULK1*	3/349	6/349	9/349

4. Enriched KEGG pathways to DEG prospect in T2 vs T1 into early weaning group

ID	Description	count	terms	BgRatio	pvalue	p.adjust	qvalue	Count.UpReg	geneID.UpReg	Count.DownReg	geneID.DownReg	GeneRatio.Up	GeneRatio.Down	GeneRatio
bta01212	Fatty acid metabolism	16	270	58/9095	6.11e-12	6.11e-12	1.57e-09	7	ACACA; ELOVL6; FADS1; FADS2; FASN; HSD17B12; SCD	9	ACADM; ACADS; ACADVL; ACAT1; ACOX3; CPT1A; CPT1B; CPT2; HADHA	7/270	9/270	16/270
bta03320	PPAR signaling pathway	16	270	84/9095	2.44e-09	2.44e-09	3.13e-07	7	ADIPOQ; FABP4; FADS2; PCK2; PLIN1; RXRG; SCD	9	ACADM; ACOX3; ANGPTL4; CPT1A; CPT1B; CPT2; FABP3; PLIN5; SLC27A1	7/270	9/270	16/270
bta00650	Butanoate metabolism	8	270	27/9095	7.40e-07	7.40e-07	6.33e-05	5	AACS; ACSM1; ACSM5; BDH1; HMGCLL1	3	ACADS; ACAT1; HADHA	5/270	3/270	8/270
bta01040	Biosynthesis of unsaturated fatty acids	8	270	30/9095	1.80e-06	1.80e-06	1.16e-04	5	ELOVL6; FADS1; FADS2; HSD17B12; SCD	5	ACOT2; ACOT2; ACOT2; ACOT2; ACOX3	5/270	5/270	8/270
bta00071	Fatty acid degradation	9	270	44/9095	4.47e-06	4.47e-06	2.29e-04	0	0	9	ACADM; ACADS; ACADVL; ACAT1; ACOX3; CPT1A; CPT1B; CPT2; HADHA	0/270	9/270	9/270
bta04936	Alcoholic liver disease	16	270	154/9095	1.26e-05	1.26e-05	5.40e-04	6	ACACA; ADIPOQ; FASN; PRKAG3; SCD; SREBF1	10	ACADM; ACADVL; ACOX3; ADIPOR2; CPT1A; CPT1B; CXCL3; IL17RA; MAP2K6; MLYCD	6/270	10/270	16/270
bta04152	AMPK signaling pathway	14	270	124/9095	1.72e-05	1.72e-05	6.32e-04	7	ACACA; ADIPOQ; FASN; PCK2; PRKAG3; SCD; SREBF1	7	ADIPOR2; CPT1A; CPT1B; FBP2; IGF1R; MLYCD; RPTOR	7/270	7/270	14/270
bta00640	Propanoate metabolism	7	270	31/9095	2.68e-05	2.68e-05	8.61e-04	3	ACACA; ACSS2; DBT	4	ACADS; ACOX3; HADHA; MLYCD	3/270	4/270	7/270
bta00533	Glycosaminoglycan biosynthesis - keratan sulfate	5	270	15/9095	5.23e-05	5.23e-05	1.49e-03	3	B4GALT2; B4GALT4; CHST1	2	ST3GAL1; ST3GAL2	3/270	2/270	5/270
bta00280	Valine, leucine and isoleucine degradation	8	270	51/9095	1.14e-04	1.14e-04	2.94e-03	4	AACS; DBT; HMGCLL1; MCCC1	4	ACADM; ACADS; ACAT1; HADHA	4/270	4/270	8/270
bta04710	Circadian rhythm	6	270	35/9095	5.09e-04	5.09e-04	1.19e-02	5	DBP; NR1D1; NR1D2; PER2; PRKAG3	1	ARNTL	5/270	1/270	6/270
bta05202	Transcriptional misregulation in cancer	15	270	200/9095	8.88e-04	8.88e-04	1.90e-02	10	CCNT2; CDKN2C; ETV5; LOC536229; MEF2C; NGFR; NR4A3; PER2; PLAT; RXRG	5	BCL6; GADD45A; IGF1R; MYC; SIX1	10/270	5/270	15/270
bta04010	MAPK signaling pathway	19	270	292/9095	1.09e-03	1.09e-03	2.16e-02	9	CACNA1A; CACNA1G; CACNG4; FGF2; FLT4; MEF2C; NGFR; NTRK2; TGFB2	10	DUSP1; DUSP7; FLNC; GADD45A; HSPA8; IGF1R; MAP2K6; MAP3K13; MAPKAPK3; MYC	9/270	10/270	19/270
bta00062	Fatty acid elongation	5	270	29/9095	1.47e-03	1.47e-03	2.70e-02	2	ELOVL6; HSD17B12	5	ACOT2; ACOT2; ACOT2; ACOT2; HADHA	2/270	5/270	5/270
bta04931	Insulin resistance	9	270	110/9095	5.33e-03	5.33e-03	8.57e-02	4	PCK2; PPP1R3C; PRKAG3; SREBF1	5	CPT1A; CPT1B; PPARGC1B; PYGB; SLC27A1	4/270	5/270	9/270
bta04920	Adipocytokine signaling pathway	7	270	72/9095	5.34e-03	5.34e-03	8.57e-02	4	ADIPOQ; PCK2; PRKAG3; RXRG	3	ADIPOR2; CPT1A; CPT1B	4/270	3/270	7/270
bta04922	Glucagon signaling pathway	8	270	103/9095	1.14e-02	1.14e-02	1.72e-01	3	ACACA; PCK2; PRKAG3	5	CPT1A; CPT1B; FBP2; PYGB; SIK2	3/270	5/270	8/270
bta00380	Tryptophan metabolism	5	270	48/9095	1.34e-02	1.34e-02	1.92e-01	1	LOC104968656	4	ACAT1; DDC; HADHA; MAOA	1/270	4/270	5/270
bta04211	Longevity regulating pathway	7	270	90/9095	1.73e-02	1.73e-02	2.25e-01	3	ADIPOQ; PRKAG3; SESN3	4	ADIPOR2; IGF1R; RPTOR; SESN1	3/270	4/270	7/270
bta00410	beta-Alanine metabolism	4	270	34/9095	1.75e-02	1.75e-02	2.25e-01	0	0	4	ACADS; ACOX3; HADHA; MLYCD	0/270	4/270	4/270
bta00100	Steroid biosynthesis	3	270	20/9095	2.03e-02	2.03e-02	2.41e-01	2	CYP51A1; LSS	1	CYP2R1	2/270	1/270	3/270
bta05222	Small cell lung cancer	7	270	94/9095	2.15e-02	2.15e-02	2.41e-01	2	COL4A3; RXRG	5	BCL2; CKS2; COL4A1; GADD45A; MYC	2/270	5/270	7/270
bta04910	Insulin signaling pathway	9	270	138/9095	2.16e-02	2.16e-02	2.41e-01	6	ACACA; FASN; PCK2; PPP1R3C; PRKAG3; SREBF1	3	FBP2; PYGB; RPTOR	6/270	3/270	9/270
bta00760	Nicotinate and nicotinamide metabolism	4	270	37/9095	2.33e-02	2.33e-02	2.49e-01	2	NAPRT; NT5E	2	NAMPT; NMRK2	2/270	2/270	4/270

bta04964	Proximal tubule bicarbonate reclamation	3	270	22/9095	2.63e-02	2.63e-02	2.70e-01	2	*ATP1A4; PCK2*	1	*SLC4A4*	2/270	1/270	3/270
bta05418	Fluid shear stress and atherosclerosis	9	270	145/9095	2.86e-02	2.86e-02	2.82e-01	5	*BMPR1B; GSTM3; MEF2C; MGST1; PLAT*	4	*BCL2; DUSP1; HSP90AB1; MAP2K6*	5/270	4/270	9/270
bta04923	Regulation of lipolysis in adipocytes	5	270	59/9095	3.02e-02	3.02e-02	2.88e-01	3	*CIDEC; FABP4; PLIN1*	2	*ADORA1; MGLL*	3/270	2/270	5/270
bta00480	Glutathione metabolism	5	270	62/9095	3.64e-02	3.64e-02	3.34e-01	4	*ANPEP; GSS; GSTM3; MGST1*	1	*ODC1*	4/270	1/270	5/270
bta04614	Renin-angiotensin system	3	270	26/9095	4.07e-02	4.07e-02	3.49e-01	1	*ANPEP*	2	*AGTR1; LNPEP*	1/270	2/270	3/270
bta00620	Pyruvate metabolism	4	270	44/9095	4.07e-02	4.07e-02	3.49e-01	3	*ACACA; ACSS2; PCK2*	1	*ACAT1*	3/270	1/270	4/270
bta04977	Vitamin digestion and absorption	3	270	27/9095	4.48e-02	4.48e-02	3.71e-01	2	*SLC19A2; SLC46A1*	1	*LOC100337457*	2/270	1/270	3/270
bta01200	Carbon metabolism	7	270	112/9095	4.88e-02	4.88e-02	3.86e-01	3	*ACSS2; GLDC; PRPS2*	4	*ACADS; ACAT1; ACOX3; FBP2*	3/270	4/270	7/270
