# Supplementary material for: Early Weaning Possibly Increases the Activity of Lipogenic and Adipogenic Pathways in Intramuscular Adipose Tissue of Nellore Calves
Source: Metabolites. 2023 Sep 21;13(9):1028. doi: 10.3390/metabo13091028 (PMC10536964; doi:10.3390/metabo13091028)
Supplement: Supplementary file 1 [file metabolites-13-01028-s001.zip › Supplementary 5 - shared DEGs .pdf]

Contrasts	Shared Genes Differentially Expressed
EW.vs.CW_M2 vs M2.vs.M1_EW	*SCD; FADS1; ACACA; FCGRT; SREBF1; NR1D1; CORO1C; LSS; CPT1B; CFAP70; KCNA5; MIR133A-2; KLF13; CSRP2; DYNLL1; MAMSTR; CHRNA3; FASN; ACOX3; SNAI3; MYH7B; ZNF385B; KCNQ4; CRELD2; ADIPOQ; DYNC2I2; GRIP2; MYLK4; PEG3; PRL; HSPH1; MPDZ; ELOVL6; FABP4*
EW.vs.CW_M1 vs M2.vs.M1_CW	*BOLA-DRB2; MAMSTR*
M2.vs.M1_EW vs M2.vs.M1_CW	*RBM3; PPM1K; TACC2; UNG; MYH7B; HSF4; PER2; MICAL3; NIPSNAP1; EYA4; SIK3; MLYCD; CTF1; TSC22D1; MGP; LOC508916; NPTX1; ZBTB7C; C1QTNF3; MAPKAPK3; CCT3; CCDC159; LMOD3; DPF3; C8G; RCSD1; SLC25A29; STBD1; PLPPR2; NAMPT; BCL2; ARHGAP35; SNAI3; RBFOX2; DDX10; FITM2; EIF4A2; CACNA1A; MIR12060; UGP2; SLC12A2; CCDC86; ATXN7L2; DNAJC1; MIR29C; CCDC15; ORMDL3; ACOT2; HOXD9; MAMSTR; GPATCH8; SLC9A5; RXRG; SPSB2; ADAM19; RNPC3; METTL7B; ARRDC1; CERS1; ASB18; VGLL2; DKK2; VWA8; CPT2; MAP2K6; TBC1D8; BTNL9; IQCA1L; CROCC; HDAC9; KIAA1671; SESN1; FBXO44; KALRN; CHRNE; MPDZ; SH3BP4; ESCO1; CKS2; IFT81; ETNPPL; PGRMC2; CCDC190; SCN1A; WDR7; ACSM5; PLA2G7; PARP3; WIPF3; SGCG; SIK2; ADCK1; ESRRG; WBP1L; ERFE; RHBDF1; ARSG; RBFA; SMOC1; PPP1R1B; IGF2R; DIPK1A; MTHFR; SLC7A8; SLC43A2; TMED8; ANK3; MIR1-2; ART3; MMRN2; LY6G5B; CNKSR2; MSS51; UCP3; VWA1; NCEH1; SPATA5; NNAT; CDC14A; DDAH2; GIPC3; TGFB2; MIR133A-2; ABCC5; PAPLN; ZNF385C; IPP; INHBB; LRRN1; GNPTAB; KLHDC8B; PLAT; ECE2; HIPK2; PLEKHA6; ARPP21; COL4A3; CACNG4; ZNF641; FLNC; RUNDC1; C19H17orf113; NSMF; CTXN3; PDK4; LOC112441908; TUFT1; SHISA2; GRIP2; CBX6; LNPEP; MIR29E; BTBD11; SAMD11; MYOM3; ADIPOR2; NIPAL3; LOC100337457; CHST1; KIF3C; SLC6A1; KIF4A; PTER; CFAP70; ADAMTS9; CEBPD; PLXDC1; RFX2; PVR; OLFML2B; MIR133A-1; ITGB6; DDR1; WEE1; NECAB3; TECRL; CYP2R1; KLHL40; GLIS2; UCK2; CCDC28B; NR4A3; RCAN2; RNF168; PIGZ*
